# A global *Staphylococcus aureus* proteome resource applied to the *in vivo* characterization of host-pathogen interactions

**DOI:** 10.1038/s41598-017-10059-w

**Published:** 2017-09-08

**Authors:** Stephan Michalik, Maren Depke, Annette Murr, Manuela Gesell Salazar, Ulrike Kusebauch, Zhi Sun, Tanja C. Meyer, Kristin Surmann, Henrike Pförtner, Petra Hildebrandt, Stefan Weiss, Laura Marcela Palma Medina, Melanie Gutjahr, Elke Hammer, Dörte Becher, Thomas Pribyl, Sven Hammerschmidt, Eric W. Deutsch, Samuel L. Bader, Michael Hecker, Robert L. Moritz, Ulrike Mäder, Uwe Völker, Frank Schmidt

**Affiliations:** 1 Interfaculty Institute for Genetics and Functional Genomics, Department of Functional Genomics, University Medicine Greifswald, Greifswald, Germany; 2 Institute for Microbiology, Ernst-Moritz-Arndt-University, Greifswald, Germany; 3 Interfaculty Institute for Genetics and Functional Genomics, Department Genetics of Microorganisms, Ernst-Moritz-Arndt-University, Greifswald, Germany; 4 Institute for Systems Biology, Seattle, WA USA; 5 ZIK-FunGene, Interfaculty Institute for Genetics and Functional Genomics, Department of Functional Genomics, University Medicine Greifswald, Greifswald, Germany

## Abstract

Data-independent acquisition mass spectrometry promises higher performance in terms of quantification and reproducibility compared to data-dependent acquisition mass spectrometry methods. To enable high-accuracy quantification of *Staphylococcus aureus* proteins, we have developed a global ion library for data-independent acquisition approaches employing high-resolution time of flight or Orbitrap instruments for this human pathogen. We applied this ion library resource to investigate the time-resolved adaptation of *S. aureus* to the intracellular niche in human bronchial epithelial cells and in a murine pneumonia model. In epithelial cells, abundance changes for more than 400 *S. aureus* proteins were quantified, revealing, e.g., the precise temporal regulation of the SigB-dependent stress response and differential regulation of translation, fermentation, and amino acid biosynthesis. Using an *in vivo* murine pneumonia model, our data-independent acquisition quantification analysis revealed for the first time the *in vivo* proteome adaptation of *S. aureus*. From approximately 2.15 × 10^5^ *S. aureus* cells, 578 proteins were identified. Increased abundance of proteins required for oxidative stress response, amino acid biosynthesis, and fermentation together with decreased abundance of ribosomal proteins and nucleotide reductase NrdEF was observed in post-infection samples compared to the pre-infection state.

## Introduction

The pathogen *Staphylococcus aureus* is a major health threat, both in the general population and in the hospital environment. Although the bacterium can be a harmless nasal or skin commensal in part of the population^1, 2^, *S. aureus* can cause a variety of diseases as an opportunistic pathogen^3^. *S. aureus* strains resistant to both standard antibiotics and to last-resort antibiotics such as vancomycin have been reported^4^. Vaccination against *S. aureus* infection has been successful in rabbit models^5^, but vaccination trials in humans have thus far failed^6^. *S. aureus* is not only an extracellular pathogen; it can also bypass the host immune system and therapeutic countermeasures by entering human cells via phagocytosis. Intracellular *S. aureus* is extremely durable and resistant, making it difficult to treat ultimately causing relapse of infections^7^.

The investigation of bacterial adaption is important for the discovery of new anti-staphylococcal strategies. During infection the interplay between host and pathogen is reflected in a changing protein composition. Until recently, only transcriptomic studies have been used to quantify gene expression of infection models. However, transcriptional profiling fails to provide information on post-transcriptional regulation, post-translational modification or protein localization. Although proteomic studies using mass spectrometry MS have partially met these requirements, there is the need to extend proteomics to provide highly reproducible, quantitative, sensitive, and comprehensive analysis with a single measurement of complex samples to enable the delineation of biological interactions relevant for infection.

Qualitative and quantitative proteomics techniques range from classical gel-based (two-dimensional gel electrophoresis (2-DE)) to more sensitive gel-free approaches, including protein or peptide labeling for relative quantitation. Modern gel-free mass spectrometry-based techniques in combination with label-free quantitation are superior to 2-DE in terms of throughput and sensitivity. The reasons for such significant improvements using gel-free MS are due to developments in bottom-up proteomics, where a protein sample is proteolytically digested into peptides typically with trypsin and analyzed by reversed-phase high-performance liquid chromatography (RP-HPLC) separation coupled to a mass spectrometer by an electrospray interface. The most common mass spectrometric method is data-dependent acquisition (DDA), where the most abundant peptide ion precursors (“TopN”) from a survey scan (MS1 spectrum) are isolated individually and fragmented resulting in an MS/MS or MS2 spectrum. DDA data are analyzed by searching the MS1 and corresponding MS2 masses using sequence database search engine tools (e.g., Comet^8^) against a species-specific protein sequence database. The search engine performs an *in silico* digestion of the protein sequence database based on the selected protease to assign putative peptide ion identifications to each MS2 spectrum. The spectral matching results are aggregated in a list of peptide identifications. The drawback of this method is the stochastic process by which ions are selected, coupled with the fact that the number of different ions that can be selected per unit time is often smaller than the number of different ions entering the instrument.

In recent years, further technical improvements of MS instruments and data analysis methods have enabled *in vitro* studies in which roughly 1,700 proteins of the *S. aureus* strain COL were identified by DDA; this corresponds to 65% of the predicted open reading frames (ORFs)^9^. However, these high protein identification rates were only possible by combining data from different samples, the use of fractionation techniques, and the separate analysis of different bacterial compartments. This required a large amount of instrument time, which is not compatible with the analysis of large samples series or with the low material quantities available from *in vivo* infection experiments.

Thus far, we have achieved the identification of ~1,450 *S. aureus* proteins using 12 separate analyses, each from 2 × 10^6^ sorted bacterial cells extracted from cell culture infection samples^10^, whereas in a single analysis, identification rates yield on average only 1,109 proteins (+/−56 proteins).

Innovative advancements in MS methods and instruments promise to render such exhaustive and time-consuming efforts unnecessary in the future. Recently introduced data-independent acquisition mass spectrometry (DIA-MS) methods do not rely on selecting the “TopN” most abundant peptide precursor ions for fragmentation. Rather, the techniques record multiplexed fragmentation spectra of all peptide precursor ions within given sequential mass windows. DIA has the advantage of collecting periodic spectral data for all peptide ions entering the instrument. Thus, it is a parallel, unbiased, and continuous process, in contrast to the DDA method, which is a serial, biased, and discontinuous process. DIA enables quantitation with high reproducibility^11^ similar to RNA transcriptomics arrays. However, the prerequisite for the most common analysis workflow of DIA data is the existence of a peptide fragment ion spectral library^12, 13^, which enables robust identification of collisional-induced fragmentation of peptide ions based on these fragment ion signatures derived from either previously collected data from traditional DDA workflows or from the data contained within the current dataset.

## Results and Discussion

### Generation of an *S. aureus* proteome DIA ion library

We developed an *S. aureus* PeptideAtlas (www.peptideatlas.org) from a comprehensive set of data (Supplemental Material 2.1, Supplemental Table 1) as a prerequisite for the generation of a peptide ion fragment spectral library. The *S. aureus* PeptideAtlas build comprises an extensive collection of high-accuracy MS/MS data sets acquired in a uniform manner, and contains an extensive collection of detected proteotypic peptides. A special feature of bacteria is the high variation in the proteome in response to environmental cues. Therefore, sample sets covering the different environmental conditions encountered in nature and extensive proteome fractionation are required for a comprehensive coverage of the bacterial proteome in the PeptideAtlas (Supplemental Material 2.2).

We have integrated our high-resolution MS/MS data sets (Supplemental Table 1) measured with standardized retention time peptides (iRT)^14^ into a very large fragment spectral ion library generated from data without iRT spike-in peptides^15^. To achieve this, the peptides of the standard ion library (12 DDA measurements with iRT-peptides, Q Exactive^TM^ and TripleTOF**®** 5600^+^) were used as “retention time landmarks” to align the RTs of the high-resolution MS data of 152 proteome measurements without iRT-peptides (Supplemental Material 2.3). The resulting DIA ion library consists of 2,057 proteins with 25,664 peptides with a length of 7 to 49 amino acids. This final ion library represents approximately 72% of the theoretical *S. aureus* proteome and is publicly available at http://www.swathatlas.org. The remaining 28% of the *S. aureus* proteome was not detected either because these proteins are not expressed under the conditions tested, are in too low abundance to be detected by DDA, or have only peptides with physiochemical properties, including hydrophobicity, hydrophilicity, isoelectric point or length, that cannot be readily detected by MS analysis. By using prediction tools such as CHEMscore^16^, CONSeQuence^17^ or DetectabilityPredictor^18^, which consider such physiochemical properties to calculate the detectability of peptides, it is found that a part of the proteome of *S. aureus* is not accessible by classical MS approaches^15^. To achieve greater proteome coverage of *S. aureus*, additional fractionation or enrichment methods, different enzyme combinations for protein digestion, or alternative MS methods such as top down proteomics must be applied in combination with cultivation of the pathogen under a wider array of different physiological conditions.

**Figure 1:**
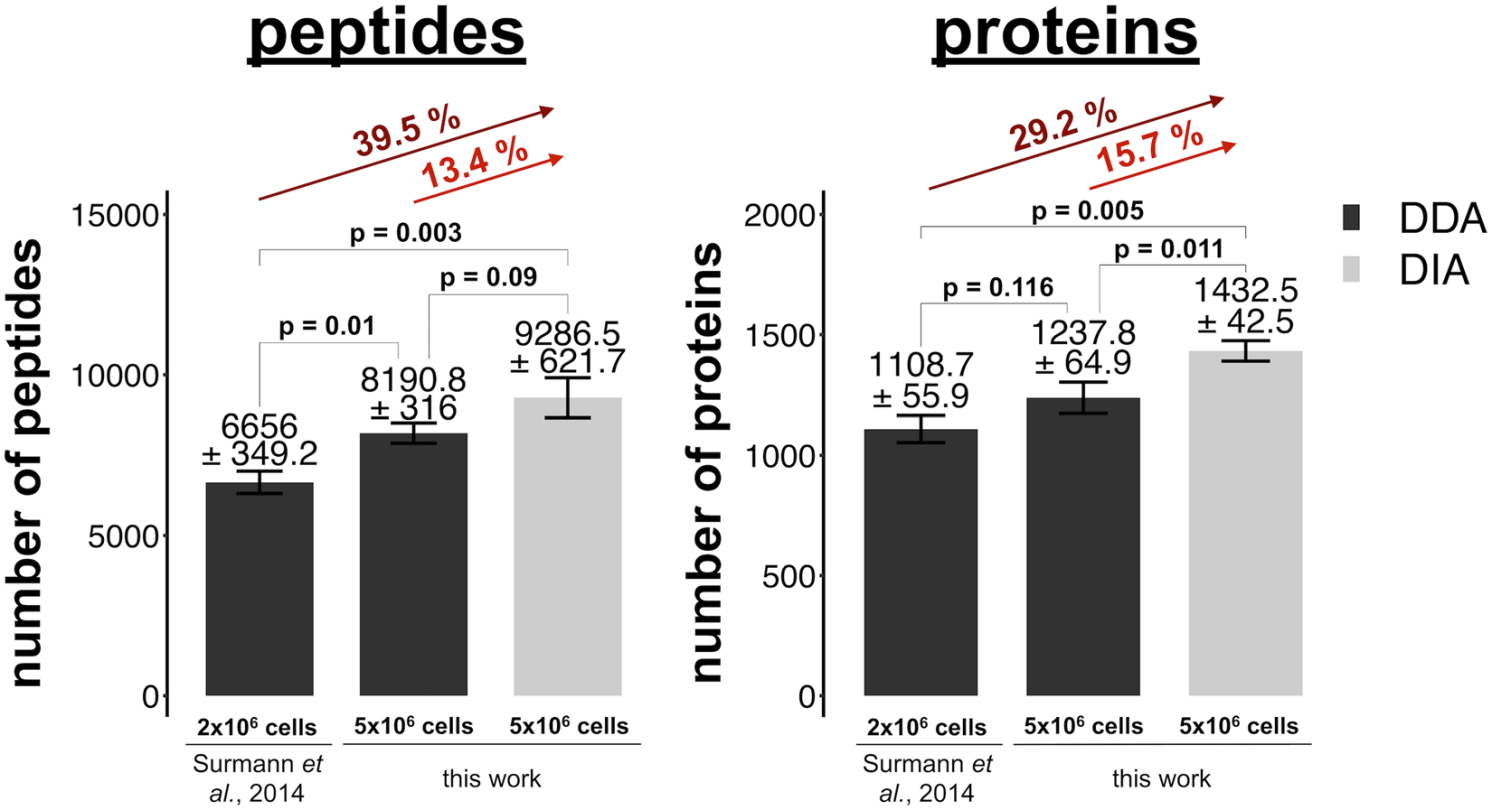
Characterization of the generated *S. aureus* ion library. Number of identifications (DDA-MS and DIA-MS). The DDA measurements of Surmann *et al*.^10^ generated from 2 × 10^6^ *S. aureus* cells are compared with the DDA-MS and DIA-MS measurements from 5 × 10^6^ *S. aureus* cells. The bar charts show the mean and standard deviation of identified peptides and proteins per single run at an FDR of 0.001. The identification rates were compared with two-sample two-sided student t-test using the Bonferroni correction for multiple testing.

The *S. aureus* spectral library developed here is a highly valuable proteomic resource and an essential basis for several targeted MS approaches such as Selected Reaction Monitoring (SRM), Parallel Reaction Monitoring (PRM) and DIA (e.g., SWATH^TM^, HRM-MS^TM^) to perform relative and absolute quantification of proteins. Furthermore, shotgun data analyses using peptide fragment spectral libraries and spectral library search tools such as SpectraST rely on high-quality spectral data and can outperform sequence-based database searches in terms of speed, sensitivity, and specificity^19^. In the present study, we identified and quantified on average with DIA-MS 1,433 proteins (+/−43 proteins) in a single run, which is 29.2% more than previously by DDA-MS analyses (Fig. 1). In total, we quantified 1,750 *S. aureus* proteins over 19 measurements in an S9 bronchial epithelial cell infection model. SRM is currently the gold standard for high-quality peptide quantification across multiple samples due to its high sensitivity, specificity, and wide dynamic range^20^. It relies on MS-generated prior-knowledge such as multiple precursor and fragment masses with known relative intensities in combination with the retention time of the targeted peptides. The *S. aureus* PeptideAtlas is a valuable proteome repository, facilitating the design of targeted proteome analysis approaches and extensive proteome quantitation.

### *S. aureus* proteome DIA ion library – range of applications

It is well known that the pathogen *S. aureus* possesses a high genomic variability, and currently there are 47 sequenced and annotated strains available in public databases. To investigate if our recently developed fragment ion library, which is based upon the *S. aureus* strain HG001, can also be used to analyze these additional 46 strains, we generated a phylogenetic tree based on each strain’s proteome and performed an *in-silico* digestion of each strain. Finally, we compared the resulting theoretical peptide sequences with the peptides in the fragment ion library. Approximately 80% of the peptides contained in our fragment ion library were observed in nearly all strains (Supplemental Fig. 1) except for *S. aureus* MSHR1132 with a coverage of approximately 60%, the reason for that is not completely understood.

The *S. aureus* fragment ion library constitutes a core set of assays which provide an excellent starting point for use with strains other than *S. aureus* HG001. However, for addressing variable parts of the proteome the fragment ion library would need additional proteomic data covering these strain-specific sets of protein variants.

Furthermore, we investigated the cross-device usability of DIA-MS assays generated with multiple mass spectrometer types (i.e., SCIEX TripleTOF**®** 5600^+^ and Thermo Q Exactive^TM^ instruments). Our data suggest that DIA-MS analysis can be performed with our *S. aureus* fragment ion library on both devices, and that the ion library is well-suited for correlation and cross-lab studies, underlining the benefit of an established, well-characterized fragment ion library for the scientific community (Supplemental Material 2.4). To address sensitivity afforded by different instruments, a thorough analysis of the dynamic range of the DIA-MS measurements of *S. aureus* protein samples revealed the necessity of applying at least 1,000 ng of digested protein or peptide sample material from approximately 1 × 10^6^ *S. aureus* cells (or more) on a Q Exactive^TM^ mass spectrometer for DIA-MS analysis (Supplemental Material 2.5).

### Human proteome interference in *S. aureus* infection experiments

**Figure 2:**
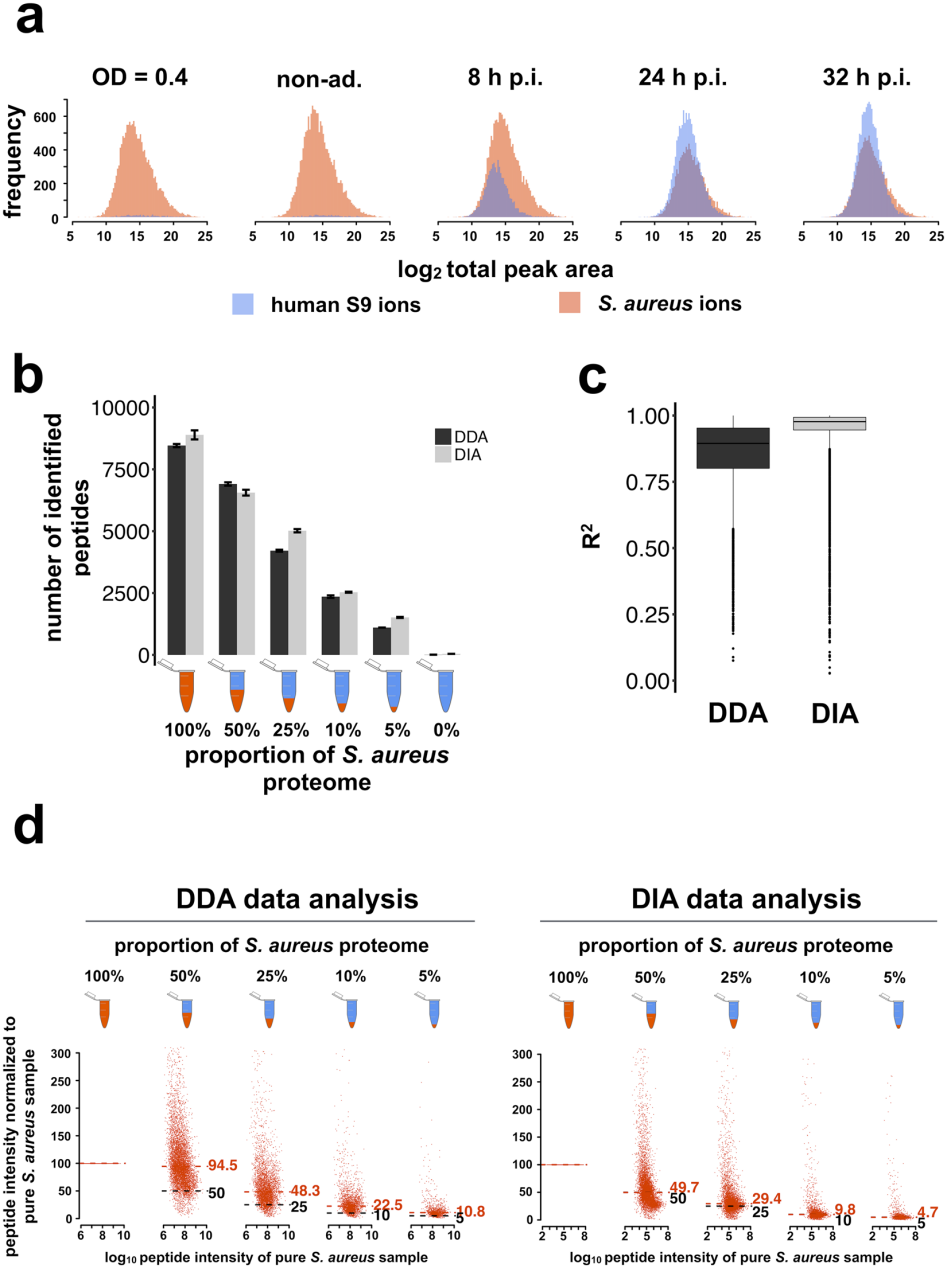
Comparison of peptide detection accuracy between DDA and DIA. (**a**) Histograms of the log_2_ total peak area of ions for *S. aureus* and for S9 human bronchial epithelial cells in the samples from the cell infection experiment. (**b**) The number of identified *S. aureus* peptides in the different proteome mixtures is presented in the barplot (DIA [Q-value < 0.001]; DDA [FDR_PSM_ < 0.001]). The error bars indicate the variance over the two technical replicates. (**c**) Normalized DIA and DDA peptide intensities were used for a linear regression modeling for each peptide against the expected values (100%, 50%, 25%, 10%, 5%). The resulting coefficients of determination (R^2^) represent the goodness of the fit. The closer R^2^ is to 1 the better is the goodness of regression. (**d**) The DIA and DDA peptide intensities of the different proteome mixture samples were normalized to the pure *S. aureus* sample for each peptide (100%), and single normalized peptide intensities were displayed in a scatterplot for each sample as a fraction of the peptide intensity of the pure *S. aureus* sample. The median of the normalized peptide intensities is displayed by the red number and dashed line. The expected median is displayed by the black number and dashed line.

The goal of the development of DIA-MS methods and the generation of databases such as the *S. aureus* PeptideAtlas described here is to provide tools and resources to analyze the adaptation reactions and pathophysiology of *S. aureus* as comprehensively as possible. This is especially important for infection-relevant settings, either in cell culture or in *in vivo* models. In contrast to classical *in vitro* shake flask experiments, both experimental settings present the challenge of intermixed host cell material, which can contaminate the bacterial samples even when purification strategies like cell sorting are applied (Fig. 2a). Host proteins in samples could interfere during the MS analysis and may cause problems in the quantification, because contaminating host peptides can have similar precursor and product ion mass values as the peptides targeted from the pathogen.

**Figure 3:**
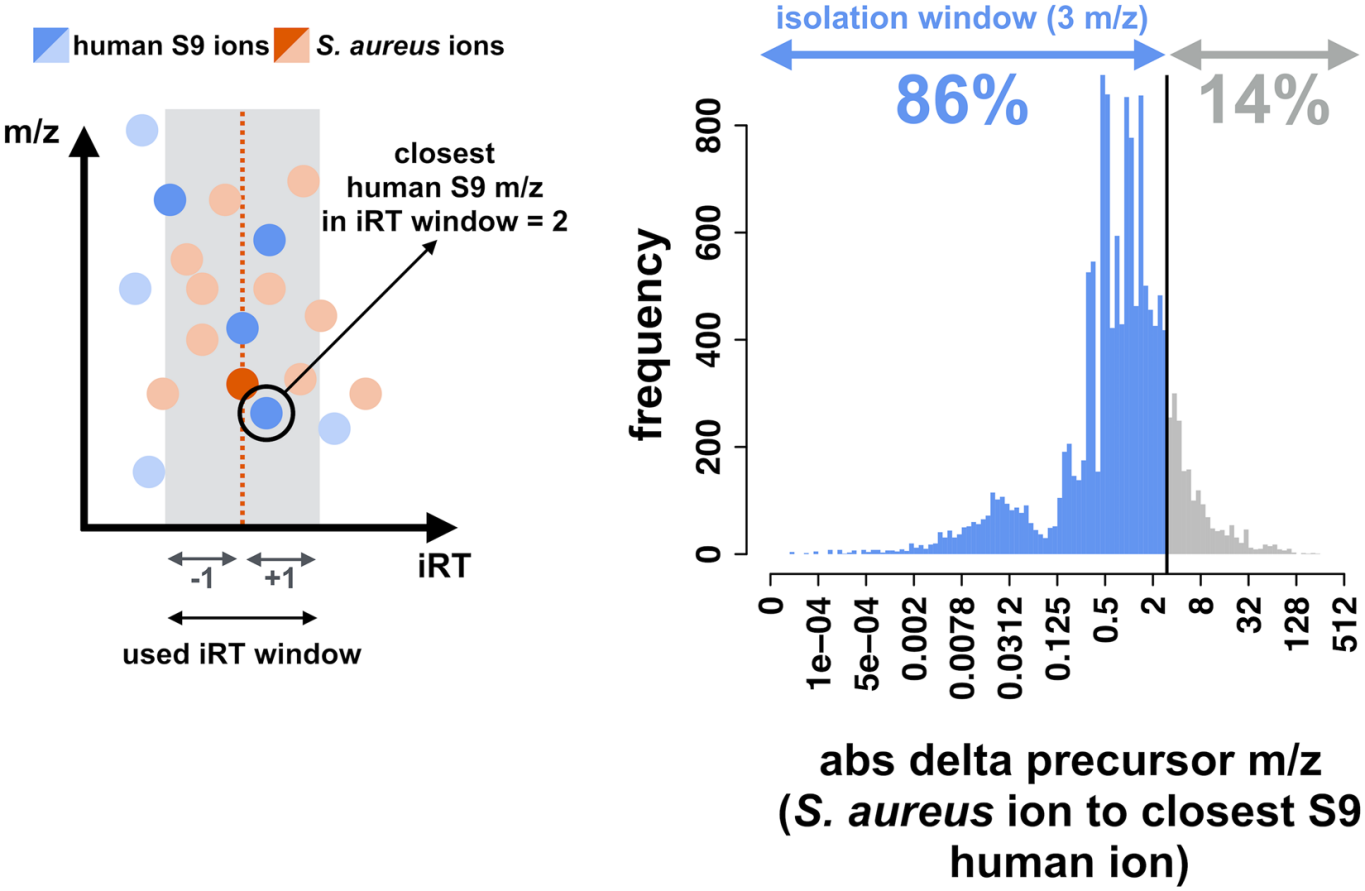
Determination of contaminating ion interference. The scheme depicts the approach for searching for the closest human precursor mass over charge (*m*/*z*) interference from the DIA ion identifications for each identified *S. aureus* ion. The search was performed in an iRT window of 2 minutes. The histogram displays the absolute difference in precursor mass over charge (abs delta precursor *m/z*) from each identified *S. aureus* ion to the closest human ion found. The proportion of *S. aureus* ions having an interfering human ion in the isolation window used in the DDA method (3 *m*/*z*) is colored in blue (below 3 *m*/*z*) and others are colored gray (above 3 *m*/*z*).

To test the host protein sample contamination interference, defined mixtures of S9 human cell protein and *S. aureus* protein extracts were prepared, measured in DDA-MS and DIA-MS mode, and analyzed with MaxQuant (DDA; FDR 0.001) and Spectronaut^TM^ (DIA; FDR 0.001), respectively (Fig. 2b). The numbers of *S. aureus* peptide identifications obtained in DDA-MS or DIA-MS analysis continuously followed the mixture gradient (Fig. 2b). Despite comparable numbers of peptide identifications, the coefficient of determination of the linear regression model (x = dilution; y = peptide intensity normalized to pure *S. aureus* sample) was significantly closer to a perfect linear model fit in the DIA-MS approach compared to the DDA-MS approach (Fig. 2c). The median of the normalized peptide intensities was appreciably closer to the expected value of the proteome dilution in the DIA-MS approach, whereas the DDA-MS approach showed a nearly 2-fold deviation from the expected value (Fig. 2d). In the DDA-MS approach, the ions were selected in an isolation window of 3 *m/z*, which was specified in the method. To determine if human peptide ions in the mixture were interfering inside the isolation window, we used the ions detected in the DIA-MS approach and searched for the closest human precursor *m/z* in an iRT window of ±1 min for every detected *S. aureus* ion. The *S. aureus* and closest human precursor *m/z* were used to calculate the difference in precursor *m/z*, and as a result, 86% of the *S. aureus* ions were contaminated by an interfering human precursor inside the isolation window of 3 *m/z* in the DDA data (Fig. 3).

In summary, only minor interference from contaminating host material was detected when using DIA-MS compared to high interferences on DDA MS1-based quantification and, therefore, DIA-MS analysis and the ion library generated in this study can be preferentially applied to investigate infection-related models or proteome mixtures, further emphasizing the value of providing publicly available resources such as the *S. aureus* PeptideAtlas (www.peptideatlas.org) and SWATHAtlas (www.swathatlas.org).

### S9 bronchial epithelial cell infection model

**Figure 4:**
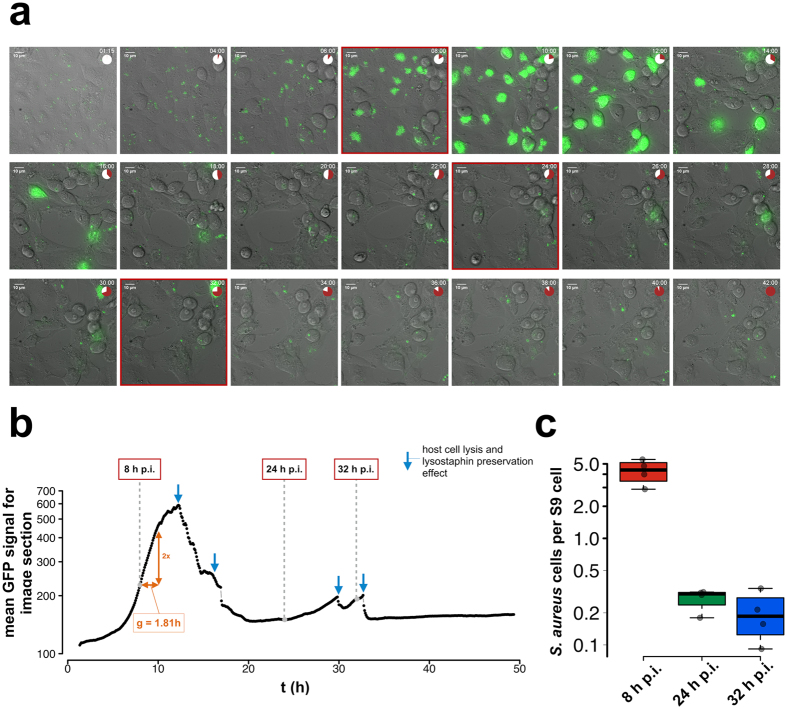
Live-cell imaging of *S. aureus* infecting S9 human bronchial epithelial cells. **(a)** The image montage summarizes the infection process over time taken from the original live-cell imaging movie (Supplemental Movie S1). The red circle sector shows the sampling time points. (**b**) Quantification of the mean GFP signal over the image section summarizes the number of GFP-carrying *S. aureus* cells of the image. A strong decrease in the GFP intensities, which is associated with the clearance of extracellular pathogen after host cell lysis by lysostaphin constantly present in the medium, is marked with a blue arrow in the plot. **(c)** The boxplot depicts the *S. aureus* cell number per S9 human bronchial epithelial cell over the sampling time points.

An S9 bronchial epithelial cell culture infection model was used to investigate effects during the encounter with the host and internalization of bacteria into host cells. The cell culture model aims to mimic processes of human infection. To gain insight into the intracellular lifestyle of *S. aureus*, especially in the lung epithelial cell environment, several cell-line infection studies were carried out. While previous studies^10, 21–23^ only focused on the very first hours (up to 8 h) post-infection (p.i.), which represent the initial adaptation to the intracellular milieu and initial intracellular growth, we also included later time points (24 h and 32 h p.i.) in the S9 bronchial epithelial cell infection model. In order to visually follow the pathogen over time inside host cells, GFP-labeled *S. aureus* HG001 was used^21, 24^ for live-cell imaging (Fig. 4a, Supplemental Movie S1). During the first hours of intracellular life, the pathogen adapted to its new environment and reached a maximal intracellular growth rate (estimated generation time = 1.81 h) between 7 and 10 h p.i., which was visualized by the quantified average GFP signal over time with a peak_max_ at approximately 12 h p.i., leading to killing of host cells after that point in time (Fig. 4b).

The extracellular medium of the cell culture system contained lysostaphin to eliminate *S. aureus* released from destroyed host cells and to preserve the cell culture, which enabled monitoring of strictly intracellular *S. aureus* over longer periods of time. The lysostaphin-mediated killing of *S. aureus* cells, which were released to the culture medium after host cell lysis, resulted in a sudden decrease of mean GFP intensity as shown in the live-cell imaging movie and a corresponding decrease of the count of internalized *S. aureus* per host cell (Fig. 4b,c). Mainly non-growing, intracellularly surviving bacteria were observed in low amounts after 12 h p.i.

**Figure 5:**
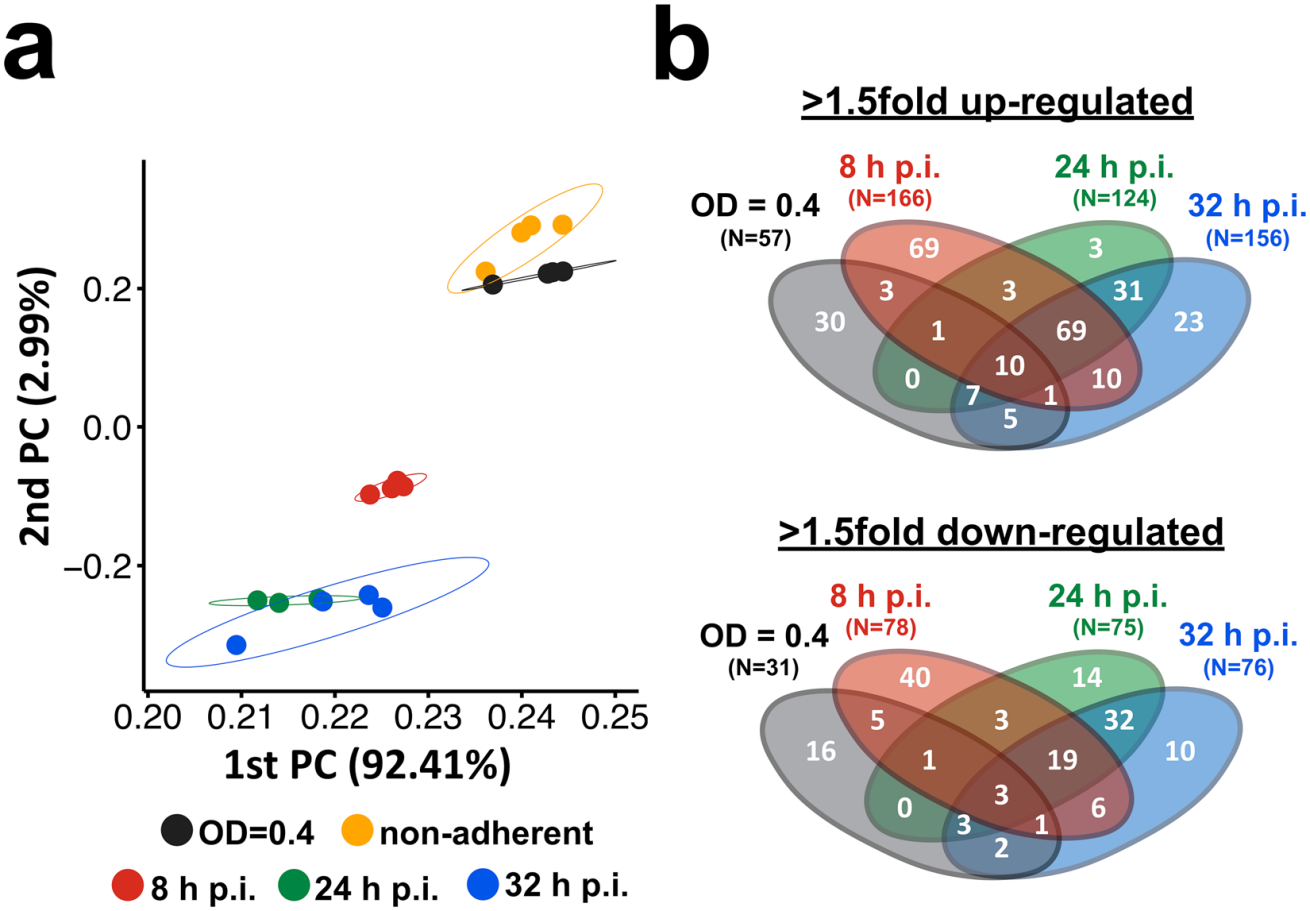
Global sample characterization and comparison of regulated proteins in S9 human bronchial epithelial cell infection experiments. (**a**) A principle component analysis (PCA) plot of the total peak area over the assays is shown for all samples from the S9 human bronchial epithelial cell infection experiment. The axis labeling lists variance explained by the corresponding principle components. The ellipses indicate the calculated 95% probability region for a bivariate normal distribution with an average center of groups. Samples were measured in biological quadruplicates. A replicate of the 24 h p.i. sample was excluded due to technical reasons. (**b**) The Venn diagrams summarize the significantly 1.5-fold down- or up-regulated proteins of all samples (normalized to the non-adherent control sample).

**Figure 6:**
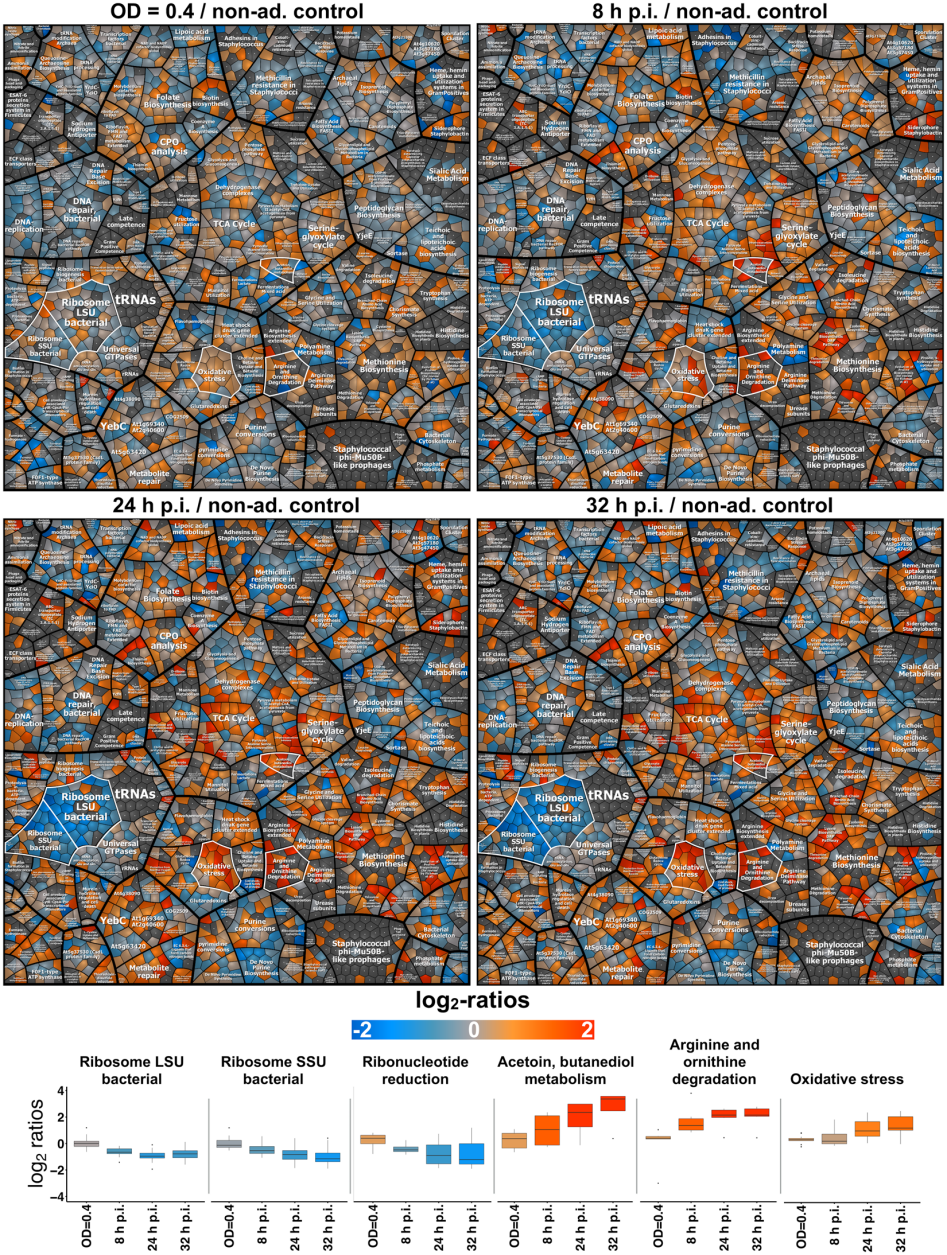
Protein level changes over the time course of the S9 human bronchial epithelial cell infection experiment displayed in Voronoi-like treemaps. Analysis of changes of abundances of *S. aureus* proteins during the time course of infection. Voronoi-like treemaps are used to illustrate the complex patterns of change. The log_2_-ratios of the protein level between sampling time points (exponentially growing *S. aureus* in pMEM [OD = 0.4], 8 h p.i., 24 h p.i., and 32 h p.i.) and the non-adherent control are depicted. Orange indicates increased amount, and blue represents proteins found in diminished amounts compared to the control. The boxplots at the figure bottom depict the log_2_-ratios of selected functional category groups highlighted (white polygons) in the treemap.

**Figure 7:**
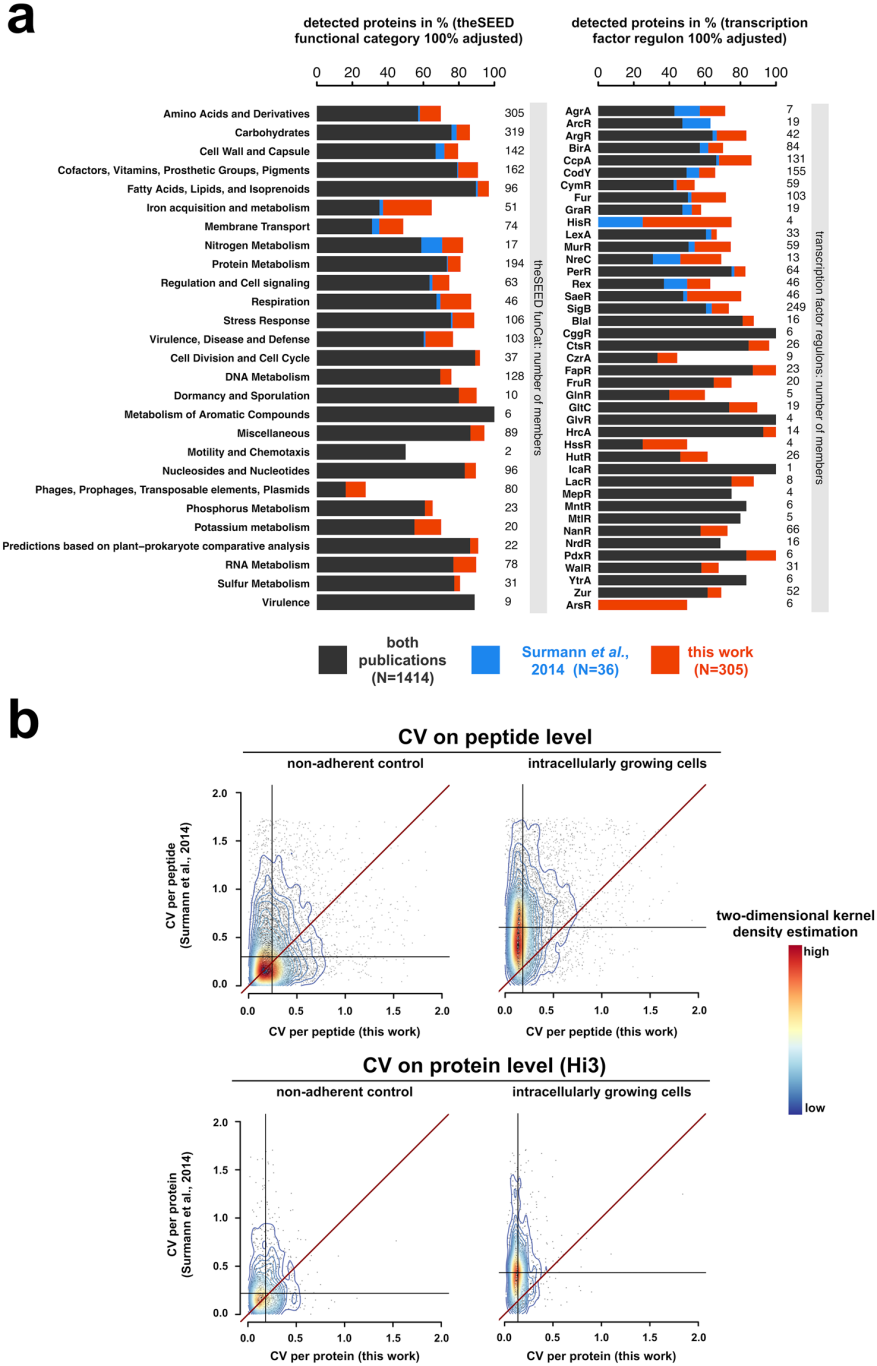
Comparison of identification rate and reproducibility of quantitative values between the work of Surmann and co-workers^60^ and this work. (**a**) The relative number of detected proteins of functional categories and transcription factor regulon members are depicted in the stacked barplots. (**b**) The coefficient of variation (CV) of peptide or protein intensity data is scattered. The peptide intensity was calculated as a sum over MS1 precursor ion area extracted with Skyline v2.5 by using Comet^8^ and ReSpect^61^ search results as an input (DDA; Surmann *et al*.^10^) or the sum over total peak area of MS2 elution groups (DIA; this work). The protein Hi3 intensity was calculated as a mean over the three most abundant peptides. The two-dimensional kernel density is plotted by colored line shapes. A more reddish color depicts a high point density and a more bluish color displays a low point density. The vertical or horizontal black lines indicate the median of the y-axis or x-axis data. The diagonal of equal CV values in both approaches is shown as a dark red line.

The DIA-MS analysis was carried out with 5 × 10^6^ *S. aureus* cells per sample and resulted in the identification of a total of 1,719 proteins over all samples. Of these, 1,309 proteins could be quantified against the non-adherent control sample (non-internalized bacteria after 1 h of infection), and 410 proteins displayed significantly altered abundance (absolute fold-change ≥1.5) in at least one sample time point compared to the control (Fig. 5, Supplemental Table S4). The magnitude of protein abundance changes increased from the earlier to the later sample points (Fig. 6). In direct comparison to the study of Surmann and co-workers^10^ we quantified 305 additional proteins and missed only 36. An increased coverage of approximately 15% in mean could be observed for the regulon-annotated proteins (Fig. 7a).

DDA data^10^ provided stochastic measurements in identification and quantitation whereas the DIA data collected (this work) proved superior in consistency of detection and quantification when comparing replicates of intracellularly growing *S. aureus* cells, which contain a higher proportion of human proteins sticking to the sample (Fig. 7b). This effect was not as intense in nearly pure bacterial samples (non-adherent control).

**Figure 8:**
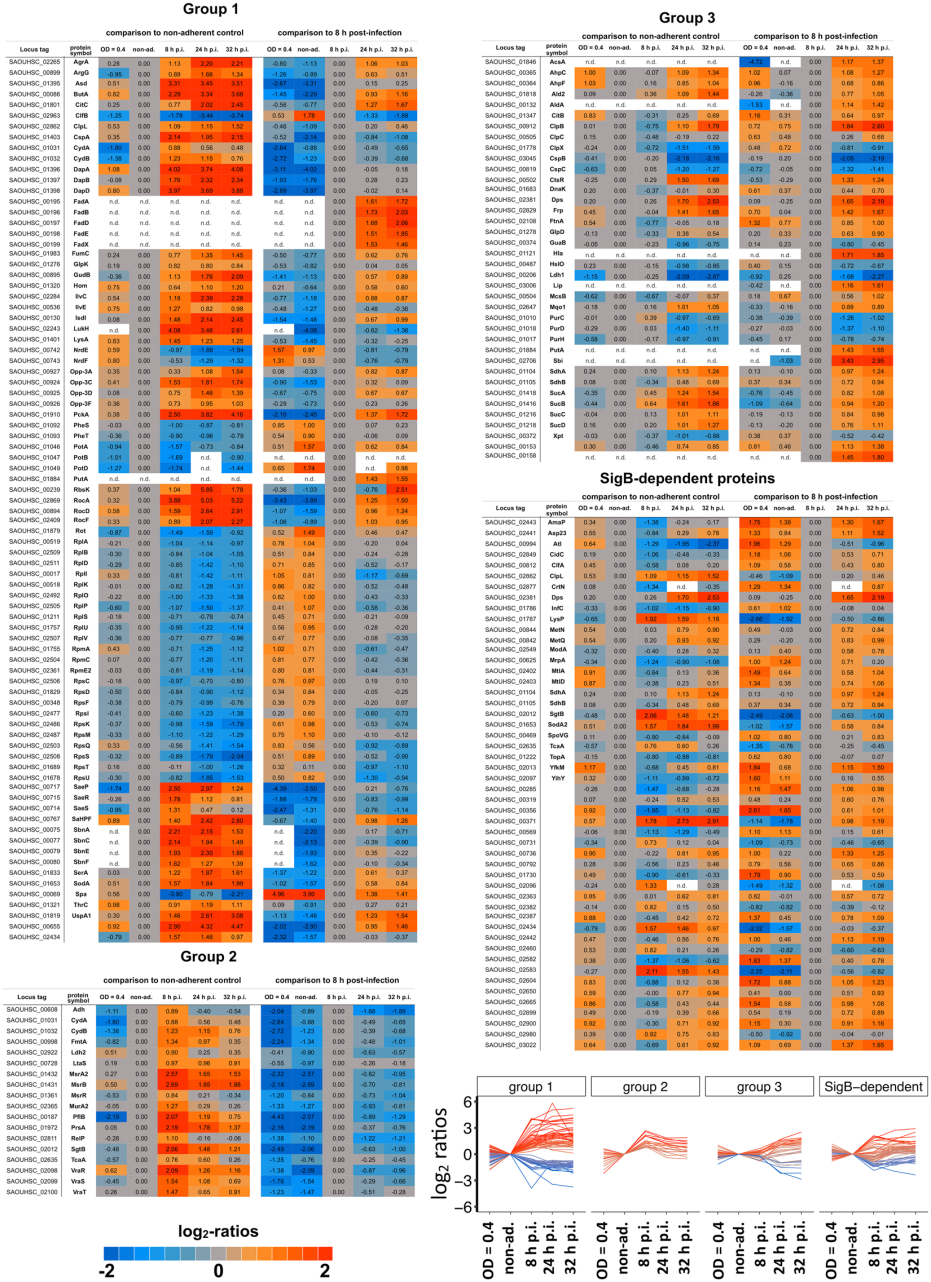
Three distinct groups of protein abundance changes during the course of infection. The ratios from the comparison to the non-adherent control sample and the comparison to the 8 h post-infection sample are colored-coded according to their log_2_-values. The line chart shows the time-dependent course of log_2_-ratio to non-adherent control. The lines are colored corresponding to the highest change in the post-infection phase (up = orange-colored/down = blue-colored).

With respect to the physiological adaptation of *S. aureus* after internalization, two different intracellular states could be distinguished: bacterial growth in the early phase (8 h p.i.) and survival of slow/non-growing bacteria in the late phase (24 h and 32 h p.i.), presumably reflecting the fact that two *S. aureus* subpopulations established inside host cells. As illustrated in Fig. 4c (bottom right graph) and the time-lapse movie (Supplemental Movie S1), the fraction of slow-/non-growing, intracellularly surviving bacteria (24 h and 32 h p.i.) constituted less than 10% of internalized staphylococci present at 8 h p.i. (measured as number of bacteria per S9 cell). Thus, changes in protein abundances observed at 8 h p.i. mainly reflect the adaptation of the intracellularly growing subpopulation, whereas the late intracellular samples (24 h and 32 h p.i.) represent reactions of slow-/non-growing, intracellularly surviving bacteria (Supplemental Fig. 2). Accordingly, proteins undergoing abundance changes during the course of infection were classified into three groups (Fig. 8): 1. proteins present in different amounts compared to the non-adherent control at all intracellular time points, i.e., proteins affected in both subpopulations; 2. proteins altered at 8 h p.i., i.e., in the intracellularly growing subpopulation, and returned to control level in the late phase (24 h and 32 h p.i.); 3. proteins exhibiting different abundance at 24 h and/or 32 h compared the 8 h sample, i.e., between intracellularly growing and slow-/non-growing staphylococci.

Group 1 included a number of proteins under the control of two global regulators modulating gene expression in response to the nutritional status of the cell, i.e., the carbon catabolite repressor CcpA and the GTP and amino acid sensing repressor CodY. Increased abundance of CcpA-regulated proteins reflects the switch from utilization of glucose to utilization of nutrients available within host cells^25^. Specifically, enzymes like GlpK (glycerol kinase), a putative dihydroxyacetone kinase (SAOUHSC_00655), PckA (phosphoenolpyruvate carboxykinase), RocF (arginase), RocD (ornithine transaminase), RocA (pyrroline-5-carboxylate dehydrogenase), GudB (glutamate dehydrogenase), and CitC (isocitrate dehydrogenase) showed increased abundance after internalization (8 h p.i.). The proteins encoded by the CcpA-repressed fatty acid degradation operon (FadA, FadB, FadD, FadE and FadX) were only found in intracellular samples and not detectable in the non-adherent control. Interestingly, levels of all these CcpA-regulated proteins appeared further enhanced in the slow-/non-growing population (24 h and 32 h p.i.), where additional CcpA-regulated proteins showed increased amounts (including, for example, GlpD, AldA, Ald2, Lip, AcsA, MurP, RbsK, PutA, and IpdC). The increased levels of GlpK and GlpD might reflect the utilization of glycerol, which is known as one of the preferred carbon sources during intracellular growth of other pathogens such as *Listeria monocytogenes* ^26^. For *S. aureus*, previous studies reported upregulation of the *uhpT* gene encoding a hexose phosphate transporter upon internalization^23, 27, 28^. Enzymes of the TCA cycle increased in level in *S. aureus* following internalization as already observed by Surmann *et al*.^10^. However, significantly higher levels at 8 h p.i. were only observed for CitC and FumC, whereas most of the TCA enzymes increased in level only at 24 h and 32 h p.i. (see below).

Derepression of the CodY regulon was reflected by higher amounts of amino acid biosynthetic enzymes (DapA, DapB, DapD, Asd, LysA, IlvE, IlvC, Hom, ThrC, SerA, and ArgG), subunits of the oligopeptide transporter Opp (OppA, OppC, OppD, OppF), and the acetoin reductase ButA at all time points in the intracellular niche. Increased levels of enzymes involved in arginine and lysine biosynthesis were already observed in three different host cell lines including S9^10^. Most of the CodY-dependent proteins showed a further slight increase at the later time points which was highest (2-fold) in the case of ButA. Remarkably, we observed increasing levels of the ribosome hibernation factor SaHPF (SAOUHSC_00767) recently identified as potential CodY target^28^ during the whole course of infection. It was shown that highest levels of 100 S ribosomes and HPF occur in *S. aureus* at the transition from exponential to stationary phase^29^ and that preservation of ribosome integrity by HPF is critical for long-term viability of *S. aureus* ^30^. Inside host cells, *S. aureus* is suggested to experience microaerophilic conditions as concluded from elevated levels of fermentation enzymes and the CydAB terminal oxidase in internalized bacteria^10^. Interestingly, except for ButA, fermentation enzymes were upregulated only in intracellularly growing bacteria (8 h p.i.) (Group 2 proteins, see below).

In agreement with previous transcriptome studies^23, 28^, we observed increased amounts of proteins involved in iron acquisition. The Fur-dependent Sbn proteins, which are responsible for the production of the siderophore staphyloferrin B^31^, the putative siderophore biosynthesis protein SAOUHSC_02434 and the heme-degrading monooxygenase IsdI were increased at all time points post-infection.

The reduced growth rate of *S. aureus* after internalization by host cells was accompanied by an approximately 2-fold decrease in ribosomal protein abundance (24 of the 56 ribosomal proteins displaying significantly altered levels) at 8 h p.i. compared to the non-adherent control, which was only slightly higher at 24 h and 32 h p.i. (Table S4). This rather small difference between 8 h p.i. and 24/32 h p.i. was unexpected when comparing intracellularly growing and slow-/non-growing bacteria.

The group of proteins found in decreased amounts in internalized staphylococci also included the ribonucleotide reductase (NrdEF), proteins involved in polyamine metabolism (PotA, PotB, PotD) and the covalently surface-anchored adhesin clumping factor B (ClfB).

Whereas most of the stress response-related proteins affected during internalization were specific for one of the two subpopulations, the universal stress protein UspA_1, the chaperone ClpL, the superoxide dismutase SodA, and the major cold-shock protein CspA were detected in higher amounts at all time points, further increasing over the infection period.

Higher expression of many virulence factor genes, in particular those regulated by the SaeRS two-component system, after internalization by S9 bronchial epithelial cells as well as human THP-1 macrophages has been recently reported at the transcriptional level^28^. Indeed, the SaeS, SaeR, and SaeP proteins encoded by the auto-regulated *sae* locus showed a significant increase in abundance compared to the non-adherent control, which was highest at the early time point. Of the SaeR targets, LukH followed the same pattern, whereas Hla and Sbi were only upregulated at 24 h and 32 h p.i. The expression of *hla* and *sbi* underlies complex regulation by the SarA transcription factor and/or regulatory RNAs^32, 33^. An increasing abundance of the virulence regulator AgrA was observed during the whole course of infection, indicating a higher upregulation in the non-growing population. In a long-term infection model with HUVEC cells, *agrA* expression in internalized *S. aureus* was highest in the acute phase (two days p.i.) and decreased five days later^34^. AgrA is the transcriptional activator of the regulatory RNAIII that inhibits translation of target genes including the virulence regulator Rot and staphylococcal protein A (Spa), which were found in decreased amounts at all time points p.i.

Group 2 comprises proteins that were specifically altered at the early time point (8 h p.i.) compared to the non-adherent control sample, thus linked to the physiological adaptation of the intracellularly growing subpopulation. Strikingly, many proteins involved in cell wall stress response including 10 VraSR-dependent proteins (e.g., PrsA, TcaA, FmtA, MurA2, RelP), the methionine sulfoxide reductases MsrA2 and MsrB, also inducible by cell wall targeting antibiotics^35^, and the putative regulator MsrR were increased in abundance at 8 h p.i. Their levels were largely reverted towards control levels in the 24 h and 32 h p.i. samples, so that most of them were significantly less abundant in the late samples compared to the early sample (Table S5). Methionine sulfoxide reductases are enzymes coping with oxidized methionine residues, and it was recently shown that a mutant lacking MsrA2 is sensitive to oxidative stress and exhibits reduced survival in mice^36^. Increased levels of the regulator VraR and the foldase PrsA, which were the most upregulated VraSR-dependent proteins in this study, were already reported for *S. aureus* internalized by S9 cells^21^.

The level of the alternative terminal oxidase CydAB was 2-fold higher at 8 h p.i. compared to the control and decreased slightly at the late time points. Several fermentation enzymes were upregulated only in early-stage intracellular bacteria including pyruvate formate lyase PflB, alcohol dehydrogenase Adh, and lactate dehydrogenase Ldh2. In contrast, Ldh1, which is highly induced under anaerobic conditions in the presence of glucose and the most abundant fermentation enzyme^37^, was unchanged after internalization and even decreased in abundance at 24 h and 32 h p.i. This observation is in good agreement with the fact that anaerobic induction of *ldh1* through inactivation of the redox-sensitive Rex repressor requires, in addition to a low NAD+/NADH ratio, the presence of glucose^38^.

Proteins belonging to Group 3 were specifically altered in the slow-/non-growing population, exhibiting different abundance in the late samples (24 h and/or 32 h p.i.) compared to 8 h p.i. (Table S5). Almost all proteins of the tricarboxylic acid cycle (CitB, SucA, SucB, SucC, SucD, SdhA, SdhB, Mqo1) showed increased amounts in the late samples, where also CitC and FumC were significantly increased compared to already elevated levels at 8 h p.i. In addition, proteins involved in coping with oxidative stress were induced, e.g., Dps (DNA protection protein), AhpCF (alkyl hydroperoxide reductase), and KatA (catalase). The same pattern was observed for another PerR-controlled protein, the ferritin-like protein FtnA, and for Frp, an NAD(P)H-flavin oxidoreductase, the *B. subtilis* ortholog of which confers protection against hypochloric acid^39^. Stress proteins under the control of the heat shock regulator CtsR were also significantly more abundant in the late samples than in the 8 h sample: ClpC (Clp protease ATP-binding subunit), the chaperones ClpB and DnaK, McsB (arginine kinase involved in CtsR degradation), and CtsR. Interestingly, it was recently shown that *Mycobacterium tuberculosis* ClpB protects the bacterium against irreversibly oxidized and aggregating proteins by sequestering them asymmetrically inside the cell and is crucial for surviving inside the host^40^. On the other hand, we observed decreased levels of HslO (Hsp33-like chaperone), ClpX (Clp protease ATP-binding subunit), and the cold-shock family proteins CspB and CspC. Enzymes involved in nucleic acid biosynthesis (e.g., PurH, PurC, PurD, GuaB, Xpt) were also significantly less abundant.

The alternative sigma factor SigB controls a large regulon including genes involved, amongst others, in stress response, cell envelope homeostasis, metabolism, and virulence. SigB is important for intracellular growth^22^ and plays a critical role in promoting bacterial intracellular persistence^34, 41^. In the present study, the amounts of only 13 SigB-dependent proteins, including ClpL, SgtB and LysP were increased and 14 were decreased in level (e.g., AmaP, CsbD, SpoVG) at 8 h p.i. compared to the non-adherent control. Strikingly, in the slow-/non-growing population (24 h and 32 h p.i.), 31 proteins that are at least under partial SigB-control, such as SpoVG and Asp23, exhibited 2-3fold higher levels than at 8 h p.i., including several hypothetical proteins. Of note, as SigB-dependent gene expression in *S. aureus* is particularly associated with the stationary phase^28, 42^, this observation is indicative of a stationary-phase status of the intracellularly surviving population analyzed at 24 h and 32 h p.i.

In summary, the data obtained from the analysis of internalized *S. aureus* in the S9 cell line infection model revealed detailed information about the stress and nutrient conditions of *S. aureus* during adaptation to the host cell milieu as well as long-term intracellular survival. Our data of early intracellular phase (8 h p.i.) and non-adherent control are in accordance with previous findings obtained for intracellularly growing *S. aureus* cells^10, 23, 24^, while also adding a lot of new information. As major adaptations to the intracellular niche, we detected increased amounts of a number of proteins under the control of CcpA and CodY, reflecting a nutrient limiting environment, and the induction of various stress responses (e.g., cell wall stress, oxidative stress) showing striking differences between intracellularly growing and slow-/non-growing staphylococci. In this study higher levels of SigB-dependent stress proteins were also recorded at the later time points, extending the previous observation of Pförtner *et al*.^22^ of an activation of SigB immediately after internalization and providing a link to the requirement for SigB for long-term intracellular survival and promotion of persister formation^41^.

### *In vivo* proteomics in a murine pneumonia model

A broad range of *S. aureus* proteome analyses have been conducted to describe and characterize the dynamic behavior of the human pathogen under laboratory conditions, mainly performed in shaking flasks^9, 43–46^. To mimic the natural behavior of the facultative pathogen in the context of human host-cell interaction, cell culture infection experiments were carried out^21, 24^. To date, these studies represent the closest interrogation of *S. aureus* proteome dynamics upon interaction with human cells, but still under laboratory-controlled conditions in a cell culture dish. The current and future challenge is true *in vivo* proteomics, which means the infection of a living organism with *S. aureus* and investigation of the dynamics of the pathogen’s proteome within the organism. Only few proteomics data have been published from true *in vivo* settings. In addition to recent research which identified the proteome of *S. aureus* during a murine bloodstream infection after six days of infection^47^, we aimed to identify *S. aureus* proteins in an *in vivo* pneumonia setting after 8 h, 24 h, and 32 h of infection to complement the data of the S9 human bronchial epithelial cell infection model.

The major challenge in conducting *in vivo* experiments with *S. aureus* is the low number of bacteria extractable post-infection and, therefore, the limiting amount of proteins. Diep and colleagues^47^ recovered 5 × 10^7^ cfu from mouse kidneys after blood stream infection, which was reflected in the 342 proteins identified in their surface enrichment proteome analysis. In our pneumonia setting we identified 578 proteins (570 proteins with two peptides in at least one sample time point) from approximately 2.15 × 10^5^ *S. aureus* cells (Supplemental Table S6) using DIA-MS. The quantified proteins comprised proteins with a wide spectrum of cellular functions. Among them were proteins required for oxidative stress response such as KatA (catalase), amino acid biosynthesis (e.g., DapD, Asd, RocD, ProC) and fermentation, e.g., PflB (pyruvate formate lyase) and ButA (acetoin diacetyl reductase), which were found with increased abundance, whereas ribosomal proteins and the nucleotide reductase NrdEF were found with decreased abundance in comparison to pre-infection conditions.

Furthermore, the ribosomal activity modulation protein SaHPF, glycolytic enzymes GapA and Pgi, the chaperone ClpB and the general stress proteins UspA_1 and UspA_2 showed increased abundance during the *in vivo* infection period, reflecting a glucose containing and stressful environment for *S. aureus* compared to the controlled, high nutrient-supplying shaking flask conditions from the pre-infection sample.

## Conclusion

In summary, we revealed new findings using DIA-MS and confirmed several previous results generated with the S9 cell infection model greatly expanding the knowledge of staphylococcal adaptation in the same experimental setup. Extending the experimental time window to 24 h and 32 h p.i. allowed identification of slow-/non-growing, intracellularly surviving bacteria with increased stress adaptation and a proteome profile indicating an aggravated nutrient limitation. This finding supports the fact that the pathogen adapts to a changing environment in the host with a variety of stresses including oxidative, acid, or cell wall stress and starvation for, e.g., glucose or iron. Enzymes involved in the detoxification of reactive oxygen species such as the catalase KatA and the Mn-dependent superoxide dismutase SodA were detected at elevated levels in internalized *S. aureus* cells.

The murine pneumonia model analysis from a very limited number of cells showed overlapping results with those known from the S9 cell infection model, as well as previously unknown, pneumonia model-specific adaptation processes. This demonstrates the power and reliability of the DIA-method for proteome analysis under infection conditions.

## Methods

### Preparation of samples for DDA-MS analysis, used for the generation of an *S. aureus* PeptideAtlas and a DIA fragment ion library

Samples for the generation of an *S. aureus* PeptideAtlas or a DIA library were obtained from *S. aureus* HG001 and from an isogenic *sigB* mutant derivative of this strain. The different experimental conditions for sample preparation included: different media (TSB, pMEM), samples from different growth phases (exponential growth, stationary phase of growth), iron limitation (2,2′-bipyridyl treatment), and iron excess. Furthermore, protein extracts from different cellular compartments (intracellular, extracellular, and membrane proteins) were analyzed. In general, protein extracts were prepared from the different samples, protein concentrations were determined, a tryptic digestion was performed, and tryptic peptides were purified using C18 RP-HPLC material. Peptides were analyzed by LC-MS/MS using different combinations of LC-instruments and MS devices. A description of the sample preparation methods is supplied in Supplemental Material 3.1.

### Acquisition of standard proteins

To increase the information included in the fragment ion spectral library, a set of proteins including virulence factors were recombinantly expressed in *E. coli* (Protagen AG, Dortmund, Germany), and proteomics data were acquired by nanoLC-MS/MS. The major goal of including recombinant proteins was to produce higher quality spectra from additional peptides per specific interest protein (e.g., virulence factors). In total, 73 recombinant proteins of *S. aureus* HG001 and other strains were included (Supplemental Table S7). Two protein mixes each containing half of the recombinant protein set were prepared to prevent the samples from becoming too complex; 20 mM ammonium bicarbonate was added to obtain a final protein concentration of 1 pmol/µl. The total protein amount of each protein mix was calculated, and trypsin (Promega, Madison, WI, USA) was added to obtain a substrate:enzyme ratio of 25:1. After 16 h incubation at 37 °C, proteolytic digestion was stopped by adding glacial acetic acid to a final concentration of 1%. Peptides were purified using ZipTip columns (Millipore, Schwalbach, Germany), dried using a vacuum centrifuge, and reconstituted in 10 µl buffer A [2% (v/v) ACN and 0.1% (v/v) acetic acid in HPLC-grade water (Baker)] before DDA acquisition on a Q Exactive^TM^ instrument.

### Preparation of bacterial *in vitro* samples for DIA-MS analysis

Growth conditions and sampling for the experiments are summarized in Table S1B. In brief, for the exponential growth and stationary phase comparison of *S. aureus* HG001, cells were grown in TSB and harvested via centrifugation (8,000 x g, 4 °C, 10 min) at OD_540nm_ = 0.5 and 4 h after entry into stationary phase. Three independent biological sampling series were carried out. Cells were disrupted using a FastPrep^**®**^ and subsequently pelleted by centrifugation for obtaining protein extracts. For mass spectrometric analysis 1 µg of protein extract was digested with trypsin (Promega, Madison, WI, USA) in a ratio of 1:25 over night at 37 °C. Thereafter, the digestion was stopped by adding 1% v/v purified trifluoroacetic acid (TFA) (Sigma). Peptide samples were purified using ZipTip_µ-C18_ columns (Millipore) and measured using a Q Exactive^TM^ mass spectrometer (Thermo-Fisher Scientific, Waltham, MA, USA). Just before measurement HRM-spike-in-mix (Biognosys AG) was added to the sample. For the on-column concentration test, a mixture of samples from cells of the exponential and stationary phase was digested as described above, followed by a dilution series and spiking in of the HRM-spike-in-mix (Biognosys AG). This procedure resulted in on-column concentrations of 10 ng, 50 ng, 100 ng, 500 ng, 1,000 ng, 2,000 ng, and 4,000 ng. Two technical replicates were measured.

### Preparation of samples from infection models subjected to DIA-MS analysis

The cell infection experiment was carried out with the S9 human cell line as described previously^22^. Samples were taken at 8 h, 24 h, and 32 h after infection. Non-adherent cells, obtained from the supernatant of S9 cells 1 h post-infection, and exponentially growing cells (pMEM OD_600nm_ = 0.4) were used as controls. The proteome measurements were carried out with 5 × 10^6^ sorted *S. aureus* cells each by on-filter digestion^24^.

The animal study was approved by the local government of Mecklenburg-Western Pomerania, Germany (LALLF M-V permit no. 7221.3‐1.1‐006/09 and 7221.3‐1.1‐019/11) and performed in strict accordance with guidelines for animal care and experimentation.

Prior to infection, *S. aureus* HG001 pJLGFP_opt_ (strain construction is described in the Supplemental Materials and Methods) was grown in pMEM until an OD_600nm_ of 0.4 was reached. The bacterial cell concentration was determined using a Guava easyCyte™ Flow Cytometer (Millipore). Eight weeks old BALB/c mice were intra-nasally infected with 6 × 10^8^ *S. aureus* cells. Lung lavage samples were taken 8 h, 24 h, and 32 h post-infection. Finally, the lavage samples were sorted in a FACSAria flow cytometer, pooled, tryptically digested^24^, and analyzed using a Q Exactive^TM^ mass spectrometer (Thermo-Fisher Scientific).

### Live-cell imaging during the cell culture infection experiment

Internalization experiments for long-term live-cell imaging were performed as described above but in special cell culture dishes (MatTek Corporation, 35 mm Glass Bottom Culture Dishes No. 0; Part No. P35G-0-20-C) with an infection volume of 4 ml. S9 cells were infected with *S. aureus* HG001 pJLGFP_opt_ at an MOI of 25. After 1 h of incubation, the supernatant was replaced by fresh eMEM containing 10 µg/µl lysostaphin. The cell culture dishes containing the infected cells were placed onto a Deltavision RT Image Restoration Workstation with an Olympus IX71 Nomarski Differential Interference Contrast microscope with a 40x objective using immersion oil. The microscopy chamber was pre-warmed to 37 °C and provided with an atmosphere of 5% CO_2_. After 15 min incubation time with lysostaphin (75 min p.i.), microscopy pictures were acquired every 5 min until about 34 h p.i. GFP-expressing bacteria were visualized using a filter with excitation 490/20 nm and emission 528/38 nm. The non-fluorescent S9 cells were observed in parallel by light microscopy. The pictures obtained were processed using Fiji (http://fiji.sc/Fiji). Deltavision files were first adjusted for brightness and contrast and then prepared using the “image5D z/TP Montage function”. Afterwards, a non-destructive scale bar was added to all slices. The time-stamper plug-in allowed labeling of observation time for all slices. Finally, the movies were exported as avi-files. The measurement of the mean GFP signal over the image was performed with Fiji via single picture analysis.

### Mass spectrometric data acquisition using different LC instruments, MS devices, and acquisition methods

*S. aureus* tryptic digests were analyzed on a TripleTOF^®^ 5600^+^ mass spectrometer (Sciex, Foster City, CA, USA) equipped with an Eksigent ekspert™ nanoLC 425 and cHiPLC^®^ system in trap-elute configuration. The 10 most intense ions were acquired in DDA mode for later use in library generation. SWATH data were generated with a SWATH^TM^ acquisition method.

Tryptic peptides of another sample set were separated on a nanoAcquity UPLC reversed phase column (BEH130, C18, 100 µm × 100 mm, Waters Corporation, Milford, USA) operated on a nanoAcquity UPLC system (Waters), and MS/MS data were recorded in data-dependent mode for precursor ions with charge 2 or 3 using an LTQ-FT instrument (Thermo-Fisher Electron, Bremen, Germany). These data were integrated into the *S. aureus* PeptideAtlas, as well as the MS data of analyses performed in DDA mode with an on-line coupled Dionex nLC system (Thermo-Fisher Scientific Inc., Idstein, Germany) connected to a Q Exactive Orbitrap-MS (Thermo-Fisher Scientific Inc.).

Mass spectrometric data-independent acquisition (DIA) and data-dependent acquisition (DDA) analyses using a Q Exactive^TM^ instrument (Thermo-Fisher Scientific) were performed in combination with LC on an UltiMate 3000 RSLC (Dionex/Thermo-Fisher Scientific, Idstein, Germany).

For further details see Supplemental Material 3.2.

### Data processing for *S. aureus* PeptideAtlas construction, *S. aureus* and S9 cell ion library building, as well as DIA data analysis

The *S. aureus* PeptideAtlas construction was based upon a Comet database search using an *S. aureus* protein database comprising 2,891 proteins in a target-decoy approach. Static and variable modifications were considered according to the sample characteristics.

The search results were processed with the Trans-Proteomic Pipeline (TPP, version 4.6)^48^ including PeptideProphet, iProphet, and ProteinProphet^49–52^. Per experiment a PSM FDR threshold was applied to maintain a constant FDR. The build named *S. aureus* HG001 2015-12 is available at www.peptideatlas.org.

Ion libraries were similarly constructed and based on a Comet database search and spectral library. Retention time values were incorporated by using the data with iRT peptides spiked-in (Biognosys, Schlieren, Switzerland) and RT alignment. The resulting aligned single-search ion library was filtered for 6–10 transitions per assay to obtain a robust, non-redundant ion library for the DIA data analysis. The procedures were carried out independently for the *S. aureus* and S9 data, resulting in separate ion libraries, which were deployed for the analysis of the LC-MS/MS data recorded in DIA mode. This data analysis was performed using Spectronaut^TM^ (v7.0.8065.3.13226 academic+)^53^. This included the comparison of the application of TripleTOF^**®**^ **-** and Q Exactive^TM^-derived *S. aureus* MS-assays. Settings for Spectronaut^TM^ analysis data extraction are given in Supplemental Table S2.

After Spectronaut^TM^ data extraction only highly significant assays (Q-value < 0.001) were normalized with respect to the control. They were subsequently analyzed protein-wise with a Wilcoxon rank sum test against an absolute fold change of 1.5 using Benjamini and Hochberg’s multiple testing correction^54^. Proteins with a p-value below 0.05 were assumed to be significantly regulated. The protein annotation and symbols used came from Aureowiki (http://aureowiki.med.uni-greifswald.de/Main_Page).

A detailed description of PeptideAtlas construction, ion library building, and DIA data analysis is given in Supplemental Material 3.3.

### Generation of a proteome-based phylogenetic tree over 46 sequenced *S. aureus* strains and its comparison to the ion library

The proteome sequences of 46 *S. aureus* strains were obtained from the NCBI sequence repository. The proteome-based unrooted and unscaled phylogenetic tree was generated by using the micropan package (v 1.0)^55^ in R 3.2.3^56^. Therefore, the single strain protein FASTA files were used for a protein blast all vs. all approach resulting in a phylogenetic similarity matrix. For comparison of the strains’ proteomes to those in the ion library an *in silico* tryptic digestion (missed cleavages [MC] = 2) was performed, and the resulting theoretical peptides were compared to the ion library.

### Visualization of proteomics results in Voronoi-like treemaps

Voronoi-like treemaps^57^ were generated using the Paver 2.0 software (Decodon GmbH, Greifswald, Germany). The application uses functional annotations on several levels and displays them in one interactive Voronoi-like treemap image. The functional annotation data was extracted from the latest “theSeed.org” build^58, 59^ (extraction date: 9^th^ of December 2015) for *S. aureus* NCTC8325 and *S. aureus* COL. The template treemap calculation of each strain was performed using the free swarm algorithm. The length of each protein (amino acid count) was normalized to the amino acid count of the largest protein to calculate Voronoi cell-size data based on protein length. These normalized data were deployed for visualization of the PeptideAtlas peptide data (Supplemental Fig. 3).

## Electronic supplementary material

Supplementary Material

Live-cell imaging movie S1
