# Supplementary material for: A global Staphylococcus aureus proteome resource applied to the in vivo characterization of host-pathogen interactions
Source: Sci Rep. 2017 Sep 8;7:9718. doi: 10.1038/s41598-017-10059-w (PMC5591248; doi:10.1038/s41598-017-10059-w)
Supplement: Supplementary file 1 — Supplementary Material [file 41598_2017_10059_MOESM1_ESM.pdf]

A global *Staphylococcus aureus* proteome resource applicable for the *in vivo* characterization of host-pathogen interactions

Stephan Michalik[1*], Maren Depke[1*], Annette Murr[1], Manuela Gesell Salazar[1], Ulrike Kusebauch[4], Zhi Sun[4], Tanja C. Meyer[1], Kristin Surmann[1], Henrike Pförtner[1], Petra Hildebrandt[1], Stefan Weiss[1], Laura Marcela Palma Medina[1], Melanie Gutjahr[1], Elke Hammer[1], Dörte Becher[2], Thomas Pribyl[3], Sven Hammerschmidt[3], Eric W. Deutsch[4], Samuel L. Bader[4], Michael Hecker[1,2], Robert L. Moritz[4], Ulrike Mäder[1], Uwe Völker[1,5], and Frank Schmidt[1,5]

[1] Interfaculty Institute for Genetics and Functional Genomics, Department of Functional Genomics, University Medicine Greifswald, Greifswald, Germany
[2] Institute for Microbiology, Ernst-Moritz-Arndt-University, Greifswald, Germany
[3] Interfaculty Institute for Genetics and Functional Genomics, Department Genetics of Microorganisms, Ernst-Moritz-Arndt-University, Greifswald, Germany
[4] Institute for Systems Biology, Seattle, WA, USA
[5] ZIK-FunGene, Interfaculty Institute for Genetics and Functional Genomics, Department of Functional Genomics, University Medicine Greifswald, Greifswald, Germany

[*] contributed equally

Corresponding author's contact information:
Frank Schmidt
University Medicine Greifswald
Interfaculty Institute for Genetics and Functional Genomics
Department of Functional Genomics
Friedrich-Ludwig-Jahn-Straße 15a
17475 Greifswald
Germany
phone: +49 (0) 3834 420 5815
fax: +49 (0) 3834 420 5809

e-mail: frank.schmidt@uni-greifswald.de

1 Supplemental Figures 3

2 Supplemental Results & Discussion 5

2.1 The *Staphylococcus aureus* PeptideAtlas as a prerequisite for novel analyses of host-pathogen interactions 5

2.2 Library size for bacterial sample analysis 7

2.3 Retention time alignment of LC-MS runs and transfer of iRT to runs without iRT peptides 8

2.4 Cross-device usability of DIA-MS assays generated with SCIEX TripleTOF® 5600^{+} and Thermo Q Exactive^{TM} 11

2.5 Dynamic range of DIA measurements of *S. aureus* protein samples 15

2.6 Benchmarking DIA measurements of growing and non-growing cell samples of *S. aureus* 17

3 Supplemental Material and Methods 23

3.1 Bacterial growth, optimized GFP strain construction, media and protein preparation, tryptic digestion, and peptide purification for samples subjected to DDA MS analysis and used for the generation of an *S. aureus* PeptideAtlas or a DIA library 23

3.1.1 Intra- and extracellular proteins from *S. aureus* HG001 pMV158GFP after iron limitation by 2,2'-bipyridyl and under control conditions 23

3.1.2 Intra- and extracellular proteins from *S. aureus* HG001 growing in TSB at different growth phases 24

3.1.3 Membrane proteins from *S. aureus* HG001 growing in TSB at different growth phases 24

3.1.4 Intra- and extracellular proteins from *S. aureus* HG001 and its isogenic Δ*sigB* mutant 25

3.1.5 Generation of S9 and *S. aureus* extract mixtures 25

3.1.6 Construction of *S. aureus* HG001 carrying optimized GFP 26

3.2 Mass spectrometric data acquisition using different LC instruments, MS devices, and acquisition methods 26

3.2.1 Mass spectrometric acquisition of TripleTOF DDA and SWATH data 26

3.2.2 Mass spectrometric acquisition of nano-HPLC-ESI-LTQ-FT data 27

3.2.3 Mass spectrometric data-dependent-acquisition (DDA) analyses using a Q Exactive^{TM} instrument 27

3.2.4 Mass spectrometric data-independent-acquisition (DIA) analyses using a Q Exactive^{TM} instrument 28

3.3 Data processing for *S. aureus* PeptideAtlas construction, *S. aureus* and S9 cell ion library building, and DIA data analysis 28

3.3.1 Data processing for *S. aureus* PeptideAtlas construction 28

3.3.2 Building an *S. aureus* fragment ion spectral library for DIA data analysis 29

3.3.3 Building an S9 human cell line fragment ion spectral library for DIA data analysis 30

3.3.4 DIA data analysis 30

3.3.5 DDA data analysis of S9 and *S. aureus* extract mixtures and TSB exponential and stationary phase samples 31

3.3.6 DIA data analysis of S9 and *S. aureus* extract mixtures 31

3.3.7 R-packages used for the analysis and plotting 32

4 Supplemental legends 34

5 Supplemental references 35

6 Supplemental tables 39

1 Supplemental Figures

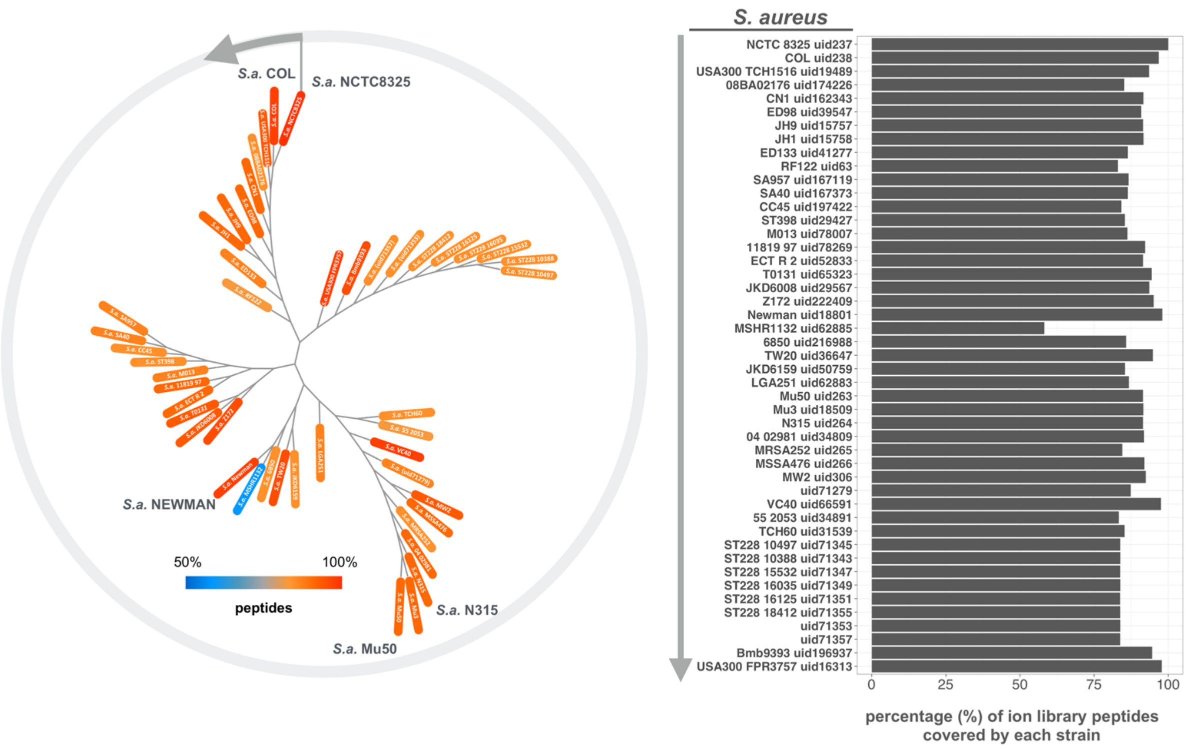

<u>Supplemental Figure 1: Coverage of the generated ion library over various *S. aureus* strains.</u> Comparison of *S. aureus* strains on the proteome level. The proteome-based, unrooted, and unscaled phylogenetic tree of 46 different *S. aureus* strains visualizes the relationship of the strains on the proteome level. The color displays the percentage of peptides covered from the ion library for each strain. More intense orange indicates a higher coverage of the ion library. The dark grey arrow indicates the reading direction of the quantitative data presented in the bar plot (left side of the figure) from the top to the bottom.

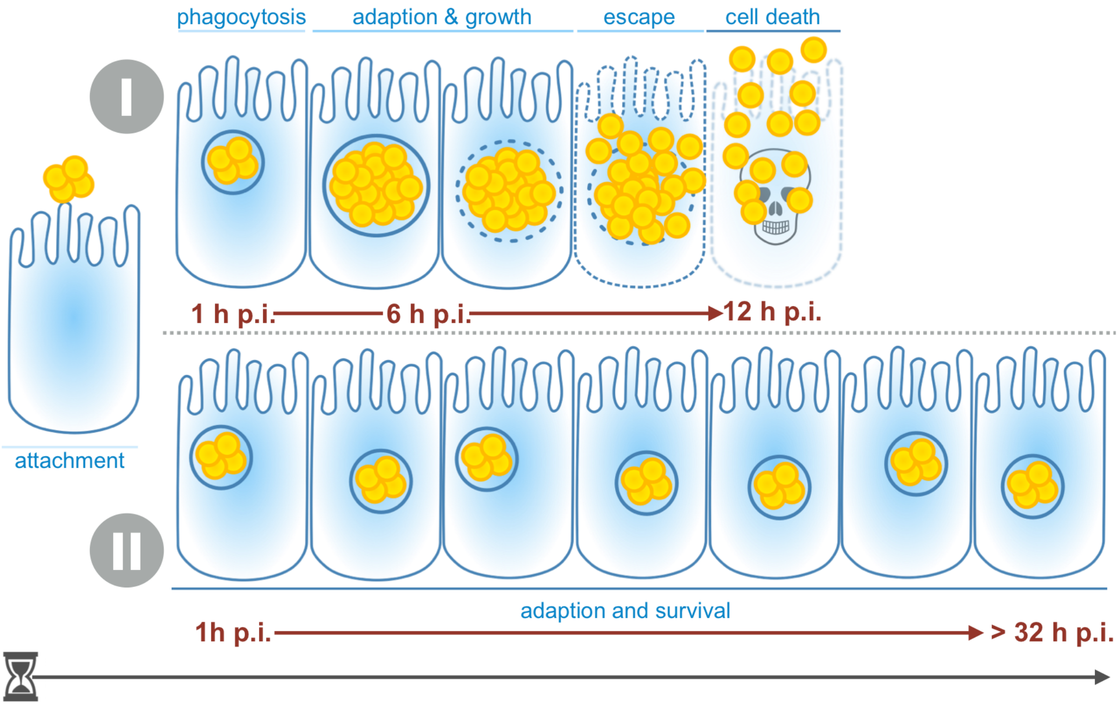

<u>Supplemental Figure 2:</u> Model of *S. aureus* infection and re-infection of host cells. The scheme displays the suggested *S. aureus* host cell infection and proposes two cell populations during infection deduced from the life cell imaging movie (Supplemental Movie S1). (I. Growing, host cell killing and escaping bacteria and possible re-infection of host cells; II. Bacteria survive in the intracellular milieu)

2 Supplemental Results & Discussion

2.1 The *Staphylococcus aureus* PeptideAtlas as a prerequisite for novel analyses of host-pathogen interactions

PeptideAtlas is a well-known, publicly available resource for proteome data[1]. Shotgun MS data from different experiments are processed with the Trans-Proteomic-Pipeline (TPP)[2] in a highly stringent analysis and results are presented to the community as PeptideAtlas builds to support experiment planning, data mining and the selection of proteotypic peptides for targeted MS approaches. Here, we generated the first *S. aureus* PeptideAtlas from previously published[3] and newly acquired *S. aureus* MS DDA data sets. To analyze the proteome of the human pathogen *S. aureus* HG001, a derivate of strain NCTC8325, we first fractionated extensively the lysate and then acquired DDA-MS data[3]. To increase the proteome coverage, we performed several additional measurements from membrane fractionated samples, intra- and extracellular proteome preparations from cells growing under iron limitation, and recombinant *S. aureus* proteins. Furthermore, data sets of extracellular proteins from *S. aureus* HG001 wild type and its isogenic Δ*sigB* mutant in different media and from a time kinetics study of GFP-expressing *S. aureus* HG001 pMV158[4] internalized by A549 cells were included to build a comprehensive *S. aureus* PeptideAtlas. After combining all data and applying strict thresholds to include only the most reliable peptide identifications and inferred proteins, we achieved the detection of 2097 proteins representing ~3/4 of the theoretical proteome. We combined all datasets (Supplementary Table S1A) and created a comprehensive *S. aureus* proteome repository which can be accessed by the research community to explore the observed peptides per protein, their spectra and related information at http://www.peptideatlas.org.

We analyzed not only *S. aureus* HG001, but also data from a different only *S. aureus* strain named COL. The proteome of *S. aureus* COL was extensively characterized in a previous study using subproteomic fractions of cytosolic proteins, membrane-bound proteins, cell surface-associated proteins, and extracellular proteins[5] (Supplementary Table S1A), resulting in the identification of 65% of the annotated proteome and we generated a separate PeptideAtlas build for this strain.

To assist in the physiological interpretation of proteomic data, we provide Voronoi-like treemaps[6] data display to provide a functionally sorted representation of the *S. aureus*

proteome in the PeptideAtlas (Supplemental Figure 3). The *S. aureus* HG001 PeptideAtlas is the most comprehensive resource where most proteins are represented by at least three peptides (median = 24; mean = 39) with 90 proteins covered by two peptides and only 126 by a single peptide. The *S. aureus* COL build likewise covers most of the proteins with at least three peptides (median = 12; mean = 17), 127 proteins with two, and 200 proteins with one peptide.

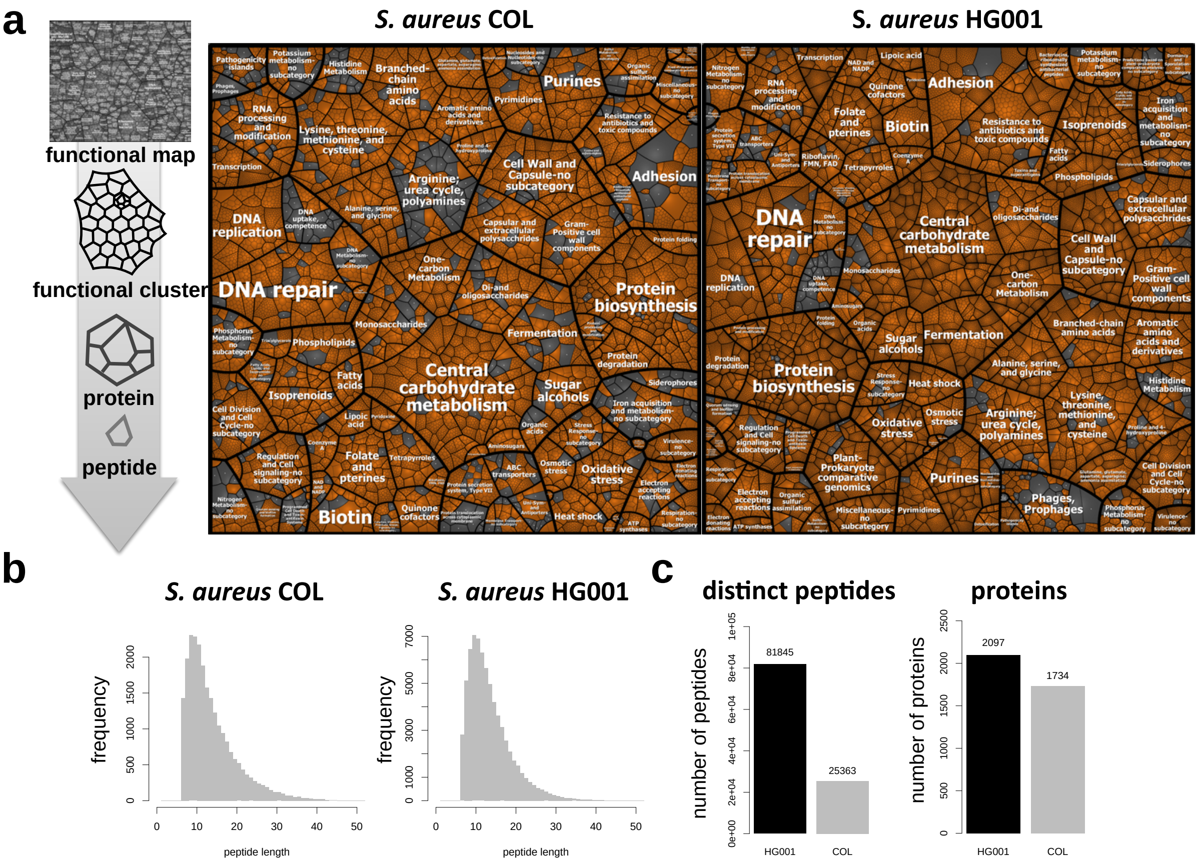

<u>Supplemental Figure 3:</u> Functional annotation coverage and comprehensiveness of the *S. aureus* PeptideAtlas.

(a) The Voronoi-like treemaps describe the number of identified peptides per annotated protein (orange) represented by the polygons, which also can be accessed via the *S. aureus* PeptideAtlas (www.peptidatlas.org).

(b) Displayed are the histograms of the identified peptide length count in the *S. aureus* HG001 and *S. aureus* COL PeptideAtlas build.

(c) The barplots visualize the distinct identified peptide and protein count in the *S. aureus* HG001 and *S. aureus* COL PeptideAtlas build.

2.2 Library size for bacterial sample analysis

In order to test if the addition of strongly different sample sets and proteome fractionation is beneficial for the library generation for a comprehensive coverage of the proteome in our bacterial setting a library comparison was carried out between a standard library (12 measurements of one sample pool [mixture of *S. aureus* grown in TSB; exp. and stat. phase]) and a comprehensive library containing strongly different sample sets and proteome fractionation (Supplemental Table S1A).

The analysis of *S. aureus* tryptic soy broth (TSB) exponential and stationary phase samples measured in DIA mode and analyzed with both libraries (standard & comprehensive) revealed an assay surplus of 24% when the comprehensive ion library including non-iRT data was used, which resulted in an increase in the number of proteins identified of 12% when compared to the analysis using the basic ion library generated from just 12 DDA data sets. As a result, the quantification was now based on more data-points, which also proved to be more robust. The

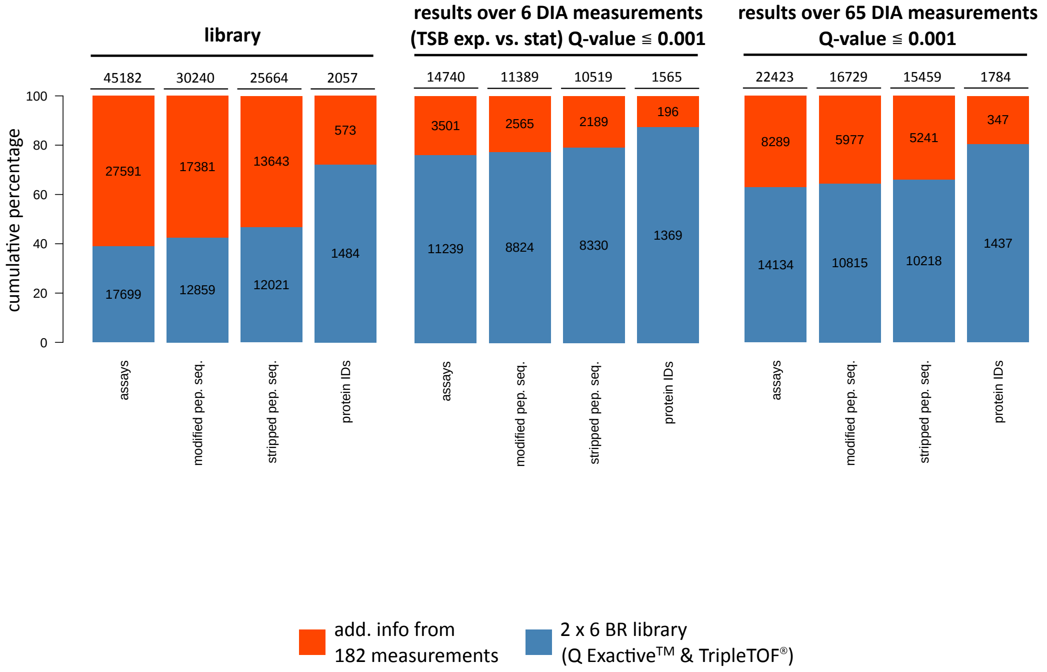

Supplemental Figure 8: Library size for data analysis.
The gain of information in the *S. aureus* ion library was shown when fractionated samples and diverse sample types (N=182) were added to a small sample set of 12 replicates measured with iRT spike-in peptides (reflecting a default ion library build). The cumulative percentage depicts the 100% scaling for each parameter. Absolute numbers are displayed in the bars.

analysis of 65 different DIA runs (Supplemental Table S1B) showed an assay surplus of approximately 37% resulting in the identification of approximately 19% more proteins (Supplemental Figure 8) when use of the expanded ion library is compared with that of the basic library. Therefore, we conclude that a larger ion library is beneficial for the investigation of organisms with a highly variable proteome available under different growth conditions.

2.3 Retention time alignment of LC-MS runs and transfer of iRT to runs without iRT peptides

A previously published dataset of our group[3] contains high resolution mass spectrometric data of highly fractionated *S. aureus* HG001 proteins (e. g. SCX or off-gel IEF fractionation). The dataset consists of measurements with different liquid chromatography (LC) gradients such as 30 min, 100 min and 120 min linear LC gradient. In order to benefit from the iRT prediction algorithm implemented in data-independent acquisition (DIA) data analysis tools like Spectronaut™ [7], all previously described high-accurate MS analysis runs[3] were – based on peptide identification – separately aligned to a reference set of 6 sample replicates with spiked-in iRT peptides[8] measured using a Q Exactive™ (Supplemental Figure 6). The retention time (RT) values for each of the peptides in the measurements with spiked-in iRT peptides were combined by using the average over the iRT resulting in a master list of peptides and corresponding iRT values. This list was compared with a peptide list of each sample measured without iRT peptide spike-in. The intersecting peptides of this comparison were incorporated in a linear regression (RT LC-MS without iRT peptides vs. RT LC-MS with iRT peptides). Afterwards, the resulting residuals of the linear regression were used to filter for those peptides exhibiting a residual in the 25 to 75% quantile range over all residuals. This filtering step was performed in order to remove outliers or LC gradient anomalies and resulted in a filtered peptide list ("landmark peptides"). The "landmark peptide" list was used to calculate a RT ratio (RT LC-MS without iRT peptides vs. RT LC-MS with iRT peptides), which was subsequently utilized for a non-parametric regression (locally weighted scatterplot smoothing - LOESS[9]), which is beneficial for the alignment of non-linear LC shifts[10]. The resulting curve-fitted values were used to transfer the iRT values from the LC-MS run with iRT peptides to the run without iRT peptides. New iRT values for peptides, which were not included in the

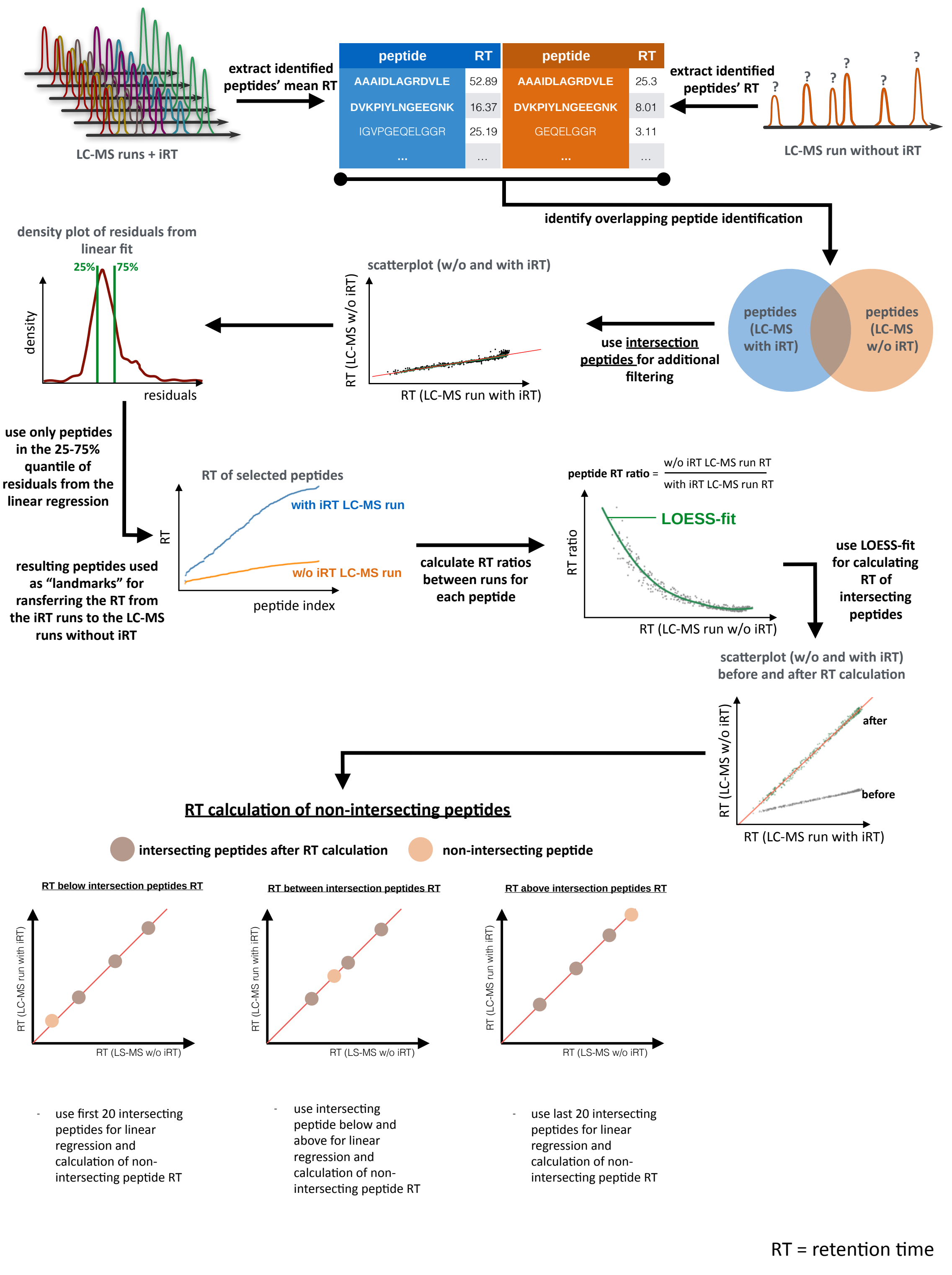

Supplemental Figure 6: Schematic representation of the iRT transferring to LC-MS runs without iRT peptides spiked-in.

"landmark peptide" list, were interpolated using a linear regression along the non-parametric regression. Briefly, for peptides with an RT value smaller than the RT values in the "landmark peptide" list, the first 20 intersecting peptides with the smallest RT values were used for a

linear regression and calculation of the non-intersecting peptide´s RT. For peptides with RT values in the range of the “landmark peptide” list peptides’ RT, the adjacent peptides with smaller and higher RT values from the “landmark peptide” list were used for a linear regression and calculation of a non-intersecting peptide’s RT. For peptides with RT values higher than the “landmark peptide” list peptides’ RT, the 20 intersecting peptides with the highest RT values were used for a linear regression and calculation of non-intersecting peptide’s RT.

To verify the quality of the alignment as well as the iRT transfer calculations an analysis over 65 different DIA-MS runs (Supplemental Table S1B) was performed. This analysis was based upon two different ion libraries: i) a library containing only peptides from iRT peptide-containing LC-MS runs and ii) a library containing only peptides for which the iRT value was generated by alignment and transfer of the iRT values (Supplemental Figure 7).

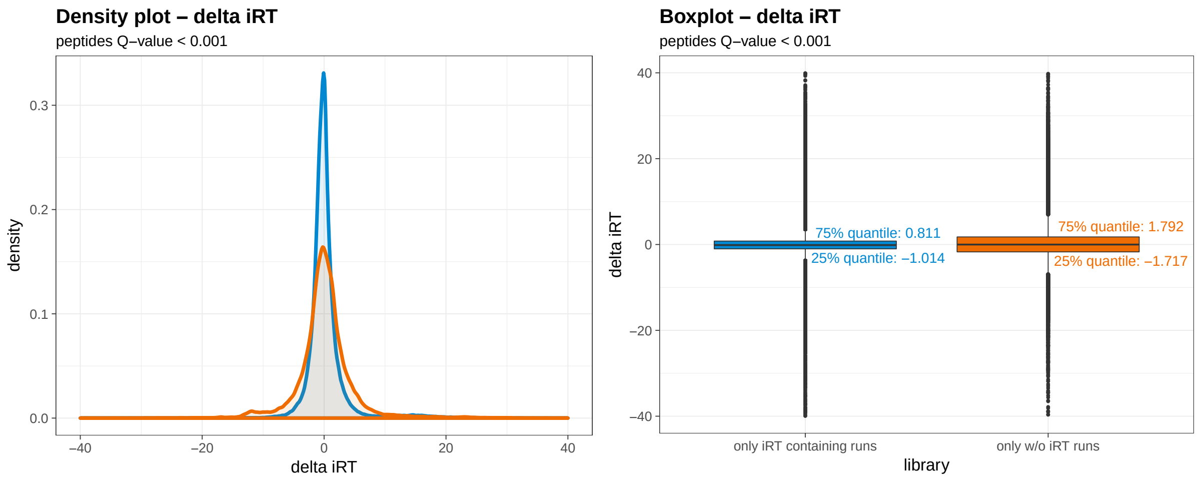

<u>Supplemental Figure 7:</u> Delta iRT of DIA analysis from 65 different DIA runs. The density plot and the corresponding boxplot are depicting the delta iRT from all peptides identified with a Q-value below 0.001. The blue colored curve/box represents the delta iRT of peptides identified with a library comprising only peptides from iRT LC-MS runs as basis. The orange colored curve/box depicts the delta iRT of peptides identified with a library including only peptides were the iRT was generated by alignment and transferring of the iRT. The 25% or 75% quantile of iRT were labeled in the boxplot.

The delta iRT values, which represent the difference between the anticipated iRT value to the empirical iRT value were reported. The distribution of delta iRT values within a 25% to 75% quantile from ca. −1 to 0.8 delta iRT for the identifications (Q-value < 0.001) using the library containing only peptides detected in LC-MS runs with iRT peptides revealed only a minor variation. The comparison to the analysis with the library containing only aligned peptides

revealed delta iRT values from ca. −1.7 to 1.8 within the 25% to 75% quantile. This slightly higher delta iRT value deviation is still in an acceptable range for the data analysis isolation window during a DIA-MS analysis.

2.4 Cross-device usability of DIA-MS assays generated with SCIEX TripleTOF® 5600^+ and Thermo Q Exactive™

The basic ion library was generated with SpecL[11] by analyzing six DDA runs derived from a Q Exactive™ and six DDA runs derived from a TripleTOF® 5600^+ instrument. Samples analyzed on both instruments were equalized with spiked-in indexed retention time (iRT) [8] peptides and converted to an empirical retention time, iRT, which represents a "fixed number relative to a standard set of reference iRT-peptides that can be transferred across laboratories and chromatographic systems"[8]. The major differences between the TripleTOF® 5600^+ and Q Exactive™ instruments are the mass detection and the fragmentation mode. TripleTOF® 5600^+ data are collected with CID fragmentation (collision-induced dissociation) and Q Exactive™ data with HCD (higher-energy C-trap dissociation) fragmentation.

In order to test i) if an ion library generated with a Q Exactive™ instrument is also suitable for DIA (data-independent acquisition) data analysis of data collected on a TripleTOF® 5600^+ instrument, ii) if we can merge the MS information obtained from both instruments in one fragment ion spectral library for data analysis purposes, and iii) if the generated assays in the fragment ion spectral library can be used to analyze data acquired on both MS devices, we measured the same sample with both instruments in DIA mode, analyzed the data with the same fragment ion spectral library and compared the results.

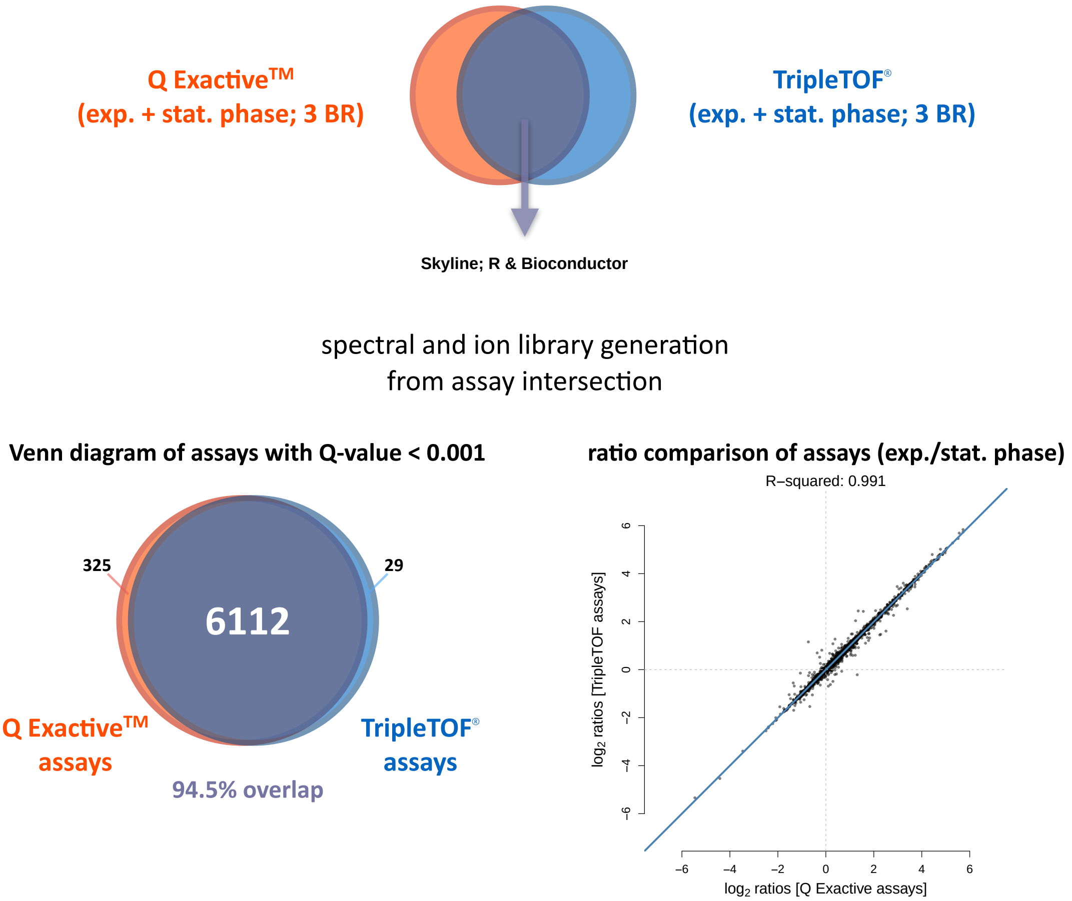

<u>Supplemental Figure 4:</u> Cross-device usability of DIA-MS assays. To determine whether assays derived from a Q Exactive^{TM} or a TripleTOF$^{®}$ instrument can be used for cross-device DIA-MS data analysis, an intersection-based assay ion library was generated. *S. aureus* HG001 TSB exponential and stationary phase samples (3 BR each) were analyzed with the redundant ion library, resulting in a large overlap (>94%) of extracted ion chromatograms (Q-value cut off < 0.001; Venn diagram) and peptide abundance patterns regardless of the origin of the assay (scatter plot).

The ion library used for this approach consisted of six DDA runs from TripleTOF® 5600^{+} and six Q Exactive^{TM} DDA measurements from the same sample. To elucidate the potential cross-platform usage of an assay, a combined intersection ion library – this means overlapping assays derived from the Q Exactive^{TM} and TripleTOF® 5600^{+} instruments – was used for the analysis of the DIA measurements with both devices (TripleTOF® 5600^{+} and Q Exactive^{TM}). If an assay was only found in either the Q Exactive^{TM} or the TripleTOF® 5600^{+} measurement the assay was neglected. This resulted in a fragment ion spectral library consisting of 13,392 assays (6,696 from Q Exactive^{TM} and 6,696 from TripleTOF® 5600^{+}) containing the exact same number of assays generated by both devices in order to eliminate a possible bias of the

comparative down-stream analysis due to a higher number of assays being derived from one device (Supplemental Figure 4).

Strikingly, the cross-assay usage evaluation was successful in most of the cases tested with Q Exactive™ DIA and TripleTOF® 5600⁺ measurements (Supplemental Figure 4). The majority of the assays (>94%) was identified even if an assay was generated on the other instrument (Supplemental Figure 4). Additionally, the assays were not only identified on one instrument while being generated by the other, but also yielded highly comparable quantitative ratio data on both devices (Supplemental Figure 4). An analysis of the assay total peak area (TPA) of growing and non-growing cells measured with the Q Exactive™ in DIA-mode and analyzed with assays derived from Q Exactive™ DIA and TripleTOF® 5600⁺ revealed a high correlation between both assay fractions (0.991 R-squared) (Supplemental Figure 4).

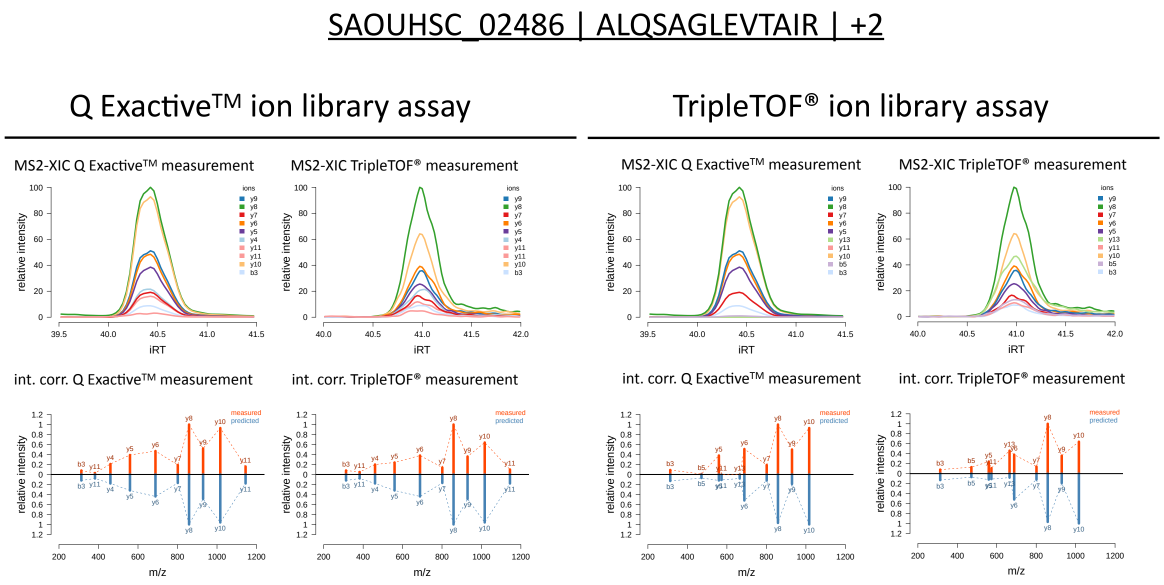

A representative example for assay cross-device usage is depicted in Supplemental Figure 5.

<u>Supplemental Figure 5:</u> Representative examples for the cross-platform assay usage. Displayed are the pseudo MS2 extracted ion chromatogram (MS2-XIC) and the MS2 relative intensity correlation of an assay (protein-ID: SAOUHSC_02486; peptide: ALQSAGLEVTAIR; charge: +2) generated from measurements obtained via the Q Exactive™ or TripleTOF® instrument.

The extracted ion chromatogram shape correlation was highly similar. The fragment ion masses and the correct relative intensity of fragment ions were mostly found with high confidence in both measurements and even with device-foreign assays. Recently, de Graaf and colleagues[12] described that the MS2 fragmentation pattern of ions derived from QqQ-CID

and HCD devices were similar, based on the calculation and comparison of cross-correlation scores. In agreement with de Graaf *et al.* we have concluded that cross-device assays can be used for the data analysis of DIA experiments in the future and might be highly beneficial for the proteomics community.

In summary, our data indicated that a DIA approach and analysis with the generated ion library can be performed on both MS instruments and that the obtained results are well-suited for correlation and lab comparison, underlining the benefit of an ion library for the community.

2.5 Dynamic range of DIA measurements of *S. aureus* protein samples

The dynamic range of the DIA method was tested by an on-column loading amount series with a digested complex cytosolic *S. aureus* proteome sample, which was measured on a Q Exactive^{TM} instrument and analyzed via Spectronaut^{TM} (Supplemental Figure 9).

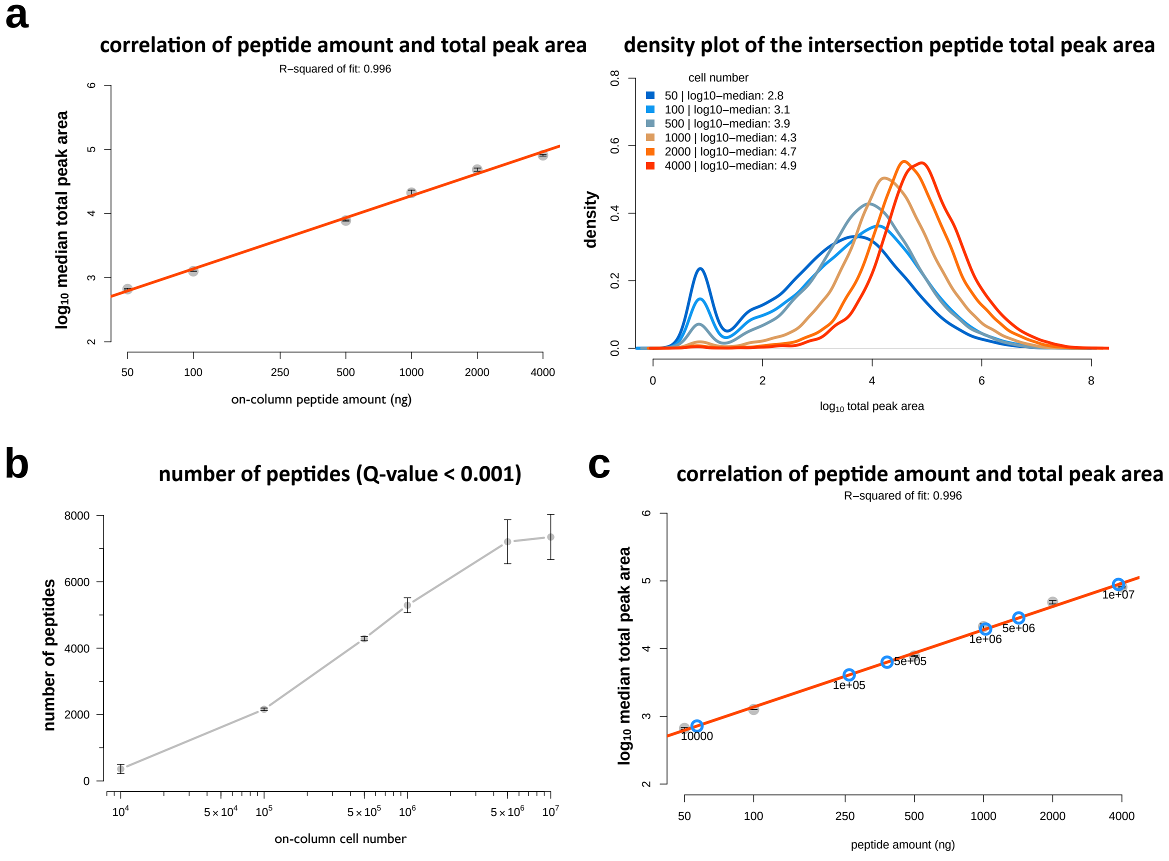

Supplemental Figure 9: Dynamic range of DIA-MS analyses.
(a) Relationship between on-column peptide load and total peak area. The median total peak area over the concentration series (50 to 4,000ng) is plotted. The linear regression indicates the linear range of the method. The kernel density plot over the concentration series depicts the distribution of the total peak area signals.
(b) Relationship between on-column cell number and the number of identified peptides. The number of peptides is depicted in relation to the number of cells digested and loaded on-column for analysis in the mass spectrometer.
(c) Relationship between on-column cell number total peak and peptide load and total peak area. Based on the linear fit (orange line) over the median total peak area of the individual on-column protein amount measurements (grey dots); the cell number measurements were displayed based on their median assay total peak area (open blue circles).

The median total peak areas were used for the estimation of the linear dynamic range. A linear range was obtained from 50 ng to 4,000 ng on-column loads on a 50 cm column. This observation implies that samples with a peptide amount far below 50 ng (e.g. 5 ng and 10 ng) are not feasible for the analysis due to the device-specific detection limits, as shown in earlier data-dependent experiments[13,14]. An on-column load of around 1,000 ng was a good point of reference for analysis, since higher on-column amounts resulted in the saturation of information gain. Furthermore, on-column loads of 1,000 ng and more displayed a lower number of identified signals due to background interference in comparison to on-column loads of less than 1,000 ng (Supplemental Figure 9, Supplemental Figure 10).

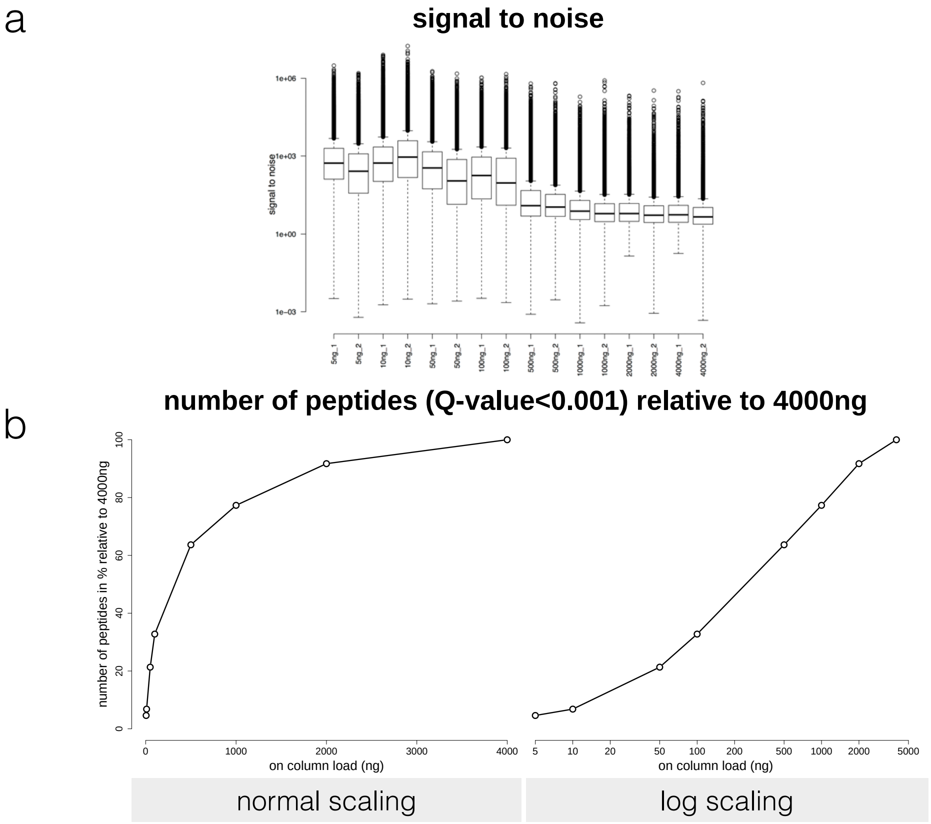

<u>Supplemental Figure 10:</u> Quality control of DIA-MS analyses.
(a) Signal-to-noise boxplot over measurements.
(b) Percentage of number of proteins or peptides identified in the whole column load series normalized to a 4,000 ng column load as maximum value.

Over the last 15 years, systems biology approaches have become the focus of research[15]. Systems biologists model complex biological processes *in silico* based on wet lab data. Here, a key element for the understanding of the functions of organisms is understanding the

complex interplay of the proteins inside a cell. Therefore, knowledge of the absolute number of proteins per cell is the essential basis for the calculations and for modeling of such interactions. For absolute protein quantification, the adjustment of recorded quantitative data to cell number data is an important criterion, rendering very crucial the determination of cell numbers for robust DIA analysis. A series of peptide extracts resulting from different *S. aureus* cell numbers was analyzed in a Q Exactive™ mass spectrometer (Supplemental Figure 9b, c). To date, very low cell numbers such as 1 x 10^2 to 1 x 10^4 have not been feasible for a global MS-based analysis, while meaningful proteome analyses on a Q Exactive™ mass spectrometer have been accomplished with cell numbers of approximately 1 x 10^5 or 1 x 10^6 (and higher).

2.6 Benchmarking DIA measurements of growing and non-growing cell samples of *S. aureus*

In order to extensively benchmark the method and the generated *S. aureus* DIA assay fragment ion library, we used the well-known model of growing and non-growing *S. aureus*

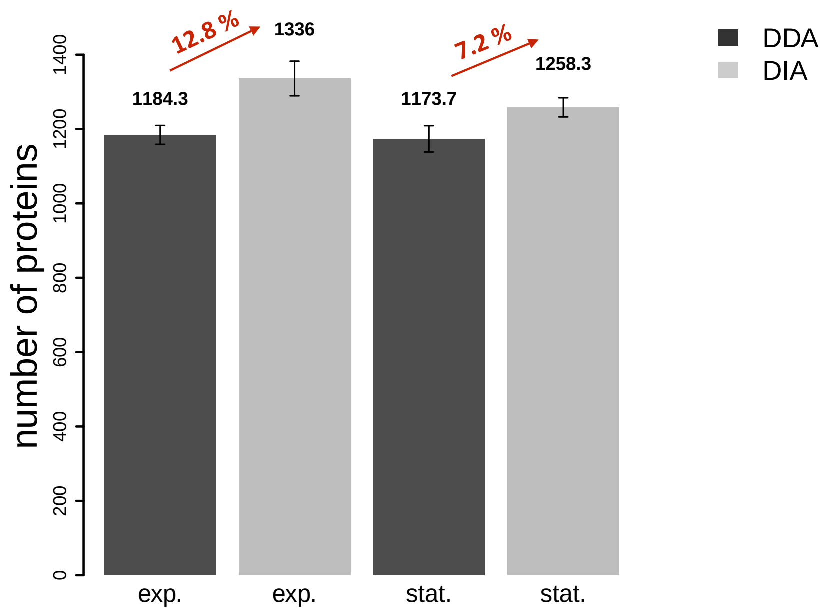

<u>Supplemental Figure 11:</u> Direct comparison of DIA-MS and DDA-MS measurement; Protein identification per sample.
Three biological replicates of *S. aureus* grown in TSB media at exponential (exp.) and at stationary (stat.) phase were used to calculate the number of protein identifications per sample in DDA-MS (black bars) as well as in DIA-MS (grey bars) analysis.

HG001 cells in the most frequently used complex medium for *S. aureus*, TSB (tryptic soy broth).

In direct comparison DIA-MS performs better in terms of protein identifications per sample and coefficient of variation of protein quantification compared to DDA-MS (Supplemental Figure 11, 12).

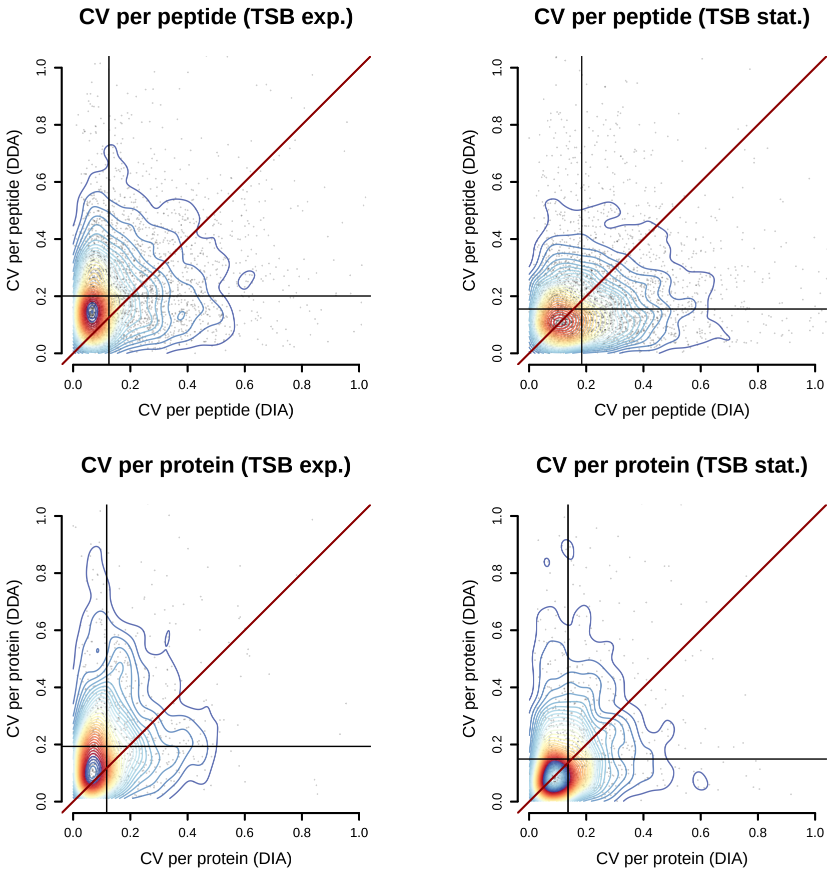

<u>Supplemental Figure 12:</u> Direct comparison of DIA-MS and DDA-MS measurement; Coefficient of variation over growth phases.

The coefficient of variation (CV) is scattered. The two-dimensional kernel density is plotted via colored line shapes. A more reddish color depicts a high point density and a more bluish color displays a low point density. The vertical or horizontal black lines indicate the median of the y-axis or x-axis data.

The goal was to obtain large magnitudes of changes in proteomes changes and to be able to compare the results with literature data[5,16,17].

The biological replicate correlation rank coefficient (Spearman correlation) for each biological state ranged from 0.95 to 0.98 (Supplemental Figure 13), showing a good reproducibility of the cultivation and DIA method itself.

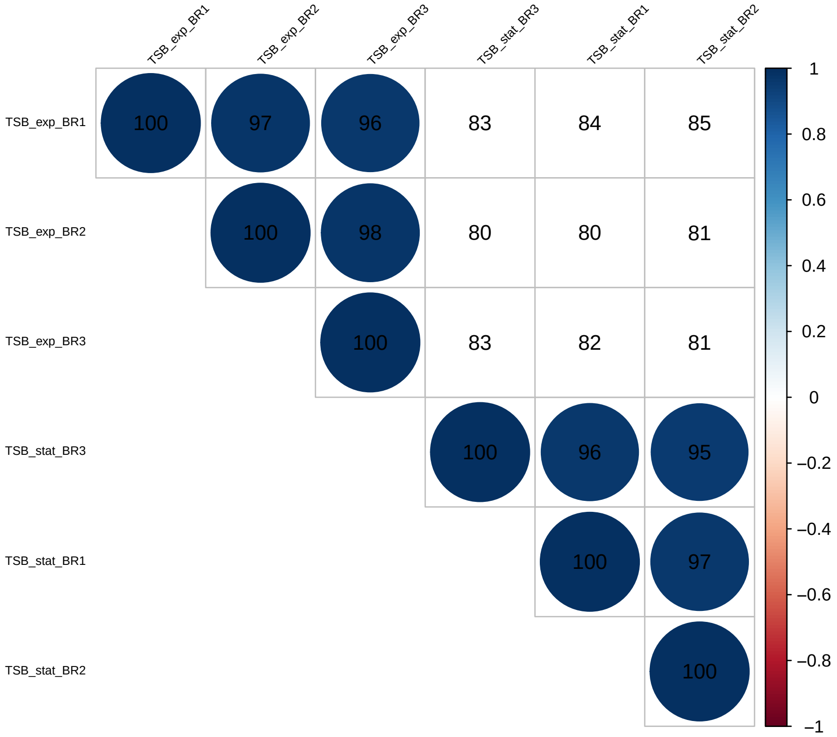

<u>Supplemental Figure 13:</u> Spearman correlation plot based on complete observations of the total peak area of the assays.
Three biological replicates (BR) of TSB exponential (exp.) and stationary (stat.) phase samples are displayed.

We were able to identify 1,565 proteins over all six samples of the experiment and to quantify 398 significant (Benjamini-Hochberg-adjusted p-value < 0.05) protein abandance changes (Supplemental Figure 14a) with an absolute fold-change of at least 1.5 comparing exponentially growing with stationary phase cells. The data is provided in Supplemental Table S3.

The findings greatly overlap (ca. 82% [517 proteins without changes out of 630 detected in both studies]) with previously generated data[5] in BioExpress® medium (Supplemental Figure 14c), a medium which is similar to TSB. The comparison with the mass spectrometry based publication of Becher and co-workers[5] with cells grown in Bioexpress® medium was favored over the 2D gel-based approach of Kohler and co-workers[16] with cells grown in TSB medium, because of a higher coverage of cytoplasmic proteins and a more robust protein quantification via $^{15}N/^{14}N$ labeling approach.

During the growth in glucose-rich complex media like TSB *S. aureus* utilizes glucose as its primary carbon source, which is rapidly consumed until entry into the stationary phase[16,18].

Glucose is preferentially metabolized via glycolysis, and acetate is produced and secreted as an overflow metabolite. As already reported by Kohler et al., 2005[16], glycolytic enzymes are highly abundant, and the TCC enzymes are repressed during the exponential growth phase[5,16]. The TCC enzymes (e.g. CitB, CitC, SucA/B, SucC/D, SdhA/B) were found to be strongly increased in their amount in the stationary growth phase[5,16,19] when glucose has been completely consumed. Glycolytic enzymes were decreased[5,16,19,20], whereas gluconeogenic enzymes (e.g. GapB, PckA) were found in elevated amounts[5,16].

Proteins involved in the transcription process such as the RNA polymerase subunits (RpoA, RpoB, RpoC, RpoE) and transcription factors like GreA and Rho were found in approx. 2-fold diminished amounts in the stationary growth phase as compared to the exponential growth phase. Furthermore, ribosomal proteins were found at a strongly reduced level in abundance in the non-growing/stationary phase cells[5,20], as well as ribosomal-associated factors such as the ribosome recycling factor Frr.

In addition, enzymes involved in cell wall biosynthesis (e.g. MurA, MurZ) and lipid synthesis (e.g. FabI, FabD) were also found in decreased amounts in the stationary phase. Furthermore, the initial and PurR-controlled part of the purine synthesis pathway was up-regulated (e.g. PurH, PurS, PurC, PurF, PurD), and exit enzymes of that pathway, which are central for important building blocks of the cell (e.g. Gmk, Hpt, Adk, NrdE, NrdF), were strongly diminished. For example, the nucleotide reductase, which has been described as highly unstable during the stationary phase[20], was diminished drastically in the stationary phase. On the other hand, Ndk, a nucleotide kinase, was up-regulated in the stationary phase, presumably to maintain the nucleotide triphosphate (NTP) pool for setting up the basic RNA synthesis. Regulating the pool of NTPs in the cell can alter translation, subsequently leading to cell reprogramming/adaption[21]. Arginine metabolism was modulated as indicated by the up-regulation of RocA, RocD, and RocF in the stationary phase.

Finally, histidine degradation enzymes (e.g. HutI, HutU, and HutG) were also found up-regulated in the stationary growth phase. Enzymes involved in one-carbon metabolism such as Fhs (formate-tetrahydrofolate-ligase), FolD (tetra-hydrofolate dehydrogenase), and SAOUHSC_01650 (5-formyltetrahydrofolate cyclo-ligase) and RibAB (riboflavin synthase) were found in increased amounts.

In addition to the CcpA (regulator of carbon catabolite control) regulated proteins like HutI-HutU-HutG and RocA-RocD-RocF, the FadA, FadB, FadD and FadE proteins, which are important for the fatty acid metabolism were found up-regulated in the stationary phase.
The ribosomal subunit interface protein, saHPF, *S. aureus* hibernation promoting factor[22], was found in a highly increased amount in the stationary phase. In a previous study[22] an increased formation of 100 S ribosomes with increasing amounts of HPF was observed. 100 S ribosomes are known to form “holding” ribosomes, which are decreased in their translational activity[23]. Interestingly, one ribosomal protein, RpsA (30S ribosomal protein S1), was found in an increased amount in the stationary phase. In other organisms S1 is essential for translation initiation, enabling the ribosome to dynamically initiate translation from a large set of mRNAs with diverse structural features[24,25].
Furthermore, several important regulators could be detected in altered amounts, including the SigB-dependent SpoVG[26] and virulence asscociated regulators like ArlR[27] and Rot[28]. WalR[29], a part of a two-component system and regulator for the cell wall metabolism was found in approx. 2fold increased amount in non-growing cells. The global effector of stress tolerance and biofilm formation[30], SpxA, was present in a 4fold higher amount in the stationary phase. Similar tendencies were recorded for other regulators like PhoP, IcaR, CtsR and SAOUHSC_02589, whereas MsrR, the cell envelope stress sensing regulatory protein[31], was detected in ~1.7-fold decreased amount.
By representing the most comprehensive insight to date into growing and non-growing *S. aureus* cells in the complex medium TSB, the data from the present study can be considered as nearly complete knowledge of *S. aureus* under standard laboratory *in vitro* growth conditions.

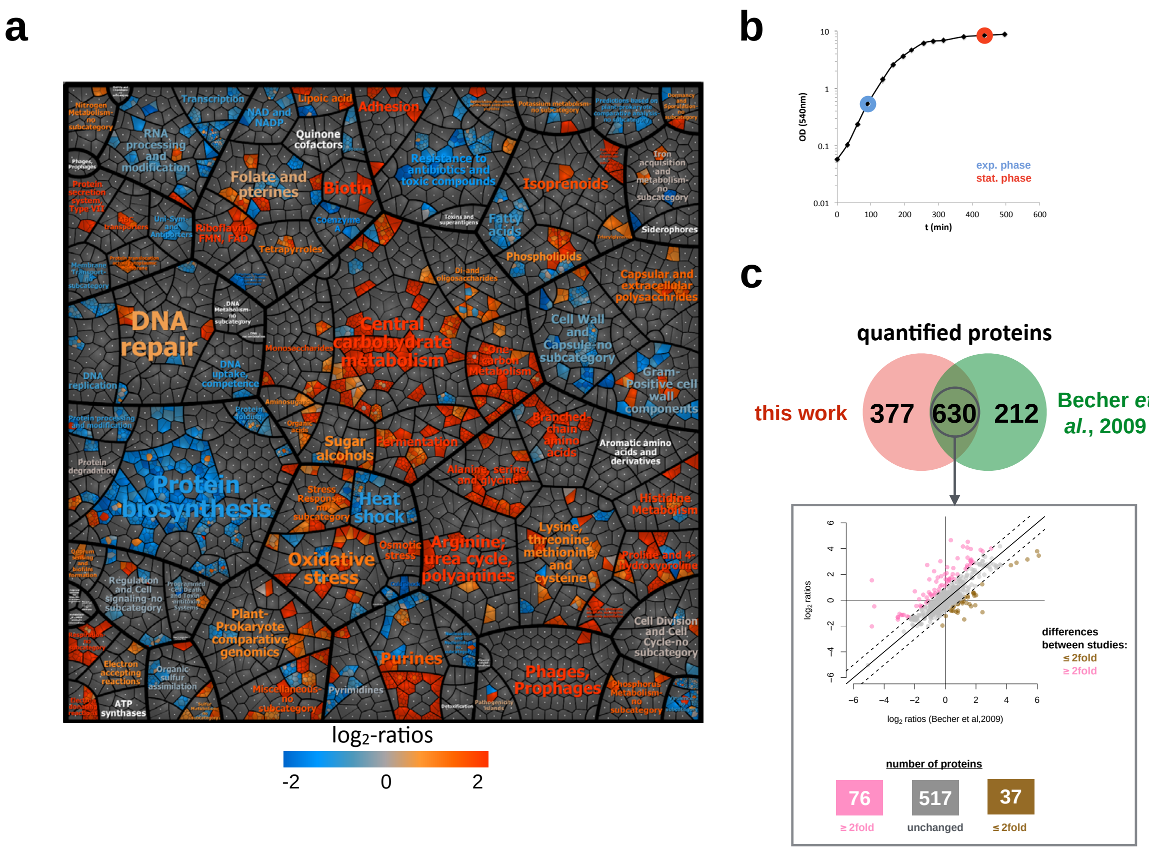

Supplemental Figure 14: DIA-MS analyses of *in vitro* shaking flask experiments with *S. aureus* HG001 cultivated in the rich medium TSB.

(a) Voronoi-like treemap comparing the proteome of exponentially growing and stationary phase *S. aureus* cells. A Voronoi-like treemap depicts the log_2-ratio of the peptide level between stationary and exponentially growing cells. Orange indicates an increased amount in the stationary phase and blue represents proteins which are found diminished in the stationary phase.

(b) Growth curve of *S. aureus* growing in TSB complex media. The exponential and stationary phase samples are marked by blue and orange circles in the growth curve.

(c) Comparison of protein abundance changes in growing and non-growing cells quantified via mass spectrometry in the present study and in a previously published study[5]. The Venn-diagram displays the differences and the intersection in protein identifications. The scatter plot reflects the quantitative ratios of the identification interaction. Proteins with a ratio variation below or above two-fold between the studies were colored pink (≥2-fold) or brown (≤2-fold).

3 Supplemental Material and Methods

3.1 Bacterial growth, optimized GFP strain construction, media and protein preparation, tryptic digestion, and peptide purification for samples subjected to DDA MS analysis and used for the generation of an *S. aureus* PeptideAtlas or a DIA library

3.1.1 Intra- and extracellular proteins from *S. aureus* HG001 pMV158GFP after iron limitation by 2,2'-bipyridyl and under control conditions

For the iron restricted cultivation *S. aureus* HG001 pMV158GFP was pre-cultured over night at 37°C in an adapted prokaryotic minimal essential medium (pMEM)[32,33] supplemented with 0.01% (w/v) yeast extract and 20 µg/ml tetracycline. The main cultures in pMEM supplemented with 600 µM 2,2'-bipyridyl to restrict the iron availability and in unsupplemented pMEM as control were inoculated from exponentially growing overnight cultures. Bacterial cell samples were taken in the exponential growth phase ($OD_{600\ nm}$ = 0.3) and 4 h after entry into the stationary growth phase. Extracellular proteins were prepared from sterile-filtrated culture supernatants by precipitation using a final concentration of 10% (v/v) trichloroacetic acid (TCA). Precipitated protein samples were washed four times with 80% (v/v) acetone and once with 100% (v/v) acetone and then dissolved in 50 mM ammonium bicarbonate (ABC)/0.1% (w/v) RapiGest® (Waters) by ultrasonication. Non-soluble contaminants were removed by centrifugation, and the protein concentration was determined using a Micro BCA™ Protein Assay Kit (Pierce). For the DDA MS analysis 2 µg of each extracellular proteome sample were digested with trypsin (1:25), the resulting peptides were purified using $ZipTip_{\mu\text{-}C18}$ pipette tips (Millipore, Schwalbach, Germany), and 1000 ng were subjected to LC-MS/MS. The mass spectrometric measurement of the intracellular proteome was performed using 2 x 10^8 cells treated with lysostaphin followed by tryptic digestion and peptide purification using $ZipTip_{C18}$ pipette tips (Millipore, Schwalbach, Germany).

3.1.2 Intra- and extracellular proteins from *S. aureus* HG001 growing in TSB at different growth phases

S. aureus HG001 was cultivated in tryptic soy broth (TSB) medium for DDA-MS analyses using QTOF (Agilent) and LTQ XL OrbiTrap (Thermo) instruments. Bacteria and culture supernatants were harvested by centrifugation during exponential growth, at entry into stationary phase (t_0), and 4 h after t_0. Protein samples from bacterial cells and from culture supernatants were prepared as previously described[3]. Equal amounts of protein from each of the three sampling points were mixed in order to obtain a single sample of intracellular proteins and another single sample of extracellular proteins. Protein samples were reduced, alkylated, tryptically digested, and purified using C18 columns as previously described[3]. Peptides resulting from the sample of intracellular proteins were subjected to fractionation according to the peptides' p*I* using an Agilent OFFGEL 3100 Fractionator system (Agilent Technologies, Santa Clara, CA, USA) and afterwards C18-purified as previously described[3].

3.1.3 Membrane proteins from *S. aureus* HG001 growing in TSB at different growth phases

The membrane fraction preparation was performed as described previously[34]. Briefly, cells were grown in TSB, and the same amount of cells was harvested during the exponential growth phase (OD_{540nm}=1) and after 4 h of the stationary phase (2,000 OD-units each). The pellets were washed with 5 ml TBS buffer (50 mM Tris, 150 mM NaCl, pH 8.0), mixed together, resuspended in lysis buffer (20 mM Tris, 10 mM $MgCl_2$, 1 mM $CaCl_2$, pH 7.5) containing cOmplete protease inhibitor (Roche), and disrupted by using a FastPrep® instrument (Thermo-Fisher). Addition of 200 U/ml DNase and 10 U/ml RNase followed by 20 min incubation at 37°C resulted in nucleic acid degradation. Centrifugation (8000 x g, 4°C, 10 min) separated cell debris and non-disrupted cells from the soluble proteins. The supernatant was used for ultra-centrifugation (100,000 x g, 4°C, 60 min), the pellet was washed with 6 ml ice-cold salt buffer (20 mM Tris, 1 M NaCl, pH 7.5) containing cOmplete protease inhibitor, followed by 1 h incubation at 4°C in an overhead shaker. A second ultra-centrifugation (100,000 x g, 4°C, 60 min) was then performed. Next, the pellet was washed with 6 ml ice-cold carbonate buffer (100 mM Na_2CO_3, 100 mM NaCl, pH 11) containing cOmplete protease inhibitor, followed by 1 h incubation at 4°C in an over-head shaker and subsequent ultra-centrifugation (100000 x g, 4°C, 60 min). The pellet was dissolved in 10% SDS, and protein concentration was determined using a Micro BCA™ Protein Assay Kit (Pierce). 20 µg of protein were used for a short gel-based separation, followed by an in-gel tryptic digestion[35] and ZipTip® peptide purification. Finally, iRT peptides (Biognosys) were added to the sample, which was subsequently analyzed using a Q Exactive™ mass spectrometer (Thermo-Fisher).

3.1.4 Intra- and extracellular proteins from *S. aureus* HG001 and its isogenic Δ*sigB* mutant

The preparation of the extracellular protein extracts of *S. aureus* HG001 and its isogenic Δ*sigB* mutant[36] was performed by removing the cells from the culture medium (TSB: exponential growth phase; 10 h into the stationary growth phase, and at 24 h; in the late stationary growth phase; or pMEM: exponential growth phase; 5 h into the stationary growth phase; and at 19 h in the late stationary growth phase) by centrifugation (15,557 x g, 4°C, 10 min). Extracellular proteins were precipitated according to Ziebandt et al.[37] with 10% w/v TCA overnight at 4°C with subsequent centrifugation (15,557 x g, 4°C, 1 h). The pellets were washed several times at room temperature with 70% ethanol and purified with 100% ethanol. Afterwards, the pellets were resolved in UT buffer (8 M urea, 2 M thiourea), and the protein concentration was determined with a Bradford-assay[38] according to the manufacturer's guide (Bio-Rad Laboratories GmbH, Munich, Germany). For gelfree analysis 2 µg of protein was diluted with 20 mM ABC to a final concentration of 0.8 M urea and 0.2 M thiourea followed by reduction with a final concentration of 2.5 mM dithiothreitol (final concentration)(GE Healthcare, Munich, Germany) in 20 mM ABC for 60 min at 60°C and alkylation with iodacetamide (Sigma) in 20 mM ammonium bicarbonate (final concentration 10 mM) for 30 min at 37°C in the dark. The samples were predigested with Lys C (Sigma) in an enzyme to substrate ratio of 1:100 for 3 h before trypsin (Promega, Madison, WI, USA) digestion at a ratio of 1:25 over night at 37°C was carried out. Thereafter, the digestion was stopped by adding 1% v/v purified acetic acid (Sigma), and the samples were purified using $ZipTip_{\mu\text{-}C18}$ columns (Millipore, Schwalbach, Germany). The eluates were used for LC-MS/MS analysis using a nano-HPLC-ESI-LTQ-FT instrument (Thermo Fisher Electron, Bremen, Germany).

3.1.5 Generation of S9 and *S. aureus* extract mixtures

S. aureus was grown in pMEM medium supplemented with 0.01% (w/v) yeast until mid-exponential phase (OD_{600nm}=0.4). The cells were harvested via centrifugation (20000 x g, 4°C, 5 min) and the pellet was washed twice in ice-cold PBS. The cells were re-suspended in 250 µl 20 mM ABC (ammonium bicarbonate) and were disrupted using liquid nitrogen and a bead mill (2min, 2600 rpm). The cell debris was removed by centrifugation (20000 x g, 4°C, 30 min). The protein concentration was determined using a Micro BCA™ Protein Assay Kit (Pierce). The protein concentration was adjusted to 1 µg/µl with 50 mM ABC. The S9 protein extract was generated by harvesting one confluent cell culture dish (diameter 10 cm). Cells were washed twice with PBS, scraped in 1 ml of PBS, pelleted by centrifugation (600 x g, 4°C, 5 min) and flash-frozen with liquid nitrogen and stored at -80°C. The cells were re-suspended in 250 µl 20 mM ABC and were disrupted using liquid nitrogen and a bead mill (2 min, 2600 rpm). The cell debris was removed by centrifugation (20000 x g, 4°C, 10 min). The protein

concentration was determined using a Micro BCA™ Protein Assay Kit (Pierce). The protein concentration was adjusted to 1 µg/µl with 50 mM ABC. Mixtures (100%, 50%, 25%, 10%, 5%, 0% of *S. aureus* protein) were generated using both extracts to a total protein content of 4 µg. The mixtures were digested using trypsin followed by ZipTip® peptide purification. The peptide mixtures were measured in DDA-MS and DIA-MS mode in a Q Exactive™.

3.1.6 Construction of *S. aureus* HG001 carrying optimized GFP

For the codon usage optimization of the GFPmut2 protein the web server OPTIMIZER[39] was used. To do so, the codon usage was optimized employing the codon usage of ribosomal proteins of *S. aureus* NCTC8325 and the guided random method, which is a Monte Carlo algorithm that selects codons at random, based on the frequencies of use of each codon in the reference set. The optimized sequence was synthesized by the company GenScript (Piscataway, NJ, USA) and replaced the initial sequence in plasmid pJL-sar-GFP[40] resulting in pJL-sar-GFPopt.

3.2 Mass spectrometric data acquisition using different LC instruments, MS devices, and acquisition methods

3.2.1 Mass spectrometric acquisition of TripleTOF DDA and SWATH data

S. aureus tryptic digests were analyzed on a TripleTOF® 5600+ mass spectrometer (Sciex, Framingham, MA, USA) equipped with an Eksigent ekspert™ nanoLC 425 and cHiPLC® system in trap-elute configuration. Samples were loaded on a cHiPLC column (75 µm x 15 cm ChromXP C18-CL, 3 µm, 120 Å) and washed for 30 min at 0.5 µl/min. Chromatographic separation was performed on a nano cHiPLC column (75 µm x 15 cm ChromXP C18-CL, 3 µm, 120 Å) using a gradient from 2-25% acetonitrile / 0.1% formic acid (v/v) for 180 min at a flow rate of 300 nL/min. Spectra were acquired in data-dependent mode in the mass range *m/z* 400-1250 with a 250 ms accumulation time. The 20 most intense ions with charge states 2-4 were selected for fragmentation with rolling collision energy and a collision energy spread of +/-5V. MS/MS spectra were collected in the range of *m/z* 100-2,000 with a 150 ms accumulation time upon a threshold of 100 cps. SWATH-MS data were collected with the MS/MS^ALL SWATH™ Acquisition method where Q1 was scanned from 400-1,200 Da and MS/MS acquired from 300-1500 Da. The Q1 transmission window was set at 26 Da wide with a 1 Da overlap with the previous window, 32 steps were used with a 100 ms MS/MS accumulation time for a total cycle time of 3.2 s. Further, samples were analyzed under slightly modified conditions by loading the cellular protein

lysate on a cHiPLC trap (200 µm x 500 µm ChromXP C18-CL, 3 µm, 120 Å) followed by a 10 min wash step at 2 µl/min and an elution gradient from 3-35% acetonitrile / 0.1% formic acid (v/v) for 120 min on a nano cHiPLC column (75 µm x 15 cm ChromXP C18-CL, 3 µm, 120 Å). MS/MS spectra in DDA mode were collected in the range of *m/z* 100-1,500 with a 150 ms accumulation time, and MS/MS in SWATH mode were acquired from *m/z* 100-2,000. All other parameters were set as described above.

3.2.2 Mass spectrometric acquisition of nano-HPLC-ESI-LTQ-FT data

Peptides were separated on a nanoAcquity UPLC reversed-phase column (BEH130, C18, 100 µm x 100 mm, Waters Corporation, Milford, USA) operated on a nanoAcquity UPLC system (Waters) using a gradient from 2% to 30% acetonitrile within 60 min at a flow rate of 400 nL/min. MS/MS data were recorded in a data-dependent mode for precursor ions with charge 2 or 3 using an LTQ-FT instrument (Thermo-Fisher Electron, Bremen, Germany). After a survey scan in the FT (scan *m/z* 300-1500 Da, resolution 50,000), MS/MS in the linear ion trap was triggered on the five most intensive ions exceeding a minimum threshold of 1,000. Target ions already selected for fragmentation were dynamically excluded for 90 s.

3.2.3 Mass spectrometric data-dependent-acquisition (DDA) analyses using a Q Exactive^{TM} instrument

MS analyses were performed with an on-line coupled UltiMate 3000RSLC system (Thermo-Fisher Scientific, Idstein, Germany) connected to a Q Exactive^{TM} Orbitrap-MS (Thermo-Fisher Scientific Inc.). For LC-separation peptides were enriched on an Acclaim PepMap 100, 100 µm × 2 cm, nanoViper C18, 5 µM, 100 Å pre-column (Thermo-Fisher Scientific Inc.) and separated using an Accucore 150-C18 with a column length of 25 cm respectively 50 cm (150 Å, 2.6 µm, Thermo Fisher Scientific Inc.) and a temperature of 40°C. For separation, a 120 min gradient was used with a solvent mixture of buffer A (5% acetonitrile (ACN) in water with 0.1% acetic acid) and increasing percentages of buffer B (ACN with 0.1% acetic acid): 2% for 10 min, 2–25% for 120 min, 25–40% for 5 min, 40–90% for 2 min, and 90% for 5 min. Peptides were eluted with a flow rate of 300 nl/min for the 25 cm column and 200 nl/min for the 50 cm column. Full-scan MS was carried out using a mass range of *m/z* 300 to 1,650. Data were acquired in a data-dependent strategy in profile mode with a resolution of 70,000 for MS at *m/z* 400 and 17,500 for MS/MS and a positive polarity. The method used allowed sequential isolation of the top ten most intense ions for fragmentation using high-energy collisional dissociation (HCD) with dynamic exclusion for 30 s and disabled early expiration. An intensity threshold of $8.3e^4$ was applied with an isolation width of 3 *m/z*, normalized collision energy of 27.5 eV, and a starting mass of *m/z* 100. The charge state screening and monoisotopic precursor selection rejected +1 and +7, +8, and >+8 charged ions.

3.2.4 Mass spectrometric data-independent-acquisition (DIA) analyses using a Q Exactive^{TM} instrument

LC-MS/MS analysis was performed on a Dionex UltiMate 3000 RSLC (Thermo-Fisher Scientific, Idstein, Germany) coupled to a QExactive mass spectrometer (Thermo Fisher Scientific, Waltham, MA, USA). The protein lysate digest was enriched on a 2 cm x 100 µm Acclaim PepMap 100 trap column (100 Å pore size, 5 µm C18 particles, Thermo-Fisher Scientific) and separated on a 50 cm x 75 µm Accucore 150-C18 analytical column (150 Å pore size, 2.6 µm particles, Thermo-Fisher Scientific) with a flow rate of 200 nl/min or a 25 cm x 75 µm Accucore 150-C18 analytical column (150 Å pore size, 2.6 µm particles, Thermo Fisher Scientific) with a flow rate of 300 nl/min at a constant temperature of 40°C. Reversed-phase chromatography was performed with a binary buffer system consisting of 0.1% acetic acid, 5% ACN in water (buffer A) and 100% ACN in 0.1% acetic acid (buffer B). The peptides were separated by applying a linear gradient from 2% to 25% buffer B over a time of 120 min and 180 min for the 25 cm and the 50 cm analytical column, respectively. Eluting peptides were ionized using the chip-based TriVersa NanoMate ion source (Advion Biosciences, Norwich, UK). For the data-independent mode the parameters from Bruderer et. al, 2013 were used[41].

3.3 Data processing for *S. aureus* PeptideAtlas construction, *S. aureus* and S9 cell ion library building, and DIA data analysis

3.3.1 Data processing for *S. aureus* PeptideAtlas construction

MS native data files in different proprietary formats were converted to mzXML or mzML using ProteoWizard 3.0.7364 msConvert[42]. MS/MS spectra were searched with Comet (2013.01 rev. 0)[43] against a reference database derived from NCBI (NCBI database year: 2014) comprising 2,891 *S. aureus* proteins. Common contaminants listed in the common repository of adventitious proteins (cRAP) and a sequence-shuffled decoy counterpart were appended to the database. For all data the parent mass error was set to ± 50 ppm and fragment mass error to 0.01 Da, and peptides were allowed to be semi-tryptic with up to two internal cleavage sites. The search parameters included either a fixed modification of +57.021464 for carbamidomethylated cysteine for reduced and alkylated samples or none for un-alkylated samples, and a variable modification of +15.9949 for oxidized methionine. For the SILAC host-pathogen internalization assay experiments, the variable modifications of +8.014199 at lysine and +10.008269 at arginine were searched. The search results were processed with the Trans-Proteomic Pipeline (TPP, version 4.6)[44] including PeptideProphet, iProphet, and ProteinProphet[45-48].

Peptide spectrum matches (PSM) derived from the database search were analyzed with PeptideProphet to assign each PSM a probability of being correct. The accurate mass binning model and the non-parametric model were used in the PeptideProphet analysis. Decoy hits were reported with a probability based on the model learned. PeptideProphet results were further processed with iProphet to refine the PSM-level probabilities and compute peptide-level probabilities based on corroborating information from the ensemble of identifications[48]. Finally, ProteinProphet was applied to obtain probabilities on the protein level based on the input peptide-level probabilities. A uniform PSM-level FDR threshold was applied to each experiment in order to achieve a protein-level FDR of approximately 1%. Data filtered at this threshold were assessed with MAYU[49] to calculate a decoy-based FDR at the PSM level (0.01%), peptide level (0.02%) and protein level (1.1%) for the *S. aureus* PeptideAtlas. The build named *S. aureus* HG001 2015-12 is available at www.peptideatlas.org. The *S. aureus* COL PeptideAtlas (*S. aureus* COL 2013-10, 0.04% peptide FDR, 0.22% protein FDR) is available as separate build.

3.3.2 Building an *S. aureus* fragment ion spectral library for DIA data analysis

MS native data files in different proprietary formats were converted to mzXML or mzML using ProteoWizard 3.0.7364 msconvert[42]. MS/MS spectra were searched with Comet (2014.02 rev. 0)[43] against a *S. aureus* NCTC8325 database comprising 2,891 proteins as described above for the *S. aureus* PeptideAtlas construction.

In addition to other essential information for building an ion library with the aim to analyze DIA data the retention time is a crucial factor. Therefore, all previously described high-accurate MS analysis runs[3] were separately aligned to a reference measurement set of six sample replicates with spiked-in iRT peptides.

For this purpose, the individual Comet-searched iProphet results were converted to a blib file (BiblioSpec algorithm description[50]) using ProteoWizard 3.0.7364[42] (iProphet cut-off score = 0.9). The resulting peptide error ranged from 0.0004 to 0.0053 with a median error of 0.003 for all files. The non-redundant blib files were used to generate a library using R[51] version 3.2.3 and the bioconductor package specL (1.5.2)[11] [parameter: max MZ error: 0.01 Da; TopN: 10; fragment ion *m/z* range: 300 - 2000; fragment ion type: b,y].

After generating the fragment ion spectral library, the peptide intersection of the ion library and the reference measurement set was compared for each file and used for retention time fitting. First, a linear regression of the sample retention time was carried out, and peptides with residuals between the 25% and the 75% quantile over all residuals were used for further analysis. Second, the ratio between the retention time of the intersecting reference set and the sample set was calculated and

used for performing a Loess fit model. Third, the fitted retention time values were used to calculate the adjusted retention time of the non-intersecting and quantile-filtered peptides.

The resulting aligned single-search ion library was filtered for 6-10 transitions per assay and peptides with two tryptic termini to obtain a robust ion library for the DIA data analysis. Redundant assays targeting the same peptide entries were filtered for the fusion of the single-search files: First, if the assays possessed equal numbers of transitions, the assay with the lowest *m/z* error was used and, second, if they did not possess the same number of transitions, the assay with the highest transition number was used. Furthermore, if an assay was found in both the iRT measurements and the non-iRT measurements and got a higher number of transitions or a lower *m/z* error in the non-iRT run, all the mass and charge information was used from the non-iRT run, and the retention time was used from the iRT run because it was empirically determined.

3.3.3 Building an S9 human cell line fragment ion spectral library for DIA data analysis

Nine measurements of S9 human cell line protein extract with spiked-in iRT peptides were searched against the human UniProt canonical database (last modified 10th of May 2015). Common contaminants listed in the common repository of adventitious proteins (cRAP), iRT peptides, and a sequence-shuffled decoy counterpart were appended to the database. For the search the parent mass error was set to ±50 ppm and peptides were allowed to be semitryptic with up to two internal cleavage sites. The search parameters included a fixed modification of +57.021464 for carbamidomethylated cysteine and a variable modification of +15.9949 for oxidized methionine. Downstream processing of the data was performed as described above for the *S. aureus* PeptideAtlas construction.

The individual Comet searched iProphet results were converted to a blib file using ProteoWizard 3.0.7364[42] (cut-off score = 0.9). The non-redundant blib files were used to generate a library using the bioconductor package specL (1.5.2)[11] [parameter: max MZ error: 0.01 Da; TopN: 10; fragment ion *m/z* range: 300-2,000; fragment ion type: b,y]. The final library was filtered for 6-10 transitions and the lowest fragment mass error.

3.3.4 DIA data analysis

The data analysis was performed using Spectronaut™ (v7.0.8065.3.13226 academic+)[52]. For TripleTOF® and Q Exactive™-derived assay comparison TSB exponential (exp.) and stationary (stat.) phase DIA measured samples were analyzed using Spectronaut™ (iRT factor = 1; interference correction = TRUE). The resulting data were used to plot the ratios of mean total peak areas (stat. / exp.) of assays (Q-value cut-off < 0.001) without missing values. The R-squared regression coefficient was calculated using the linear fit model on scattered data.

For the Spectronaut™ analysis the data was extracted with the settings as shown in Supplemental Table S2.

Only highly significant assays (Q-value < 0.001) were used for the analysis. The intensity of the samples was normalized on the assay ratio basis against the control. Ratios for each assay were calculated by generating the pairwise ratios from the experimental conditions versus the control in all combinations based on the replicates in each experiment. The obtained values were tested protein-wise using the Wilcox rank sum test against an absolute fold change of 1.5. The p-value was subsequently adjusted for multiple testing using Benjamini and Hochberg's method[53], and proteins with a p-value below 0.05 were assumed to be significantly regulated. The peptide and protein ratios were determined by using the median over the corresponding assay ratios.

The protein annotation and symbols used were from aureowiki (http://aureowiki.med.uni-greifswald.de/Main_Page).

3.3.5 DDA data analysis of S9 and *S. aureus* extract mixtures and TSB exponential and stationary phase samples

The DDA data analysis of the S9 and *S. aureus* proteome mixtures was performed using MaxQuant (v1.5.3.30) and a combined FASTA file containing human (www.uniprot.org / June 2015) and *S. aureus* (described above) proteins. The DDA data analysis of the TSB exponential and stationary phase samples was performed using MaxQuant (v1.5.3.30) and an *S. aureus* FASTA (described above).

For all data the first search mass error was set to ± 20 ppm and the main search mass error ± 4.5 ppm, and peptides were allowed to be full-tryptic with up to two internal cleavage sites. The search parameters included a variable modification of +15.9949 for oxidized methionine. Peptide false-discovery rate (FDR) was set to 0.001 and protein was set to a FDR of 0.01. The peptide and protein intensity data was used for median-median normalization over all samples. Followed by filtering for peptides uniquely belonging to *S. aureus*. For the DDA data analysis of the S9 and *S. aureus* proteome mixtures the two technical replicates were aggregated using the mean. The peptide intensities were 100 percent normalized to the pure (100%) *S. aureus* sample for each peptide.

3.3.6 DIA data analysis of S9 and *S. aureus* extract mixtures

The DIA data analysis of the S9 and *S. aureus* proteome mixtures was performed using Spectronaut™ and the S9 and *S. aureus* ion library (section 2.3.2 and 2.3.3). The analysis was performed using a Q-value of 0.001. The ion total peak was summed up for each peptide to obtain the peptide intensities. The peptide intensity data was used for median-median normalization over all samples. Followed by filtering for peptides uniquely belonging to *S. aureus*. The two technical replicates were aggregated

using the mean. The peptide intensities were 100 percent normalized to the pure (100%) *S. aureus* sample for each peptide.

3.3.7 R-packages used for the analysis and plotting

Package	***Author***	***Title***	***Version***
BiocParallel	Bioconductor Package Maintainer, Martin Morgan, Valerie Obenchain, Michel Lang, Ryan Thompson	Bioconductor facilities for parallel evaluation	v1.2.22
data.table	M. Dowle, A. Srinivasan, T. Short, S. Lianoglou; with contributions from R. Saporta, E. Antonyan	Extension of Data.frame	v1.9.6
gplots	Gregory R. Warnes, Ben Bolker, Lodewijk Bonebakker, Robert Gentleman, Wolfgang Huber, Andy Liaw, Thomas Lumley, Martin Maechler, Arni Magnusson, Steffen Moeller, Marc Schwartz, Bill Venables	Various R Programming Tools for Plotting Data	v2.17.0
ggplot2	Hadley Wickham	ggplot2: Elegant Graphics for Data Analysis	v2.2.1
made4	Aedin Culhane	Multivariate analysis of microarray data using ADE4	v1.42.0
MASS	Brian Ripley, Bill Venables, Douglas M. Bates, Kurt Hornik, Albrecht Gebhardt, David Firth	Support Functions and Datasets for Venables and Ripley's MASS	v7.3-45
micropan	Lars Snipen and Kristian Hovde Liland	micropan: Microbial Pan-Genome Analysis	1.0
plotrix	Jim Lemon, Ben Bolker, Sander Oom, Eduardo Klein, Barry Rowlingson, Hadley Wickham, Anupam Tyagi, Olivier Eterradossi, Gabor Grothendieck, Michael Toews, John Kane, Rolf Turner, Carl Witthoft, Julian Stander, Thomas Petzoldt, Remko Duursma, Elisa Biancotto, Ofir Levy, Christophe Dutang, Peter Solymos, Robby Engelmann, Michael Hecker, Felix Steinbeck, Hans Borchers, Henrik Singmann, Ted Toal, Derek Ogle	Various Plotting Functions	v3.6-1

protViz	Christian Panse, Jonas Grossmann, Simon Barkow-Oesterreicher	Visualizing and Analyzing Mass Spectrometry Related Data inProteomics	v0.2.9
RColorBrewer	Erich Neuwirth	ColorBrewer Palettes	v1.1-2
reshape	Hadley Wickham	Flexibly reshape data.	v0.8.5
scales	Hadley Wickham, RStudio	Scale Functions for Visualization	v0.3.0
seqinr	Delphine Charif, Jean R. Lobry, Anamaria Necsulea, Leonor Palmeira, Simon Penel, Guy Perriere	Biological Sequences Retrieval and Analysis	v3.1-3
sfsmisc	Martin Maechler *et al.*	Utilities from "Seminar für Statistik" ETH Zurich	v1.0-29
specL	Christian Trachsel, Christian Panse, Jonas Grossmann, Witold E. Wolski	specL - Prepare Peptide Spectrum Matches for Use in Targeted Proteomics	v1.5.2
stringi	Marek Gagolewski, Bartek Tartanus , IBM and other contributors, Unicode, Inc.	Character String Processing Facilities	v1.0-1
stringr	Hadley Wickham, RStudio	Simple, Consistent Wrappers for Common String Operations	v1.0.0

4 Supplemental legends

Supplemental Movie S1:

Live-cell imaging movie showing S. *aureus* behavior during infection of S9 human bronchial epithelial cells over time. Human S9 cells are displayed in the Nomarski Differential Interference Contrast (DIC) in transmitted-light channel (greyish), and the intracellular GFP-labeled *S. aureus* cells are shown in green.

5 Supplemental references

1. Desiere, F. *et al.* The PeptideAtlas project. *Nucleic Acids Res* **34,** D655–8 (2006).
2. Deutsch, E. W. *et al.* Trans-Proteomic Pipeline, a standardized data processing pipeline for large-scale reproducible proteomics informatics. *Proteomics Clin Appl* (2015). doi:10.1002/prca.201400164
3. Depke, M. *et al.* A peptide resource for the analysis of *Staphylococcus aureu*s in host-pathogen interaction studies. *PROTEOMICS* **15,** 3648–3661 (2015).
4. Surmann, K. *et al.* A proteomic perspective of the interplay of *Staphylococcus aureus* and human alveolar epithelial cells during infection. *Journal of Proteomics* **128,** 203–217 (2015).
5. Becher, D. *et al.* A proteomic view of an important human pathogen--towards the quantification of the entire *Staphylococcus aureus* proteome. *PLoS ONE* **4,** e8176 (2009).
6. Bernhardt, J., Funke, S., Hecker, M. & Siebourg, J. Visualizing Gene Expression Data via Voronoi Treemaps. *2009 Sixth International Symposium on Voronoi Diagrams* 233–241 (2009).
7. Bruderer, R., Bernhardt, O. M., Gandhi, T. & Reiter, L. High-precision iRT prediction in the targeted analysis of data-independent acquisition and its impact on identification and quantitation. *PROTEOMICS* **16,** 2246–2256 (2016).
8. Escher, C. *et al.* Using iRT, a normalized retention time for more targeted measurement of peptides. *PROTEOMICS* **12,** 1111–1121 (2012).
9. Cleveland, W. S. Robust Locally Weighted Regression and Smoothing Scatterplots. *Journal of the American Statistical Association* **74,** 829–836 (1979).
10. Podwojski, K. *et al.* Retention time alignment algorithms for LC/MS data must consider non-linear shifts. *Bioinformatics* **25,** 758–764 (2009).
11. Panse, C., Trachsel, C., Grossmann, J. & Schlapbach, R. specL--an R/Bioconductor package to prepare peptide spectrum matches for use in targeted proteomics. *Bioinformatics* **31,** 2228–2231 (2015).
12. de Graaf, E. L., Altelaar, A. F. M., van Breukelen, B., Mohammed, S. & Heck, A. J. R. Improving SRM assay development: a global comparison between triple quadrupole, ion trap, and higher energy CID peptide fragmentation spectra. *Journal of Proteome Research* **10,** 4334–4341 (2011).
13. Sun, L., Zhu, G. & Dovichi, N. J. Comparison of the LTQ-Orbitrap Velos and the Q-Exactive for proteomic analysis of 1-1000 ng RAW 264.7 cell lysate digests. *Rapid Commun. Mass Spectrom.* **27,** 157–162 (2013).
14. Zhang, Y. *et al.* Q Exactive – A True Qual-Quan HR/AM Mass Spectrometer for Routine Discovery and Target Quantification in Proteomics. *Proceedings 60th ASMS Conference on Mass Spectrometry 2011* 1–1 (2011).
15. Chuang, H.-Y., Hofree, M. & Ideker, T. A decade of systems biology. *Annu Rev Cell Dev Biol* **26,** 721–744 (2010).
16. Kohler, C. *et al.* Proteome analyses of *Staphylococcus aureus* in growing and non-growing cells: a physiological approach. *International Jl of Medical Microbiology* **295,** 547–565 (2005).
17. Hecker, M., Becher, D., Fuchs, S. & Engelmann, S. A proteomic view of cell physiology and virulence of *Staphylococcus aureus*. *Int J Med Microbiol* **300,** 76–87 (2010).

18. Kohler, C. *et al.* Physiological characterization of a heme-deficient mutant of *Staphylococcus aureus* by a proteomic approach. *J Bacteriol* **185,** 6928–6937 (2003).
19. Maass, S. *et al.* Efficient, global-scale quantification of absolute protein amounts by integration of targeted mass spectrometry and two-dimensional gel-based proteomics. *Anal Chem* **83,** 2677–2684 (2011).
20. Michalik, S. *et al.* Life and death of proteins: a case study of glucose-starved *Staphylococcus aureus. Mol Cell Proteomics* **11,** 558–570 (2012).
21. Schneider, D. A., Gaal, T. & Gourse, R. L. NTP-sensing by rRNA promoters in *Escherichia coli* is direct. *Proc Natl Acad Sci USA* **99,** 8602–8607 (2002).
22. Ueta, M., Wada, C. & Wada, A. Formation of 100S ribosomes in *Staphylococcus aureus* by the hibernation promoting factor homolog SaHPF. **15,** 43–58 (2010).
23. Polikanov, Y. S., Blaha, G. M. & Steitz, T. A. How hibernation factors RMF, HPF, and YfiA turn off protein synthesis. *Science* **336,** 915–918 (2012).
24. Duval, M. *et al. Escherichia coli* ribosomal protein S1 unfolds structured mRNAs onto the ribosome for active translation initiation. *PLoS Biol* **11,** e1001731 (2013).
25. Delvillani, F., Papiani, G., Deho, G. & Briani, F. S1 ribosomal protein and the interplay between translation and mRNA decay. *Nucleic Acids Res* **39,** 7702–7715 (2011).
26. Schulthess, B. *et al.* The B-Dependent yabJ-spoVG Operon Is Involved in the Regulation of Extracellular Nuclease, Lipase, and Protease Expression in *Staphylococcus aureus*. *J Bacteriol* **193,** 4954–4962 (2011).
27. Toledo-Arana, A. *et al. Staphylococcus aureus* develops an alternative, *ica*-independent biofilm in the absence of the *arlRS* two-component system. *J Bacteriol* **187,** 5318–5329 (2005).
28. Zhu, Y. *et al.* Structure of Rot, a global regulator of virulence genes in *Staphylococcus aureus*. *Acta Crystallogr D Biol Crystallogr* **70,** 2467–2476 (2014).
29. Dubrac, S., Boneca, I. G., Poupel, O. & Msadek, T. New insights into the WalK/WalR (YycG/YycF) essential signal transduction pathway reveal a major role in controlling cell wall metabolism and biofilm formation in *Staphylococcus aureus. J Bacteriol* **189,** 8257–8269 (2007).
30. Pamp, S. J., Frees, D., Engelmann, S., Hecker, M. & Ingmer, H. Spx is a global effector impacting stress tolerance and biofilm formation in *Staphylococcus aureus. J Bacteriol* **188,** 4861–4870 (2006).
31. Hübscher, J. *et al.* MsrR contributes to cell surface characteristics and virulence in *Staphylococcus aureus. FEMS Microbiol Lett* **295,** 251–260 (2009).
32. Depke, M. *et al.* Labeling of the pathogenic bacterium *Staphylococcus aureus* with gold or ferric oxide-core nanoparticles highlights new capabilities for investigation of host-pathogen interactions. *Cytometry A* **85,** 140–150 (2014).
33. Pförtner, H. *et al.* Activation of the alternative sigma factor SigB of *Staphylococcus aureus* following internalization by epithelial cells - an *in vivo* proteomics perspective. *Int J Med Microbiol* **304,** 177–187 (2014).
34. Dreisbach, A. *et al.* Profiling the surfacome of *Staphylococcus aureus. PROTEOMICS* **10,** 3082–3096 (2010).
35. Eymann, C. *et al.* A comprehensive proteome map of growing *Bacillus subtilis*

cells. *PROTEOMICS* **4,** 2849–2876 (2004).
36. Kullik, I., Giachino, P. & Fuchs, T. Deletion of the alternative sigma factor *sigmaB* in *Staphylococcus aureus* reveals its function as a global regulator of virulence genes. *J Bacteriol* **180,** 4814–4820 (1998).
37. Ziebandt, A.-K. *et al.* The influence of *agr* and *sigmaB* in growth phase dependent regulation of virulence factors in *Staphylococcus aureus. PROTEOMICS* **4,** 3034–3047 (2004).
38. Bradford, M. M. A rapid and sensitive method for the quantitation of microgram quantities of protein utilizing the principle of protein-dye binding. *Anal Biochem* **72,** 248–254 (1976).
39. Puigbò, P., Guzmán, E., Romeu, A. & Garcia-Vallvé, S. OPTIMIZER: a web server for optimizing the codon usage of DNA sequences. *Nucleic Acids Res* **35,** W126–31 (2007).
40. Liese, J., Rooijakkers, S. H. M., van Strijp, J. A. G., Novick, R. P. & Dustin, M. L. Intravital two-photon microscopy of host-pathogen interactions in a mouse model of *Staphylococcus aureus* skin abscess formation. *Cell Microbiol* **15,** 891–909 (2013).
41. Bruderer, R. M. *et al.* Comparison of DIA and Shotgun Quantitation on a Thermo Q Exactive. *http://www.biognosys.ch/fileadmin/Uploads/Application%20Notes/Biognosys_Application_Note_HRM_DIA_Q_Exactive.pdf* 1–3 (2013). Available at: http://www.biognosys.ch/fileadmin/Uploads/Application%20Notes/Biognosys_Application_Note_HRM_DIA_Q_Exactive.pdf. (Accessed: 24 April 2014)
42. Chambers, M. C. *et al.* A cross-platform toolkit for mass spectrometry and proteomics. *Nature Biotechnology* **30,** 918–920 (2012).
43. Eng, J. K., Jahan, T. A. & Hoopmann, M. R. Comet: an open-source MS/MS sequence database search tool. *PROTEOMICS* **13,** 22–24 (2013).
44. Deutsch, E. W. *et al.* A guided tour of the Trans-Proteomic Pipeline. *PROTEOMICS* **10,** 1150–1159 (2010).
45. Keller, A., Nesvizhskii, A. I., Kolker, E. & Aebersold, R. Empirical statistical model to estimate the accuracy of peptide identifications made by MS/MS and database search. *Anal. Chem.* **74,** 5383–5392 (2002).
46. Nesvizhskii, A. I., Keller, A., Kolker, E. & Aebersold, R. A statistical model for identifying proteins by tandem mass spectrometry. *Anal. Chem.* **75,** 4646–4658 (2003).
47. Keller, A., Eng, J., Zhang, N., Li, X.-J. & Aebersold, R. A uniform proteomics MS/MS analysis platform utilizing open XML file formats. *Mol Syst Biol* **1,** 2005.0017 (2005).
48. Shteynberg, D. *et al.* iProphet: multi-level integrative analysis of shotgun proteomic data improves peptide and protein identification rates and error estimates. *Molecular & Cellular Proteomics* **10,** M111.007690 (2011).
49. Reiter, L. *et al.* Protein identification false discovery rates for very large proteomics data sets generated by tandem mass spectrometry. *Molecular & Cellular Proteomics* **8,** 2405–2417 (2009).
50. Frewen, B. & MacCoss, M. J. Using BiblioSpec for creating and searching tandem MS peptide libraries. *Curr Protoc Bioinformatics* **Chapter 13,** Unit 13.7 (2007).
51. Team, R. D. C. R: A Language and Environment for Statistical Computing. GNU

General Public License (GPL) – http://www.gnu.org/licenses/old–licenses/gpl–2.0.html (2014).

52. Bernhardt, O. M., Selevsek, N. & Gillet, L. C. Spectronaut: A fast and efficient algorithm for MRM-like processing of data independent acquisition (SWATH-MS) data. *Proceedings 60th ASMS Conference on Mass Spectrometry* (2012).

53. Benjamini, Y. & Hochberg, Y. Controlling the false discovery rate: a practical and powerful approach to multiple testing. *Journal of the Royal Statistical Society. Series B (Methodological)* **57,** 289–300 (1995).

6 Supplemental tables

Supplemental Table S1 (page: 41-45):

Overview of the DDA measurements (a) used for the PeptideAtlas and ion library generation and of the DIA measurements (b) used for the analysis of biological samples.

Supplemental Table S2 (page: 46):

Parameters used for the Spectronaut^{TM} (Biognosys, Schlieren/Zurich, Switzerland) DIA analysis.

Supplemental Table S3 (page: 47-50):

Significantly regulated proteins (Benjamini-Hochberg corrected p-value < 0.05; absolute fold-change > 1.5) on comparing exponential and stationary growth phase of *S. aureus* HG001 cells grown in tryptic soy broth. Orange depicts up-regulation, blue visualizes down-regulation in the stationary phase. In addition the table contains also ratios from exponential and stationary growth phase of *S. aureus* COL cells grown in BioExpress medium extracted from Becher et al., 2009[5].

Supplemental Table S4 (page: 51-57):

Significantly regulated proteins (Benjamini-Hochberg corrected p-value < 0.05; absolute fold-change > 1.5) of the S9 human bronchial epithelial cell infection model. Orange depicts up-regulation, blue visualizes down-regulation in host-cell-internalized *S. aureus* as compared to the non-adherent cell control.

Supplemental Table S5 (page: 58-64):

Comparison of intracellularly growing cells (8h post-infection) versus non-adherent control and intracellularly growing cells to 24h or 32h post-infection.

Significantly regulated proteins (Benjamini-Hochberg corrected p-value < 0.05; absolute fold-change > 1.5) of the S9 human bronchial epithelial cell infection model. Orange depicts up-regulation, blue visualizes down-regulation in host-cell-internalized *S. aureus* as compared to the non-adherent cell control.

Supplemental Table S6 (page: 65-77):

Proteins detected in samples of the murine pneumonia infection model and S9 cell infection model (at least 2 peptides per protein and in one sample). The log_2-ratios against the individual control (murine pneumonia model = pre-infection; S9 cell infection model = non-adherent bacteria after 1 h) were colored using a blue to orange color space. Bluish color indicates a lower and orange color indicates a higher level as compared to the control. The colored arrows display the median tendency. For example, if the arrow points "up" the median of the 8, 24, 32 h p.i. samples is more than 1.5-fold higher as compared to the control.

Supplemental Table S7 (page: 78):

List of recombinant *S. aureus* proteins measured for incorporation into the ion library.

Supplemental Table S1A: Data sets used for the generation of *S. aureus* PeptideAtlas (DDA samples)

S. aureus strain	sample material and cultivation conditions	sampling points	biological replicates	fractionation	MS instrument type	number of raw files	used for DIA library	reference
HG001, HG001 Δ*sigB*	culture supernatants from rich medium TSB, culture supernatants from minimal medium pMEM	exponential growth, stationary growth (TSB:10 h; pMEM: 5 h), late stationary growth (TSB: 24 h, pMEM: 19 h)	4	non-fractionated	ESI-LTQ-FT	48	NO	this paper
HG001	bacteria from cultivation in rich medium TSB	mixed sample from exponential growth, entry into stationary growth phase, and stationary growth phase	3	non-fractionated<br>off-gel electrophoresis/pI (peptides)<br>SCX (peptides)	Q Exactive™	3<br>120<br>21	YES	Depke, Michalik et al. Proteomics, 2015
HG001	bacteria from cultivation in rich medium TSB	mixed sample from exponential growth, entry into stationary growth phase, and stationary growth phase	1	non-fractionated	Q Exactive™	6	YES	this paper

HG001	bacteria from cultivation in rich medium TSB	mixed sample from exponential growth, entry into stationary growth phase, and stationary growth phase	1	non-fractionated	TripleTOF®	12	YES	this paper
HG001	extracellular and intracellular proteome from unstressed cells growing in pMEM and from cells during iron limitation by adding 2,2′-bipyridyl	exponential and stationary growth phase; intracellular and extracellular proteome	3	non-fractionated	Q Exactive™	24	YES	this paper
HG001	bacteria from cultivation in rich medium TSB	mixed sample from exponential growth, entry into stationary growth phase, and stationary growth phase	1	membrane fraction	Q Exactive™	4	YES	this paper
HG001	purified recombinant proteins; overexpressed and purified His-tagged *S. aureus* proteins in *E. coli* SCS1	-		non-fractionated	Q Exactive™	4	YES	this paper
HG001	bacteria from cultivation in rich medium TSB	mixed sample from exponential growth, entry into stationary growth phase, and	1	off-gel electrophoresis/pI (peptides)	QTOF	24	NO	this paper
		stationary growth phase						
HG001	bacteria from cultivation in rich medium TSB	mixed sample from exponential growth, entry into stationary growth phase, and	1	off-gel electrophoresis/pI (peptides)	LTQ XL OrbiTrap	24	NO	this paper

		stationary growth phase						
HG001	culture supernatants from rich medium TSB	extracellular proteome; mixed sample from supernatants of exponential growth, entry into stationary growth phase, and stationary growth phase	1	non-fractionated	QTOF	1	NO	this paper
HG001	culture supernatants from rich medium TSB	extracellular proteome; mixed sample from supernatants of exponential growth, entry into stationary growth phase, and stationary growth phase	1 (measured in 2 technical replicates)	non-fractionated	LTQ XL OrbiTrap	2	NO	this paper
HG001 pMV158-GFP	internalized bacteria from cell culture infection model using human alveolar A549 cells and corresponding control cells	1.5 h, 2.5 h, 3.5 h, 4.5 h, 5.5 h, and 6.5 h after internalization into A549 cells; 1 h extracellular non-adherent bacteria; 1.5 h medium control; ^{13}C SILAC control	3	non-fractionated	Orbitrap Velos	27	NO	Surmann et al. J Prot, 2015
COL	bacteria from cultivation in BioExpress® 1000 medium and culture supernatants	exponential growth, stationary growth (5 h)	4	cell surface proteins, 1D gel (proteins)<br>cytosolic proteins, 1D gel (proteins)<br>extracellular proteins, 1D gel (proteins)	LTQ-Orbitrap	40<br>100<br>44	NO	Becher et al. PLoS One, 2009

				membrane proteins, 1D gel (proteins)		48	

Supplemental Table S1B: samples generated for DIA analysis

S. aureus strain	sample material and cultivation conditions	sampling points	biological replicates	fractionation	MS instrument type	number of raw files	reference
HG001	bacteria from cultivation in rich medium TSB	exponential growth, stationary growth (4 h)	3	non-fractionated	Q Exactive™	6	this paper
HG001	bacteria from cultivation in rich medium TSB	mixed sample from exponential growth, entry into stationary growth phase, and stationary growth phase	1	non-fractionated	Q Exactive™	3	this paper
HG001	bacteria from cultivation in rich medium TSB	mixed sample from exponential growth, entry into stationary growth phase, and stationary growth phase	1	non-fractionated	TripleTOF®	3	this paper
HG001	bacteria from cultivation in rich medium TSB	mixed sample from exponential growth, entry into stationary growth phase, and stationary growth phase<br>5ng, 10ng, 50ng, 100ng, 500ng, 1000ng, 2000ng, 4000ng on-column load	1 (measured in 2 technical replicates)	non-fractionated	Q Exactive™	16	this paper
HG001	bacteria from cultivation in rich medium TSB	mixed sample from exponential growth, entry into stationary growth phase, and stationary growth phase<br>on-column load of peptides from 1E4, 1E5, 5E5, 1E6, 5E6, 1E7 *S. aureus* cells	1 (measured in 2 technical replicates)	non-fractionated	Q Exactive™	12	this paper
HG001	S9 cell line infection experiment	OD=0.4 in pMEM, non-adherent *S.aureus* cells, S9 internalized *S. aureus* 8 h, 24 h, 32 h p.i.	3 or 4	non-fractionated	Q Exactive™	19	this paper

HG001	murine infection model	pre-infection *S.aureus* cells, *S.aureus* from lavage preparation 8 h, 24 h, 32 h p.i.	3, finally combined to one sample	non-fractionated	Q Exactive™	6	this paper

Supplemental table S2: Spectronaut™ parameters

**peak detection**	XIC extraction window: dynamic window<br>correction factor = 1
**identification**	dynamic score refinement = TRUE<br>processing base entity = RUN<br>generate decoys = TRUE<br>decoy generation method = workflow specific decoy generator<br>include MS1 scoring = TRUE
**calibration strategy**	iRT calibration strategy: linear iRT calibration
**quantification**	interference correction: TRUE<br>cross run normalization: FALSE

supplemental table S3: Significant regulated proteins in comparison of TSB exponentially and stationary phase S. aureus

locus tag	protein symbol	description	theSEED functional category	median fold-change	median fold-change (direction)	median ratio over assays (TSB stat. /exp.)	IQR	p-value BH adjusted	log2 median ratio over assays (TSB stat. /exp.)	log2 ratio Becher *et al.*, 2009	locus tag (COL)
SAOUHSC_00487	HslO	Hsp33-like chaperonin	Adhesion	2.28	down	0.44	0.080	1.68E-08	-1.19	n.d.	SACOL0556
SAOUHSC_00544	SdrC	sdrC protein	Adhesion	6.70	up	6.70	3.851	4.04E-02	2.74	n.d.	SACOL0608
SAOUHSC_00812	ClfA	clumping factor	Adhesion	17.79	up	17.79	14.242	1.56E-05	4.15	n.d.	SACOL0856
SAOUHSC_01501	EbpS	elastin binding protein	Adhesion	3.56	up	3.56	1.194	6.22E-27	1.83	n.d.	SACOL1522
SAOUHSC_02990	SraP	hypothetical protein	Adhesion	7.90	up	7.90	8.565	1.12E-04	2.98	n.d.	SACOL2676
SAOUHSC_01633	GcvPA	glycine dehydrogenase subunit 1	Alanine, serine, and glycine	3.34	up	3.34	1.257	5.85E-14	1.74	1.74	SACOL1594
SAOUHSC_01634	GcvT	glycine cleavage system aminomethyltransferase T	Alanine, serine, and glycine	4.46	up	4.46	2.428	1.21E-12	2.16	1.83	SACOL1595
SAOUHSC_00532	Kbl	2-amino-3-ketobutyrate coenzyme A ligase	Alanine, serine, and glycine;Lysine, threonine, methionine, and cysteine	4.01	up	4.01	1.822	1.58E-18	2.00	1.71	SACOL0596
SAOUHSC_00535		hypothetical protein	Alanine, serine, and glycine;Lysine, threonine, methionine, and cysteine	5.09	up	5.09	2.347	4.06E-08	2.35	1.83	SACOL0599
SAOUHSC_02564	UreG	urease accessory protein UreG	Arginine; urea cycle, polyamines	1.68	up	1.68	0.527	2.71E-02	0.75	-0.10	SACOL2285
SAOUHSC_00894	RocD	ornithine--oxo-acid transaminase	Arginine; urea cycle, polyamines;Proline and 4-hydroxyproline	14.02	up	14.02	11.229	4.54E-10	3.81	n.d.	SACOL0960
SAOUHSC_01809	AccD	acetyl-CoA carboxylase subunit beta	Bacteriocins, ribosomally synthesized antibacterial peptides;Fatty acids	1.80	down	0.55	0.262	5.05E-04	-0.85	-0.69	SACOL1748
SAOUHSC_01014	PurF	amidophosphoribosyltransferase	Bacteriocins, ribosomally synthesized antibacterial peptides;Purines	6.51	up	6.51	6.903	8.55E-10	2.70	3.30	SACOL1079
SAOUHSC_00198	FadE	hypothetical protein	Biotin	20.18	up	20.18	3.647	4.04E-02	4.34	n.d.	SACOL0214
SAOUHSC_00195	FadA	acetyl-CoA acetyltransferase	Biotin;Branched-chain amino acids;Fatty Acids, Lipids, and Isoprenoids - no subcategory;One-carbon Metabolism	49.41	up	49.41	9.988	4.04E-02	5.63	n.d.	SACOL0211
SAOUHSC_02589		hypothetical protein	Capsular and extracellular polysacchrides	3.35	up	3.35	1.497	4.04E-02	1.75	n.d.	SACOL2308
SAOUHSC_01462	GpsB	hypothetical protein	Cell Division and Cell Cycle - no subcategory	3.05	up	3.05	1.080	5.82E-12	1.61	0.25	SACOL1484
SAOUHSC_02337	MurA1	UDP-N-acetylglucosamine 1-carboxyvinyltransferase	Cell Wall and Capsule - no subcategory	2.22	down	0.45	0.148	1.16E-22	-1.15	-0.61	SACOL2092
SAOUHSC_02365	MurA2	UDP-N-acetylglucosamine 1-carboxyvinyltransferase	Cell Wall and Capsule - no subcategory	1.61	down	0.62	0.177	2.27E-02	-0.69	-0.43	SACOL2116
SAOUHSC_01373	FemA	methicillin resistance factor, FemA	Cell Wall and Capsule - no subcategory;Resistance to antibiotics and toxic compounds	2.48	down	0.40	0.185	8.26E-13	-1.31	n.d.	SACOL1410
SAOUHSC_00153		indolepyruvate decarboxylase	Central carbohydrate metabolism	4.02	up	4.02	1.141	4.24E-13	2.01	-0.03	SACOL0173
SAOUHSC_01064	PycA	pyruvate carboxylase	Central carbohydrate metabolism	2.60	up	2.60	1.047	5.64E-48	1.38	0.42	SACOL1123
SAOUHSC_01418	SucA	2-oxoglutarate dehydrogenase E1 component	Central carbohydrate metabolism	8.24	up	8.24	6.819	1.44E-11	3.04	2.44	SACOL1449
SAOUHSC_01801	CitC	isocitrate dehydrogenase	Central carbohydrate metabolism	8.03	up	8.03	5.035	3.35E-17	3.01	2.42	SACOL1741
SAOUHSC_01818	Ald2	alanine dehydrogenase	Central carbohydrate metabolism	6.78	up	6.78	5.716	4.24E-13	2.76	3.57	SACOL1758
SAOUHSC_01846	AcsA	acetyl-CoA synthetase	Central carbohydrate metabolism	14.17	up	14.17	5.024	1.56E-05	3.82	6.83	SACOL1783
SAOUHSC_01983	FumC	fumarate hydratase	Central carbohydrate metabolism	6.21	up	6.21	3.311	1.88E-14	2.64	2.74	SACOL1908
SAOUHSC_02647	Mqo1	malate:quinone oxidoreductase	Central carbohydrate metabolism	9.56	up	9.56	4.919	1.87E-09	3.26	0.80	SACOL2362
SAOUHSC_02703	GpmA	phosphoglyceromutase	Central carbohydrate metabolism	5.34	up	5.34	2.378	2.71E-15	2.42	1.70	SACOL2415
SAOUHSC_01416	SucB	dihydrolipoamide succinyltransferase	Central carbohydrate metabolism;Lipoic acid	10.70	up	10.70	24.419	6.60E-14	3.42	2.47	SACOL1448
SAOUHSC_01216	SucC	succinyl-CoA synthetase subunit beta	Central carbohydrate metabolism;One-carbon Metabolism	7.07	up	7.07	5.345	4.07E-24	2.82	2.25	SACOL1262
SAOUHSC_01218	SucD	succinyl-CoA synthetase subunit alpha	Central carbohydrate metabolism;One-carbon Metabolism	6.80	up	6.80	3.829	2.18E-10	2.77	2.23	SACOL1263
SAOUHSC_01910	PckA	phosphoenolpyruvate carboxykinase	Central carbohydrate metabolism;One-carbon Metabolism	11.75	up	11.75	3.896	8.80E-11	3.55	n.d.	SACOL1838
SAOUHSC_00866		D-isomer specific 2-hydroxyacid dehydrogenase NAD binding domain-containing	Central carbohydrate metabolism;Organic acids	1.71	up	1.71	0.549	8.37E-03	0.77	-0.13	SACOL0932
SAOUHSC_02577		D-isomer specific 2-hydroxyacid dehydrogenase NAD binding domain-containing	Central carbohydrate metabolism;Organic acids	4.44	up	4.44	1.415	5.95E-19	2.15	2.20	SACOL2296
SAOUHSC_00795	GapA	glyceraldehyde-3-phosphate dehydrogenase, type I	Central carbohydrate metabolism;Oxidative stress;Pyridoxine	1.75	down	0.57	0.180	5.98E-07	-0.81	-0.92	SACOL0838
SAOUHSC_00132	AldA	aldehyde dehydrogenase	Central carbohydrate metabolism;Phospholipids	6.54	up	6.54	0.514	4.04E-02	2.71	4.28	SACOL0154
SAOUHSC_02142	AldH	aldehyde dehydrogenase	Central carbohydrate metabolism;Phospholipids	2.77	up	2.77	1.175	7.36E-16	1.47	1.10	SACOL1984
SAOUHSC_01075	CoaD	phosphopantetheine adenylyltransferase	Coenzyme A	2.28	down	0.44	0.166	2.15E-06	-1.19	-0.39	SACOL1134
SAOUHSC_02918	PanC	pantoate--beta-alanine ligase	Coenzyme A	3.88	down	0.26	0.088	1.15E-12	-1.95	-0.19	SACOL2614
SAOUHSC_01824	ThiI	thiamine biosynthesis protein ThiI	Cofactors, Vitamins, Prosthetic Groups, Pigments - no subcategory	3.65	down	0.27	0.061	1.52E-07	-1.87	-1.59	SACOL1764
SAOUHSC_00819	CspC	hypothetical protein	Cold shock	4.78	down	0.21	0.113	1.06E-07	-2.26	n.d.	SACOL0861
SAOUHSC_00437	TreP	hypothetical protein	Di- and oligosaccharides	2.58	up	2.58	0.750	5.79E-03	1.37	n.d.	SACOL0516
SAOUHSC_02848	GlcB	PTS system glucose-specific transporter subunit IIABC	Di- and oligosaccharides	2.08	up	2.08	1.011	6.38E-03	1.06	n.d.	SACOL2552
SAOUHSC_00776	UvrB	excinuclease ABC, B subunit	DNA repair	2.38	up	2.38	2.155	1.22E-04	1.25	0.07	SACOL0823
SAOUHSC_00779	UvrB2	excinuclease ABC subunit B	DNA repair	2.99	up	2.99	0.617	3.43E-10	1.58	n.d.	
SAOUHSC_01658	Nfo	endonuclease IV	DNA repair	1.85	down	0.54	0.163	1.51E-03	-0.89	-0.50	SACOL1614
SAOUHSC_02123	PcrA	ATP-dependent DNA helicase PcrA	DNA repair	1.99	down	0.50	0.219	3.11E-05	-0.99	-1.44	SACOL1966
SAOUHSC_02861		methylated-DNA--protein-cysteine methyltransferase	DNA repair	3.73	up	3.73	1.541	3.00E-05	1.90	1.41	SACOL2562
SAOUHSC_01241	PolC	DNA polymerase III PolC	DNA replication	1.81	down	0.55	0.215	1.80E-04	-0.85	-0.89	SACOL1283
SAOUHSC_01351	ParE	DNA topoisomerase IV subunit B	DNA replication;Resistance to antibiotics and toxic compounds	1.89	down	0.53	0.164	5.79E-19	-0.92	n.d.	SACOL1389
SAOUHSC_01352	ParC	DNA topoisomerase IV subunit A	DNA replication;Resistance to antibiotics and toxic compounds	1.68	down	0.60	0.194	9.80E-04	-0.74	-0.89	SACOL1390
SAOUHSC_02517	TopB	DNA topoisomerase III	DNA uptake, competence	2.10	down	0.48	0.159	6.70E-12	-1.07	n.d.	SACOL2243
SAOUHSC_00469	SpoVG	regulatory protein SpoVG	Dormancy and Sporulation - no subcategory	8.59	up	8.59	4.374	4.22E-06	3.10	n.d.	SACOL0541
SAOUHSC_00483		hypothetical protein	Dormancy and Sporulation - no subcategory	2.19	down	0.46	0.142	1.56E-05	-1.13	-1.71	SACOL0552
SAOUHSC_00875		hypothetical protein	Electron donating reactions	3.05	up	3.05	1.427	5.04E-13	1.61	n.d.	SACOL0941
SAOUHSC_01104	SdhA	succinate dehydrogenase flavoprotein subunit	Electron donating reactions;Central carbohydrate metabolism;One-carbon Metabolism	9.43	up	9.43	7.235	7.36E-16	3.24	3.04	SACOL1159
SAOUHSC_01105	SdhB	succinate dehydrogenase iron-sulfur subunit	Electron donating reactions;One-carbon Metabolism;Central carbohydrate metabolism	7.26	up	7.26	3.578	8.06E-04	2.86	2.95	SACOL1160
SAOUHSC_00920	FabH	3-oxoacyl-(acyl carrier protein) synthase III	Fatty acids	1.76	down	0.57	0.166	3.34E-06	-0.81	-1.05	SACOL0987
SAOUHSC_00947	FabI	enoyl-(acyl carrier protein) reductase	Fatty acids	1.94	down	0.52	0.278	1.74E-03	-0.96	-0.06	SACOL1016
SAOUHSC_01198	FabD	malonyl CoA-acyl carrier protein transacylase	Fatty acids	2.00	down	0.50	0.125	5.34E-11	-1.00	-0.80	SACOL1244
SAOUHSC_01199	FabG	3-oxoacyl-(acyl-carrier-protein) reductase	Fatty acids	1.76	down	0.57	0.209	5.61E-08	-0.81	-0.94	SACOL1245
SAOUHSC_01623	AccC	acetyl-CoA carboxylase biotin carboxylase subunit	Fatty acids;Miscellaneous - no subcategory	1.76	down	0.57	0.217	1.28E-02	-0.82	-0.37	SACOL1571
SAOUHSC_00086	ButA	acetoin reductase	Fermentation	18.03	up	18.03	8.075	4.24E-13	4.17	1.15	SACOL0111
SAOUHSC_00206	Ldh1	L-lactate dehydrogenase	Fermentation	2.92	up	2.92	2.320	1.51E-11	1.54	-4.80	SACOL0222
SAOUHSC_02922	Ldh2	L-lactate dehydrogenase	Fermentation	1.98	up	1.98	0.696	1.96E-08	0.98	0.68	SACOL2618
SAOUHSC_00196	FadB	hypothetical protein	Fermentation;Branched-chain amino acids;Fatty Acids, Lipids, and Isoprenoids - no subcategory	27.52	up	27.52	7.802	2.81E-12	4.78	n.d.	SACOL0212
SAOUHSC_02468	BudB	acetolactate synthase	Fermentation;Organic acids;Programmed Cell Death and Toxin-antitoxin Systems	2.03	up	2.03	0.955	2.22E-09	1.02	n.d.	SACOL2199
SAOUHSC_00608	Adh	alcohol dehydrogenase	Fermentation;Phospholipids	3.88	up	3.88	3.086	1.57E-03	1.96	n.d.	SACOL0660
SAOUHSC_00187	PflB	formate acetyltransferase	Fermentation;Respiration - no subcategory	7.11	up	7.11	5.947	3.00E-05	2.83	n.d.	SACOL0204
SAOUHSC_02542	MoeA	molybdopterin biosynthesis protein moeA	Folate and pterines	2.93	up	2.93	0.799	6.23E-10	1.55	0.09	SACOL2266
SAOUHSC_02544	MoaB	molybdopterin precursor biosynthesis moaB	Folate and pterines	2.05	up	2.05	0.581	1.49E-08	1.04	0.25	SACOL2268
SAOUHSC_02549	ModA	molybdenum ABC transporter periplasmic molybdate-binding protein	Folate and pterines	3.02	up	3.02	1.417	7.68E-11	1.59	n.d.	SACOL2272
SAOUHSC_02360	Tdk	thymidine kinase	Folate and pterines;Pyrimidines	2.41	down	0.42	0.074	7.75E-08	-1.27	-0.97	SACOL2111
SAOUHSC_00549	FolE2	putative GTP cyclohydrolase	Folate and pterines;RNA processing and modification	1.79	down	0.56	0.211	3.18E-03	-0.84	n.d.	SACOL0613
SAOUHSC_00895	GudB	glutamate dehydrogenase, NAD-specific	Glutamine, glutamate, aspartate, asparagine; ammonia assimilation	9.86	up	9.86	4.401	1.56E-05	3.30	1.45	SACOL0961
SAOUHSC_00222	TarL'	tagB protein	Gram-Positive cell wall components	3.42	up	3.42	1.183	6.28E-06	1.77	0.41	SACOL0238
SAOUHSC_00548		hypothetical protein	Gram-Positive cell wall components	2.42	up	2.42	0.724	1.88E-11	1.28	n.d.	SACOL0612
SAOUHSC_00641	TagH	teichoic acids export protein ATP-binding subunit	Gram-Positive cell wall components	2.76	down	0.36	0.081	4.06E-08	-1.47	n.d.	SACOL0694
SAOUHSC_00642	TagG	teichoic acid biosynthesis protein	Gram-Positive cell wall components	1.99	down	0.50	0.220	4.43E-04	-0.99	n.d.	SACOL0695
SAOUHSC_00728	LtaS	hypothetical protein	Gram-Positive cell wall components	4.66	down	0.21	0.085	2.91E-07	-2.22	n.d.	SACOL0778
SAOUHSC_01871		polysaccharide biosynthesis protein	Gram-Positive cell wall components	1.82	down	0.55	0.117	2.32E-02	-0.87	n.d.	SACOL1804
SAOUHSC_00870	DltB	dltB protein	Gram-Positive cell wall components;Resistance to antibiotics and toxic compounds	2.31	down	0.43	0.153	5.79E-03	-1.21	n.d.	SACOL0936
SAOUHSC_00871	DltC	D-alanine--poly(phosphoribitol) ligase subunit 2	Gram-Positive cell wall components;Resistance to antibiotics and toxic compounds	1.93	down	0.52	0.218	6.25E-03	-0.95	n.d.	SACOL0937
SAOUHSC_01681	PrmA	ribosomal protein L11 methyltransferase	Heat shock;Protein biosynthesis	2.09	down	0.48	0.235	2.82E-02	-1.06	-1.52	SACOL1635
SAOUHSC_01683	DnaK	molecular chaperone DnaK	Heat shock;Protein folding	1.66	down	0.60	0.191	1.95E-08	-0.73	-0.52	SACOL1637
SAOUHSC_02606	HutI	imidazolonepropionase	Histidine Metabolism	13.91	up	13.91	3.034	4.04E-02	3.80	5.99	SACOL2323
SAOUHSC_02607	HutU	urocanate hydratase	Histidine Metabolism	11.00	up	11.00	4.789	8.06E-04	3.46	6.10	SACOL2324
SAOUHSC_01738	HisS	histidyl-tRNA synthetase	Histidine Metabolism;Organic sulfur assimilation;Regulation and Cell signaling - no subcategory	1.82	down	0.55	0.166	3.33E-03	-0.86	-0.92	SACOL1686
SAOUHSC_00020	WalR	two-component response regulator	Iron acquisition and metabolism - no subcategory	2.35	up	2.35	0.665	2.91E-10	1.23	0.42	SACOL0019
SAOUHSC_00130	IsdI	heme-degrading monooxygenase IsdI	Iron acquisition and metabolism - no subcategory	2.76	up	2.76	0.758	1.19E-03	1.46	-0.59	SACOL0152
SAOUHSC_00749	SstD	hypothetical protein	Iron acquisition and metabolism - no subcategory	4.82	down	0.21	0.091	1.88E-11	-2.27	n.d.	SACOL0799
SAOUHSC_01632	GcvPB	glycine dehydrogenase subunit 2	Lipoic acid;Alanine, serine, and glycine	3.64	up	3.64	2.012	2.63E-11	1.86	n.d.	SACOL1593
SAOUHSC_00861	LipA	lipoyl synthase	Lipoic acid;Plant-Prokaryote comparative genomics	3.94	down	0.25	0.097	6.41E-09	-1.98	-1.85	SACOL0927
SAOUHSC_00842	MetN1	ABC transporter ATP-binding protein	Lysine, threonine, methionine, and cysteine	2.22	up	2.22	0.916	2.28E-05	1.15	n.d.	SACOL0882
SAOUHSC_01320	Hom	homoserine dehydrogenase	Lysine, threonine, methionine, and cysteine	2.42	up	2.42	1.055	6.23E-06	1.27	n.d.	SACOL1362
SAOUHSC_01321	ThrC	threonine synthase	Lysine, threonine, methionine, and cysteine	3.14	up	3.14	0.499	4.24E-13	1.65	0.57	SACOL1363
SAOUHSC_01909	MetK	S-adenosylmethionine synthetase	Lysine, threonine, methionine, and cysteine	2.26	down	0.44	0.160	9.80E-16	-1.18	-0.34	SACOL1837
SAOUHSC_00510	CysE	serine acetyltransferase	Lysine, threonine, methionine, and cysteine;Plant-Prokaryote comparative genomics	3.20	up	3.20	0.798	3.81E-03	1.68	0.27	SACOL0575
SAOUHSC_02483	CbiO	cobalt transporter ATP-binding subunit	Membrane Transport - no subcategory;Folate and pterines;NAD and NADP	1.80	down	0.56	0.111	1.73E-04	-0.85	n.d.	SACOL2211
SAOUHSC_02447		hypothetical protein	Miscellaneous - no subcategory	5.09	up	5.09	2.494	3.16E-11	2.35	1.92	SACOL2178
SAOUHSC_01845	Fhs	formate--tetrahydrofolate ligase	Miscellaneous - no subcategory;One-carbon Metabolism;Folate and pterines	8.86	up	8.86	8.058	1.32E-33	3.15	1.69	SACOL1782
SAOUHSC_01018	PurD	phosphoribosylamine--glycine ligase	Miscellaneous - no subcategory;Purines	6.63	up	6.63	4.512	5.54E-14	2.73	2.50	SACOL1083
SAOUHSC_02368	PyrG	CTP synthetase	Miscellaneous - no subcategory;Pyrimidines	1.87	down	0.53	0.266	2.91E-07	-0.91	-0.73	SACOL2119
SAOUHSC_00712		hypothetical protein	Monosaccharides	2.05	up	2.05	1.398	2.99E-07	1.03	0.50	SACOL0763
SAOUHSC_01907		hypothetical protein	Monosaccharides	5.40	up	5.40	2.638	1.88E-11	2.43	1.32	SACOL1835
SAOUHSC_02793		hypothetical protein	Monosaccharides;Cell Wall and Capsule - no subcategory;Capsular and extracellular polysacchrides	2.60	up	2.60	1.069	9.67E-12	1.38	0.77	SACOL2501
SAOUHSC_01901	Tal	putative translaldolase	Monosaccharides;Central carbohydrate metabolism	1.73	up	1.73	0.725	5.20E-05	0.79	0.79	SACOL1831

locus tag	protein symbol	description	theSEED functional category	median fold-change	median fold-change (direction)	median ratio over assays (TSB stat. /exp.)	IQR	p-value BH adjusted	log2 median ratio over assays (TSB stat. /exp.)	log2 ratio Becher *et al.*, 2009	locus tag (COL)
SAOUHSC_02122	LigA	DNA ligase, NAD-dependent	NAD and NADP;DNA repair	1.75	down	0.57	0.164	1.66E-05	-0.80	n.d.	SACOL1965
SAOUHSC_00229	ScdA	cell wall biosynthesis protein ScdA	Nitrogen Metabolism - no subcategory;Stress Response - no subcategory	1.99	up	1.99	0.843	1.52E-04	0.99	n.d.	SACOL0244
SAOUHSC_00743	NrdF	ribonucleotide-diphosphate reductase subunit beta	Nucleosides and Nucleotides - no subcategory	3.07	down	0.33	0.181	2.27E-11	-1.62	n.d.	SACOL0793
SAOUHSC_00742	NrdE	ribonucleotide-diphosphate reductase subunit alpha	Nucleosides and Nucleotides - no subcategory;Oxidative stress	2.30	down	0.43	0.232	1.58E-18	-1.20	-1.37	SACOL0792
SAOUHSC_02828		hypothetical protein	One-carbon Metabolism	2.03	up	2.03	0.547	3.56E-07	1.02	1.18	SACOL2533
SAOUHSC_00552	NagB	hypothetical protein	One-carbon Metabolism;Aminosugars;Capsular and extracellular polysacchrides;Cell Wall and Capsule - no subcategory	1.87	up	1.87	0.493	1.79E-05	0.90	0.60	SACOL0616
SAOUHSC_01007	FolD	bifunctional 5,10-methylene-tetrahydrofolate dehydrogenase/ 5,10-methylene-	One-carbon Metabolism;Folate and pterines	7.63	up	7.63	3.883	7.42E-20	2.93	1.84	SACOL1072
SAOUHSC_01802	CitZ	hypothetical protein	One-carbon Metabolism;Respiration - no subcategory;Central carbohydrate metabolism	5.24	up	5.24	2.981	1.57E-03	2.39	2.95	SACOL1742
SAOUHSC_00367	TcyP	hypothetical protein	Organic sulfur assimilation	5.02	up	5.02	1.557	8.06E-04	2.33	n.d.	SACOL0454
SAOUHSC_00873	NifU	hypothetical protein	Organic sulfur assimilation	2.11	down	0.47	0.149	8.01E-08	-1.08	n.d.	SACOL0939
SAOUHSC_01735		hypothetical protein	Organic sulfur assimilation	2.00	down	0.50	0.118	5.74E-10	-1.00	-1.13	SACOL1683
SAOUHSC_01737	AspS	aspartyl-tRNA synthetase	Organic sulfur assimilation	2.06	down	0.49	0.155	4.56E-16	-1.04	-1.13	SACOL1685
SAOUHSC_02933	BetB	betaine aldehyde dehydrogenase	Osmotic stress	3.06	up	3.06	1.451	4.06E-08	1.61	-1.05	SACOL2628
SAOUHSC_00757	PepT	peptidase T	Oxidative stress	1.56	up	1.56	0.731	4.03E-02	0.64	0.44	SACOL0806
SAOUHSC_01327	KatA	catalase	Oxidative stress	23.09	up	23.09	14.299	4.24E-13	4.53	1.47	SACOL1368
SAOUHSC_02381	Dps	hypothetical protein	Oxidative stress	3.22	up	3.22	2.324	2.46E-09	1.69	0.84	SACOL2131
SAOUHSC_01794	GapB	glyceraldehyde 3-phosphate dehydrogenase 2	Oxidative stress;Central carbohydrate metabolism	5.69	up	5.69	3.547	1.56E-05	2.51	3.31	SACOL1734
SAOUHSC_00204	Hmp	globin domain-containing protein	Oxidative stress;Stress Response - no subcategory	3.38	up	3.38	1.935	7.75E-08	1.76	-0.50	SACOL0220
SAOUHSC_00426	MetQ2	ABC transporter substrate-binding protein	Pathogenicity islands;Lysine, threonine, methionine, and cysteine	3.63	up	3.63	2.181	4.98E-02	1.86	n.d.	SACOL0506
SAOUHSC_01239		hypothetical protein	Periplasmic Stress;Regulation and Cell signaling - no subcategory	2.29	down	0.44	0.110	1.73E-04	-1.19	n.d.	SACOL1281
SAOUHSC_02071		single-strand DNA-binding protein	Phages, Prophages	4.30	up	4.30	1.309	1.56E-05	2.10	n.d.	
SAOUHSC_01193	FakA	hypothetical protein	Phospholipids	1.64	down	0.61	0.283	2.15E-02	-0.71	-0.46	SACOL1240
SAOUHSC_01197	PlsX	putative glycerol-3-phosphate acyltransferase PlsX	Phospholipids	1.76	down	0.57	0.259	3.78E-02	-0.81	-0.78	SACOL1243
SAOUHSC_02363		aldehyde dehydrogenase	Phospholipids;Central carbohydrate metabolism	3.26	up	3.26	1.812	1.09E-19	1.70	1.36	SACOL2114
SAOUHSC_01672	YbeY	hypothetical protein	Phosphorus Metabolism - no subcategory	1.61	up	1.61	0.501	3.91E-02	0.68	0.55	SACOL1627
SAOUHSC_01800	PhoP	alkaline phosphatase synthesis transcriptional regulatory protein	Phosphorus Metabolism - no subcategory	2.79	up	2.79	1.509	8.06E-04	1.48	1.01	SACOL1740
SAOUHSC_00220	TarI'	2-C-methyl-D-erythritol 4-phosphate cytidylyltransferase	Plant-Prokaryote comparative genomics;Gram-Positive cell wall components;Isoprenoids	3.01	up	3.01	1.165	2.15E-02	1.59	0.25	SACOL0236
SAOUHSC_01016	PurN	phosphoribosylglycinamide formyltransferase	Potassium metabolism - no subcategory;Purines	6.90	up	6.90	4.597	5.79E-03	2.79	2.71	SACOL1081
SAOUHSC_00801	SecG	preprotein translocase subunit SecG	Programmed Cell Death and Toxin-antitoxin Systems	1.78	down	0.56	0.261	5.37E-03	-0.83	n.d.	SACOL0844
SAOUHSC_01597	ProC	pyrroline-5-carboxylate reductase	Proline and 4-hydroxyproline	2.35	up	2.35	1.345	9.04E-06	1.24	2.41	SACOL1546
SAOUHSC_02409	RocF	arginase	Proline and 4-hydroxyproline;Arginine; urea cycle, polyamines	7.45	up	7.45	1.518	4.04E-02	2.90	4.65	SACOL2154
SAOUHSC_02869	RocA	1-pyrroline-5-carboxylate dehydrogenase	Proline and 4-hydroxyproline;Arginine; urea cycle, polyamines	8.94	up	8.94	3.664	6.23E-10	3.16	5.36	SACOL2569
SAOUHSC_01695	RsfS	hypothetical protein	Proline and 4-hydroxyproline;NAD and NADP	2.58	down	0.39	0.334	7.28E-06	-1.37	-1.85	SACOL1648
SAOUHSC_00017	RplI	50S ribosomal protein L9	Protein biosynthesis	3.18	down	0.31	0.071	2.91E-07	-1.67	-0.99	SACOL0015
SAOUHSC_00346	YchF	GTP-dependent nucleic acid-binding protein EngD	Protein biosynthesis	1.96	down	0.51	0.131	8.28E-18	-0.97	-0.31	SACOL0435
SAOUHSC_00348	RpsF	30S ribosomal protein S6	Protein biosynthesis	2.57	down	0.39	0.252	3.87E-05	-1.36	n.d.	SACOL0437
SAOUHSC_00463	RnmV	hypothetical protein	Protein biosynthesis	2.33	down	0.43	0.093	5.79E-03	-1.22	n.d.	SACOL0535
SAOUHSC_00464	KsgA	dimethyladenosine transferase	Protein biosynthesis	1.74	down	0.57	0.198	8.59E-03	-0.80	-1.02	SACOL0536
SAOUHSC_00474	RplY	50S ribosomal protein L25/general stress protein Ctc	Protein biosynthesis	2.69	down	0.37	0.112	2.83E-09	-1.43	-1.34	SACOL0545
SAOUHSC_00518	RplK	50S ribosomal protein L11	Protein biosynthesis	3.01	down	0.33	0.156	3.37E-10	-1.59	n.d.	SACOL0583
SAOUHSC_00519	RplA	50S ribosomal protein L1	Protein biosynthesis	2.83	down	0.35	0.178	4.37E-16	-1.50	-3.10	SACOL0584
SAOUHSC_00520	RplJ	50S ribosomal protein L10	Protein biosynthesis	2.27	down	0.44	0.264	2.64E-05	-1.18	n.d.	SACOL0585
SAOUHSC_00521	RplL	50S ribosomal protein L7/L12	Protein biosynthesis	2.35	down	0.43	0.164	3.99E-11	-1.23	-1.63	SACOL0586
SAOUHSC_00767	SaHPF	Staphylococcus aureus hibernation promoting factor	Protein biosynthesis	25.28	up	25.28	24.916	2.18E-10	4.66	0.36	SACOL0815
SAOUHSC_01058	TypA	GTP-binding protein TypA	Protein biosynthesis	2.70	down	0.37	0.161	2.05E-15	-1.43	n.d.	SACOL1118
SAOUHSC_01208	RpsP	30S ribosomal protein S16	Protein biosynthesis	2.19	down	0.46	0.084	3.97E-07	-1.13	-2.71	SACOL1254
SAOUHSC_01211	RplS	50S ribosomal protein L19	Protein biosynthesis	2.95	down	0.34	0.132	3.98E-11	-1.56	-1.80	SACOL1257
SAOUHSC_01232	RpsB	30S ribosomal protein S2	Protein biosynthesis	2.45	down	0.41	0.166	9.44E-18	-1.29	-2.05	SACOL1274
SAOUHSC_01234	Tsf	elongation factor Ts	Protein biosynthesis	1.80	down	0.56	0.236	2.04E-09	-0.85	n.d.	SACOL1276
SAOUHSC_01236	Frr	ribosome recycling factor	Protein biosynthesis	1.94	down	0.52	0.156	7.59E-06	-0.95	-0.43	SACOL1278
SAOUHSC_01493	RpsA	30S ribosomal protein S1	Protein biosynthesis	3.07	up	3.07	0.842	4.61E-41	1.62	0.02	SACOL1516
SAOUHSC_01678	RpsU	30S ribosomal protein S21	Protein biosynthesis	4.03	down	0.25	0.036	2.15E-06	-2.01	-4.80	SACOL1632
SAOUHSC_01689	RpsT	30S ribosomal protein S20	Protein biosynthesis	2.23	down	0.45	0.093	5.40E-09	-1.15	-2.89	SACOL1642
SAOUHSC_01700		GTP-binding protein YqeH	Protein biosynthesis	1.92	down	0.52	0.305	5.21E-05	-0.94	n.d.	SACOL1653
SAOUHSC_01755	RpmA	50S ribosomal protein L27	Protein biosynthesis	3.64	down	0.27	0.160	6.74E-07	-1.86	-2.10	SACOL1700
SAOUHSC_01757	RplU	50S ribosomal protein L21	Protein biosynthesis	2.51	down	0.40	0.140	1.70E-10	-1.33	-2.10	SACOL1702
SAOUHSC_01829	RpsD	30S ribosomal protein S4	Protein biosynthesis	2.47	down	0.40	0.412	1.98E-10	-1.30	n.d.	SACOL1769
SAOUHSC_02361	RpmE2	50S ribosomal protein L31 type B	Protein biosynthesis	3.63	down	0.28	0.175	2.71E-08	-1.86	-2.16	SACOL2112
SAOUHSC_02477	RpsI	30S ribosomal protein S9	Protein biosynthesis	2.39	down	0.42	0.236	1.49E-05	-1.26	n.d.	SACOL2206
SAOUHSC_02478	RplM	50S ribosomal protein L13	Protein biosynthesis	2.87	down	0.35	0.124	3.43E-15	-1.52	n.d.	SACOL2207
SAOUHSC_02484	RplQ	50S ribosomal protein L17	Protein biosynthesis	2.30	down	0.44	0.098	1.56E-08	-1.20	-1.76	SACOL2212
SAOUHSC_02486	RpsK	30S ribosomal protein S11	Protein biosynthesis	2.18	down	0.46	0.188	2.10E-05	-1.12	-2.80	SACOL2214
SAOUHSC_02487	RpsM	30S ribosomal protein S13	Protein biosynthesis	2.60	down	0.38	0.172	1.26E-11	-1.38	-2.10	SACOL2215
SAOUHSC_02492	RplO	50S ribosomal protein L15	Protein biosynthesis	2.62	down	0.38	0.165	3.35E-17	-1.39	n.d.	SACOL2220
SAOUHSC_02493	RpmD	50S ribosomal protein L30	Protein biosynthesis	2.63	down	0.38	0.132	8.97E-09	-1.39	-1.85	SACOL2221
SAOUHSC_02495	RplR	50S ribosomal protein L18	Protein biosynthesis	2.55	down	0.39	0.246	8.52E-03	-1.35	-2.05	SACOL2223
SAOUHSC_02496	RplF	50S ribosomal protein L6	Protein biosynthesis	2.56	down	0.39	0.166	9.17E-17	-1.35	-2.05	SACOL2224
SAOUHSC_02498	RpsH	30S ribosomal protein S8	Protein biosynthesis	2.47	down	0.40	0.135	7.77E-08	-1.30	n.d.	SACOL2225
SAOUHSC_02500	RplE	50S ribosomal protein L5	Protein biosynthesis	2.28	down	0.44	0.329	9.21E-11	-1.19	-2.10	SACOL2227
SAOUHSC_02503	RpsQ	30S ribosomal protein S17	Protein biosynthesis	2.39	down	0.42	0.173	9.17E-04	-1.26	-3.10	SACOL2230
SAOUHSC_02504	RpmC	50S ribosomal protein L29	Protein biosynthesis	2.87	down	0.35	0.090	6.23E-10	-1.52	-2.05	SACOL2231
SAOUHSC_02505	RplP	50S ribosomal protein L16	Protein biosynthesis	2.80	down	0.36	0.255	1.58E-07	-1.49	-1.99	SACOL2232
SAOUHSC_02506	RpsC	30S ribosomal protein S3	Protein biosynthesis	2.38	down	0.42	0.151	1.15E-17	-1.25	-2.48	SACOL2233
SAOUHSC_02507	RplV	50S ribosomal protein L22	Protein biosynthesis	2.73	down	0.37	0.178	7.68E-11	-1.45	-2.28	SACOL2234
SAOUHSC_02508	RpsS	30S ribosomal protein S19	Protein biosynthesis	2.80	down	0.36	0.141	2.62E-02	-1.48	-2.41	SACOL2235
SAOUHSC_02509	RplB	50S ribosomal protein L2	Protein biosynthesis	2.63	down	0.38	0.126	4.41E-17	-1.40	-2.28	SACOL2236
SAOUHSC_02510	RplW	50S ribosomal protein L23	Protein biosynthesis	2.02	down	0.49	0.353	1.96E-02	-1.02	-1.99	SACOL2237
SAOUHSC_02511	RplD	50S ribosomal protein L4	Protein biosynthesis	2.97	down	0.34	0.100	7.91E-13	-1.57	-1.94	SACOL2238
SAOUHSC_02512	RplC	50S ribosomal protein L3	Protein biosynthesis	2.72	down	0.37	0.188	2.05E-14	-1.44	-1.85	SACOL2239
SAOUHSC_03053	TrmE	tRNA modification GTPase TrmE	Protein biosynthesis	1.92	down	0.52	0.131	8.59E-05	-0.94	-0.69	SACOL2738
SAOUHSC_01183	Fmt	methionyl-tRNA formyltransferase	Protein biosynthesis;Folate and pterines	1.63	down	0.61	0.155	2.63E-04	-0.70	0.03	SACOL1228
SAOUHSC_01688	LepA	GTP-binding protein LepA	Protein biosynthesis;Heat shock;Tetrapyrroles	2.14	down	0.47	0.172	5.85E-14	-1.10	-0.85	SACOL1641
SAOUHSC_00529	FusA	elongation factor G	Protein biosynthesis;Virulence - no subcategory	1.72	down	0.58	0.264	3.43E-05	-0.78	-0.92	SACOL0593
SAOUHSC_01626	PepQ2	proline dipeptidase	Protein degradation	2.08	up	2.08	0.720	6.60E-14	1.06	0.54	SACOL1588
SAOUHSC_01778	ClpX	ATP-dependent protease ATP-binding subunit ClpX	Protein degradation	2.16	down	0.46	0.145	2.30E-12	-1.11	-1.10	SACOL1721
SAOUHSC_02255	GroES	co-chaperonin GroES	Protein folding	1.60	down	0.63	0.153	2.34E-02	-0.67	-0.10	SACOL2017
SAOUHSC_01682	DnaJ	chaperone protein DnaJ	Protein folding;Heat shock	2.12	down	0.47	0.176	5.73E-09	-1.08	-0.69	SACOL1636
SAOUHSC_01684	GrpE	heat shock protein GrpE	Protein folding;Heat shock	2.55	down	0.39	0.197	3.81E-16	-1.35	-0.80	SACOL1638
SAOUHSC_00903	SpsB	Signal peptidase IB	Protein processing and modification	1.75	down	0.57	0.188	4.04E-05	-0.81	0.82	SACOL0969
SAOUHSC_02494	RpsE	30S ribosomal protein S5	Protein processing and modification;Protein biosynthesis	2.44	down	0.41	0.189	6.43E-17	-1.29	-3.10	SACOL2222
SAOUHSC_00527	RpsL	30S ribosomal protein S12	Protein processing and modification;Virulence - no subcategory;Protein biosynthesis	2.79	down	0.36	0.180	1.68E-07	-1.48	-2.16	SACOL0591
SAOUHSC_00257	EsxA	hypothetical protein	Protein secretion system, Type VII	6.64	up	6.64	2.614	1.26E-10	2.73	-1.19	SACOL0271
SAOUHSC_00485	Hpt	hypoxanthine phosphoribosyltransferase	Purines	2.63	down	0.38	0.163	2.77E-12	-1.40	-0.94	SACOL0554
SAOUHSC_00539		hypothetical protein	Purines	2.55	down	0.39	0.092	5.98E-09	-1.35	-1.34	SACOL0603
SAOUHSC_01009	PurK	phosphoribosylaminoimidazole carboxylase ATPase subunit	Purines	5.93	up	5.93	5.033	4.24E-13	2.57	2.89	SACOL1074
SAOUHSC_01010	PurC	phosphoribosylaminoimidazole-succinocarboxamide synthase	Purines	6.57	up	6.57	2.482	6.26E-15	2.72	3.62	SACOL1075
SAOUHSC_01011	PurS	phosphoribosylformylglycinamidine synthase, PurS protein	Purines	6.13	up	6.13	4.284	6.23E-10	2.62	3.37	SACOL1076
SAOUHSC_01012	PurQ	phosphoribosylformylglycinamidine synthase I	Purines	6.59	up	6.59	3.451	1.26E-10	2.72	2.56	SACOL1077
SAOUHSC_01013	PurL	phosphoribosylformylglycinamidine synthase II	Purines	6.37	up	6.37	4.629	2.82E-20	2.67	2.66	SACOL1078
SAOUHSC_01015	PurM	phosphoribosylaminoimidazole synthetase	Purines	6.29	up	6.29	1.327	1.88E-11	2.65	2.42	SACOL1080
SAOUHSC_01017	PurH	bifunctional phosphoribosylaminoimidazolecarboxamide formyltransferase/IMP	Purines	6.95	up	6.95	4.634	2.78E-21	2.80	2.24	SACOL1082
SAOUHSC_01176	Gmk	guanylate kinase	Purines	1.76	down	0.57	0.154	2.15E-02	-0.81	-0.43	SACOL1221
SAOUHSC_01330	GuaC	guanosine 5-monophosphate oxidoreductase	Purines	1.67	up	1.67	0.916	1.46E-02	0.74	-1.07	SACOL1371
SAOUHSC_02126	PurB	adenylosuccinate lyase	Purines	1.57	up	1.57	0.778	6.30E-03	0.65	n.d.	SACOL1969
SAOUHSC_02490	Adk	adenylate kinase	Purines	1.80	down	0.56	0.350	3.17E-03	-0.85	-0.92	SACOL2218
SAOUHSC_00019	PurA	adenylosuccinate synthetase	Purines;Histidine Metabolism;Miscellaneous - no subcategory	1.99	up	1.99	0.911	8.53E-14	0.99	n.d.	SACOL0018
SAOUHSC_01485	Ndk	nucleoside diphosphate kinase	Purines;Pyrimidines	4.59	up	4.59	2.291	5.49E-12	2.20	1.95	SACOL1509
SAOUHSC_01743	Apt	adenine phosphoribosyltransferase	Purines;Regulation and Cell signaling - no subcategory	1.87	down	0.54	0.243	7.29E-03	-0.90	-1.02	SACOL1690
SAOUHSC_00500	PdxT	glutamine amidotransferase subunit PdxT	Pyridoxine	1.75	down	0.57	0.169	1.70E-03	-0.81	n.d.	SACOL0565
SAOUHSC_00451	Tmk	thymidylate kinase	Pyrimidines	1.79	down	0.56	0.162	1.94E-06	-0.84	-0.11	SACOL0524

locus tag	protein symbol	description	theSEED functional category	median fold-change	median fold-change (direction)	median ratio over assays (TSB stat. /exp.)	IQR	p-value BH adjusted	log2 median ratio over assays (TSB stat. /exp.)	log2 ratio Becher *et al.*, 2009	locus tag (COL)
**SAOUHSC_01715**	Udk	uridine kinase	Pyrimidines	2.92	down	0.34	0.070	5.49E-12	-1.55	n.d.	**SACOL1666**
**SAOUHSC_02377**	Pdp	pyrimidine-nucleoside phosphorylase	Pyrimidines;Monosaccharides	6.83	up	6.83	3.535	5.54E-14	2.77	3.14	**SACOL2128**
**SAOUHSC_03001**	IcaR	ica operon transcriptional regulator IcaR	Quorum sensing and biofilm formation	1.86	up	1.86	0.485	5.12E-04	0.89	0.74	**SACOL2688**
**SAOUHSC_01361**	MsrR	transcriptional regulator	Regulation and Cell signaling - no subcategory	1.65	down	0.61	0.194	8.32E-03	-0.72	n.d.	**SACOL1398**
**SAOUHSC_00201**	Opp-5A	hypothetical protein	Regulation and Cell signaling - no subcategory;ABC transporters	3.02	up	3.02	1.781	5.27E-03	1.59	n.d.	**SACOL0217**
**SAOUHSC_01979**		hypothetical protein	Resistance to antibiotics and toxic compounds	32.88	down	0.03	0.005	4.06E-08	-5.04	-5.06	**SACOL1904**
**SAOUHSC_02630**		hypothetical protein	Resistance to antibiotics and toxic compounds	2.23	down	0.45	0.119	1.64E-02	-1.16	n.d.	**SACOL2348**
**SAOUHSC_01467**	Pbp2	penicillin-binding protein 2	Resistance to antibiotics and toxic compounds;Cell Division and Cell Cycle - no subcategory;Cell Wall and Capsule - no subcategory	1.72	down	0.58	0.158	6.19E-16	-0.78	0.35	**SACOL1490**
**SAOUHSC_00646**	Pbp4	penicillin-binding protein 4	Resistance to antibiotics and toxic compounds;Cell Wall and Capsule - no subcategory	3.98	down	0.25	0.142	4.41E-04	-1.99	n.d.	**SACOL0699**
**SAOUHSC_02873**	CopA	cation transporter E1-E2 family ATPase	Resistance to antibiotics and toxic compounds;Electron accepting reactions	1.71	up	1.71	0.679	4.99E-05	0.77	n.d.	**SACOL2572**
**SAOUHSC_01644**		hypothetical protein	Resistance to antibiotics and toxic compounds;Potassium metabolism - no subcategory;Oxidative stress;Central carbohydrate metabolism	1.89	down	0.53	0.089	3.43E-10	-0.92	n.d.	**SACOL1602**
**SAOUHSC_02581**		hypothetical protein	Respiration - no subcategory	4.65	up	4.65	1.768	5.79E-03	2.22	n.d.	**SACOL2300**
**SAOUHSC_02582**	FdhA	formate dehydrogenase subunit alpha	Respiration - no subcategory	8.42	up	8.42	5.782	4.24E-13	3.07	1.62	**SACOL2301**
**SAOUHSC_01347**	CitB	aconitate hydratase	Respiration - no subcategory;One-carbon Metabolism;Central carbohydrate metabolism	5.18	up	5.18	2.803	5.64E-48	2.37	1.39	**SACOL1385**
**SAOUHSC_01886**	RibH	6,7-dimethyl-8-ribityllumazine synthase	Riboflavin, FMN, FAD	8.08	up	8.08	7.291	5.98E-09	3.01	-0.33	**SACOL1817**
**SAOUHSC_01889**	RibD	riboflavin biosynthesis protein RibD	Riboflavin, FMN, FAD	3.64	up	3.64	3.166	4.33E-04	1.86	-0.28	**SACOL1820**
**SAOUHSC_00739**	QueF	7-cyano-7-deazaguanine reductase	RNA processing and modification	2.04	down	0.49	0.103	4.06E-08	-1.03	-1.07	**SACOL0789**
**SAOUHSC_00951**		hypothetical protein	RNA processing and modification	10.38	up	10.38	5.719	6.23E-10	3.38	2.60	**SACOL1020**
**SAOUHSC_01095**	RnhC	ribonuclease HIII	RNA processing and modification	2.77	down	0.36	0.133	1.10E-02	-1.47	n.d.	**SACOL1150**
**SAOUHSC_01659**		hypothetical protein	RNA processing and modification	1.84	down	0.54	0.190	6.76E-04	-0.88	-0.46	**SACOL1615**
**SAOUHSC_01749**	QueA	S-adenosylmethionine:tRNA ribosyltransferase-isomerase	RNA processing and modification	1.64	down	0.61	0.148	4.87E-02	-0.71	-1.52	**SACOL1695**
**SAOUHSC_02097**		hypothetical protein	RNA processing and modification	2.34	up	2.34	1.116	1.23E-08	1.23	n.d.	**SACOL1941**
**SAOUHSC_02316**	CshA	DEAD-box ATP dependent DNA helicase	RNA processing and modification	2.82	down	0.36	0.173	1.82E-29	-1.49	-2.22	**SACOL2072**
**SAOUHSC_02279**		hypothetical protein	RNA processing and modification;Cell Division and Cell Cycle - no subcategory;Cell Wall and Capsule - no subcategory;Protein biosynthesis	1.61	down	0.62	0.109	1.34E-02	-0.68	n.d.	**SACOL2040**
**SAOUHSC_01269**	MiaB	(dimethylallyl)adenosine tRNA methylthiotransferase	RNA processing and modification;Protein processing and modification	2.06	down	0.48	0.181	5.83E-04	-1.04	-2.99	**SACOL1312**
**SAOUHSC_01055**		inositol monophosphatase family protein	Stress Response - no subcategory	2.05	up	2.05	0.902	8.77E-05	1.04	n.d.	**SACOL1116**
**SAOUHSC_01413**		hypothetical protein	Stress Response - no subcategory	1.73	down	0.58	0.199	3.97E-05	-0.79	n.d.	**SACOL1445**
**SAOUHSC_01814**	UspA2	hypothetical protein	Stress Response - no subcategory	2.08	up	2.08	0.644	2.33E-11	1.06	0.82	**SACOL1753**
**SAOUHSC_01819**	UspA1	hypothetical protein	Stress Response - no subcategory	15.59	up	15.59	6.018	1.87E-09	3.96	1.66	**SACOL1759**
**SAOUHSC_02402**	MtlA	PTS system mannitol-specific transporter subunit IIA	Sugar alcohols	3.30	up	3.30	3.647	7.00E-08	1.72	1.76	**SACOL2148**
**SAOUHSC_02403**	MtlD	mannitol-1-phosphate 5-dehydrogenase	Sugar alcohols	3.42	up	3.42	1.709	2.13E-11	1.77	1.71	**SACOL2149**
**SAOUHSC_01276**	GlpK	glycerol kinase	Sugar alcohols;Phospholipids;Central carbohydrate metabolism	1.90	down	0.53	0.161	1.15E-12	-0.93	2.43	**SACOL1320**
**SAOUHSC_00364**	AhpF	alkyl hydroperoxide reductase subunit F	Sulfur Metabolism - no subcategory	1.68	up	1.68	0.532	7.75E-08	0.75	0.76	**SACOL0451**
**SAOUHSC_00365**	AhpC	alkyl hydroperoxide reductase subunit C	Sulfur Metabolism - no subcategory	2.40	up	2.40	1.339	1.70E-18	1.27	0.96	**SACOL0452**
**SAOUHSC_01999**		bacterioferritin comigratory protein	Sulfur Metabolism - no subcategory	2.60	up	2.60	0.477	2.49E-09	1.38	0.81	**SACOL1921**
**SAOUHSC_01499**		hypothetical protein	Sulfur Metabolism - no subcategory;Pyrimidines	1.98	down	0.51	0.239	1.59E-03	-0.98	-0.48	**SACOL1520**
**SAOUHSC_00573**	HemQ	putative heme peroxidase	Tetrapyrroles	2.15	up	2.15	0.910	4.06E-08	1.10	n.d.	**SACOL0633**
**SAOUHSC_02000**	GsaB	glutamate-1-semialdehyde aminotransferase	Tetrapyrroles	2.27	up	2.27	1.690	3.58E-16	1.18	-0.23	**SACOL1922**
**SAOUHSC_01245**		hypothetical protein	Transcription	1.73	down	0.58	0.102	4.25E-05	-0.79	-0.92	**SACOL1287**
**SAOUHSC_01714**	GreA	transcription elongation factor GreA	Transcription	1.78	down	0.56	0.267	8.14E-03	-0.83	-0.92	**SACOL1665**
**SAOUHSC_02362**	Rho	transcription termination factor Rho	Transcription	1.63	down	0.61	0.206	5.57E-03	-0.71	-1.02	**SACOL2113**
**SAOUHSC_02369**	RpoE	DNA-directed RNA polymerase subunit delta	Transcription	2.22	down	0.45	0.219	9.28E-10	-1.15	-0.92	**SACOL2120**
**SAOUHSC_02485**	RpoA	DNA-directed RNA polymerase subunit alpha	Transcription	1.95	down	0.51	0.301	1.66E-10	-0.97	-1.07	**SACOL2213**
**SAOUHSC_00524**	RpoB	DNA-directed RNA polymerase subunit beta	Transcription;Virulence - no subcategory	1.85	down	0.54	0.249	2.68E-18	-0.89	-0.92	**SACOL0588**
**SAOUHSC_01279**		hydrolase alpha/beta fold domain-containing protein	Triacylglycerols	2.22	up	2.22	0.415	1.12E-04	1.15	n.d.	**SACOL1322**
**SAOUHSC_00886**	MnhD	putative monovalent cation/H+ antiporter subunit D	Uni- Sym- and Antiporters	1.79	down	0.56	0.129	2.50E-03	-0.84	n.d.	**SACOL0952**
**SAOUHSC_00889**	MnhA	monovalent cation/H+ antiporter subunit A	Uni- Sym- and Antiporters	2.14	down	0.47	0.080	2.15E-06	-1.10	n.d.	**SACOL0955**
**SAOUHSC_00528**	RpsG	30S ribosomal protein S7	Virulence - no subcategory;Protein biosynthesis	2.55	down	0.39	0.150	3.18E-10	-1.35	-2.28	**SACOL0592**
**SAOUHSC_01784**	RplT	50S ribosomal protein L20	Virulence - no subcategory;Protein biosynthesis	2.55	down	0.39	0.318	4.15E-02	-1.35	-1.94	**SACOL1725**
**SAOUHSC_01785**	RpmI	50S ribosomal protein L35	Virulence - no subcategory;Protein biosynthesis	4.81	down	0.21	0.120	5.79E-03	-2.27	-1.76	**SACOL1726**
**SAOUHSC_00525**	RpoC	DNA-directed RNA polymerase subunit beta	Virulence - no subcategory;Transcription	1.62	down	0.62	0.275	7.33E-03	-0.70	-0.85	**SACOL0589**
**SAOUHSC_00039**		hypothetical protein		3.59	down	0.28	0.092	4.56E-03	-1.84	-1.67	**SACOL0067**
**SAOUHSC_00044**		hypothetical protein		2.00	up	2.00	1.873	3.41E-02	1.00	0.85	**SACOL0072**
**SAOUHSC_00135**		hypothetical protein		2.25	up	2.25	1.263	6.23E-09	1.17	1.94	**SACOL0157**
**SAOUHSC_00182**		hypothetical protein		4.11	down	0.24	0.176	8.06E-04	-2.04	n.d.	**SACOL0199**
**SAOUHSC_00197**	FadD	hypothetical protein		26.05	up	26.05	10.794	2.91E-07	4.70	n.d.	**SACOL0213**
**SAOUHSC_00200**	PrsS	hypothetical protein		1.96	down	0.51	0.173	5.37E-03	-0.97	n.d.	**SACOL0216**
**SAOUHSC_00284**		5-nucleotidase		8.55	up	8.55	3.739	8.06E-04	3.10	n.d.	**SACOL0303**
**SAOUHSC_00290**	PfoR	hypothetical protein		5.34	down	0.19	0.126	5.79E-03	-2.42	n.d.	**SACOL0307**
**SAOUHSC_00301**		hypothetical protein		1.79	down	0.56	0.226	2.36E-02	-0.84	n.d.	**SACOL0391**
**SAOUHSC_00309**		hypothetical protein		4.29	up	4.29	2.198	8.96E-10	2.10	0.08	**SACOL0399**
**SAOUHSC_00330**		hypothetical protein		1.89	down	0.53	0.193	4.98E-02	-0.92	n.d.	**SACOL0419**
**SAOUHSC_00356**		hypothetical protein		3.93	up	3.93	2.933	4.06E-08	1.97	2.55	**SACOL0444**
**SAOUHSC_00366**	NfrA	NAD(P)H-flavin oxidoreductase		2.07	up	2.07	0.588	8.39E-03	1.05	0.51	**SACOL0453**
**SAOUHSC_00371**		hypothetical protein		15.05	up	15.05	10.725	1.15E-12	3.91	2.30	**SACOL0457**
**SAOUHSC_00405**		hypothetical protein		2.59	up	2.59	1.190	8.06E-04	1.37	1.14	**SACOL0486**
**SAOUHSC_00486**	FtsH	hypothetical protein		1.87	down	0.54	0.311	8.92E-09	-0.90	0.70	**SACOL0555**
**SAOUHSC_00502**	CtsR	hypothetical protein		1.95	up	1.95	1.312	4.93E-02	0.96	n.d.	**SACOL0567**
**SAOUHSC_00533**	HchA	chaperone protein HchA		1.89	up	1.89	1.168	2.26E-05	0.91	0.62	**SACOL0597**
**SAOUHSC_00538**		haloacid dehalogenase-like hydrolase		2.36	up	2.36	0.440	1.31E-09	1.24	n.d.	**SACOL0602**
**SAOUHSC_00603**		hypothetical protein		1.70	up	1.70	1.005	1.78E-04	0.77	1.21	**SACOL0655**
**SAOUHSC_00607**		hypothetical protein		2.33	down	0.43	0.097	8.06E-04	-1.22	-0.47	**SACOL0659**
**SAOUHSC_00634**	MntC	ABC transporter substrate-binding protein		3.13	up	3.13	0.951	7.59E-14	1.64	n.d.	**SACOL0688**
**SAOUHSC_00637**	MntA	hypothetical protein		2.25	up	2.25	0.862	9.10E-06	1.17	n.d.	**SACOL0690**
**SAOUHSC_00647**	AbcA	hypothetical protein		4.02	up	4.02	2.140	1.87E-12	2.01	n.d.	**SACOL0700**
**SAOUHSC_00652**	FhuA	iron compound ABC transporter ATP-binding protein		5.36	down	0.19	0.160	7.00E-08	-2.42	-2.71	**SACOL0704**
**SAOUHSC_00663**		hypothetical protein		1.93	up	1.93	0.533	2.01E-07	0.95	0.43	**SACOL0714**
**SAOUHSC_00686**		hypothetical protein		2.11	up	2.11	0.755	1.36E-08	1.08	-0.33	**SACOL0738**
**SAOUHSC_00788**		hypothetical protein		4.01	up	4.01	1.752	8.06E-04	2.00	0.79	**SACOL0831**
**SAOUHSC_00792**		hypothetical protein		3.40	up	3.40	1.308	1.26E-10	1.77	1.17	**SACOL0834**
**SAOUHSC_00838**		hypothetical protein		2.54	up	2.54	1.429	1.66E-09	1.34	0.57	**SACOL0879**
**SAOUHSC_00862**		hypothetical protein		2.03	down	0.49	0.305	5.20E-03	-1.02	-1.07	**SACOL0928**
**SAOUHSC_00934**	SpxA	transcriptional regulator Spx		4.06	up	4.06	4.437	1.19E-12	2.02	0.48	**SACOL1002**
**SAOUHSC_00964**		hypothetical protein		2.15	down	0.46	0.213	7.33E-04	-1.11	n.d.	**SACOL1035**
**SAOUHSC_01027**		hypothetical protein		4.80	up	4.80	3.252	8.55E-10	2.26	-0.73	**SACOL1090**
**SAOUHSC_01039**		hypothetical protein		1.62	down	0.62	0.209	3.35E-02	-0.70	n.d.	**SACOL1101**
**SAOUHSC_01138**		hypothetical protein		9.25	up	9.25	3.487	1.56E-05	3.21	n.d.	**SACOL1189**
**SAOUHSC_01154**	SepF	hypothetical protein		2.78	down	0.36	0.255	2.29E-05	-1.47	1.23	**SACOL1202**
**SAOUHSC_01184**	Sun	sun protein		2.55	down	0.39	0.120	1.16E-08	-1.35	-0.97	**SACOL1229**
**SAOUHSC_01185**		ribosomal RNA large subunit methyltransferase N		7.06	down	0.14	0.029	8.06E-04	-2.82	n.d.	**SACOL1230**
**SAOUHSC_01186**	Stp1	hypothetical protein		1.64	down	0.61	0.125	7.35E-03	-0.72	-0.28	**SACOL1231**
**SAOUHSC_01187**	PknB	hypothetical protein		1.64	down	0.61	0.194	7.92E-04	-0.71	n.d.	**SACOL1232**
**SAOUHSC_01222**	TopA	DNA topoisomerase I		2.67	down	0.38	0.154	1.15E-12	-1.41	-0.53	**SACOL1267**
**SAOUHSC_01223**	Gid	tRNA (uracil-5-)-methyltransferase Gid		2.44	down	0.41	0.129	1.53E-24	-1.29	n.d.	**SACOL1268**
**SAOUHSC_01235**	PyrH	uridylate kinase		1.71	down	0.59	0.183	1.07E-05	-0.77	n.d.	**SACOL1277**
**SAOUHSC_01256**		hypothetical protein		1.78	down	0.56	0.240	3.57E-02	-0.83	-1.28	**SACOL1298**
**SAOUHSC_01264**		hypothetical protein		3.55	up	3.55	4.256	1.17E-04	1.83	n.d.	**SACOL1306**
**SAOUHSC_01284**		hypothetical protein		2.40	down	0.42	0.173	7.22E-11	-1.26	n.d.	**SACOL1327**
**SAOUHSC_01317**		hypothetical protein		1.62	up	1.62	0.549	2.06E-02	0.69	n.d.	**SACOL1358**
**SAOUHSC_01336**		hypothetical protein		2.02	down	0.49	0.165	1.09E-03	-1.02	n.d.	**SACOL1376**
**SAOUHSC_01349**		hypothetical protein		2.18	up	2.18	0.546	2.98E-07	1.12	0.93	**SACOL1387**
**SAOUHSC_01392**		ABC transporter ATP-binding protein		1.94	down	0.52	0.204	1.81E-12	-0.96	-0.82	**SACOL1427**
**SAOUHSC_01408**		hypothetical protein		1.77	up	1.77	0.560	5.29E-05	0.82	-0.43	**SACOL1441**
**SAOUHSC_01415**		hypothetical protein		2.44	up	2.44	1.761	3.53E-08	1.29	n.d.	**SACOL1447**
**SAOUHSC_01420**	ArlR	DNA-binding response regulator		2.16	up	2.16	0.506	7.15E-03	1.11	n.d.	**SACOL1451**
**SAOUHSC_01425**		hypothetical protein		1.82	down	0.55	0.221	4.06E-02	-0.86	n.d.	**SACOL1454**
**SAOUHSC_01433**	FakB2	hypothetical protein		1.99	up	1.99	0.986	2.68E-08	0.99	0.79	**SACOL1460**
**SAOUHSC_01476**		hypothetical protein		2.68	down	0.37	0.205	1.56E-05	-1.42	-1.41	**SACOL1499**
**SAOUHSC_01575**		helix-turn-helix domain-containing protein		1.82	up	1.82	0.434	3.18E-07	0.87	n.d.	
**SAOUHSC_01594**		hypothetical protein		1.96	up	1.96	0.385	1.56E-05	0.97	0.82	**SACOL1543**

locus tag	protein symbol	description	theSEED functional category	median fold-change	median fold-change (direction)	median ratio over assays (TSB stat. /exp.)	IQR	p-value BH adjusted	log2 median ratio over assays (TSB stat. /exp.)	log2 ratio Becher *et al.*, 2009	locus tag (COL)
SAOUHSC_01606		peptidase T		3.12	up	3.12	1.017	5.39E-11	1.64	0.93	SACOL1555
SAOUHSC_01661		hypothetical protein		3.54	down	0.28	0.100	4.06E-08	-1.82	-1.55	SACOL1617
SAOUHSC_01664		hypothetical protein		1.86	up	1.86	0.842	1.58E-06	0.89	n.d.	SACOL1620
SAOUHSC_01675		hypothetical protein		1.80	up	1.80	0.558	1.42E-04	0.84	n.d.	SACOL1629
SAOUHSC_01676		hypothetical protein		1.65	up	1.65	0.550	2.82E-09	0.73	0.11	SACOL1630
SAOUHSC_01718		hypothetical protein		2.06	down	0.49	0.070	4.04E-02	-1.04	n.d.	SACOL1669
SAOUHSC_01721		hypothetical protein		1.64	up	1.64	0.161	9.68E-03	0.72	-0.09	SACOL1672
SAOUHSC_01729		hypothetical protein		6.99	up	6.99	4.362	5.98E-09	2.80	n.d.	SACOL1679
SAOUHSC_01730	CsbD	hypothetical protein		2.10	up	2.10	0.805	1.49E-08	1.07	n.d.	SACOL1680
SAOUHSC_01779	Tig	trigger factor		1.95	down	0.51	0.150	1.55E-18	-0.96	-0.53	SACOL1722
SAOUHSC_01786	InfC	translation initiation factor IF-3		2.80	down	0.36	0.169	1.88E-11	-1.48	-2.05	SACOL1727
SAOUHSC_01816	PepQ	hypothetical protein		1.87	up	1.87	0.800	5.10E-03	0.90	0.94	SACOL1756
SAOUHSC_01839	TyrS	tyrosyl-tRNA synthetase		1.71	down	0.58	0.222	5.21E-05	-0.78	-0.61	SACOL1778
SAOUHSC_01854		hypothetical protein		3.13	up	3.13	1.426	9.37E-34	1.65	0.89	SACOL1788
SAOUHSC_01855		hypothetical protein		2.78	up	2.78	1.094	9.74E-17	1.48	0.21	SACOL1789
SAOUHSC_01860		hypothetical protein		1.72	up	1.72	0.360	1.43E-05	0.78	0.32	SACOL1794
SAOUHSC_01868		dipeptidase PepV		2.15	up	2.15	1.244	1.91E-22	1.11	-0.02	SACOL1801
SAOUHSC_01869		hypothetical protein		5.89	up	5.89	2.310	1.88E-14	2.56	0.12	SACOL1802
SAOUHSC_01879	Rot	virulence factor regulator protein		3.22	down	0.31	0.368	3.78E-03	-1.69	n.d.	SACOL1812
SAOUHSC_01968	Hit	hypothetical protein		1.88	up	1.88	0.542	9.75E-07	0.91	n.d.	SACOL1894
SAOUHSC_01969		hypothetical protein		2.40	up	2.40	0.971	7.37E-11	1.26	n.d.	SACOL1895
SAOUHSC_01978		hypothetical protein		1.86	down	0.54	0.083	5.79E-03	-0.89	n.d.	SACOL1903
SAOUHSC_01987		hypothetical protein		2.59	up	2.59	1.938	7.59E-14	1.37	1.18	SACOL1912
SAOUHSC_01998		hypothetical protein		2.32	up	2.32	0.940	2.64E-05	1.21	n.d.	SACOL1920
SAOUHSC_02013		hypothetical protein		7.37	up	7.37	5.024	9.67E-12	2.88	2.04	SACOL1933
SAOUHSC_02106		hypothetical protein		2.33	down	0.43	0.266	6.17E-04	-1.22	0.03	SACOL1950
SAOUHSC_02107		UDP-N-acetylmuramyl tripeptide synthetase		2.31	down	0.43	0.195	3.28E-15	-1.21	-0.36	SACOL1951
SAOUHSC_02145		hypothetical protein		4.57	up	4.57	2.280	2.91E-07	2.19	n.d.	SACOL1987
SAOUHSC_02150		hypothetical protein		3.32	up	3.32	1.523	4.24E-13	1.73	1.77	SACOL1992
SAOUHSC_02152	PmtC	ABC transporter ATP-binding protein		4.55	up	4.55	2.971	1.56E-10	2.19	n.d.	SACOL1994
SAOUHSC_02154	PmtA	ABC transporter ATP-binding protein		2.82	up	2.82	3.090	1.69E-02	1.50	n.d.	SACOL1996
SAOUHSC_02218		hypothetical protein		3.11	up	3.11	1.365	5.65E-07	1.64	n.d.	
SAOUHSC_02387		hypothetical protein		5.84	up	5.84	2.904	2.38E-10	2.55	1.42	SACOL2136
SAOUHSC_02425		hypothetical protein		18.01	up	18.01	66.272	5.79E-03	4.17	3.57	SACOL2163
SAOUHSC_02441	Asp23	alkaline shock protein 23		8.41	up	8.41	8.100	1.70E-18	3.07	1.68	SACOL2173
SAOUHSC_02442		hypothetical protein		20.98	up	20.98	11.256	1.12E-04	4.39	n.d.	SACOL2174
SAOUHSC_02443	AmaP	hypothetical protein		13.86	up	13.86	11.184	4.06E-08	3.79	2.44	SACOL2175
SAOUHSC_02445		hypothetical protein		2.13	up	2.13	0.529	1.16E-06	1.09	1.12	SACOL2177
SAOUHSC_02519		hypothetical protein		2.34	up	2.34	0.653	4.06E-08	1.23	1.71	SACOL2245
SAOUHSC_02554	FhuD2	hypothetical protein		4.01	down	0.25	0.064	1.51E-11	-2.00	-1.13	SACOL2277
SAOUHSC_02568		hypothetical protein		2.03	up	2.03	1.188	1.30E-03	1.02	0.70	SACOL2288
SAOUHSC_02574		hypothetical protein		1.95	down	0.51	0.409	9.87E-03	-0.96	0.81	SACOL2293
SAOUHSC_02585		hypothetical protein		2.04	up	2.04	1.738	1.57E-03	1.03	n.d.	SACOL2304
SAOUHSC_02604		hypothetical protein		17.04	up	17.04	11.282	1.26E-10	4.09	1.43	SACOL2321
SAOUHSC_02611	LyrA	hypothetical protein		1.84	down	0.54	0.103	8.06E-04	-0.88	n.d.	SACOL2328
SAOUHSC_02650		hypothetical protein		9.33	up	9.33	5.188	2.91E-07	3.22	n.d.	SACOL2365
SAOUHSC_02665		hypothetical protein		7.28	up	7.28	4.355	3.14E-09	2.86	1.63	SACOL2379
SAOUHSC_02670		hypothetical protein		2.41	up	2.41	0.393	1.12E-04	1.27	1.30	SACOL2385
SAOUHSC_02690		hypothetical protein		2.00	down	0.50	0.150	1.56E-06	-1.00	n.d.	SACOL2403
SAOUHSC_02724		hypothetical protein		3.05	up	3.05	1.292	1.28E-11	1.61	n.d.	SACOL2436
SAOUHSC_02731		hypothetical protein		1.84	down	0.54	0.210	7.32E-03	-0.88	n.d.	SACOL2442
SAOUHSC_02737		epimerase/dehydratase		1.97	down	0.51	0.201	1.14E-02	-0.98	-0.28	SACOL2446
SAOUHSC_02774		hypothetical protein		7.68	up	7.68	5.771	7.75E-08	2.94	n.d.	SACOL2484
SAOUHSC_02778		hypothetical protein		3.95	up	3.95	1.572	5.98E-09	1.98	1.41	SACOL2488
SAOUHSC_02820		hypothetical protein		2.26	up	2.26	1.608	9.45E-03	1.17	n.d.	SACOL2525
SAOUHSC_02827		hypothetical protein		2.76	up	2.76	1.529	6.45E-04	1.46	1.32	SACOL2532
SAOUHSC_02829	Frp	NAD(P)H-flavin oxidoreductase		2.84	up	2.84	0.912	1.47E-13	1.50	n.d.	SACOL2534
SAOUHSC_02844		hypothetical protein		1.96	up	1.96	0.644	7.75E-08	0.97	n.d.	SACOL2549
SAOUHSC_02862	ClpL	ATP-dependent Clp protease, ATP-binding subunit ClpC		16.08	up	16.08	13.824	1.56E-05	4.01	2.99	SACOL2563
SAOUHSC_02866		hypothetical protein		2.70	down	0.37	0.134	1.92E-08	-1.43	n.d.	SACOL2566
SAOUHSC_02887	IsaA	immunodominant antigen A		2.44	down	0.41	0.275	3.20E-06	-1.29	-2.05	SACOL2584
SAOUHSC_02899		hypothetical protein		6.41	up	6.41	3.288	4.06E-08	2.68	1.42	SACOL2596
SAOUHSC_02900		hypothetical protein		7.57	up	7.57	6.903	4.24E-13	2.92	1.70	SACOL2597
SAOUHSC_02929		acetyl-CoA synthetase		1.78	up	1.78	0.814	7.75E-08	0.83	0.48	SACOL2624
SAOUHSC_02972	IsaB	immunodominant antigen B		17.74	up	17.74	4.885	1.12E-04	4.15	n.d.	SACOL2660
SAOUHSC_02978		phage infection protein		1.78	down	0.56	0.185	3.84E-06	-0.83	n.d.	SACOL2665
SAOUHSC_02979		N-acetylmuramoyl-L-alanine amidase		3.10	up	3.10	1.197	5.79E-03	1.63	n.d.	SACOL2666
SAOUHSC_02980		hypothetical protein		2.07	up	2.07	0.863	1.14E-06	1.05	0.18	SACOL2667
SAOUHSC_03022		hypothetical protein		3.56	up	3.56	1.883	5.85E-14	1.83	2.21	SACOL2711
SAOUHSC_03052	GidA	tRNA uridine 5-carboxymethylaminomethyl modification enzyme GidA		2.23	down	0.45	0.174	4.25E-09	-1.16	-1.37	SACOL2737

log_2-ratios

-2 0 2

4

supplemental table S4: significant regulated proteins in comparison of non-adherent and S9 infection with S. aureus (cell culture OD=0.4, 8h p.i., 24h p.i. and 32h p.i.)

locus tag	protein symbol	description	theSEED functional category	log2 ratio OD=0.4	log2 ratio 8h p.i.	log2 ratio 24h p.i.	log2 ratio 32h p.i.	median over 8,24 and 32h ratio; median tendence - 1.5fc cutoff	median fold-change OD=0.4	median fold-change (direction) OD=0.4	median ratio over assays against non-ad. OD=0.4	p-value BH adjusted OD=0.4	median fold-change 8h p.i.	median fold-change (direction) 8h p.i.	median ratio over assays against non-ad. 8h p.i.	p-value BH adjusted 8h p.i.	median fold-change 24h p.i.	median fold-change (direction) 24h p.i.	median ratio over assays against non-ad. 24h p.i.	p-value BH adjusted 24h p.i.	median fold-change 32h p.i.	median fold-change (direction) 32h p.i.	median ratio over assays against non-ad. 32h p.i.	p-value BH adjusted 32h p.i.
SAOUHSC_00924	Opp-3C	hypothetical protein	ABC transporters	0.41	1.53	1.81	1.74	▲	1.33	up	1.33	1.000E+00	2.893	up	2.893	3.67E-08	3.502	up	3.502	7.23E-08	3.337	up	3.337	4.58E-08
SAOUHSC_00925	Opp-3D	hypothetical protein	ABC transporters	0.08	0.75	1.46	1.39	▲	1.06	up	1.06	1.000E+00	1.677	up	1.677	7.49E-03	2.743	up	2.743	6.48E-10	2.627	up	2.627	4.87E-09
SAOUHSC_00926	Opp-3F	oligopeptide ABC transporter ATP-binding protein	ABC transporters	0.36	0.73	0.95	1.03	▲	1.28	up	1.28	1.000E+00	1.654	up	1.654	1.40E-02	1.937	up	1.937	4.57E-05	2.044	up	2.044	6.15E-07
SAOUHSC_02767	Opp-1A	peptide ABC transporter peptide-binding protein	ABC transporters;Motility and Chemotaxis - no subcategory	-2.16	0.19	-0.05	0.22	–	4.46	down	0.22	4.155E-05	1.143	up	1.143	1.00E+00	1.036	down	0.965	1.00E+00	1.162	up	1.162	1.00E+00
SAOUHSC_00927	Opp-3A	oligopeptide ABC transporter substrate-binding protein	ABC transporters;Regulation and Cell signaling - no subcategory	0.35	0.33	1.08	1.54	▲	1.28	up	1.28	1.000E+00	1.256	up	1.256	1.00E+00	2.114	up	2.114	1.08E-04	2.910	up	2.910	1.61E-08
SAOUHSC_00069	Spa	protein A	Adhesion	0.56	-3.90	-0.79	-2.21	▼	1.47	up	1.47	2.898E-01	14.941	down	0.067	1.00E-12	1.729	down	0.579	9.43E-01	4.628	down	0.216	4.30E-03
SAOUHSC_00487	HslO	Hsp33-like chaperonin	Adhesion	0.23	-0.15	-0.97	-0.85	▼	1.17	up	1.17	1.000E+00	1.112	down	0.899	1.00E+00	1.966	down	0.509	2.17E-03	1.802	down	0.555	1.96E-03
SAOUHSC_00544	SdrC	sdrC protein	Adhesion	-1.07	-0.93	n.d.	n.d.	▼	2.10	down	0.48	1.309E-02	1.901	down	0.526	2.70E-02								
SAOUHSC_02963	ClfB	clumping factor B	Adhesion	-1.25	-1.78	-3.44	-3.74	▼	2.38	down	0.42	2.410E-17	3.433	down	0.291	4.65E-26	10.882	down	0.092	6.27E-08	13.400	down	0.075	9.40E-06
SAOUHSC_01634	GcvT	glycine cleavage system aminomethyltransferase T	Alanine, serine, and glycine	-0.49	0.27	0.60	0.81	▲	1.40	down	0.71	1.000E+00	1.208	up	1.208	1.00E+00	1.513	up	1.513	4.01E-02	1.748	up	1.748	1.72E-06
SAOUHSC_02305	Alr	alanine racemase	Alanine, serine, and glycine;Central carbohydrate metabolism;RNA processing and modification	-0.16	-0.32	-1.20	-0.89	▼	1.12	down	0.89	1.000E+00	1.252	down	0.798	1.00E+00	2.303	down	0.434	5.47E-04	1.857	down	0.539	2.26E-01
SAOUHSC_00308		hypothetical protein	Alanine, serine, and glycine;Lipoic acid;Plant-Prokaryote comparative genomics;Central carbohydrate metabolism	-0.27	1.50	1.32	1.51	▲	1.21	down	0.83	1.000E+00	2.819	up	2.819	1.02E-04	2.501	up	2.501	8.57E-01	2.843	up	2.843	8.01E-01
SAOUHSC_00532	Kbl	2-amino-3-ketobutyrate coenzyme A ligase	Alanine, serine, and glycine;Lysine, threonine, methionine, and cysteine	0.07	0.42	0.39	0.79	–	1.05	up	1.05	1.000E+00	1.341	up	1.341	1.00E+00	1.313	up	1.313	1.00E+00	1.734	up	1.734	4.89E-04
SAOUHSC_00535		hypothetical protein	Alanine, serine, and glycine;Lysine, threonine, methionine, and cysteine	-0.24	0.78	1.30	1.61	▲	1.18	down	0.85	1.000E+00	1.723	up	1.723	7.58E-03	2.469	up	2.469	5.09E-09	3.058	up	3.058	6.52E-11
SAOUHSC_00009	SerS	seryl-tRNA synthetase	Alanine, serine, and glycine;Protein biosynthesis	0.25	0.69	0.15	0.16	–	1.19	up	1.19	1.000E+00	1.617	up	1.617	1.02E-02	1.112	up	1.112	1.00E+00	1.114	up	1.114	1.00E+00
SAOUHSC_01833	SerA	D-3-phosphoglycerate dehydrogenase	Alanine, serine, and glycine;Pyridoxine	0.03	1.22	1.87	1.61	▲	1.02	up	1.02	1.000E+00	2.331	up	2.331	4.02E-12	3.662	up	3.662	3.68E-14	3.061	up	3.061	2.03E-16
SAOUHSC_01045		hypothetical protein	Arginine; urea cycle, polyamines	-0.01	-1.51	n.d.	-0.30	▼	1.01	down	0.99	1.000E+00	2.856	down	0.350	1.40E-04					1.227	down	0.815	1.00E+00
SAOUHSC_01046	PotA	ABC transporter	Arginine; urea cycle, polyamines	-0.94	-1.57	-0.73	-0.84	▼	1.92	down	0.52	1.681E-04	2.970	down	0.337	2.71E-07	1.653	down	0.605	1.00E+00	1.790	down	0.559	1.00E+00
SAOUHSC_01047	PotB	spermidine/putrescine ABC transporter permease	Arginine; urea cycle, polyamines	-1.01	-1.69	n.d.	-0.90	▼	2.01	down	0.50	4.408E-03	3.228	down	0.310	2.27E-02					1.867	down	0.536	2.98E-01
SAOUHSC_01049	PotD	spermidine/putrescine ABC transporter spermidine/putrescine-binding protein	Arginine; urea cycle, polyamines	-1.27	-1.74	n.d.	-1.44	▼	2.42	down	0.41	1.438E-03	3.341	down	0.299	2.01E-05					2.705	down	0.370	3.70E-01
SAOUHSC_01617	ArgR	arginine repressor	Arginine; urea cycle, polyamines	0.23	0.79	0.45	0.58	▲	1.18	up	1.18	1.000E+00	1.731	up	1.731	1.70E-02	1.365	up	1.365	1.00E+00	1.497	up	1.497	1.00E+00
SAOUHSC_02244		succinyl-diaminopimelate desuccinylase	Arginine; urea cycle, polyamines	1.15	2.17	2.03	2.13	▲	2.22	up	2.22	4.750E-01	4.505	up	4.505	4.92E-01	4.085	up	4.085	4.45E-02	4.390	up	4.390	4.02E-02
SAOUHSC_00076	SbnB	ornithine cyclodeaminase	Arginine; urea cycle, polyamines;Lysine, threonine, methionine, and cysteine;Proline and 4-hydroxyproline;Siderophores	-2.99	2.02	2.41	2.20	▲	7.96	down	0.13	5.917E-01	4.050	up	4.050	1.86E-10	5.305	up	5.305	1.82E-13	4.593	up	4.593	1.02E-10
SAOUHSC_00894	RocD	ornithine--oxo-acid transaminase	Arginine; urea cycle, polyamines;Proline and 4-hydroxyproline	0.58	1.59	2.64	2.91	▲	1.50	up	1.50	1.000E+00	3.002	up	3.002	1.24E-23	6.226	up	6.226	5.49E-17	7.524	up	7.524	4.95E-23
SAOUHSC_00899	ArgG	argininosuccinate synthase	Arginine; urea cycle, polyamines;Proline and 4-hydroxyproline;Miscellaneous - no subcategory	-0.95	0.89	1.66	1.34	▲	1.93	down	0.52	1.000E+00	1.850	up	1.850	8.80E-03	3.161	up	3.161	2.83E-03	2.532	up	2.532	2.67E-05
SAOUHSC_00733	HisC	histidinol-phosphate aminotransferase	Aromatic amino acids and derivatives	0.53	1.41	1.49	1.46	▲	1.44	up	1.44	1.000E+00	2.652	up	2.652	1.98E-04	2.811	up	2.811	6.72E-05	2.750	up	2.750	5.70E-05
SAOUHSC_00724		chorismate binding enzyme	Aromatic amino acids and derivatives;Folate and pterines	-0.06	0.94	2.70	0.40	▲	1.04	down	0.96	1.000E+00	1.921	up	1.921	1.54E-01	6.480	up	6.480	2.10E-02	1.321	up	1.321	1.00E+00
SAOUHSC_01014	PurF	amidophosphoribosyltransferase	Bacteriocins, ribosomally synthesized antibacterial peptides;Purines	-0.07	0.54	-1.00	-0.81	▼	1.05	down	0.95	1.000E+00	1.451	up	1.451	1.00E+00	2.006	down	0.498	1.04E-07	1.754	down	0.570	1.68E-02
SAOUHSC_00144	AusA	hypothetical protein	Biotin	-0.63	1.85	2.15	2.27	▲	1.55	down	0.65	1.000E+00	3.600	up	3.600	5.87E-21	4.429	up	4.429	7.66E-11	4.826	up	4.826	3.33E-18
SAOUHSC_02713	BioF	hypothetical protein	Biotin	1.29	1.32	-0.14	-0.08	–	2.44	up	2.44	2.506E-07	2.494	up	2.494	8.55E-09	1.100	down	0.909	1.00E+00	1.056	down	0.947	1.00E+00
SAOUHSC_02714	BioB	biotin synthase	Biotin	1.45	0.57	-2.17	-1.84	▼	2.74	up	2.74	9.083E-16	1.486	up	1.486	1.00E+00	4.495	down	0.222	1.11E-05	3.582	down	0.279	3.47E-05
SAOUHSC_02715	BioA	adenosylmethionine-8-amino-7-oxononanoate aminotransferase	Biotin	1.07	0.89	0.64	0.36	▲	2.10	up	2.10	6.236E-10	1.853	up	1.853	2.30E-05	1.560	up	1.560	4.34E-01	1.281	up	1.281	1.00E+00
SAOUHSC_02716	BioD	dethiobiotin synthase	Biotin	1.06	0.72	-0.11	-0.37	–	2.09	up	2.09	7.263E-05	1.650	up	1.650	1.49E-01	1.077	down	0.928	1.00E+00	1.292	down	0.774	1.00E+00
SAOUHSC_00336	Thl	acetyl-CoA acetyltransferase	Biotin;Fermentation;Isoprenoids;Fatty Acids, Lipids, and Isoprenoids - no subcategory;One-carbon Metabolism;Branched-chain amino acids	-0.14	0.81	-0.11	-0.18	–	1.10	down	0.91	1.000E+00	1.757	up	1.757	3.90E-09	1.082	down	0.924	1.00E+00	1.131	down	0.884	1.00E+00
SAOUHSC_02284	IlvC	ketol-acid reductoisomerase	Branched-chain amino acids;Coenzyme A	0.54	1.18	2.39	2.28	▲	1.45	up	1.45	1.000E+00	2.273	up	2.273	8.81E-07	5.239	up	5.239	9.09E-10	4.852	up	4.852	1.75E-11
SAOUHSC_02589		hypothetical protein	Capsular and extracellular polysacchrides	0.45	0.27	0.64	0.75	▲	1.37	up	1.37	1.000E+00	1.206	up	1.206	1.00E+00	1.560	up	1.560	1.00E+00	1.683	up	1.683	4.33E-02
SAOUHSC_01589	ScpA	hypothetical protein	Cell Division and Cell Cycle - no subcategory	-1.41	-1.35	n.d.	-1.18	▼	2.66	down	0.38	1.000E+00	2.546	down	0.393	4.98E-03					2.261	down	0.442	4.02E-02
SAOUHSC_02012	SgtB	glycosyltransferase	Cell Wall and Capsule - no subcategory	-0.48	2.06	1.48	1.21	▲	1.39	down	0.72	1.000E+00	4.172	up	4.172	7.71E-18	2.781	up	2.781	6.48E-10	2.308	up	2.308	8.81E-11
SAOUHSC_02318	Ddl	D-alanyl-alanine synthetase A	Cell Wall and Capsule - no subcategory	0.15	0.60	0.57	0.45	–	1.11	up	1.11	1.000E+00	1.519	up	1.519	4.19E-02	1.487	up	1.487	2.89E-01	1.365	up	1.365	1.00E+00
SAOUHSC_02365	MurA2	UDP-N-acetylglucosamine 1-carboxyvinyltransferase	Cell Wall and Capsule - no subcategory	-0.05	1.27	0.29	0.26	–	1.04	down	0.96	1.000E+00	2.405	up	2.405	7.03E-22	1.223	up	1.223	1.00E+00	1.195	up	1.195	1.00E+00
SAOUHSC_00994	Atl	bifunctional autolysin	Cell Wall and Capsule - no subcategory;Regulation and Cell signaling - no subcategory;Adhesion;Quorum sensing and biofilm formation	0.64	-1.29	-1.95	-2.37	▼	1.56	up	1.56	8.675E-07	2.440	down	0.410	2.14E-37	3.865	down	0.259	1.82E-24	5.178	down	0.193	4.03E-24
SAOUHSC_00153		indolepyruvate decarboxylase	Central carbohydrate metabolism	0.30	-0.46	0.74	0.85	▲	1.23	up	1.23	1.000E+00	1.377	down	0.726	1.00E+00	1.669	up	1.669	4.70E-01	1.800	up	1.800	1.27E-03
SAOUHSC_00655		dihydroxyacetone kinase subunit DhaK	Central carbohydrate metabolism	0.92	2.90	4.32	4.47	▲	1.90	up	1.90	4.074E-02	7.468	up	7.468	5.63E-08	19.910	up	19.910	5.78E-06	22.173	up	22.173	4.90E-08
SAOUHSC_00797	TpiA	triosephosphate isomerase	Central carbohydrate metabolism	0.86	0.05	-0.34	-0.32	–	1.81	up	1.81	6.613E-08	1.035	up	1.035	1.00E+00	1.265	down	0.791	1.00E+00	1.252	down	0.799	1.00E+00
SAOUHSC_01337	Tkt	transketolase	Central carbohydrate metabolism	0.37	0.83	0.14	0.31	–	1.29	up	1.29	1.000E+00	1.777	up	1.777	3.38E-16	1.099	up	1.099	1.00E+00	1.241	up	1.241	1.00E+00
SAOUHSC_01418	SucA	2-oxoglutarate dehydrogenase E1 component	Central carbohydrate metabolism	-0.35	0.45	1.24	1.54	▲	1.27	down	0.79	1.000E+00	1.367	up	1.367	1.00E+00	2.364	up	2.364	1.70E-22	2.917	up	2.917	9.34E-41
SAOUHSC_01710		acetyl-CoA carboxylase, biotin carboxyl carrier protein	Central carbohydrate metabolism	0.45	0.08	0.88	1.04	▲	1.37	up	1.37	1.000E+00	1.057	up	1.057	1.00E+00	1.838	up	1.838	2.78E-01	2.060	up	2.060	6.85E-03
SAOUHSC_01801	CitC	isocitrate dehydrogenase	Central carbohydrate metabolism	0.25	0.77	2.02	2.45	▲	1.19	up	1.19	1.000E+00	1.701	up	1.701	1.04E-04	4.048	up	4.048	4.62E-22	5.463	up	5.463	1.19E-29
SAOUHSC_01818	Ald2	alanine dehydrogenase	Central carbohydrate metabolism	0.09	0.36	1.09	1.44	▲	1.06	up	1.06	1.000E+00	1.287	up	1.287	1.00E+00	2.122	up	2.122	2.71E-12	2.718	up	2.718	7.36E-25
SAOUHSC_01867	Dat	D-alanine aminotransferase	Central carbohydrate metabolism	0.30	0.90	0.41	0.39	–	1.23	up	1.23	1.000E+00	1.869	up	1.869	1.49E-11	1.332	up	1.332	1.00E+00	1.306	up	1.306	1.00E+00
SAOUHSC_01983	FumC	fumarate hydratase	Central carbohydrate metabolism	0.24	0.77	1.35	1.45	▲	1.18	up	1.18	1.000E+00	1.700	up	1.700	1.35E-02	2.550	up	2.550	1.78E-12	2.740	up	2.740	3.58E-21
SAOUHSC_02647	Mqo1	malate:quinone oxidoreductase	Central carbohydrate metabolism	-0.18	0.16	1.01	1.05	▲	1.13	down	0.88	1.000E+00	1.116	up	1.116	1.00E+00	2.014	up	2.014	6.51E-05	2.068	up	2.068	8.17E-11
SAOUHSC_00963		lipoyltransferase and lipoate-protein ligase	Central carbohydrate metabolism;Alanine, serine, and glycine;Plant-Prokaryote comparative genomics;Lipoic acid	0.18	1.02	0.38	0.58	▲	1.13	up	1.13	1.000E+00	2.034	up	2.034	8.84E-06	1.304	up	1.304	1.00E+00	1.497	up	1.497	1.00E+00
SAOUHSC_00536	IlvE	branched-chain amino acid aminotransferase	Central carbohydrate metabolism;Branched-chain amino acids;Alanine, serine, and glycine	0.75	1.27	0.82	0.98	▲	1.69	up	1.69	9.942E-06	2.419	up	2.419	9.31E-35	1.766	up	1.766	6.72E-07	1.972	up	1.972	2.88E-16
SAOUHSC_00574	EutD	phosphotransacetylase	Central carbohydrate metabolism;Fermentation	0.33	0.67	0.11	0.21	–	1.26	up	1.26	1.000E+00	1.588	up	1.588	4.65E-02	1.079	up	1.079	1.00E+00	1.159	up	1.159	1.00E+00
SAOUHSC_01416	SucB	dihydrolipoamide succinyltransferase	Central carbohydrate metabolism;Lipoic acid	-0.44	0.64	1.61	1.86	▲	1.36	down	0.74	1.000E+00	1.561	up	1.561	1.26E-01	3.052	up	3.052	1.45E-13	3.638	up	3.638	1.66E-17
SAOUHSC_00553	HxlA	hypothetical protein	Central carbohydrate metabolism;One-carbon Metabolism	1.08	0.38	-0.07	0.12	–	2.11	up	2.11	4.226E-10	1.300	up	1.300	1.00E+00	1.049	down	0.954	1.00E+00	1.085	up	1.085	1.00E+00
SAOUHSC_01216	SucC	succinyl-CoA synthetase subunit beta	Central carbohydrate metabolism;One-carbon Metabolism	-0.04	0.13	1.01	1.11	▲	1.03	down	0.97	1.000E+00	1.097	up	1.097	1.00E+00	2.017	up	2.017	1.28E-15	2.159	up	2.159	1.13E-31
SAOUHSC_01218	SucD	succinyl-CoA synthetase subunit alpha	Central carbohydrate metabolism;One-carbon Metabolism	0.16	0.20	1.01	1.27	▲	1.12	up	1.12	1.000E+00	1.149	up	1.149	1.00E+00	2.021	up	2.021	1.55E-09	2.420	up	2.420	2.73E-20
SAOUHSC_01910	PckA	phosphoenolpyruvate carboxykinase	Central carbohydrate metabolism;One-carbon Metabolism	0.38	2.50	3.82	4.16	▲	1.30	up	1.30	1.000E+00	5.660	up	5.660	7.59E-07	14.152	up	14.152	2.75E-04	17.934	up	17.934	2.61E-06

locus tag	protein symbol	description	theSEED functional category	log2 ratio OD=0.4	log2 ratio 8h p.i.	log2 ratio 24h p.i.	log2 ratio 32h p.i.	median over 8,24 and 32h ratio; median tendence - 1.5fc cutoff	median fold-change OD=0.4	median fold-change (direction) OD=0.4	median ratio over assays against non-ad. OD=0.4	p-value BH adjusted OD=0.4	median fold-change 8h p.i.	median fold-change (direction) 8h p.i.	median ratio over assays against non-ad. 8h p.i.	p-value BH adjusted 8h p.i.	median fold-change 24h p.i.	median fold-change (direction) 24h p.i.	median ratio over assays against non-ad. 24h p.i.	p-value BH adjusted 24h p.i.	median fold-change 32h p.i.	median fold-change (direction) 32h p.i.	median ratio over assays against non-ad. 32h p.i.	p-value BH adjusted 32h p.i.
SAOUHSC_02143		hypothetical protein	Central carbohydrate metabolism;One-carbon Metabolism	0.53	0.79	0.54	0.66	▲	1.45	up	1.45	9.250E-02	1.732	up	1.732	2.07E-04	1.457	up	1.457	1.00E+00	1.578	up	1.578	5.67E-01
SAOUHSC_02577		D-isomer specific 2-hydroxyacid dehydrogenase NAD binding domain-containing protein	Central carbohydrate metabolism;Organic acids	0.32	1.12	2.01	2.18	▲	1.25	up	1.25	1.000E+00	2.181	up	2.181	6.74E-23	4.019	up	4.019	1.14E-27	4.518	up	4.518	3.82E-38
SAOUHSC_02142	AldH	aldehyde dehydrogenase	Central carbohydrate metabolism;Phospholipids	0.12	0.67	1.20	1.43	▲	1.09	up	1.09	1.000E+00	1.592	up	1.592	1.25E-02	2.296	up	2.296	8.39E-14	2.701	up	2.701	2.20E-25
SAOUHSC_02849	CidC	pyruvate oxidase	Central carbohydrate metabolism;Programmed Cell Death and Toxin-antitoxin Systems	0.19	-1.07	-0.48	-0.32	–	1.14	up	1.14	1.000E+00	2.092	down	0.478	1.72E-11	1.398	down	0.715	1.00E+00	1.252	down	0.799	1.00E+00
SAOUHSC_00879	AmpA	cytosol aminopeptidase	Central carbohydrate metabolism;Protein degradation	-1.23	-0.34	0.12	0.37	–	2.35	down	0.43	3.301E-03	1.264	down	0.791	1.00E+00	1.087	up	1.087	1.00E+00	1.296	up	1.296	1.00E+00
SAOUHSC_02918	PanC	pantoate--beta-alanine ligase	Coenzyme A	-0.34	-0.42	-1.28	-1.41	▼	1.26	down	0.79	1.000E+00	1.340	down	0.747	1.00E+00	2.436	down	0.410	4.45E-02	2.658	down	0.376	1.88E-05
SAOUHSC_01824	ThiI	thiamine biosynthesis protein ThiI	Cofactors, Vitamins, Prosthetic Groups, Pigments - no subcategory	-0.95	-0.56	-0.32	-0.47	–	1.94	down	0.52	1.071E-05	1.470	down	0.680	1.00E+00	1.250	down	0.800	1.00E+00	1.390	down	0.720	1.00E+00
SAOUHSC_02329	ThiM	hydroxyethylthiazole kinase	Cofactors, Vitamins, Prosthetic Groups, Pigments - no subcategory	0.59	2.72	2.35	2.59	▲	1.50	up	1.50	1.000E+00	6.581	up	6.581	2.13E-07	5.113	up	5.113	4.02E-05	6.006	up	6.006	2.61E-06
SAOUHSC_02330	ThiD2	phosphomethylpyrimidine kinase	Cofactors, Vitamins, Prosthetic Groups, Pigments - no subcategory	0.42	1.40	2.55	2.30	▲	1.34	up	1.34	1.000E+00	2.647	up	2.647	3.45E-03	5.868	up	5.868	4.86E-01	4.933	up	4.933	2.59E-01
SAOUHSC_00819	CspC	hypothetical protein	Cold shock	-0.63	0.05	-1.20	-1.27	▼	1.55	down	0.64	1.000E+00	1.036	up	1.036	1.00E+00	2.296	down	0.436	4.21E-02	2.414	down	0.414	2.45E-03
SAOUHSC_01403	CspA	cold shock protein	Cold shock	0.35	2.14	1.95	2.15	▲	1.27	up	1.27	1.000E+00	4.410	up	4.410	1.02E-02	3.862	up	3.862	2.74E-01	4.426	up	4.426	1.11E-03
SAOUHSC_03045	CspB	cold shock protein	Cold shock	-0.41	-0.20	-2.18	-2.16	▼	1.32	down	0.75	1.000E+00	1.148	down	0.871	1.00E+00	4.529	down	0.221	1.89E-03	4.463	down	0.224	6.08E-06
SAOUHSC_01430	Crr	phosphotransferase system enzyme IIA	Di- and oligosaccharides	0.59	2.32	1.47	1.59	▲	1.51	up	1.51	1.000E+00	4.985	up	4.985	8.59E-22	2.778	up	2.778	1.78E-12	3.008	up	3.008	1.36E-16
SAOUHSC_02848	GlcB	PTS system glucose-specific transporter subunit IIABC	Di- and oligosaccharides	-0.08	0.50	0.79	0.82	▲	1.05	down	0.95	1.000E+00	1.412	up	1.412	1.00E+00	1.730	up	1.730	8.25E-04	1.763	up	1.763	3.79E-06
SAOUHSC_00349	Ssb	bacteriophage L54a, single-stranded DNA binding protein	DNA repair	-0.28	-0.87	-0.92	-0.53	▼	1.21	down	0.83	1.000E+00	1.833	down	0.546	4.96E-03	1.896	down	0.527	1.66E-01	1.449	down	0.690	1.00E+00
SAOUHSC_02123	PcrA	ATP-dependent DNA helicase PcrA	DNA repair	-0.53	-0.46	-0.74	-0.88	▼	1.44	down	0.69	1.000E+00	1.372	down	0.729	1.00E+00	1.676	down	0.597	2.10E-01	1.841	down	0.543	1.12E-08
SAOUHSC_01454		hypothetical protein	DNA repair;DNA replication	-0.80	-0.60	-1.04	-1.22	▼	1.74	down	0.58	4.294E-02	1.511	down	0.662	1.00E+00	2.063	down	0.485	4.82E-04	2.330	down	0.429	2.95E-06
SAOUHSC_01241	PolC	DNA polymerase III PolC	DNA replication	-0.45	-0.01	-0.68	-0.84	▼	1.37	down	0.73	1.000E+00	1.006	down	0.994	1.00E+00	1.604	down	0.623	1.00E+00	1.788	down	0.559	1.59E-02
SAOUHSC_01351	ParE	DNA topoisomerase IV subunit B	DNA replication;Resistance to antibiotics and toxic compounds	-0.36	-0.74	-0.96	-0.82	▼	1.28	down	0.78	1.000E+00	1.666	down	0.600	9.67E-02	1.949	down	0.513	1.11E-03	1.771	down	0.565	2.19E-04
SAOUHSC_01352	ParC	DNA topoisomerase IV subunit A	DNA replication;Resistance to antibiotics and toxic compounds	-0.32	-0.33	-0.77	-0.65	▼	1.24	down	0.80	1.000E+00	1.255	down	0.797	1.00E+00	1.700	down	0.588	1.41E-02	1.574	down	0.635	1.00E+00
SAOUHSC_00935	TrfA	adaptor protein	DNA uptake, competence	0.46	-1.00	n.d.	n.d.	▼	1.38	up	1.38	1.000E+00	1.998	down	0.501	9.45E-05								
SAOUHSC_02517	TopB	DNA topoisomerase III	DNA uptake, competence	-0.14	-1.04	-1.28	-1.77	▼	1.10	down	0.91	1.000E+00	2.055	down	0.487	3.38E-09	2.430	down	0.411	4.86E-01	3.405	down	0.294	1.44E-01
SAOUHSC_00469	SpoVG	regulatory protein SpoVG	Dormancy and Sporulation - no subcategory	0.11	-0.80	-0.64	-0.09	▼	1.08	up	1.08	1.000E+00	1.739	down	0.575	3.10E-03	1.557	down	0.642	1.00E+00	1.067	down	0.937	1.00E+00
SAOUHSC_00481		hypothetical protein	Dormancy and Sporulation - no subcategory;Heat shock	0.91	0.52	0.06	-0.07	–	1.88	up	1.88	1.167E-02	1.431	up	1.431	1.00E+00	1.042	up	1.042	1.00E+00	1.051	down	0.952	1.00E+00
SAOUHSC_01031	CydA	cytochrome d ubiquinol oxidase subunit I	Electron accepting reactions	-1.80	0.88	0.57	0.48	–	3.49	down	0.29	7.673E-04	1.845	up	1.845	9.58E-04	1.479	up	1.479	4.57E-01	1.395	up	1.395	5.37E-01
SAOUHSC_01032	CydB	cytochrome d ubiquinol oxidase subunit II	Electron accepting reactions	-1.38	1.23	1.15	0.76	▲	2.61	down	0.38	4.454E-03	2.339	up	2.339	4.07E-07	2.223	up	2.223	9.92E-02	1.692	up	1.692	1.10E-01
SAOUHSC_01104	SdhA	succinate dehydrogenase flavoprotein subunit	Electron donating reactions;Central carbohydrate metabolism;One-carbon Metabolism	0.24	0.10	1.13	1.24	▲	1.18	up	1.18	1.000E+00	1.069	up	1.069	1.00E+00	2.187	up	2.187	1.45E-13	2.362	up	2.362	1.53E-26
SAOUHSC_01105	SdhB	succinate dehydrogenase iron-sulfur subunit	Electron donating reactions;One-carbon Metabolism;Central carbohydrate metabolism	0.08	-0.34	0.48	0.69	–	1.06	up	1.06	1.000E+00	1.269	down	0.788	1.00E+00	1.393	up	1.393	1.00E+00	1.618	up	1.618	1.59E-02
SAOUHSC_00920	FabH	3-oxoacyl-(acyl carrier protein) synthase III	Fatty acids	-1.39	-0.86	-0.96	-0.77	▼	2.63	down	0.38	4.292E-13	1.809	down	0.553	4.13E-05	1.943	down	0.515	1.85E-05	1.708	down	0.585	1.65E-03
SAOUHSC_00921	FabF	3-oxoacyl- synthase	Fatty acids	-0.92	-0.52	-0.61	-0.37	–	1.89	down	0.53	1.156E-07	1.435	down	0.697	1.00E+00	1.529	down	0.654	1.00E+00	1.290	down	0.775	1.00E+00
SAOUHSC_00086	ButA	acetoin reductase	Fermentation	0.82	2.29	3.34	3.68	▲	1.76	up	1.76	3.457E-03	4.897	up	4.897	4.32E-16	10.129	up	10.129	9.74E-13	12.818	up	12.818	3.65E-17
SAOUHSC_00206	Ldh1	L-lactate dehydrogenase	Fermentation	-1.15	-0.25	-2.09	-2.87	▼	2.22	down	0.45	3.910E-03	1.193	down	0.838	1.00E+00	4.254	down	0.235	1.77E-07	7.290	down	0.137	1.47E-02
SAOUHSC_02922	Ldh2	L-lactate dehydrogenase	Fermentation	0.51	0.89	0.25	0.35	–	1.43	up	1.43	1.000E+00	1.860	up	1.860	1.18E-05	1.188	up	1.188	1.00E+00	1.272	up	1.272	1.00E+00
SAOUHSC_00608	Adh	alcohol dehydrogenase	Fermentation;Phospholipids	-1.11	0.89	-0.40	-0.54	–	2.16	down	0.46	6.072E-10	1.859	up	1.859	7.87E-06	1.319	down	0.758	1.00E+00	1.458	down	0.686	1.00E+00
SAOUHSC_00187	PflB	formate acetyltransferase	Fermentation;Respiration - no subcategory	-2.19	2.07	1.19	0.75	▲	4.57	down	0.22	4.750E-01	4.198	up	4.198	2.31E-17	2.288	up	2.288	5.10E-07	1.677	up	1.677	3.06E-03
SAOUHSC_01434	DfrA	dihydrofolate reductase	Folate and pterines	0.49	0.72	0.99	1.03	▲	1.40	up	1.40	1.000E+00	1.652	up	1.652	7.97E-01	1.981	up	1.981	1.75E-04	2.042	up	2.042	2.33E-05
SAOUHSC_02360	Tdk	thymidine kinase	Folate and pterines;Pyrimidines	-0.32	-0.36	-0.86	-0.71	▼	1.25	down	0.80	1.000E+00	1.285	down	0.778	1.00E+00	1.814	down	0.551	1.52E-02	1.635	down	0.612	3.72E-01
SAOUHSC_00549	FolE2	putative GTP cyclohydrolase	Folate and pterines;RNA processing and modification	0.87	-0.27	-0.40	-0.01	–	1.83	up	1.83	1.064E-03	1.206	down	0.829	1.00E+00	1.316	down	0.760	1.00E+00	1.004	down	0.996	1.00E+00
SAOUHSC_00895	GudB	glutamate dehydrogenase, NAD-specific	Glutamine, glutamate, aspartate, asparagine; ammonia assimilation	-0.36	1.13	1.76	2.09	▲	1.28	down	0.78	1.000E+00	2.182	up	2.182	3.70E-24	3.380	up	3.380	6.76E-25	4.267	up	4.267	1.19E-38
SAOUHSC_00640	TagA	teichoic acid biosynthesis protein	Gram-Positive cell wall components	-1.59	0.02	-0.77	-0.18	–	3.00	down	0.33	2.079E-02	1.015	up	1.015	1.00E+00	1.703	down	0.587	1.00E+00	1.134	down	0.882	1.00E+00
SAOUHSC_00728	LtaS	hypothetical protein	Gram-Positive cell wall components	0.19	0.97	0.96	0.91	▲	1.14	up	1.14	1.000E+00	1.965	up	1.965	2.93E-03	1.944	up	1.944	2.27E-02	1.877	up	1.877	2.78E-02
SAOUHSC_00973	TarM	hypothetical protein	Gram-Positive cell wall components	-0.50	-1.55	-0.46	-0.41	–	1.41	down	0.71	1.000E+00	2.921	down	0.342	1.61E-04	1.380	down	0.725	1.00E+00	1.329	down	0.753	1.00E+00
SAOUHSC_00974		glycosyl transferase, group 1	Gram-Positive cell wall components	-0.13	-1.15	-0.18	-0.16	–	1.09	down	0.91	1.000E+00	2.219	down	0.451	8.30E-07	1.133	down	0.882	1.00E+00	1.119	down	0.894	1.00E+00
SAOUHSC_02649		hypothetical protein	Gram-Positive cell wall components	0.03	1.26	n.d.	1.07	▲	1.02	up	1.02	1.000E+00	2.395	up	2.395	2.32E-06					2.093	up	2.093	8.01E-01
SAOUHSC_00225	TarI	2-C-methyl-D-erythritol 4-phosphate cytidylyltransferase	Gram-Positive cell wall components;Plant-Prokaryote comparative genomics;Isoprenoids	0.76	0.37	0.44	0.62	–	1.70	up	1.70	7.673E-04	1.296	up	1.296	1.00E+00	1.357	up	1.357	1.00E+00	1.536	up	1.536	2.25E-01
SAOUHSC_00872	DltD	extramembranal protein	Gram-Positive cell wall components;Resistance to antibiotics and toxic compounds	-0.60	-1.20	-0.44	-0.40	–	1.52	down	0.66	1.000E+00	2.289	down	0.437	1.83E-10	1.359	down	0.736	1.00E+00	1.316	down	0.760	1.00E+00
SAOUHSC_01679	MtaB	hypothetical protein	Heat shock;Protein processing and modification;RNA processing and modification	-0.23	-1.36	n.d.	n.d.	▼	1.17	down	0.85	1.000E+00	2.564	down	0.390	3.84E-04								
SAOUHSC_01738	HisS	histidyl-tRNA synthetase	Histidine Metabolism;Organic sulfur assimilation;Regulation and Cell signaling - no subcategory	-0.16	-0.50	-1.03	-0.89	▼	1.11	down	0.90	1.000E+00	1.417	down	0.706	1.00E+00	2.035	down	0.491	2.20E-04	1.847	down	0.541	1.51E-04
SAOUHSC_00020	WalR	two-component response regulator	Iron acquisition and metabolism - no subcategory	0.42	0.78	0.70	0.85	▲	1.34	up	1.34	1.000E+00	1.713	up	1.713	8.60E-04	1.625	up	1.625	1.41E-02	1.802	up	1.802	5.05E-04
SAOUHSC_00130	IsdI	heme-degrading monooxygenase IsdI	Iron acquisition and metabolism - no subcategory	0.08	1.48	2.15	2.45	▲	1.06	up	1.06	1.000E+00	2.782	up	2.782	5.81E-09	4.423	up	4.423	2.12E-11	5.460	up	5.460	2.66E-11
SAOUHSC_00749	SstD	hypothetical protein	Iron acquisition and metabolism - no subcategory	-2.15	-0.27	-0.81	-1.18	▼	4.42	down	0.23	5.936E-12	1.202	down	0.832	1.00E+00	1.755	down	0.570	7.45E-01	2.270	down	0.441	4.83E-03
SAOUHSC_02434		hypothetical protein	Iron acquisition and metabolism - no subcategory	-0.79	1.57	1.46	0.97	▲	1.72	down	0.58	3.892E-01	2.961	up	2.961	2.32E-14	2.754	up	2.754	2.70E-10	1.965	up	1.965	9.62E-06
SAOUHSC_02436		hypothetical protein	Iron acquisition and metabolism - no subcategory	-0.42	1.02	0.77	0.64	▲	1.33	down	0.75	1.000E+00	2.025	up	2.025	6.93E-11	1.707	up	1.707	1.42E-02	1.561	up	1.561	1.38E-01
SAOUHSC_01081	IsdA	hypothetical protein	Iron acquisition and metabolism - no subcategory;Gram-Positive cell wall components	-2.10	0.43	-1.64	-2.12	▼	4.28	down	0.23	1.229E-11	1.348	up	1.348	1.00E+00	3.126	down	0.320	1.58E-06	4.361	down	0.229	4.90E-08
SAOUHSC_02860	MvaS	HMG-CoA synthase	Isoprenoids	0.35	1.06	0.40	0.45	–	1.27	up	1.27	1.000E+00	2.090	up	2.090	2.75E-18	1.321	up	1.321	1.00E+00	1.362	up	1.362	1.00E+00
SAOUHSC_02877	CrtN	squalene synthase	Isoprenoids	0.08	-1.34	n.d.	-0.35	▼	1.06	up	1.06	1.000E+00	2.538	down	0.394	2.24E-03					1.276	down	0.783	1.00E+00
SAOUHSC_01632	GcvPB	glycine dehydrogenase subunit 2	Lipoic acid;Alanine, serine, and glycine	-0.57	0.36	0.48	0.76	–	1.49	down	0.67	1.000E+00	1.282	up	1.282	1.00E+00	1.398	up	1.398	1.00E+00	1.699	up	1.699	2.87E-05
SAOUHSC_00861	LipA	lipoyl synthase	Lipoic acid;Plant-Prokaryote comparative genomics	-0.61	-1.42	-2.15	-2.26	▼	1.53	down	0.65	1.000E+00	2.668	down	0.375	3.02E-13	4.426	down	0.226	2.66E-03	4.802	down	0.208	8.72E-09
SAOUHSC_00338	MetE	5-methyltetrahydropteroyltriglutamate--homocysteine S-methyltransferase	Lysine, threonine, methionine, and cysteine	0.47	0.69	0.79	1.21	▲	1.39	up	1.39	1.000E+00	1.618	up	1.618	1.69E-01	1.724	up	1.724	5.73E-01	2.316	up	2.316	3.30E-03
SAOUHSC_00488	CysK	hypothetical protein	Lysine, threonine, methionine, and cysteine	0.38	0.55	0.66	0.76	▲	1.30	up	1.30	1.000E+00	1.463	up	1.463	1.00E+00	1.575	up	1.575	1.22E-02	1.699	up	1.699	1.64E-05
SAOUHSC_00842	MetN1	ABC transporter ATP-binding protein	Lysine, threonine, methionine, and cysteine	0.54	0.20	0.93	0.92	▲	1.45	up	1.45	1.000E+00	1.151	up	1.151	1.00E+00	1.908	up	1.908	3.80E-03	1.888	up	1.888	1.63E-03
SAOUHSC_01320	Hom	homoserine dehydrogenase	Lysine, threonine, methionine, and cysteine	0.75	0.64	1.11	1.20	▲	1.68	up	1.68	3.336E-04	1.563	up	1.563	1.62E-01	2.151	up	2.151	3.01E-09	2.301	up	2.301	9.54E-14
SAOUHSC_01321	ThrC	threonine synthase	Lysine, threonine, methionine, and cysteine	0.98	0.91	1.19	1.11	▲	1.97	up	1.97	1.842E-07	1.884	up	1.884	4.83E-07	2.280	up	2.280	7.98E-08	2.158	up	2.158	2.11E-08

locus tag	protein symbol	description	theSEED functional category	log2 ratio OD=0.4	log2 ratio 8h p.i.	log2 ratio 24h p.i.	log2 ratio 32h p.i.	median over 8,24 and 32h ratio; median tendence - 1.5fc cutoff	median fold-change OD=0.4	median fold-change (direction) OD=0.4	median ratio over assays against non-ad. OD=0.4	p-value BH adjusted OD=0.4	median fold-change 8h p.i.	median fold-change (direction) 8h p.i.	median ratio over assays against non-ad. 8h p.i.	p-value BH adjusted 8h p.i.	median fold-change 24h p.i.	median fold-change (direction) 24h p.i.	median ratio over assays against non-ad. 24h p.i.	p-value BH adjusted 24h p.i.	median fold-change 32h p.i.	median fold-change (direction) 32h p.i.	median ratio over assays against non-ad. 32h p.i.	p-value BH adjusted 32h p.i.
SAOUHSC_01395	Asd	aspartate semialdehyde dehydrogenase	Lysine, threonine, methionine, and cysteine	0.51	3.31	3.45	3.51	▲	1.43	up	1.43	1.000E+00	9.921	up	9.921	2.55E-28	10.934	up	10.934	1.73E-20	11.424	up	11.424	1.15E-26
SAOUHSC_01396	DapA	dihydrodipicolinate synthase	Lysine, threonine, methionine, and cysteine	1.08	4.02	3.74	4.08	▲	2.12	up	2.12	3.890E-01	16.229	up	16.229	2.10E-09	13.404	up	13.404	4.41E-07	16.900	up	16.900	3.62E-09
SAOUHSC_01397	DapB	dihydrodipicolinate reductase	Lysine, threonine, methionine, and cysteine	-0.08	1.76	2.32	2.34	▲	1.06	down	0.95	1.000E+00	3.380	up	3.380	1.55E-02	5.011	up	5.011	6.76E-03	5.048	up	5.048	4.75E-04
SAOUHSC_01398	DapD	2,3,4,5-tetrahydropyridine-2-carboxylate N-succinyltransferase	Lysine, threonine, methionine, and cysteine	0.80	3.97	3.69	3.89	▲	1.74	up	1.74	4.012E-01	15.649	up	15.649	1.81E-10	12.945	up	12.945	1.23E-12	14.779	up	14.779	1.34E-09
SAOUHSC_01399		hypothetical protein	Lysine, threonine, methionine, and cysteine	0.45	2.60	2.87	2.93	▲	1.37	up	1.37	1.000E+00	6.065	up	6.065	3.65E-14	7.291	up	7.291	1.99E-09	7.610	up	7.610	3.81E-14
SAOUHSC_01401	LysA	diaminopimelate decarboxylase	Lysine, threonine, methionine, and cysteine	0.83	1.45	1.23	1.25	▲	1.78	up	1.78	1.262E-05	2.734	up	2.734	5.87E-21	2.340	up	2.340	9.04E-11	2.380	up	2.380	1.71E-15
SAOUHSC_02947	CysJ	sulfite reductase (NADPH) flavoprotein alpha-component	Lysine, threonine, methionine, and cysteine	0.10	0.47	1.05	0.77	▲	1.07	up	1.07	1.000E+00	1.385	up	1.385	1.00E+00	2.065	up	2.065	5.49E-06	1.703	up	1.703	3.53E-03
SAOUHSC_02447		hypothetical protein	Miscellaneous - no subcategory	0.89	0.26	1.29	1.69	▲	1.85	up	1.85	1.672E-04	1.195	up	1.195	1.00E+00	2.452	up	2.452	7.07E-09	3.223	up	3.223	1.95E-16
SAOUHSC_01624	AccB	acetyl-CoA carboxylase, biotin carboxyl carrier protein	Miscellaneous - no subcategory;Fatty acids	-0.35	-0.17	-1.05	-0.76	▼	1.28	down	0.78	1.000E+00	1.128	down	0.887	1.00E+00	2.072	down	0.483	4.38E-03	1.696	down	0.590	6.79E-01
SAOUHSC_01018	PurD	phosphoribosylamine--glycine ligase	Miscellaneous - no subcategory;Purines	-0.29	0.03	-1.40	-1.11	▼	1.22	down	0.82	1.000E+00	1.018	up	1.018	1.00E+00	2.642	down	0.378	6.11E-14	2.161	down	0.463	1.50E-11
SAOUHSC_00101	DeoB	phosphopentomutase	Monosaccharides	0.45	0.35	0.30	0.77	–	1.37	up	1.37	1.000E+00	1.277	up	1.277	1.00E+00	1.230	up	1.230	1.00E+00	1.710	up	1.710	5.58E-03
SAOUHSC_00239	RbsK	ribokinase	Monosaccharides	0.37	1.04	5.85	1.78	▲	1.29	up	1.29	1.000E+00	2.054	up	2.054	5.12E-02	57.531	up	57.531	1.00E+00	3.427	up	3.427	4.02E-02
SAOUHSC_00708	FruA	fructose specific permease	Monosaccharides	-1.80	-0.84	-0.46	-0.35	–	3.48	down	0.29	1.771E-15	1.795	down	0.557	2.24E-03	1.374	down	0.728	1.00E+00	1.274	down	0.785	1.00E+00
SAOUHSC_01907		hypothetical protein	Monosaccharides	0.11	1.41	1.95	2.08	▲	1.08	up	1.08	1.000E+00	2.653	up	2.653	3.66E-13	3.868	up	3.868	8.10E-10	4.226	up	4.226	4.77E-13
SAOUHSC_02793		hypothetical protein	Monosaccharides;Cell Wall and Capsule - no subcategory;Capsular and extracellular polysaccharides	0.10	0.91	0.21	0.42	–	1.07	up	1.07	1.000E+00	1.883	up	1.883	1.62E-08	1.158	up	1.158	1.00E+00	1.334	up	1.334	1.00E+00
SAOUHSC_01901	Tal	putative translaldolase	Monosaccharides;Central carbohydrate metabolism	0.63	0.74	0.25	0.15	–	1.55	up	1.55	9.379E-01	1.671	up	1.671	1.13E-02	1.188	up	1.188	1.00E+00	1.106	up	1.106	1.00E+00
SAOUHSC_00501	NupC	hypothetical protein	Monosaccharides;Purines	-1.10	-0.21	-0.12	-0.15	–	2.14	down	0.47	2.170E-06	1.158	down	0.864	1.00E+00	1.089	down	0.919	1.00E+00	1.110	down	0.901	1.00E+00
SAOUHSC_01287	GlnA	glutamine synthetase, type I	Nitrogen Metabolism - no subcategory;Glutamine, glutamate, aspartate, asparagine; ammonia assimilation;Cell Wall and Capsule - no subcategory	0.64	-0.02	-0.09	0.17	–	1.56	up	1.56	1.441E-02	1.014	down	0.987	1.00E+00	1.063	down	0.941	1.00E+00	1.124	up	1.124	1.00E+00
SAOUHSC_00229	ScdA	cell wall biosynthesis protein ScdA	Nitrogen Metabolism - no subcategory;Stress Response - no subcategory	-0.55	1.32	-0.42	-0.30	–	1.46	down	0.68	1.000E+00	2.488	up	2.488	1.61E-03	1.340	down	0.746	1.00E+00	1.228	down	0.814	1.00E+00
SAOUHSC_00743	NrdF	ribonucleotide-diphosphate reductase subunit beta	Nucleosides and Nucleotides - no subcategory	0.80	-0.53	-1.29	-1.32	▼	1.74	up	1.74	4.281E-04	1.442	down	0.693	1.00E+00	2.451	down	0.408	1.02E-04	2.489	down	0.402	3.85E-08
SAOUHSC_01587	RluB	ribosomal large subunit pseudouridine synthase B	Nucleosides and Nucleotides - no subcategory;Cell Division and Cell Cycle - no subcategory	-0.22	-0.38	-0.99	-0.74	▼	1.16	down	0.86	1.000E+00	1.300	down	0.769	1.00E+00	1.982	down	0.504	3.31E-05	1.665	down	0.601	2.68E-01
SAOUHSC_00742	NrdE	ribonucleotide-diphosphate reductase subunit alpha	Nucleosides and Nucleotides - no subcategory;Oxidative stress	0.59	-0.97	-1.88	-1.94	▼	1.50	up	1.50	1.000E+00	1.963	down	0.509	1.59E-18	3.681	down	0.272	2.68E-26	3.841	down	0.260	6.67E-37
SAOUHSC_02828		hypothetical protein	One-carbon Metabolism	0.37	0.13	1.81	1.63	▲	1.30	up	1.30	1.000E+00	1.091	up	1.091	1.00E+00	3.510	up	3.510	3.28E-01	3.100	up	3.100	1.00E-02
SAOUHSC_00552	NagB	hypothetical protein	One-carbon Metabolism;Aminosugars;Capsular and extracellular polysaccharides;Cell Wall and Capsule - no subcategory	0.41	1.43	0.92	0.91	▲	1.33	up	1.33	1.000E+00	2.694	up	2.694	7.13E-13	1.887	up	1.887	3.72E-03	1.880	up	1.880	2.68E-03
SAOUHSC_00900	Pgi	glucose-6-phosphate isomerase	One-carbon Metabolism;Central carbohydrate metabolism	-0.05	0.80	-0.10	0.09	–	1.03	down	0.97	1.000E+00	1.738	up	1.738	8.08E-06	1.071	down	0.934	1.00E+00	1.065	up	1.065	1.00E+00
SAOUHSC_01599	Zwf	glucose-6-phosphate 1-dehydrogenase	One-carbon Metabolism;Central carbohydrate metabolism;Fermentation	0.19	0.73	0.15	0.10	–	1.14	up	1.14	1.000E+00	1.656	up	1.656	1.57E-03	1.111	up	1.111	1.00E+00	1.072	up	1.072	1.00E+00
SAOUHSC_01007	FolD	bifunctional 5,10-methylene-tetrahydrofolate dehydrogenase/ 5,10-methylene-tetrahydrofolate cyclohydrolase	One-carbon Metabolism;Folate and pterines	0.39	0.73	0.41	0.61	▲	1.31	up	1.31	1.000E+00	1.654	up	1.654	4.49E-03	1.332	up	1.332	1.00E+00	1.531	up	1.531	3.65E-01
SAOUHSC_00731		ABC transporter	Osmotic stress	-0.34	0.73	0.12	0.04	–	1.27	down	0.79	1.000E+00	1.656	up	1.656	1.15E-02	1.084	up	1.084	1.00E+00	1.029	up	1.029	1.00E+00
SAOUHSC_02744	OpuCA	amino acid ABC transporter ATP-binding protein	Osmotic stress	0.74	0.90	1.17	1.38	▲	1.68	up	1.68	4.238E-01	1.871	up	1.871	8.35E-02	2.248	up	2.248	3.69E-03	2.599	up	2.599	2.03E-06
SAOUHSC_02932	BetA	choline dehydrogenase	Osmotic stress	0.42	-1.50	-0.77	-0.51	▼	1.34	up	1.34	1.000E+00	2.838	down	0.352	1.05E-05	1.711	down	0.584	8.20E-03	1.424	down	0.702	1.00E+00
SAOUHSC_02933	BetB	betaine aldehyde dehydrogenase	Osmotic stress	0.91	-0.95	-0.33	0.13	–	1.88	up	1.88	3.251E-10	1.935	down	0.517	3.56E-04	1.261	down	0.793	1.00E+00	1.091	up	1.091	1.00E+00
SAOUHSC_00831	OsmC	hypothetical protein	Oxidative stress	1.06	-0.30	0.06	n.d.	–	2.08	up	2.08	7.673E-04	1.231	down	0.812	1.00E+00	1.044	up	1.044	1.00E+00				
SAOUHSC_01282	BsaA	glutathione peroxidase	Oxidative stress	0.47	0.86	0.51	0.73	▲	1.39	up	1.39	1.000E+00	1.820	up	1.820	1.58E-05	1.425	up	1.425	1.00E+00	1.657	up	1.657	2.49E-02
SAOUHSC_01327	KatA	catalase	Oxidative stress	0.82	0.22	0.88	1.42	▲	1.77	up	1.77	3.283E-06	1.164	up	1.164	1.00E+00	1.841	up	1.841	4.41E-07	2.674	up	2.674	1.05E-25
SAOUHSC_02381	Dps	hypothetical protein	Oxidative stress	0.20	0.26	1.70	2.53	▲	1.15	up	1.15	1.000E+00	1.195	up	1.195	1.00E+00	3.250	up	3.250	2.68E-05	5.759	up	5.759	2.87E-13
SAOUHSC_01794	GapB	glyceraldehyde 3-phosphate dehydrogenase 2	Oxidative stress;Central carbohydrate metabolism	-0.38	0.75	0.68	1.44	▲	1.30	down	0.77	1.000E+00	1.679	up	1.679	9.24E-02	1.599	up	1.599	9.21E-01	2.716	up	2.716	9.03E-04
SAOUHSC_01653	SodA	superoxide dismutase, Mn	Oxidative stress;Nitrogen Metabolism - no subcategory	0.51	1.57	1.84	1.99	▲	1.42	up	1.42	1.000E+00	2.961	up	2.961	6.64E-08	3.584	up	3.584	4.10E-08	3.962	up	3.962	7.00E-13
SAOUHSC_00204	Hmp	globin domain-containing protein	Oxidative stress;Stress Response - no subcategory	-0.86	1.48	-0.08	0.07	–	1.81	down	0.55	2.501E-01	2.790	up	2.790	4.48E-18	1.060	down	0.943	1.00E+00	1.053	up	1.053	1.00E+00
SAOUHSC_00426	MetQ2	ABC transporter substrate-binding protein	Pathogenicity islands;Lysine, threonine, methionine, and cysteine	0.23	0.02	1.33	1.17	▲	1.18	up	1.18	1.000E+00	1.015	up	1.015	1.00E+00	2.514	up	2.514	1.13E-02	2.253	up	2.253	9.88E-03
SAOUHSC_00844	MetQ1	hypothetical protein	Pathogenicity islands;Lysine, threonine, methionine, and cysteine	0.54	0.03	0.79	0.90	▲	1.46	up	1.46	1.000E+00	1.024	up	1.024	1.00E+00	1.733	up	1.733	5.32E-03	1.873	up	1.873	6.16E-06
SAOUHSC_01239		hypothetical protein	Periplasmic Stress;Regulation and Cell signaling - no subcategory	-0.15	0.02	-0.66	-0.73	▼	1.11	down	0.90	1.000E+00	1.012	up	1.012	1.00E+00	1.584	down	0.631	1.00E+00	1.658	down	0.603	1.06E-02
SAOUHSC_02363		aldehyde dehydrogenase	Phospholipids;Central carbohydrate metabolism	0.85	0.01	0.62	0.81	▲	1.81	up	1.81	5.993E-05	1.010	up	1.010	1.00E+00	1.534	up	1.534	9.92E-02	1.753	up	1.753	5.86E-05
SAOUHSC_01800	PhoP	alkaline phosphatase synthesis transcriptional regulatory protein	Phosphorus Metabolism - no subcategory	-0.14	0.81	1.64	1.92	▲	1.10	down	0.91	1.000E+00	1.749	up	1.749	8.80E-03	3.120	up	3.120	3.32E-09	3.794	up	3.794	5.87E-14
SAOUHSC_00220	TarI'	2-C-methyl-D-erythritol 4-phosphate cytidylyltransferase	Plant-Prokaryote comparative genomics;Gram-Positive cell wall components;Isoprenoids	0.69	1.11	1.22	1.04	▲	1.61	up	1.61	8.248E-01	2.163	up	2.163	2.94E-03	2.331	up	2.331	1.73E-02	2.052	up	2.052	2.58E-02
SAOUHSC_02627		hypothetical protein	Polysaccharides	-1.19	-0.07	0.19	0.24	–	2.28	down	0.44	4.038E-04	1.051	down	0.952	1.00E+00	1.144	up	1.144	1.00E+00	1.182	up	1.182	1.00E+00
SAOUHSC_01874		hypothetical protein	Potassium metabolism - no subcategory;Oxidative stress	1.11	0.46	-0.09	0.42	–	2.16	up	2.16	2.372E-04	1.376	up	1.376	1.00E+00	1.064	down	0.940	1.00E+00	1.339	up	1.339	1.00E+00
SAOUHSC_02303	MazF	hypothetical protein	Programmed Cell Death and Toxin-antitoxin Systems	-0.43	-0.43	-1.49	-0.82	▼	1.35	down	0.74	1.000E+00	1.345	down	0.743	1.00E+00	2.801	down	0.357	3.60E-03	1.763	down	0.567	3.72E-01
SAOUHSC_01597	ProC	pyrroline-5-carboxylate reductase	Proline and 4-hydroxyproline	0.84	0.83	1.10	1.02	▲	1.80	up	1.80	2.745E-02	1.781	up	1.781	1.39E-02	2.140	up	2.140	4.24E-05	2.033	up	2.033	5.93E-08
SAOUHSC_02409	RocF	arginase	Proline and 4-hydroxyproline;Arginine; urea cycle, polyamines	0.33	0.89	2.07	2.27	▲	1.26	up	1.26	1.000E+00	1.852	up	1.852	1.46E-02	4.190	up	4.190	9.98E-04	4.814	up	4.814	9.48E-08
SAOUHSC_02869	RocA	1-pyrroline-5-carboxylate dehydrogenase	Proline and 4-hydroxyproline;Arginine; urea cycle, polyamines	0.32	3.89	5.03	5.22	▲	1.25	up	1.25	1.000E+00	14.792	up	14.792	1.25E-12	32.773	up	32.773	6.48E-10	37.244	up	37.244	4.77E-13
SAOUHSC_01695	RsfS	hypothetical protein	Proline and 4-hydroxyproline;NAD and NADP	0.15	-0.83	-1.36	-1.23	▼	1.11	up	1.11	1.000E+00	1.779	down	0.562	2.20E-01	2.565	down	0.390	2.03E-04	2.346	down	0.426	3.97E-05
SAOUHSC_00017	RplI	50S ribosomal protein L9	Protein biosynthesis	0.33	-0.81	-1.42	-1.11	▼	1.26	up	1.26	1.000E+00	1.758	down	0.569	3.03E-01	2.676	down	0.374	2.82E-04	2.157	down	0.464	3.29E-02
SAOUHSC_00348	RpsF	30S ribosomal protein S6	Protein biosynthesis	-0.38	-0.79	-0.99	-0.76	▼	1.30	down	0.77	1.000E+00	1.730	down	0.578	2.38E-01	1.992	down	0.502	1.88E-03	1.692	down	0.591	2.82E-01
SAOUHSC_00518	RplK	50S ribosomal protein L11	Protein biosynthesis	-0.01	-0.82	-1.28	-1.31	▼	1.01	down	0.99	1.000E+00	1.766	down	0.566	1.47E-03	2.436	down	0.411	2.89E-08	2.474	down	0.404	2.18E-12
SAOUHSC_00519	RplA	50S ribosomal protein L1	Protein biosynthesis	-0.21	-1.04	-1.14	-0.97	▼	1.16	down	0.86	1.000E+00	2.051	down	0.488	2.11E-06	2.200	down	0.454	6.21E-06	1.955	down	0.511	4.21E-05
SAOUHSC_00767	SaHPF	Staphylococcus aureus hibernation promoting factor	Protein biosynthesis	0.89	1.40	2.42	2.80	▲	1.86	up	1.86	1.016E-04	2.633	up	2.633	9.68E-12	5.365	up	5.365	2.41E-14	6.943	up	6.943	5.81E-19
SAOUHSC_01058	TypA	GTP-binding protein TypA	Protein biosynthesis	-0.70	-0.99	-0.68	-0.52	▼	1.62	down	0.62	1.000E+00	1.984	down	0.504	1.21E-13	1.603	down	0.624	1.00E+00	1.430	down	0.699	1.00E+00
SAOUHSC_01092	PheS	phenylalanyl-tRNA synthetase subunit alpha	Protein biosynthesis	-0.03	-1.00	-0.87	-0.81	▼	1.02	down	0.98	1.000E+00	1.994	down	0.501	3.95E-10	1.831	down	0.546	6.39E-04	1.750	down	0.571	4.11E-04
SAOUHSC_01093	PheT	phenylalanyl-tRNA synthetase subunit beta	Protein biosynthesis	-0.36	-0.90	-0.96	-0.79	▼	1.28	down	0.78	1.000E+00	1.864	down	0.537	1.58E-12	1.951	down	0.512	3.84E-06	1.727	down	0.579	2.20E-03
SAOUHSC_01211	RplS	50S ribosomal protein L19	Protein biosynthesis	-0.18	-0.71	-0.78	-0.74	▼	1.14	down	0.88	1.000E+00	1.630	down	0.613	6.34E-02	1.718	down	0.582	7.30E-02	1.675	down	0.597	3.13E-02
SAOUHSC_01678	RpsU	30S ribosomal protein S21	Protein biosynthesis	-0.30	-0.82	-1.85	-1.53	▼	1.23	down	0.81	1.000E+00	1.764	down	0.567	4.77E-01	3.607	down	0.277	1.54E-02	2.886	down	0.346	8.14E-03
SAOUHSC_01689	RpsT	30S ribosomal protein S20	Protein biosynthesis	0.16	-0.11	-1.00	-1.26	▼	1.12	up	1.12	1.000E+00	1.078	down	0.928	1.00E+00	2.003	down	0.499	4.01E-02	2.396	down	0.417	3.91E-03
SAOUHSC_01755	RpmA	50S ribosomal protein L27	Protein biosynthesis	0.43	-0.71	-1.25	-1.12	▼	1.34	up	1.34	1.000E+00	1.637	down	0.611	1.00E+00	2.382	down	0.420	1.55E-03	2.175	down	0.460	5.53E-05

locus tag	protein symbol	description	theSEED functional category	log2 ratio OD=0.4	log2 ratio 8h p.i.	log2 ratio 24h p.i.	log2 ratio 32h p.i.	median over 8,24 and 32h ratio; median tendence - 1.5fc cutoff	median fold-change OD=0.4	median fold-change (direction) OD=0.4	median ratio over assays against non-ad. OD=0.4	p-value BH adjusted OD=0.4	median fold-change 8h p.i.	median fold-change (direction) 8h p.i.	median ratio over assays against non-ad. 8h p.i.	p-value BH adjusted 8h p.i.	median fold-change 24h p.i.	median fold-change (direction) 24h p.i.	median ratio over assays against non-ad. 24h p.i.	p-value BH adjusted 24h p.i.	median fold-change 32h p.i.	median fold-change (direction) 32h p.i.	median ratio over assays against non-ad. 32h p.i.	p-value BH adjusted 32h p.i.
SAOUHSC_01757	RplU	50S ribosomal protein L21	Protein biosynthesis	-0.35	-0.95	-1.22	-1.14	▼	1.28	down	0.78	1.000E+00	1.933	down	0.517	1.14E-06	2.336	down	0.428	1.34E-11	2.206	down	0.453	1.19E-11
SAOUHSC_01829	RpsD	30S ribosomal protein S4	Protein biosynthesis	-0.50	-0.84	-0.90	-1.12	▼	1.42	down	0.71	1.000E+00	1.789	down	0.559	3.36E-02	1.862	down	0.537	4.02E-01	2.167	down	0.461	2.65E-06
SAOUHSC_02361	RpmE2	50S ribosomal protein L31 type B	Protein biosynthesis	-0.03	-0.81	-1.19	-1.14	▼	1.02	down	0.98	1.000E+00	1.759	down	0.569	2.54E-01	2.287	down	0.437	1.47E-04	2.197	down	0.455	1.05E-07
SAOUHSC_02477	RpsI	30S ribosomal protein S9	Protein biosynthesis	-0.41	-0.60	-1.23	-1.38	▼	1.33	down	0.75	1.000E+00	1.514	down	0.660	1.00E+00	2.338	down	0.428	5.10E-06	2.609	down	0.383	1.17E-08
SAOUHSC_02486	RpsK	30S ribosomal protein S11	Protein biosynthesis	-0.37	-0.98	-1.59	-1.79	▼	1.29	down	0.77	1.000E+00	1.973	down	0.507	4.76E-03	3.009	down	0.332	4.91E-08	3.455	down	0.289	6.59E-12
SAOUHSC_02487	RpsM	30S ribosomal protein S13	Protein biosynthesis	-0.33	-1.10	-1.22	-1.29	▼	1.26	down	0.79	1.000E+00	2.150	down	0.465	2.58E-06	2.333	down	0.429	2.06E-06	2.447	down	0.409	6.28E-12
SAOUHSC_02492	RplO	50S ribosomal protein L15	Protein biosynthesis	-0.22	-1.00	-1.33	-1.38	▼	1.16	down	0.86	1.000E+00	2.000	down	0.500	6.23E-04	2.522	down	0.397	1.96E-05	2.609	down	0.383	7.64E-09
SAOUHSC_02495	RplR	50S ribosomal protein L18	Protein biosynthesis	-0.03	-0.61	-0.95	-0.93	▼	1.02	down	0.98	1.000E+00	1.525	down	0.656	8.90E-01	1.936	down	0.517	4.93E-03	1.903	down	0.525	6.28E-06
SAOUHSC_02503	RpsQ	30S ribosomal protein S17	Protein biosynthesis	0.33	-0.56	-1.41	-1.54	▼	1.26	up	1.26	1.000E+00	1.478	down	0.676	1.00E+00	2.658	down	0.376	5.13E-03	2.916	down	0.343	6.85E-03
SAOUHSC_02504	RpmC	50S ribosomal protein L29	Protein biosynthesis	0.07	-0.77	-1.20	-1.11	▼	1.05	up	1.05	1.000E+00	1.705	down	0.587	1.00E+00	2.295	down	0.436	1.31E-03	2.151	down	0.465	8.51E-07
SAOUHSC_02505	RplP	50S ribosomal protein L16	Protein biosynthesis	-0.60	-1.07	-1.50	-1.37	▼	1.51	down	0.66	1.000E+00	2.104	down	0.475	1.21E-01	2.837	down	0.352	3.73E-06	2.579	down	0.388	4.96E-06
SAOUHSC_02506	RpsC	30S ribosomal protein S3	Protein biosynthesis	-0.18	-0.97	-0.70	-0.80	▼	1.13	down	0.89	1.000E+00	1.955	down	0.511	1.45E-03	1.625	down	0.615	9.70E-01	1.741	down	0.574	1.05E-04
SAOUHSC_02507	RplV	50S ribosomal protein L22	Protein biosynthesis	-0.36	-0.77	-0.77	-0.96	▼	1.29	down	0.78	1.000E+00	1.701	down	0.588	3.67E-01	1.707	down	0.586	8.57E-01	1.948	down	0.513	9.77E-04
SAOUHSC_02508	RpsS	30S ribosomal protein S19	Protein biosynthesis	-0.32	-0.89	-1.79	-2.04	▼	1.25	down	0.80	1.000E+00	1.848	down	0.541	6.39E-01	3.456	down	0.289	5.04E-02	4.123	down	0.243	2.59E-08
SAOUHSC_02509	RplB	50S ribosomal protein L2	Protein biosynthesis	-0.30	-0.84	-1.04	-1.05	▼	1.23	down	0.81	1.000E+00	1.789	down	0.559	1.69E-01	2.061	down	0.485	6.28E-04	2.065	down	0.484	1.10E-04
SAOUHSC_02511	RplD	50S ribosomal protein L4	Protein biosynthesis	-0.29	-0.85	-1.42	-1.10	▼	1.23	down	0.82	1.000E+00	1.802	down	0.555	1.52E-02	2.677	down	0.374	1.66E-05	2.143	down	0.467	1.53E-04
SAOUHSC_01038	Def	peptide deformylase	Protein biosynthesis;Central carbohydrate metabolism	0.74	-0.04	-0.10	-0.01	–	1.67	up	1.67	2.646E-03	1.025	down	0.975	1.00E+00	1.068	down	0.936	1.00E+00	1.006	down	0.994	1.00E+00
SAOUHSC_00027	OrfX	rRNA large subunit methyltransferase	Protein biosynthesis;NAD and NADP	-0.11	0.45	0.60	0.89	▲	1.08	down	0.93	1.000E+00	1.370	up	1.370	1.00E+00	1.519	up	1.519	1.00E+00	1.860	up	1.860	8.00E-03
SAOUHSC_00324		ribosomal-protein-serine acetyltransferase	Protein biosynthesis;Protein processing and modification	0.74	1.01	2.04	2.30	▲	1.67	up	1.67	1.000E+00	2.018	up	2.018	4.09E-03	4.122	up	4.122	9.98E-04	4.924	up	4.924	3.47E-05
SAOUHSC_01626	PepQ2	proline dipeptidase	Protein degradation	0.81	0.87	0.29	0.55	–	1.76	up	1.76	2.494E-04	1.824	up	1.824	1.20E-06	1.219	up	1.219	1.00E+00	1.464	up	1.464	7.13E-01
SAOUHSC_01778	ClpX	ATP-dependent protease ATP-binding subunit ClpX	Protein degradation	-0.24	-0.72	-1.53	-1.66	▼	1.18	down	0.85	1.000E+00	1.642	down	0.609	2.65E-01	2.880	down	0.347	4.86E-11	3.164	down	0.316	1.32E-17
SAOUHSC_01861		hypothetical protein	Protein degradation	0.35	-0.95	n.d.	n.d.	▼	1.27	up	1.27	1.000E+00	1.937	down	0.516	2.95E-02								
SAOUHSC_02102	Map	methionine aminopeptidase	Protein degradation;Protein biosynthesis	0.69	0.03	-0.03	-0.16	–	1.62	up	1.62	3.393E-02	1.023	up	1.023	1.00E+00	1.021	down	0.979	1.00E+00	1.115	down	0.897	1.00E+00
SAOUHSC_01972	PrsA	protein export protein PrsA	Protein folding	0.05	2.19	1.78	1.37	▲	1.03	up	1.03	1.000E+00	4.556	up	4.556	7.89E-25	3.429	up	3.429	9.86E-18	2.591	up	2.591	3.25E-15
SAOUHSC_00912	ClpB	ATP-dependent Clp protease, ATP-binding subunit ClpB	Protein folding;Protein degradation	0.01	-0.75	1.10	1.79	▲	1.01	up	1.01	1.000E+00	1.687	down	0.593	2.86E-02	2.138	up	2.138	3.33E-28	3.461	up	3.461	5.55E-78
SAOUHSC_01360	MsrA1	methionine sulfoxide reductase A	Protein processing and modification	0.52	1.08	1.02	0.99	▲	1.43	up	1.43	1.000E+00	2.112	up	2.112	8.96E-04	2.027	up	2.027	1.64E-01	1.988	up	1.988	4.52E-03
SAOUHSC_01431	MsrB	methionine sulfoxide reductase B	Protein processing and modification	0.50	2.69	1.85	1.89	▲	1.42	up	1.42	1.000E+00	6.467	up	6.467	9.68E-12	3.595	up	3.595	9.72E-09	3.694	up	3.694	8.04E-10
SAOUHSC_01432	MsrA2	methionine sulfoxide reductase A	Protein processing and modification	0.27	2.57	1.65	1.53	▲	1.20	up	1.20	1.000E+00	5.945	up	5.945	4.50E-14	3.146	up	3.146	1.87E-09	2.888	up	2.888	3.67E-12
SAOUHSC_02494	RpsE	30S ribosomal protein S5	Protein processing and modification;Protein biosynthesis	-0.01	-0.60	-1.11	-1.28	▼	1.01	down	0.99	1.000E+00	1.520	down	0.658	1.00E+00	2.159	down	0.463	1.73E-02	2.426	down	0.412	2.14E-03
SAOUHSC_00257	EsxA	hypothetical protein	Protein secretion system, Type VII	0.16	0.31	0.67	1.16	▲	1.12	up	1.12	1.000E+00	1.239	up	1.239	1.00E+00	1.593	up	1.593	1.76E-01	2.241	up	2.241	7.71E-04
SAOUHSC_00372	Xpt	xanthine phosphoribosyltransferase	Purines	-0.03	-0.37	-1.01	-0.88	▼	1.02	down	0.98	1.000E+00	1.290	down	0.775	1.00E+00	2.020	down	0.495	7.95E-05	1.841	down	0.543	1.70E-03
SAOUHSC_00374	GuaB	inosine-5-monophosphate dehydrogenase	Purines	-0.05	-0.23	-0.96	-0.75	▼	1.04	down	0.96	1.000E+00	1.176	down	0.850	1.00E+00	1.939	down	0.516	6.86E-06	1.679	down	0.596	8.42E-03
SAOUHSC_01010	PurC	phosphoribosylaminoimidazole-succinocarboxamide synthase	Purines	-0.01	0.39	-0.97	-0.69	▼	1.00	down	1.00	1.000E+00	1.309	up	1.309	1.00E+00	1.955	down	0.512	6.71E-08	1.610	down	0.621	2.26E-01
SAOUHSC_01011	PurS	phosphoribosylformylglycinamidine synthase, PurS protein	Purines	0.27	0.73	-0.84	-0.71	▼	1.21	up	1.21	1.000E+00	1.658	up	1.658	2.91E-03	1.795	down	0.557	5.96E-02	1.635	down	0.611	1.00E+00
SAOUHSC_01012	PurQ	phosphoribosylformylglycinamidine synthase I	Purines	0.14	0.54	-0.84	-0.71	▼	1.10	up	1.10	1.000E+00	1.450	up	1.450	7.30E-01	1.791	down	0.558	1.22E-02	1.633	down	0.613	2.74E-01
SAOUHSC_01015	PurM	phosphoribosylaminoimidazole synthetase	Purines	0.09	0.63	-0.56	-0.34	–	1.07	up	1.07	1.000E+00	1.542	up	1.542	1.61E-02	1.478	down	0.677	1.00E+00	1.269	down	0.788	1.00E+00
SAOUHSC_01017	PurH	bifunctional phosphoribosylaminoimidazolecarboxamide formyltransferase/IMP cyclohydrolase	Purines	-0.58	-0.17	-0.97	-0.91	▼	1.49	down	0.67	1.000E+00	1.123	down	0.890	1.00E+00	1.959	down	0.510	7.29E-03	1.878	down	0.532	5.07E-10
SAOUHSC_01330	GuaC	guanosine 5-monophosphate oxidoreductase	Purines	-0.63	-0.62	-0.86	-0.67	▼	1.55	down	0.65	8.267E-01	1.534	down	0.652	1.00E+00	1.816	down	0.551	2.32E-04	1.591	down	0.628	4.29E-01
SAOUHSC_01485	Ndk	nucleoside diphosphate kinase	Purines;Pyrimidines	0.42	0.28	1.11	1.12	▲	1.34	up	1.34	1.000E+00	1.215	up	1.215	1.00E+00	2.165	up	2.165	4.79E-04	2.171	up	2.171	2.74E-06
SAOUHSC_01170	CarB	carbamoyl phosphate synthase large subunit	Pyrimidines	-0.78	-0.81	-0.88	-0.66	▼	1.71	down	0.58	9.771E-06	1.755	down	0.570	6.33E-10	1.835	down	0.545	3.37E-05	1.579	down	0.633	1.00E+00
SAOUHSC_02377	Pdp	pyrimidine-nucleoside phosphorylase	Pyrimidines;Monosaccharides	-0.16	1.14	1.87	2.10	▲	1.12	down	0.89	1.000E+00	2.205	up	2.205	4.16E-32	3.647	up	3.647	2.58E-30	4.291	up	4.291	2.25E-44
SAOUHSC_00940		hypothetical protein	Regulation and Cell signaling - no subcategory	0.32	1.04	n.d.	n.d.	▲	1.25	up	1.25	1.000E+00	2.051	up	2.051	1.22E-04								
SAOUHSC_01361	MsrR	transcriptional regulator	Regulation and Cell signaling - no subcategory	-0.53	0.84	0.21	-0.34	–	1.45	down	0.69	1.000E+00	1.795	up	1.795	2.89E-05	1.157	up	1.157	1.00E+00	1.264	down	0.791	1.00E+00
SAOUHSC_02583		transcriptional regulator	Regulation and Cell signaling - no subcategory	-0.27	2.11	1.55	1.43	▲	1.21	down	0.83	1.000E+00	4.315	up	4.315	6.02E-19	2.925	up	2.925	5.94E-11	2.690	up	2.690	6.64E-15
SAOUHSC_00201	Opp-5A	hypothetical protein	Regulation and Cell signaling - no subcategory;ABC transporters	-0.05	1.62	n.d.	0.39	▲	1.04	down	0.97	1.000E+00	3.077	up	3.077	2.99E-10					1.310	up	1.310	1.00E+00
SAOUHSC_01979		hypothetical protein	Resistance to antibiotics and toxic compounds	-0.43	-0.92	-2.33	-2.71	▼	1.34	down	0.74	1.000E+00	1.894	down	0.528	2.70E-06	5.038	down	0.198	3.11E-11	6.538	down	0.153	1.82E-11
SAOUHSC_02630		hypothetical protein	Resistance to antibiotics and toxic compounds	-1.73	-1.56	-1.60	-1.82	▼	3.32	down	0.30	1.000E+00	2.955	down	0.338	1.54E-01	3.031	down	0.330	2.18E-01	3.519	down	0.284	3.58E-02
SAOUHSC_02635	TcaA	hypothetical protein	Resistance to antibiotics and toxic compounds	-0.57	0.76	0.60	0.26	▲	1.48	down	0.67	1.000E+00	1.688	up	1.688	9.26E-04	1.515	up	1.515	1.00E+00	1.198	up	1.198	1.00E+00
SAOUHSC_00835		hypothetical protein	Resistance to antibiotics and toxic compounds;Electron accepting reactions	0.44	1.41	0.94	1.14	▲	1.36	up	1.36	1.000E+00	2.664	up	2.664	4.17E-12	1.918	up	1.918	5.47E-04	2.202	up	2.202	9.06E-08
SAOUHSC_02873	CopA	cation transporter E1-E2 family ATPase	Resistance to antibiotics and toxic compounds;Electron accepting reactions	-0.07	1.42	1.29	1.29	▲	1.05	down	0.95	1.000E+00	2.679	up	2.679	1.39E-11	2.440	up	2.440	2.39E-03	2.443	up	2.443	2.32E-03
SAOUHSC_00998	FmtA	fmt protein	Resistance to antibiotics and toxic compounds;Regulation and Cell signaling - no subcategory	-0.82	1.34	0.97	0.35	▲	1.77	down	0.57	6.660E-01	2.523	up	2.523	3.30E-07	1.956	up	1.956	1.06E-01	1.274	up	1.274	1.00E+00
SAOUHSC_02582	FdhA	formate dehydrogenase subunit alpha	Respiration - no subcategory	0.38	-1.37	-1.06	-0.62	▼	1.30	up	1.30	1.000E+00	2.578	down	0.388	1.97E-10	2.081	down	0.481	9.00E-02	1.535	down	0.651	1.00E+00
SAOUHSC_01347	CitB	aconitate hydratase	Respiration - no subcategory;One-carbon Metabolism;Central carbohydrate metabolism	0.83	-0.31	0.25	0.69	–	1.77	up	1.77	1.093E-14	1.243	down	0.804	1.00E+00	1.185	up	1.185	1.00E+00	1.617	up	1.617	1.30E-02
SAOUHSC_01886	RibH	6,7-dimethyl-8-ribityllumazine synthase	Riboflavin, FMN, FAD	0.21	0.10	0.28	0.82	–	1.15	up	1.15	1.000E+00	1.073	up	1.073	1.00E+00	1.213	up	1.213	1.00E+00	1.770	up	1.770	5.16E-03
SAOUHSC_00951		hypothetical protein	RNA processing and modification	0.30	1.11	2.45	2.54	▲	1.23	up	1.23	1.000E+00	2.161	up	2.161	6.50E-11	5.452	up	5.452	4.21E-13	5.804	up	5.804	9.58E-18
SAOUHSC_01440		hypothetical protein	RNA processing and modification	0.57	-1.29	-0.10	-0.14	–	1.48	up	1.48	1.000E+00	2.454	down	0.408	6.23E-04	1.071	down	0.934	1.00E+00	1.099	down	0.910	1.00E+00
SAOUHSC_02097		hypothetical protein	RNA processing and modification	0.32	-1.11	-0.99	-0.72	▼	1.25	up	1.25	1.000E+00	2.164	down	0.462	1.60E-04	1.981	down	0.505	1.00E+00	1.649	down	0.606	1.00E+00
SAOUHSC_02316	CshA	DEAD-box ATP dependent DNA helicase	RNA processing and modification	-0.67	-1.07	-0.99	-0.90	▼	1.59	down	0.63	1.000E+00	2.105	down	0.475	5.87E-21	1.990	down	0.503	7.78E-06	1.860	down	0.538	2.20E-06
SAOUHSC_00721	QueC	hypothetical protein	RNA processing and modification;Miscellaneous - no subcategory	0.79	-1.23	-0.37	-0.24	–	1.73	up	1.73	2.927E-01	2.344	down	0.427	3.09E-02	1.295	down	0.772	1.00E+00	1.185	down	0.844	1.00E+00
SAOUHSC_01269	MiaB	(dimethylallyl)adenosine tRNA methylthiotransferase	RNA processing and modification;Protein processing and modification	0.01	-1.29	-2.83	-2.78	▼	1.01	up	1.01	1.000E+00	2.437	down	0.410	2.14E-10	7.098	down	0.141	2.74E-01	6.888	down	0.145	2.12E-02
SAOUHSC_00074	SirA	periplasmic binding protein	Siderophores	-2.39	0.12	-0.03	-0.15	–	5.24	down	0.19	5.607E-19	1.090	up	1.090	1.00E+00	1.020	down	0.980	1.00E+00	1.113	down	0.899	1.00E+00
SAOUHSC_00075	SbnA	hypothetical protein	Siderophores	n.d.	2.21	2.15	1.53	▲					4.619	up	4.619	7.59E-07	4.443	up	4.443	4.86E-01	2.882	up	2.882	4.95E-06
SAOUHSC_00077	SbnC	hypothetical protein	Siderophores	n.d.	2.14	1.94	1.49	▲					4.405	up	4.405	2.01E-05	3.838	up	3.838	2.74E-03	2.811	up	2.811	2.09E-03
SAOUHSC_00079	SbnE	hypothetical protein	Siderophores	n.d.	1.93	2.30	1.88	▲					3.805	up	3.805	2.91E-10	4.940	up	4.940	4.02E-05	3.679	up	3.679	4.95E-06
SAOUHSC_00080	SbnF	hypothetical protein	Siderophores	n.d.	1.62	1.27	1.39	▲					3.070	up	3.070	7.98E-05	2.408	up	2.408	3.04E-02	2.614	up	2.614	1.27E-03

locus tag	protein symbol	description	theSEED functional category	log2 ratio OD=0.4	log2 ratio 8h p.i.	log2 ratio 24h p.i.	log2 ratio 32h p.i.	median over 8,24 and 32h ratio; median tendence - 1.5fc cutoff	median fold-change OD=0.4	median fold-change (direction) OD=0.4	median ratio over assays against non-ad. OD=0.4	p-value BH adjusted OD=0.4	median fold-change 8h p.i.	median fold-change (direction) 8h p.i.	median ratio over assays against non-ad. 8h p.i.	p-value BH adjusted 8h p.i.	median fold-change 24h p.i.	median fold-change (direction) 24h p.i.	median ratio over assays against non-ad. 24h p.i.	p-value BH adjusted 24h p.i.	median fold-change 32h p.i.	median fold-change (direction) 32h p.i.	median ratio over assays against non-ad. 32h p.i.	p-value BH adjusted 32h p.i.
SAOUHSC_01055		inositol monophosphatase family protein	Stress Response - no subcategory	-0.01	0.15	0.89	0.67	▲	1.01	down	0.99	1.000E+00	1.109	up	1.109	1.00E+00	1.851	up	1.851	3.57E-02	1.592	up	1.592	3.88E-01
SAOUHSC_01814	UspA2	hypothetical protein	Stress Response - no subcategory	0.08	0.37	0.54	0.75	–	1.06	up	1.06	1.000E+00	1.288	up	1.288	1.00E+00	1.453	up	1.453	1.00E+00	1.680	up	1.680	3.20E-04
SAOUHSC_01819	UspA1	hypothetical protein	Stress Response - no subcategory	0.30	1.46	2.61	3.08	▲	1.23	up	1.23	1.000E+00	2.745	up	2.745	1.30E-22	6.120	up	6.120	6.82E-19	8.468	up	8.468	4.61E-25
SAOUHSC_02299	RsbW	serine-protein kinase RsbW	Stress Response - no subcategory	0.28	-0.57	-0.92	-0.53	–	1.21	up	1.21	1.000E+00	1.480	down	0.676	1.00E+00	1.893	down	0.528	8.13E-03	1.449	down	0.690	1.00E+00
SAOUHSC_02430	HtsA	ABC transporter periplasmic binding protein	Stress Response - no subcategory;Iron acquisition and metabolism - no subcategory	-1.87	-0.05	0.10	0.26	–	3.64	down	0.27	1.577E-12	1.036	down	0.965	1.00E+00	1.073	up	1.073	1.00E+00	1.196	up	1.196	1.00E+00
SAOUHSC_02298	SigB	RNA polymerase sigma factor SigB	Stress Response - no subcategory;Transcription;Quorum sensing and biofilm formation;Resistance to antibiotics and toxic compounds	0.42	-0.54	-0.98	-0.77	▼	1.34	up	1.34	1.000E+00	1.449	down	0.690	1.00E+00	1.978	down	0.506	2.74E-02	1.706	down	0.586	1.77E-01
SAOUHSC_02402	MtlA	PTS system mannitol-specific transporter subunit IIA	Sugar alcohols	0.91	-0.64	0.13	0.36	–	1.88	up	1.88	1.474E-06	1.555	down	0.643	1.00E+00	1.097	up	1.097	1.00E+00	1.284	up	1.284	1.00E+00
SAOUHSC_02403	MtlD	mannitol-1-phosphate 5-dehydrogenase	Sugar alcohols	0.87	-0.38	0.23	0.51	–	1.83	up	1.83	1.474E-06	1.299	down	0.770	1.00E+00	1.169	up	1.169	1.00E+00	1.426	up	1.426	1.00E+00
SAOUHSC_01276	GlpK	glycerol kinase	Sugar alcohols;Phospholipids;Central carbohydrate metabolism	0.19	0.82	0.80	0.84	▲	1.14	up	1.14	1.000E+00	1.761	up	1.761	3.09E-05	1.737	up	1.737	6.28E-04	1.793	up	1.793	1.57E-06
SAOUHSC_00364	AhpF	alkyl hydroperoxide reductase subunit F	Sulfur Metabolism - no subcategory	1.03	0.16	0.85	1.04	▲	2.04	up	2.04	1.697E-17	1.116	up	1.116	1.00E+00	1.803	up	1.803	5.72E-07	2.062	up	2.062	1.05E-16
SAOUHSC_00365	AhpC	alkyl hydroperoxide reductase subunit C	Sulfur Metabolism - no subcategory	1.00	-0.07	1.09	1.34	▲	2.00	up	2.00	5.273E-10	1.053	down	0.950	1.00E+00	2.125	up	2.125	4.10E-08	2.525	up	2.525	4.83E-13
SAOUHSC_01822	Tpx	thiol peroxidase	Sulfur Metabolism - no subcategory	0.71	0.58	0.49	0.72	▲	1.63	up	1.63	2.646E-03	1.492	up	1.492	2.20E-01	1.409	up	1.409	1.00E+00	1.644	up	1.644	1.58E-02
SAOUHSC_01999		bacterioferritin comigratory protein	Sulfur Metabolism - no subcategory	0.87	1.07	1.04	1.31	▲	1.83	up	1.83	1.575E-01	2.096	up	2.096	5.78E-04	2.056	up	2.056	3.72E-03	2.481	up	2.481	4.49E-05
SAOUHSC_01177	RpoZ	DNA-directed RNA polymerase subunit omega	Transcription	-0.22	-0.45	-1.01	-0.96	▼	1.17	down	0.86	1.000E+00	1.364	down	0.733	1.00E+00	2.016	down	0.496	1.01E-02	1.947	down	0.514	9.62E-04
SAOUHSC_02362	Rho	transcription termination factor Rho	Transcription	-0.82	0.05	-0.16	-0.30	–	1.76	down	0.57	1.016E-04	1.035	up	1.035	1.00E+00	1.120	down	0.893	1.00E+00	1.235	down	0.810	1.00E+00
SAOUHSC_02485	RpoA	DNA-directed RNA polymerase subunit alpha	Transcription	-0.49	-0.57	-0.85	-0.65	▼	1.40	down	0.71	1.000E+00	1.488	down	0.672	1.00E+00	1.801	down	0.555	4.34E-03	1.565	down	0.639	6.26E-01
SAOUHSC_00625	MnhA2	putative monovalent cation/H+ antiporter subunit A	Uni- Sym- and Antiporters	-0.34	-1.24	-0.90	-1.08	▼	1.26	down	0.79	1.000E+00	2.368	down	0.422	4.61E-02	1.872	down	0.534	1.00E+00	2.110	down	0.474	3.18E-01
SAOUHSC_00528	RpsG	30S ribosomal protein S7	Virulence - no subcategory;Protein biosynthesis	-0.20	-1.01	-1.70	-1.93	▼	1.15	down	0.87	1.000E+00	2.008	down	0.498	1.17E-02	3.244	down	0.308	1.06E-08	3.802	down	0.263	7.88E-17
SAOUHSC_01784	RplT	50S ribosomal protein L20	Virulence - no subcategory;Protein biosynthesis	-0.15	-0.58	-1.06	-0.97	▼	1.11	down	0.90	1.000E+00	1.491	down	0.671	1.00E+00	2.089	down	0.479	8.20E-03	1.962	down	0.510	8.10E-03
SAOUHSC_00044		hypothetical protein		0.01	1.05	0.56	0.82	▲	1.00	up	1.00	1.000E+00	2.064	up	2.064	2.30E-02	1.477	up	1.477	1.00E+00	1.770	up	1.770	5.07E-01
SAOUHSC_00070	SarS	accessory regulator-like protein		0.29	-2.05	-0.89	-0.65	▼	1.22	up	1.22	1.000E+00	4.132	down	0.242	3.90E-09	1.856	down	0.539	2.25E-01	1.569	down	0.637	7.61E-01
SAOUHSC_00164		hypothetical protein		0.87	1.31	1.56	1.98	▲	1.82	up	1.82	1.000E+00	2.476	up	2.476	4.81E-02	2.958	up	2.958	5.45E-02	3.952	up	3.952	9.03E-04
SAOUHSC_00253		hypothetical protein		-1.38	-2.51	-1.55	-1.13	▼	2.60	down	0.38	3.476E-05	5.689	down	0.176	2.58E-04	2.927	down	0.342	1.89E-03	2.191	down	0.456	1.74E-04
SAOUHSC_00285		hypothetical protein		-0.26	-1.47	-0.68	-0.28	▼	1.20	down	0.83	1.000E+00	2.763	down	0.362	1.48E-06	1.603	down	0.624	1.00E+00	1.211	down	0.826	1.00E+00
SAOUHSC_00356		hypothetical protein		0.92	-1.85	-1.13	-0.82	▼	1.89	up	1.89	2.600E-07	3.608	down	0.277	6.88E-09	2.186	down	0.457	2.10E-02	1.766	down	0.566	4.55E-01
SAOUHSC_00371		hypothetical protein		0.57	1.78	2.73	2.91	▲	1.49	up	1.49	1.000E+00	3.439	up	3.439	5.56E-16	6.614	up	6.614	1.78E-12	7.540	up	7.540	7.55E-17
SAOUHSC_00427	Sle1	autolysin		-0.34	-1.18	-1.99	-2.45	▼	1.26	down	0.79	1.000E+00	2.266	down	0.441	5.91E-03	3.973	down	0.252	8.16E-02	5.474	down	0.183	2.59E-01
SAOUHSC_00428		hypothetical protein		-0.08	2.59	1.87	1.60	▲	1.06	down	0.95	1.000E+00	6.027	up	6.027	2.15E-11	3.654	up	3.654	5.17E-06	3.035	up	3.035	2.17E-06
SAOUHSC_00502	CtsR	hypothetical protein		-0.25	0.29	1.51	1.69	▲	1.19	down	0.84	1.000E+00	1.219	up	1.219	1.00E+00	2.838	up	2.838	8.10E-10	3.220	up	3.220	4.99E-10
SAOUHSC_00533	HchA	chaperone protein HchA		0.78	-0.08	-0.13	-0.02	–	1.72	up	1.72	1.398E-03	1.058	down	0.945	1.00E+00	1.093	down	0.915	1.00E+00	1.014	down	0.986	1.00E+00
SAOUHSC_00538		haloacid dehalogenase-like hydrolase		0.63	1.33	0.64	0.75	▲	1.55	up	1.55	1.000E+00	2.522	up	2.522	1.49E-11	1.564	up	1.564	9.38E-01	1.684	up	1.684	2.48E-01
SAOUHSC_00569		hypothetical protein		-0.06	-1.13	-1.29	-0.49	▼	1.04	down	0.96	1.000E+00	2.181	down	0.458	2.35E-03	2.448	down	0.408	2.51E-01	1.400	down	0.714	1.00E+00
SAOUHSC_00603		hypothetical protein		0.37	0.90	0.81	0.67	▲	1.30	up	1.30	1.000E+00	1.868	up	1.868	1.65E-02	1.757	up	1.757	5.35E-01	1.592	up	1.592	1.00E+00
SAOUHSC_00634	MntC	ABC transporter substrate-binding protein		-0.56	-1.94	-2.68	-3.14	▼	1.47	down	0.68	1.000E+00	3.831	down	0.261	1.15E-38	6.422	down	0.156	1.04E-12	8.805	down	0.114	3.38E-18
SAOUHSC_00637	MntA	hypothetical protein		-0.46	-0.85	-0.82	-0.88	▼	1.37	down	0.73	1.000E+00	1.802	down	0.555	2.45E-01	1.770	down	0.565	2.14E-01	1.842	down	0.543	5.88E-04
SAOUHSC_00647	AbcA	hypothetical protein		0.28	0.29	1.38	1.45	▲	1.22	up	1.22	1.000E+00	1.223	up	1.223	1.00E+00	2.609	up	2.609	4.87E-26	2.724	up	2.724	8.27E-38
SAOUHSC_00652	FhuA	iron compound ABC transporter ATP-binding protein		-2.06	0.01	-0.50	-0.77	–	4.18	down	0.24	1.337E-15	1.009	up	1.009	1.00E+00	1.415	down	0.707	1.00E+00	1.701	down	0.588	1.22E-02
SAOUHSC_00659		hypothetical protein		0.54	1.55	1.54	1.78	▲	1.45	up	1.45	1.000E+00	2.927	up	2.927	1.84E-02	2.914	up	2.914	1.35E-01	3.430	up	3.430	1.19E-01
SAOUHSC_00694	MgrA	hypothetical protein		0.35	0.83	0.00	-0.01	–	1.28	up	1.28	1.000E+00	1.776	up	1.776	1.75E-04	1.003	up	1.003	1.00E+00	1.007	down	0.993	1.00E+00
SAOUHSC_00714	SaeS	sensor histidine kinase SaeS		-0.95	1.31	0.47	0.12	–	1.94	down	0.52	1.961E-02	2.476	up	2.476	6.18E-14	1.382	up	1.382	1.00E+00	1.086	up	1.086	1.00E+00
SAOUHSC_00715	SaeR	response regulator		-0.26	1.78	1.12	0.82	▲	1.20	down	0.83	1.000E+00	3.441	up	3.441	6.44E-10	2.177	up	2.177	2.38E-02	1.760	up	1.760	1.71E-01
SAOUHSC_00717	SaeP	hypothetical protein		-1.74	2.50	2.97	1.24	▲	3.35	down	0.30	4.323E-01	5.661	up	5.661	7.59E-07	7.828	up	7.828	4.86E-01	2.369	up	2.369	2.12E-02
SAOUHSC_00736		hypothetical protein		0.90	-0.22	0.81	0.95	▲	1.86	up	1.86	1.854E-01	1.165	down	0.858	1.00E+00	1.753	up	1.753	2.78E-01	1.932	up	1.932	2.10E-02
SAOUHSC_00754		hypothetical protein		-0.09	-0.90	-1.04	-1.26	▼	1.06	down	0.94	1.000E+00	1.870	down	0.535	8.52E-04	2.051	down	0.488	9.92E-02	2.393	down	0.418	1.70E-03
SAOUHSC_00763		hypothetical protein		0.59	1.33	n.d.	n.d.	▲	1.50	up	1.50	1.000E+00	2.510	up	2.510	1.22E-02								
SAOUHSC_00833		hypothetical protein		-0.94	0.91	0.38	0.43	–	1.92	down	0.52	6.263E-02	1.885	up	1.885	3.88E-03	1.301	up	1.301	1.00E+00	1.347	up	1.347	1.00E+00
SAOUHSC_00834		thioredoxin		0.66	1.02	0.38	0.36	–	1.58	up	1.58	1.000E+00	2.030	up	2.030	3.09E-06	1.304	up	1.304	1.00E+00	1.279	up	1.279	1.00E+00
SAOUHSC_00850	SufU	hypothetical protein		0.98	-0.33	-0.17	0.02	–	1.98	up	1.98	4.002E-03	1.253	down	0.798	1.00E+00	1.122	down	0.891	1.00E+00	1.015	up	1.015	1.00E+00
SAOUHSC_00863		hypothetical protein		0.63	1.07	0.27	0.26	–	1.55	up	1.55	5.831E-01	2.093	up	2.093	1.23E-04	1.210	up	1.210	1.00E+00	1.198	up	1.198	1.00E+00
SAOUHSC_00865		hypothetical protein		0.39	0.83	0.38	0.47	–	1.31	up	1.31	1.000E+00	1.782	up	1.782	1.43E-04	1.299	up	1.299	1.00E+00	1.381	up	1.381	1.00E+00
SAOUHSC_00934	SpxA	transcriptional regulator Spx		1.19	1.25	0.66	0.57	▲	2.28	up	2.28	4.226E-10	2.378	up	2.378	8.24E-15	1.575	up	1.575	8.11E-03	1.481	up	1.481	1.00E+00
SAOUHSC_00958	HtrA	serine protease HtrA		-0.49	-0.32	-0.22	-1.42	–	1.40	down	0.71	1.000E+00	1.251	down	0.799	1.00E+00	1.164	down	0.859	1.00E+00	2.669	down	0.375	2.04E-03
SAOUHSC_00972		hypothetical protein		0.26	-0.90	-0.63	-0.32	▼	1.20	up	1.20	1.000E+00	1.871	down	0.534	6.91E-04	1.543	down	0.648	1.00E+00	1.244	down	0.804	1.00E+00
SAOUHSC_01054		hypothetical protein		0.48	0.82	0.66	0.60	▲	1.40	up	1.40	1.000E+00	1.770	up	1.770	2.30E-02	1.579	up	1.579	5.75E-01	1.513	up	1.513	7.68E-01
SAOUHSC_01138		hypothetical protein		0.76	0.76	1.88	2.07	▲	1.70	up	1.70	1.736E-02	1.694	up	1.694	8.18E-02	3.679	up	3.679	6.75E-11	4.185	up	4.185	4.59E-10
SAOUHSC_01154	SepF	hypothetical protein		0.04	0.04	-1.43	-1.43	▼	1.03	up	1.03	1.000E+00	1.027	up	1.027	1.00E+00	2.699	down	0.370	2.35E-07	2.690	down	0.372	1.31E-09
SAOUHSC_01185		ribosomal RNA large subunit methyltransferase N		-0.57	-2.03	-2.47	-2.57	▼	1.48	down	0.68	1.000E+00	4.076	down	0.245	2.56E-12	5.538	down	0.181	1.45E-04	5.953	down	0.168	4.90E-08
SAOUHSC_01222	TopA	DNA topoisomerase I		-0.15	-0.80	-0.88	-0.81	▼	1.11	down	0.90	1.000E+00	1.746	down	0.573	9.50E-03	1.845	down	0.542	4.40E-02	1.750	down	0.571	1.19E-01
SAOUHSC_01324		hypothetical protein		-0.40	1.28	1.32	1.09	▲	1.32	down	0.76	1.000E+00	2.432	up	2.432	1.09E-07	2.488	up	2.488	1.53E-03	2.134	up	2.134	1.96E-04
SAOUHSC_01349		hypothetical protein		0.59	1.48	0.65	0.93	▲	1.50	up	1.50	1.000E+00	2.798	up	2.798	3.72E-11	1.574	up	1.574	4.86E-01	1.906	up	1.906	7.20E-02
SAOUHSC_01383		hypothetical protein		0.30	0.69	0.25	0.22	–	1.24	up	1.24	1.000E+00	1.610	up	1.610	8.80E-03	1.185	up	1.185	1.00E+00	1.168	up	1.168	1.00E+00
SAOUHSC_01392		ABC transporter ATP-binding protein		-0.19	-0.77	-0.73	-0.64	▼	1.14	down	0.87	1.000E+00	1.701	down	0.588	1.80E-02	1.664	down	0.601	6.00E-01	1.558	down	0.642	1.00E+00
SAOUHSC_01408		hypothetical protein		0.41	0.74	0.32	0.29	–	1.33	up	1.33	1.000E+00	1.669	up	1.669	2.46E-04	1.250	up	1.250	1.00E+00	1.224	up	1.224	1.00E+00
SAOUHSC_01415		hypothetical protein		0.48	0.70	1.05	1.29	▲	1.40	up	1.40	1.000E+00	1.627	up	1.627	2.24E-01	2.072	up	2.072	8.67E-08	2.448	up	2.448	2.77E-17
SAOUHSC_01425		hypothetical protein		1.16	0.12	0.32	0.12	–	2.24	up	2.24	9.329E-03	1.087	up	1.087	1.00E+00	1.244	up	1.244	1.00E+00	1.086	up	1.086	1.00E+00
SAOUHSC_01427	CtpA	hypothetical protein		-0.09	0.81	0.47	0.08	–	1.07	down	0.94	1.000E+00	1.757	up	1.757	2.66E-07	1.389	up	1.389	1.00E+00	1.059	up	1.059	1.00E+00

locus tag	protein symbol	description	theSEED functional category	log2 ratio OD=0.4	log2 ratio 8h p.i.	log2 ratio 24h p.i.	log2 ratio 32h p.i.	median over 8,24 and 32h ratio; median tendence - 1.5fc cutoff	median fold-change OD=0.4	median fold-change (direction) OD=0.4	median ratio over assays against non-ad. OD=0.4	p-value BH adjusted OD=0.4	median fold-change 8h p.i.	median fold-change (direction) 8h p.i.	median ratio over assays against non-ad. 8h p.i.	p-value BH adjusted 8h p.i.	median fold-change 24h p.i.	median fold-change (direction) 24h p.i.	median ratio over assays against non-ad. 24h p.i.	p-value BH adjusted 24h p.i.	median fold-change 32h p.i.	median fold-change (direction) 32h p.i.	median ratio over assays against non-ad. 32h p.i.	p-value BH adjusted 32h p.i.
SAOUHSC_01429		hypothetical protein		-0.52	1.60	-0.19	-0.49	–	1.43	down	0.70	1.000E+00	3.041	up	3.041	2.01E-05	1.139	down	0.878	1.00E+00	1.405	down	0.712	1.00E+00
SAOUHSC_01438		hypothetical protein		0.66	0.91	0.12	0.28	–	1.58	up	1.58	3.074E-01	1.874	up	1.874	1.51E-03	1.086	up	1.086	1.00E+00	1.213	up	1.213	1.00E+00
SAOUHSC_01575		helix-turn-helix domain-containing protein		0.65	0.96	1.09	1.16	▲	1.57	up	1.57	1.000E+00	1.945	up	1.945	1.15E-06	2.128	up	2.128	3.39E-04	2.236	up	2.236	1.17E-08
SAOUHSC_01606		peptidase T		0.51	0.79	1.00	1.17	▲	1.42	up	1.42	1.000E+00	1.732	up	1.732	9.24E-02	1.994	up	1.994	2.23E-03	2.248	up	2.248	3.81E-07
SAOUHSC_01665		CBS domain-containing protein		-0.03	0.26	n.d.	0.83	–	1.02	down	0.98	1.000E+00	1.200	up	1.200	1.00E+00					1.777	up	1.777	2.25E-02
SAOUHSC_01666	GlyS	glycyl-tRNA synthetase		0.66	-0.07	-0.17	-0.01	–	1.58	up	1.58	3.910E-03	1.047	down	0.955	1.00E+00	1.123	down	0.891	1.00E+00	1.004	down	0.996	1.00E+00
SAOUHSC_01701		hypothetical protein		-1.42	-1.01	-0.51	-1.52	▼	2.68	down	0.37	1.749E-07	2.017	down	0.496	1.70E-04	1.425	down	0.702	1.00E+00	2.870	down	0.348	2.46E-04
SAOUHSC_01708		LamB/YcsF family protein		0.53	0.26	0.67	1.04	▲	1.44	up	1.44	1.000E+00	1.195	up	1.195	1.00E+00	1.586	up	1.586	7.60E-01	2.055	up	2.055	9.88E-03
SAOUHSC_01716		hypothetical protein		-0.70	-1.57	n.d.	-2.07	▼	1.63	down	0.62	1.000E+00	2.967	down	0.337	3.81E-05					4.209	down	0.238	2.59E-01
SAOUHSC_01717		hypothetical protein		-0.50	-1.38	-1.51	-2.25	▼	1.41	down	0.71	1.000E+00	2.607	down	0.384	1.34E-07	2.852	down	0.351	2.74E-01	4.765	down	0.210	2.59E-01
SAOUHSC_01720		Holliday junction resolvase-like protein		0.36	0.87	0.23	0.28	–	1.28	up	1.28	1.000E+00	1.822	up	1.822	1.58E-02	1.177	up	1.177	1.00E+00	1.217	up	1.217	1.00E+00
SAOUHSC_01721		hypothetical protein		0.57	0.88	0.34	0.75	▲	1.48	up	1.48	1.000E+00	1.842	up	1.842	1.35E-02	1.265	up	1.265	1.00E+00	1.679	up	1.679	2.86E-01
SAOUHSC_01729		hypothetical protein		2.17	-0.03	0.42	-0.36	–	4.49	up	4.49	3.192E-03	1.019	down	0.981	1.00E+00	1.333	up	1.333	1.00E+00	1.280	down	0.781	1.00E+00
SAOUHSC_01730	CsbD	hypothetical protein		0.49	-0.90	-0.61	-0.33	▼	1.40	up	1.40	1.000E+00	1.864	down	0.536	1.51E-03	1.525	down	0.656	1.00E+00	1.258	down	0.795	1.00E+00
SAOUHSC_01761		hypothetical protein		-0.24	3.01	2.26	1.51	▲	1.18	down	0.85	1.000E+00	8.072	up	8.072	5.63E-08	4.799	up	4.799	1.45E-04	2.851	up	2.851	1.59E-04
SAOUHSC_01782		hypothetical protein		0.51	0.83	0.28	0.23	–	1.42	up	1.42	1.000E+00	1.782	up	1.782	6.23E-04	1.210	up	1.210	1.00E+00	1.169	up	1.169	1.00E+00
SAOUHSC_01786	InfC	translation initiation factor IF-3		-0.33	-1.02	-1.15	-0.90	▼	1.26	down	0.79	1.000E+00	2.028	down	0.493	7.09E-07	2.225	down	0.449	1.27E-05	1.872	down	0.534	2.42E-04
SAOUHSC_01787	LysP	hypothetical protein		-0.65	1.92	1.59	1.18	▲	1.57	down	0.64	1.000E+00	3.777	up	3.777	3.67E-09	3.020	up	3.020	4.85E-08	2.263	up	2.263	6.17E-07
SAOUHSC_01816	PepQ	hypothetical protein		0.14	1.67	0.94	1.07	▲	1.10	up	1.10	1.000E+00	3.181	up	3.181	1.42E-13	1.924	up	1.924	1.26E-04	2.097	up	2.097	1.01E-06
SAOUHSC_01828		GAF domain-containing protein		0.74	0.98	0.70	0.66	▲	1.67	up	1.67	9.935E-02	1.972	up	1.972	1.46E-04	1.625	up	1.625	4.66E-01	1.579	up	1.579	5.80E-01
SAOUHSC_01838		hypothetical protein		-0.29	2.46	1.24	0.61	▲	1.23	down	0.82	1.000E+00	5.495	up	5.495	9.42E-30	2.354	up	2.354	4.03E-11	1.528	up	1.528	1.05E-01
SAOUHSC_01868		dipeptidase PepV		0.52	1.19	0.55	0.74	▲	1.43	up	1.43	1.000E+00	2.287	up	2.287	2.02E-32	1.460	up	1.460	1.00E+00	1.673	up	1.673	5.17E-04
SAOUHSC_01877		hypothetical protein		-0.29	-1.11	n.d.	n.d.	▼	1.22	down	0.82	1.000E+00	2.160	down	0.463	3.55E-02								
SAOUHSC_01878		hypothetical protein		0.39	0.96	0.95	0.63	▲	1.31	up	1.31	1.000E+00	1.940	up	1.940	1.22E-02	1.932	up	1.932	6.76E-02	1.551	up	1.551	4.53E-01
SAOUHSC_01879	Rot	virulence factor regulator protein		-0.87	-1.49	-1.59	-0.92	▼	1.82	down	0.55	5.952E-01	2.805	down	0.357	2.36E-05	3.016	down	0.332	4.74E-02	1.886	down	0.530	1.34E-02
SAOUHSC_01957		hypothetical protein		-0.29	0.13	1.12	0.88	▲	1.22	down	0.82	1.000E+00	1.096	up	1.096	1.00E+00	2.181	up	2.181	1.00E+00	1.844	up	1.844	8.71E-03
SAOUHSC_01964	TRAP	hypothetical protein		0.69	0.90	0.71	0.78	▲	1.62	up	1.62	1.081E-02	1.867	up	1.867	4.01E-04	1.631	up	1.631	1.05E-01	1.723	up	1.723	2.14E-02
SAOUHSC_01968	Hit	hypothetical protein		0.74	0.81	1.13	1.46	▲	1.67	up	1.67	1.844E-01	1.758	up	1.758	2.67E-03	2.191	up	2.191	5.89E-07	2.742	up	2.742	3.69E-11
SAOUHSC_01977		hypothetical protein		0.67	0.48	0.17	0.56	–	1.59	up	1.59	3.189E-02	1.391	up	1.391	1.00E+00	1.127	up	1.127	1.00E+00	1.470	up	1.470	8.11E-01
SAOUHSC_01987		hypothetical protein		0.87	0.35	0.52	0.49	–	1.82	up	1.82	1.156E-07	1.272	up	1.272	1.00E+00	1.437	up	1.437	1.00E+00	1.408	up	1.408	1.00E+00
SAOUHSC_01998		hypothetical protein		0.06	0.21	0.99	1.06	▲	1.04	up	1.04	1.000E+00	1.159	up	1.159	1.00E+00	1.984	up	1.984	3.59E-02	2.079	up	2.079	1.41E-01
SAOUHSC_02013		hypothetical protein		1.17	-0.68	0.45	0.82	–	2.25	up	2.25	3.023E-08	1.606	down	0.622	1.07E-01	1.361	up	1.361	1.00E+00	1.760	up	1.760	3.23E-04
SAOUHSC_02096		hypothetical protein		-0.24	1.33	n.d.	0.28	▲	1.18	down	0.85	1.000E+00	2.512	up	2.512	4.34E-02					1.214	up	1.214	1.00E+00
SAOUHSC_02098	VraR	DNA-binding response regulator VraR		0.62	2.09	1.26	1.16	▲	1.53	up	1.53	6.939E-01	4.270	up	4.270	5.39E-25	2.387	up	2.387	1.02E-09	2.240	up	2.240	4.18E-10
SAOUHSC_02099	VraS	histidine kinase		-0.45	1.54	1.08	0.69	▲	1.37	down	0.73	1.000E+00	2.900	up	2.900	1.53E-09	2.116	up	2.116	7.53E-05	1.614	up	1.614	2.10E-02
SAOUHSC_02100	VraT	hypothetical protein		0.26	1.47	0.65	0.91	▲	1.20	up	1.20	1.000E+00	2.777	up	2.777	4.69E-05	1.570	up	1.570	8.28E-01	1.883	up	1.883	6.45E-01
SAOUHSC_02150		hypothetical protein		0.95	1.19	1.54	1.83	▲	1.93	up	1.93	1.156E-07	2.286	up	2.286	3.75E-10	2.902	up	2.902	4.37E-09	3.548	up	3.548	2.83E-17
SAOUHSC_02151	PmtD	hypothetical protein		1.02	1.78	2.63	2.47	▲	2.02	up	2.02	5.373E-01	3.430	up	3.430	1.81E-03	6.185	up	6.185	2.36E-02	5.536	up	5.536	1.70E-03
SAOUHSC_02152	PmtC	ABC transporter ATP-binding protein		0.39	1.15	1.84	1.98	▲	1.31	up	1.31	1.000E+00	2.213	up	2.213	4.50E-14	3.578	up	3.578	2.89E-16	3.952	up	3.952	4.95E-23
SAOUHSC_02155		hypothetical protein		0.51	1.80	n.d.	n.d.	▲	1.42	up	1.42	1.000E+00	3.480	up	3.480	4.25E-02								
SAOUHSC_02235		repressor		0.12	0.80	0.64	0.55	▲	1.09	up	1.09	1.000E+00	1.740	up	1.740	3.32E-02	1.559	up	1.559	5.60E-01	1.465	up	1.465	1.00E+00
SAOUHSC_02243	LukH	hypothetical protein		n.d.	4.08	3.46	2.62	▲					16.943	up	16.943	1.81E-03	10.978	up	10.978	1.27E-02	6.127	up	6.127	4.02E-02
SAOUHSC_02259		hypothetical protein		0.27	0.84	0.04	0.76	▲	1.21	up	1.21	1.000E+00	1.791	up	1.791	1.46E-02	1.029	up	1.029	1.00E+00	1.688	up	1.688	4.16E-01
SAOUHSC_02264	AgrC	accessory gene regulator protein C		0.48	0.13	1.28	1.07	▲	1.40	up	1.40	1.000E+00	1.094	up	1.094	1.00E+00	2.437	up	2.437	1.30E-03	2.105	up	2.105	3.91E-03
SAOUHSC_02265	AgrA	accessory gene regulator protein A		0.28	1.13	2.20	2.21	▲	1.21	up	1.21	1.000E+00	2.181	up	2.181	4.23E-09	4.597	up	4.597	3.91E-10	4.625	up	4.625	1.82E-11
SAOUHSC_02315	KdpE	DNA-binding response regulator		-0.06	-1.90	-0.06	0.25	–	1.04	down	0.96	1.000E+00	3.736	down	0.268	3.81E-05	1.041	down	0.960	1.00E+00	1.187	up	1.187	1.00E+00
SAOUHSC_02364		hypothetical protein		0.16	1.10	n.d.	0.47	▲	1.12	up	1.12	1.000E+00	2.148	up	2.148	6.88E-03					1.384	up	1.384	1.00E+00
SAOUHSC_02367		hypothetical protein		0.53	0.51	1.06	1.18	▲	1.44	up	1.44	1.000E+00	1.422	up	1.422	7.37E-01	2.084	up	2.084	9.68E-03	2.269	up	2.269	5.49E-04
SAOUHSC_02372		hypothetical protein		0.06	-0.05	-1.49	-1.66	▼	1.04	up	1.04	1.000E+00	1.033	down	0.968	1.00E+00	2.804	down	0.357	9.92E-02	3.157	down	0.317	2.12E-02
SAOUHSC_02382		hypothetical protein		-0.14	0.82	0.15	0.50	–	1.10	down	0.91	1.000E+00	1.771	up	1.771	2.44E-02	1.113	up	1.113	1.00E+00	1.417	up	1.417	1.00E+00
SAOUHSC_02387		hypothetical protein		0.88	-0.45	0.42	0.73	–	1.84	up	1.84	3.670E-03	1.368	down	0.731	1.00E+00	1.338	up	1.338	1.00E+00	1.653	up	1.653	8.85E-01
SAOUHSC_02425		hypothetical protein		0.79	2.62	3.78	4.10	▲	1.73	up	1.73	6.660E-01	6.166	up	6.166	2.15E-11	13.783	up	13.783	2.27E-07	17.120	up	17.120	1.82E-11
SAOUHSC_02441	Asp23	alkaline shock protein 23		0.55	-0.84	0.29	0.78	–	1.46	up	1.46	1.000E+00	1.786	down	0.560	6.02E-01	1.225	up	1.225	1.00E+00	1.718	up	1.718	3.32E-03
SAOUHSC_02443	AmaP	hypothetical protein		0.34	-1.38	-0.24	0.17	–	1.26	up	1.26	1.000E+00	2.600	down	0.385	1.56E-06	1.183	down	0.845	1.00E+00	1.122	up	1.122	1.00E+00
SAOUHSC_02460		hypothetical protein		0.53	0.82	0.21	0.26	–	1.44	up	1.44	1.000E+00	1.771	up	1.771	6.32E-04	1.160	up	1.160	1.00E+00	1.199	up	1.199	1.00E+00
SAOUHSC_02519		hypothetical protein		0.05	0.48	1.33	0.94	▲	1.04	up	1.04	1.000E+00	1.396	up	1.396	1.00E+00	2.512	up	2.512	2.36E-02	1.919	up	1.919	2.08E-02
SAOUHSC_02554	FhuD2	hypothetical protein		-1.70	0.21	-0.28	-0.41	–	3.26	down	0.31	1.035E-30	1.157	up	1.157	1.00E+00	1.213	down	0.825	1.00E+00	1.330	down	0.752	1.00E+00
SAOUHSC_02566	SarR	hypothetical protein		-0.76	-0.05	-0.34	-0.25	–	1.69	down	0.59	3.704E-03	1.036	down	0.965	1.00E+00	1.267	down	0.790	1.00E+00	1.187	down	0.843	1.00E+00
SAOUHSC_02568		hypothetical protein		0.87	1.48	1.26	1.64	▲	1.83	up	1.83	1.490E-02	2.786	up	2.786	1.81E-11	2.390	up	2.390	1.19E-06	3.110	up	3.110	4.98E-11
SAOUHSC_02571	SsaA	secretory antigen		-0.53	-0.74	1.81	1.16	▲	1.44	down	0.69	1.000E+00	1.670	down	0.599	1.00E+00	3.514	up	3.514	2.08E-08	2.240	up	2.240	1.88E-05
SAOUHSC_02604		hypothetical protein		0.83	-0.88	0.12	0.38	–	1.78	up	1.78	2.119E-03	1.843	down	0.543	6.61E-01	1.085	up	1.085	1.00E+00	1.301	up	1.301	1.00E+00
SAOUHSC_02618		hypothetical protein		-0.46	-1.42	n.d.	-0.41	▼	1.38	down	0.73	1.000E+00	2.684	down	0.373	1.40E-04					1.327	down	0.754	1.00E+00
SAOUHSC_02619		hypothetical protein		-0.53	-1.17	n.d.	-0.34	▼	1.44	down	0.69	1.000E+00	2.251	down	0.444	1.26E-02					1.263	down	0.792	1.00E+00
SAOUHSC_02626		hypothetical protein		0.56	0.80	0.15	0.24	–	1.47	up	1.47	1.000E+00	1.745	up	1.745	2.64E-02	1.110	up	1.110	1.00E+00	1.178	up	1.178	1.00E+00
SAOUHSC_02650		hypothetical protein		0.59	-0.00	0.77	0.94	▲	1.51	up	1.51	1.000E+00	1.001	down	0.999	1.00E+00	1.709	up	1.709	5.39E-01	1.916	up	1.916	5.23E-05
SAOUHSC_02665		hypothetical protein		0.86	-0.58	0.43	0.44	–	1.82	up	1.82	7.263E-05	1.491	down	0.671	1.00E+00	1.343	up	1.343	1.00E+00	1.356	up	1.356	1.00E+00
SAOUHSC_02667	GltT	hypothetical protein		-0.10	-1.16	-0.05	-0.66	▼	1.07	down	0.93	1.000E+00	2.236	down	0.447	4.34E-02	1.033	down	0.968	1.00E+00	1.582	down	0.632	1.00E+00

locus tag	protein symbol	description	theSEED functional category	log2 ratio OD=0.4	log2 ratio 8h p.i.	log2 ratio 24h p.i.	log2 ratio 32h p.i.	median over 8,24 and 32h ratio; median tendence - 1.5fc cutoff	median fold-change OD=0.4	median fold-change (direction) OD=0.4	median ratio over assays against non-ad. OD=0.4	p-value BH adjusted OD=0.4	median fold-change 8h p.i.	median fold-change (direction) 8h p.i.	median ratio over assays against non-ad. 8h p.i.	p-value BH adjusted 8h p.i.	median fold-change 24h p.i.	median fold-change (direction) 24h p.i.	median ratio over assays against non-ad. 24h p.i.	p-value BH adjusted 24h p.i.	median fold-change 32h p.i.	median fold-change (direction) 32h p.i.	median ratio over assays against non-ad. 32h p.i.	p-value BH adjusted 32h p.i.
SAOUHSC_02668		hypothetical protein		-0.48	-0.39	-1.15	-1.34	▼	1.39	down	0.72	1.000E+00	1.308	down	0.764	1.00E+00	2.221	down	0.450	6.39E-04	2.528	down	0.396	1.47E-06
SAOUHSC_02669	SarZ	hypothetical protein		-0.31	0.79	0.91	0.94	▲	1.24	down	0.80	1.000E+00	1.731	up	1.731	4.10E-02	1.881	up	1.881	2.78E-01	1.916	up	1.916	1.03E-01
SAOUHSC_02690		hypothetical protein		-0.77	-0.87	0.01	-0.57	–	1.71	down	0.59	1.000E+00	1.825	down	0.548	4.98E-03	1.007	up	1.007	1.00E+00	1.481	down	0.675	1.00E+00
SAOUHSC_02700	MdeA	hypothetical protein		-0.31	-1.42	-1.09	-1.23	▼	1.24	down	0.81	1.000E+00	2.667	down	0.375	2.27E-02	2.130	down	0.469	6.87E-01	2.339	down	0.428	4.58E-01
SAOUHSC_02724		hypothetical protein		0.59	2.46	2.09	2.03	▲	1.51	up	1.51	1.000E+00	5.508	up	5.508	1.03E-10	4.250	up	4.250	5.94E-11	4.085	up	4.085	1.07E-09
SAOUHSC_02747		hypothetical protein		0.46	1.22	0.59	0.79	▲	1.37	up	1.37	1.000E+00	2.332	up	2.332	3.64E-09	1.506	up	1.506	1.00E+00	1.733	up	1.733	4.77E-02
SAOUHSC_02759		hypothetical protein		-0.32	0.40	0.52	0.81	–	1.25	down	0.80	1.000E+00	1.317	up	1.317	1.00E+00	1.439	up	1.439	9.88E-01	1.751	up	1.751	2.67E-03
SAOUHSC_02774		hypothetical protein		1.52	-0.61	2.72	0.73	▲	2.86	up	2.86	2.797E-05	1.527	down	0.655	1.00E+00	6.604	up	6.604	4.86E-01	1.658	up	1.658	9.59E-02
SAOUHSC_02811	RelP	hypothetical protein		-0.28	1.10	-0.16	-0.06	–	1.22	down	0.82	1.000E+00	2.147	up	2.147	2.86E-09	1.118	down	0.894	1.00E+00	1.043	down	0.959	1.00E+00
SAOUHSC_02820		hypothetical protein		-0.56	-2.08	-0.99	-0.69	▼	1.47	down	0.68	1.000E+00	4.222	down	0.237	1.23E-07	1.992	down	0.502	8.74E-01	1.610	down	0.621	8.13E-01
SAOUHSC_02827		hypothetical protein		0.92	0.25	0.56	0.68	–	1.90	up	1.90	1.348E-02	1.188	up	1.188	1.00E+00	1.475	up	1.475	1.00E+00	1.597	up	1.597	7.76E-01
SAOUHSC_02829	Frp	NAD(P)H-flavin oxidoreductase		0.45	-0.04	1.41	1.65	▲	1.37	up	1.37	1.000E+00	1.028	down	0.973	1.00E+00	2.657	up	2.657	5.06E-07	3.141	up	3.141	2.10E-12
SAOUHSC_02844		hypothetical protein		0.15	0.66	0.91	0.90	▲	1.11	up	1.11	1.000E+00	1.576	up	1.576	5.03E-02	1.881	up	1.881	5.53E-05	1.868	up	1.868	3.47E-05
SAOUHSC_02846		hypothetical protein		0.22	1.38	0.67	0.53	▲	1.17	up	1.17	1.000E+00	2.598	up	2.598	1.40E-04	1.586	up	1.586	1.00E+00	1.446	up	1.446	1.00E+00
SAOUHSC_02862	ClpL	ATP-dependent Clp protease, ATP-binding subunit ClpC		0.53	1.09	1.15	1.52	▲	1.44	up	1.44	1.000E+00	2.129	up	2.129	1.60E-11	2.225	up	2.225	5.36E-10	2.871	up	2.871	9.66E-23
SAOUHSC_02866		hypothetical protein		0.06	-2.10	n.d.	-0.86	▼	1.05	up	1.05	1.000E+00	4.290	down	0.233	7.59E-07					1.816	down	0.551	7.30E-02
SAOUHSC_02886		hypothetical protein		-0.20	-1.67	n.d.	n.d.	▼	1.15	down	0.87	1.000E+00	3.175	down	0.315	2.59E-04								
SAOUHSC_02887	IsaA	immunodominant antigen A		0.92	0.38	1.32	0.80	▲	1.90	up	1.90	3.320E-03	1.298	up	1.298	1.00E+00	2.490	up	2.490	2.02E-05	1.745	up	1.745	3.62E-03
SAOUHSC_02900		hypothetical protein		0.92	-0.30	0.71	0.92	▲	1.89	up	1.89	6.014E-09	1.234	down	0.810	1.00E+00	1.635	up	1.635	3.28E-01	1.896	up	1.896	9.34E-06
SAOUHSC_02910		hypothetical protein		0.25	1.12	0.87	0.68	▲	1.19	up	1.19	1.000E+00	2.166	up	2.166	2.24E-05	1.831	up	1.831	1.00E+00	1.607	up	1.607	9.69E-01
SAOUHSC_02972	IsaB	immunodominant antigen B		-0.56	2.44	3.68	3.90	▲	1.47	down	0.68	1.000E+00	5.421	up	5.421	4.25E-02	12.840	up	12.840	8.16E-02	14.908	up	14.908	2.12E-02
SAOUHSC_02978		phage infection protein		-0.26	-0.73	-1.03	-0.93	▼	1.20	down	0.84	1.000E+00	1.656	down	0.604	1.21E-01	2.044	down	0.489	2.03E-06	1.909	down	0.524	1.37E-06
SAOUHSC_02980		hypothetical protein		0.39	0.92	0.75	0.83	▲	1.31	up	1.31	1.000E+00	1.891	up	1.891	1.22E-04	1.677	up	1.677	5.50E-02	1.778	up	1.778	3.50E-04
SAOUHSC_03022		hypothetical protein		0.64	-0.69	0.61	0.92	▲	1.56	up	1.56	1.000E+00	1.612	down	0.620	1.00E+00	1.525	up	1.525	1.00E+00	1.895	up	1.895	2.20E-03
SAOUHSC_03024		hypothetical protein		0.32	-0.18	-1.06	-1.55	▼	1.25	up	1.25	1.000E+00	1.134	down	0.882	1.00E+00	2.079	down	0.481	2.10E-02	2.937	down	0.340	2.59E-01
SAOUHSC_03034		hypothetical protein		1.10	1.62	2.45	2.44	▲	2.15	up	2.15	7.437E-03	3.067	up	3.067	4.32E-07	5.453	up	5.453	1.58E-06	5.442	up	5.442	3.62E-09

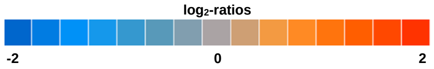

supplemental table S5: significant regulated proteins in comparison of 8h p.i. and S9 infection with S. aureus (cell culture OD=0.4, non-ad., 24h p.i. and 32h p.i.)

The comparison of the early intracellular phase sample (8h p.i.) and late intracellular phase samples (24h and 32h p.i.) revealed proteins that were not significantly altered in their amount by using the non-adherent control as point of reference, because proteins were not detected in the non-adherent control sample or proteins were altered by opposing changes (early phase / late phase).

locus tag	protein symbol	description	theSEED functional category	log2 ratio OD=0.4	log2 ratio non-adherent	log2 ratio 24h p.i.	log2 ratio 32h p.i.	median over 24 and 32h ratio; median tendence - 1.5fc cutoff	median fold-change OD=0.4	median fold-change (direction) OD=0.4	median ratio over assays against 8h p.i. OD=0.4	p-value BH adjusted OD=0.4	median fold-change non-ad.	median fold-change (direction) non-ad.	median ratio over assays against 8h p.i. non-ad.	p-value BH adjusted non-ad.	median fold-change 24h p.i.	median fold-change (direction) 24h p.i.	median ratio over assays against 8h p.i. 24h p.i.	p-value BH adjusted 24h p.i.	median fold-change 32h p.i.	median fold-change (direction) 32h p.i.	median ratio over assays against 8h p.i. 32h p.i.	p-value BH adjusted 32h p.i.
SAOUHSC_00924	Opp-3C	hypothetical protein	ABC transporters	-0.90	-1.53	0.32	0.09	-	1.87	down	0.53	7.646E-02	2.89	down	0.35	5.480E-07	1.25	up	1.25	1.000E+00	1.07	up	1.07	1.000E+00
SAOUHSC_02767	Opp-1A	peptide ABC transporter peptide-binding protein	ABC transporters;Motility and Chemotaxis - no subcategory	-2.09	-0.19	-0.16	0.04	-	4.24	down	0.24	1.400E-06	1.14	down	0.87	1.000E+00	1.12	down	0.89	1.000E+00	1.03	up	1.03	1.000E+00
SAOUHSC_00201	Opp-5A	hypothetical protein	ABC transporters;Regulation and Cell signaling - no subcategory	-1.74	-1.62	n.d.	-1.22	▼	3.35	down	0.30	1.050E-09	3.08	down	0.32	4.030E-07					2.33	down	0.43	3.074E-01
SAOUHSC_00927	Opp-3A	oligopeptide ABC transporter substrate-binding protein	ABC transporters;Regulation and Cell signaling - no subcategory	0.08	-0.33	0.82	0.87	▲	1.06	up	1.06	1.000E+00	1.26	down	0.80	1.000E+00	1.76	up	1.76	5.348E-02	1.83	up	1.83	3.063E-04
SAOUHSC_00069	Spa	protein A	Adhesion	4.96	3.90	1.38	1.41	▲	31.15	up	31.15	2.540E-13	14.94	up	14.94	6.940E-13	2.60	up	2.60	4.987E-03	2.66	up	2.66	5.720E-09
SAOUHSC_00544	SdrC	sdrC protein	Adhesion	-0.07	0.93	n.d.	n.d.	-	1.05	down	0.96	1.000E+00	1.90	up	1.90	4.674E-03								
SAOUHSC_00812	ClfA	clumping factor	Adhesion	1.09	0.58	0.43	0.80	▲	2.13	up	2.13	6.290E-17	1.49	up	1.49	5.871E-01	1.34	up	1.34	1.000E+00	1.74	up	1.74	1.300E-08
SAOUHSC_02161	Eap|map	MHC class II analog protein	Adhesion	n.d.	n.d.	0.95	-0.24	-									1.94	up	1.94	1.719E-04	1.18	down	0.85	1.000E+00
SAOUHSC_02963	ClfB	clumping factor B	Adhesion	0.53	1.78	-1.33	-1.88	▼	1.44	up	1.44	1.000E+00	3.43	up	3.43	2.650E-26	2.51	down	0.40	9.470E-08	3.67	down	0.27	9.390E-06
SAOUHSC_01633	GcvPA	glycine dehydrogenase subunit 1	Alanine, serine, and glycine	-0.82	-0.34	0.17	0.27	-	1.76	down	0.57	3.268E-02	1.27	down	0.79	1.000E+00	1.13	up	1.13	1.000E+00	1.21	up	1.21	1.000E+00
SAOUHSC_01634	GcvT	glycine cleavage system aminomethyltransferase T	Alanine, serine, and glycine	-0.90	-0.27	0.29	0.45	-	1.87	down	0.53	1.628E-04	1.21	down	0.83	1.000E+00	1.23	up	1.23	1.000E+00	1.37	up	1.37	1.000E+00
SAOUHSC_01832		hypothetical protein	Alanine, serine, and glycine	-1.77	-0.52	-0.33	-0.15	-	3.40	down	0.29	6.820E-05	1.43	down	0.70	1.000E+00	1.25	down	0.80	1.000E+00	1.11	down	0.90	1.000E+00
SAOUHSC_00536	IlvE	branched-chain amino acid aminotransferase	Alanine, serine, and glycine;Branched-chain amino acids;Central carbohydrate metabolism	-0.48	-1.27	-0.48	-0.36	-	1.39	down	0.72	1.000E+00	2.42	down	0.41	7.900E-22	1.39	down	0.72	1.000E+00	1.28	down	0.78	1.000E+00
SAOUHSC_00308		hypothetical protein	Alanine, serine, and glycine;Central carbohydrate metabolism;Lipoic acid;Plant-Prokaryote comparative genomics	-1.50	-1.50	-0.34	-0.16	-	2.82	down	0.35	9.990E-08	2.82	down	0.35	1.171E-03	1.27	down	0.79	1.000E+00	1.11	down	0.90	1.000E+00
SAOUHSC_01632	GcvPB	glycine dehydrogenase subunit 2	Alanine, serine, and glycine;Lipoic acid	-0.84	-0.36	0.25	0.42	-	1.79	down	0.56	1.407E-02	1.28	down	0.78	1.000E+00	1.19	up	1.19	1.000E+00	1.34	up	1.34	1.000E+00
SAOUHSC_00535		hypothetical protein	Alanine, serine, and glycine;Lysine, threonine, methionine, and cysteine	-1.02	-0.78	0.64	0.81	▲	2.02	down	0.49	8.810E-05	1.72	down	0.58	3.626E-01	1.56	up	1.56	5.271E-01	1.75	up	1.75	2.090E-04
SAOUHSC_01833	SerA	D-3-phosphoglycerate dehydrogenase	Alanine, serine, and glycine;Pyridoxine;Miscellaneous - no subcategory	-1.37	-1.22	0.61	0.37	-	2.59	down	0.39	4.370E-11	2.33	down	0.43	7.130E-09	1.53	up	1.53	4.633E-01	1.29	up	1.29	1.000E+00
SAOUHSC_00552	NagB	hypothetical protein	Aminosugars;One-carbon Metabolism;Capsular and extracellular polysacchrides;Cell Wall and Capsule - no subcategory	-1.01	-1.43	-0.44	-0.64	-	2.01	down	0.50	6.300E-09	2.69	down	0.37	4.200E-11	1.36	down	0.74	1.000E+00	1.56	down	0.64	1.000E+00
SAOUHSC_01046	PotA	ABC transporter	Arginine; urea cycle, polyamines	0.51	1.57	0.62	0.84	▲	1.42	up	1.42	1.000E+00	2.97	up	2.97	4.700E-10	1.54	up	1.54	5.207E-01	1.79	up	1.79	8.540E-05
SAOUHSC_01049	PotD	spermidine/putrescine ABC transporter spermidine/putrescine-binding protein	Arginine; urea cycle, polyamines	0.65	1.74	n.d.	0.98	▲	1.57	up	1.57	1.000E+00	3.34	up	3.34	1.860E-05					1.97	up	1.97	1.000E+00
SAOUHSC_00076	SbnB	ornithine cyclodeaminase	Arginine; urea cycle, polyamines;Lysine, threonine, methionine, and cysteine;Proline and 4-hydroxyproline;Siderophores	-5.29	-2.02	0.35	0.07	-	39.08	down	0.03	2.895E-01	4.05	down	0.25	2.510E-09	1.27	up	1.27	1.000E+00	1.05	up	1.05	1.000E+00
SAOUHSC_00894	RocD	ornithine--oxo-acid transaminase	Arginine; urea cycle, polyamines;Proline and 4-hydroxyproline	-1.07	-1.59	0.96	1.24	▲	2.10	down	0.48	2.900E-21	3.00	down	0.33	1.510E-23	1.95	up	1.95	6.200E-14	2.36	up	2.36	4.050E-34
SAOUHSC_02869	RocA	1-pyrroline-5-carboxylate dehydrogenase	Arginine; urea cycle, polyamines;Proline and 4-hydroxyproline	-3.43	-3.89	1.25	1.50	▲	10.79	down	0.09	2.390E-18	14.79	down	0.07	1.390E-12	2.39	up	2.39	3.930E-41	2.83	up	2.83	3.670E-57
SAOUHSC_02409	RocF	arginase	Arginine; urea cycle, polyamines;Proline and 4-hydroxyproline;Plant-Prokaryote comparative genomics	-1.06	-0.89	1.03	0.95	▲	2.09	down	0.48	1.679E-03	1.85	down	0.54	9.251E-02	2.04	up	2.04	7.160E-03	1.94	up	1.94	7.060E-08
SAOUHSC_00733	HisC	histidinol-phosphate aminotransferase	Aromatic amino acids and derivatives	-1.01	-1.41	0.22	0.22	-	2.02	down	0.50	5.986E-04	2.65	down	0.38	3.052E-03	1.17	up	1.17	1.000E+00	1.17	up	1.17	1.000E+00
SAOUHSC_02343	AtpG	F0F1 ATP synthase subunit gamma	ATP synthases	-0.67	-0.17	-0.54	-0.42	-	1.59	down	0.63	4.331E-02	1.12	down	0.89	1.000E+00	1.46	down	0.69	1.000E+00	1.33	down	0.75	1.000E+00
SAOUHSC_00144	AusA	hypothetical protein	Biotin	-2.64	-1.85	0.34	0.38	-	6.25	down	0.16	2.980E-12	3.60	down	0.28	3.200E-18	1.26	up	1.26	1.000E+00	1.30	up	1.30	1.000E+00
SAOUHSC_02713	BioF	hypothetical protein	Biotin	-0.09	-1.32	-1.46	-1.16	▼	1.07	down	0.94	1.000E+00	2.49	down	0.40	3.040E-07	2.76	down	0.36	6.720E-02	2.23	down	0.45	1.808E-03
SAOUHSC_02714	BioB	biotin synthase	Biotin	0.83	-0.57	-2.86	-2.95	▼	1.78	up	1.78	1.900E-06	1.49	down	0.67	1.000E+00	7.25	down	0.14	1.680E-05	7.74	down	0.13	9.390E-06
SAOUHSC_02715	BioA	adenosylmethionine-8-amino-7-oxononanoate aminotransferase	Biotin	0.08	-0.89	-0.38	-0.63	-	1.06	up	1.06	1.000E+00	1.85	down	0.54	6.609E-03	1.31	down	0.77	1.000E+00	1.55	down	0.65	1.000E+00
SAOUHSC_02716	BioD	dethiobiotin synthase	Biotin	0.51	-0.72	-0.84	-1.10	▼	1.42	up	1.42	1.000E+00	1.65	down	0.61	9.487E-01	1.79	down	0.56	1.053E-03	2.15	down	0.47	1.200E-11
SAOUHSC_02284	IlvC	ketol-acid reductoisomerase	Branched-chain amino acids;Coenzyme A	-0.77	-1.18	0.88	0.87	▲	1.71	down	0.58	7.483E-01	2.27	down	0.44	8.966E-04	1.83	up	1.83	5.350E-05	1.83	up	1.83	2.040E-07
SAOUHSC_02282	IlvB	acetolactate synthase large subunit	Branched-chain amino acids;Fermentation	-1.61	-2.35	0.34	0.56	-	3.05	down	0.33	3.480E-05	5.11	down	0.20	1.179E-02	1.26	up	1.26	1.000E+00	1.47	up	1.47	1.000E+00
SAOUHSC_00196	FadB	hypothetical protein	Branched-chain amino acids;Fermentation;Fatty Acids, Lipids, and Isoprenoids - no subcategory	n.d.	n.d.	1.73	2.03	▲									3.31	up	3.31	2.590E-63	4.08	up	4.08	5.900E-90
SAOUHSC_00336	Thl	acetyl-CoA acetyltransferase	Branched-chain amino acids;Fermentation;One-carbon Metabolism;Biotin;Fatty Acids, Lipids, and Isoprenoids - no subcategory;Isoprenoids	-1.00	-0.81	-1.01	-1.00	▼	1.99	down	0.50	7.540E-17	1.76	down	0.57	2.035E-03	2.01	down	0.50	1.380E-09	1.99	down	0.50	1.430E-11
SAOUHSC_00195	FadA	acetyl-CoA acetyltransferase	Branched-chain amino acids;One-carbon Metabolism;Biotin;Fatty Acids, Lipids, and Isoprenoids - no subcategory	n.d.	n.d.	1.61	1.72	▲									3.06	up	3.06	1.720E-12	3.30	up	3.30	4.790E-20
SAOUHSC_00158		PTS system protein	Capsular and extracellular polysacchrides	n.d.	n.d.	1.45	1.80	▲									2.73	up	2.73	1.300E-08	3.49	up	3.49	6.960E-13
SAOUHSC_01204	Smc	SMC domain-containing protein	Cell Division and Cell Cycle - no subcategory	0.01	0.66	-0.08	0.10	-	1.01	up	1.01	1.000E+00	1.58	up	1.58	1.969E-04	1.06	down	0.95	1.000E+00	1.07	up	1.07	1.000E+00
SAOUHSC_01827	EzrA	septation ring formation regulator EzrA	Cell Division and Cell Cycle - no subcategory	0.43	0.63	0.47	0.60	-	1.34	up	1.34	1.000E+00	1.54	up	1.54	3.556E-02	1.38	up	1.38	1.000E+00	1.51	up	1.51	1.589E-01
SAOUHSC_01652	Pbp3	penicillin-binding protein 3	Cell Division and Cell Cycle - no subcategory;Cell Wall and Capsule - no subcategory	-0.26	-0.00	-0.68	-0.77	▼	1.20	down	0.83	1.000E+00	1.00	down	1.00	1.000E+00	1.60	down	0.62	6.517E-01	1.70	down	0.59	4.007E-03
SAOUHSC_02012	SgtB	glycosyltransferase	Cell Wall and Capsule - no subcategory	-2.49	-2.06	-0.63	-1.00	▼	5.61	down	0.18	3.430E-14	4.17	down	0.24	9.520E-18	1.55	down	0.64	1.000E+00	2.01	down	0.50	5.110E-08
SAOUHSC_02365	MurA2	UDP-N-acetylglucosamine 1-carboxyvinyltransferase	Cell Wall and Capsule - no subcategory	-1.33	-1.27	-0.93	-0.81	▼	2.52	down	0.40	3.460E-20	2.40	down	0.42	2.580E-12	1.90	down	0.53	2.514E-03	1.75	down	0.57	5.320E-07
SAOUHSC_00994	Atl	bifunctional autolysin	Cell Wall and Capsule - no subcategory;Quorum sensing and biofilm formation;Regulation and Cell signaling - no subcategory;Adhesion	1.96	1.29	-0.51	-0.96	▼	3.88	up	3.88	2.590E-96	2.44	up	2.44	3.840E-61	1.42	down	0.70	1.000E+00	1.94	down	0.51	9.970E-06
SAOUHSC_00153		indolepyruvate decarboxylase	Central carbohydrate metabolism	0.81	0.46	1.13	1.38	▲	1.76	up	1.76	1.125E-03	1.38	up	1.38	9.618E-01	2.18	up	2.18	3.840E-09	2.60	up	2.60	1.250E-15
SAOUHSC_00655		dihydroxyacetone kinase subunit DhaK	Central carbohydrate metabolism	-2.02	-2.90	0.95	1.46	▲	4.06	down	0.25	6.170E-07	7.47	down	0.13	5.370E-08	1.93	up	1.93	1.469E-04	2.75	up	2.75	5.700E-10
SAOUHSC_00656		hypothetical protein	Central carbohydrate metabolism	-1.10	-1.84	1.21	1.21	▲	2.14	down	0.47	9.488E-01	3.58	down	0.28	9.459E-01	2.32	up	2.32	1.680E-05	2.31	up	2.31	8.250E-12
SAOUHSC_00658		phosphotransferase mannnose-specific family component IIA	Central carbohydrate metabolism	n.d.	-1.86	0.38	0.98	▲					3.62	down	0.28	5.235E-01	1.30	up	1.30	1.000E+00	1.97	up	1.97	5.722E-04
SAOUHSC_00797	TpiA	triosephosphate isomerase	Central carbohydrate metabolism	0.66	-0.05	-0.44	-0.41	-	1.58	up	1.58	9.470E-05	1.04	down	0.97	1.000E+00	1.36	down	0.74	1.000E+00	1.32	down	0.75	1.000E+00
SAOUHSC_01418	SucA	2-oxoglutarate dehydrogenase E1 component	Central carbohydrate metabolism	-0.76	-0.45	0.82	1.08	▲	1.69	down	0.59	8.457E-04	1.37	down	0.73	1.000E+00	1.76	up	1.76	4.250E-09	2.12	up	2.12	9.800E-38
SAOUHSC_01710		acetyl-CoA carboxylase, biotin carboxyl carrier protein	Central carbohydrate metabolism	0.41	-0.08	0.61	0.92	▲	1.33	up	1.33	1.000E+00	1.06	down	0.95	1.000E+00	1.53	up	1.53	1.000E+00	1.89	up	1.89	2.180E-02
SAOUHSC_01801	CitC	isocitrate dehydrogenase	Central carbohydrate metabolism	-0.56	-0.77	1.27	1.67	▲	1.47	down	0.68	1.000E+00	1.70	down	0.59	1.000E+00	2.42	up	2.42	5.510E-21	3.18	up	3.18	1.070E-31
SAOUHSC_01818	Ald2	alanine dehydrogenase	Central carbohydrate metabolism	-0.26	-0.36	0.77	1.05	▲	1.20	down	0.84	1.000E+00	1.29	down	0.78	1.000E+00	1.71	up	1.71	2.910E-06	2.07	up	2.07	9.440E-26
SAOUHSC_01846	AcsA	acetyl-CoA synthetase	Central carbohydrate metabolism	-4.72	n.d.	1.17	1.37	▲	26.30	down	0.04	4.280E-02					2.26	up	2.26	9.230E-15	2.59	up	2.59	5.260E-25
SAOUHSC_01867	Dat	D-alanine aminotransferase	Central carbohydrate metabolism	-0.57	-0.90	-0.51	-0.56	-	1.49	down	0.67	1.000E+00	1.87	down	0.54	4.576E-03	1.43	down	0.70	1.000E+00	1.47	down	0.68	1.000E+00
SAOUHSC_01983	FumC	fumarate hydratase	Central carbohydrate metabolism	-0.50	-0.77	0.62	0.76	▲	1.41	down	0.71	1.000E+00	1.70	down	0.59	1.000E+00	1.54	up	1.54	4.306E-01	1.69	up	1.69	3.030E-07
SAOUHSC_02647	Mqo1	malate:quinone oxidoreductase	Central carbohydrate metabolism	-0.33	-0.16	0.89	0.89	▲	1.26	down	0.79	1.000E+00	1.12	down	0.90	1.000E+00	1.86	up	1.86	6.529E-04	1.86	up	1.86	1.640E-08

The comparison of the early intracellular phase sample (8h p.i.) and late intracellular phase samples (24h and 32h p.i.) revealed proteins that were not significantly altered in their amount by using the non-adherent control as point of reference, because proteins were not detected in the non-adherent control sample or proteins were altered by opposing changes (early phase / late phase).

locus tag	protein symbol	description	theSEED functional category	log2 ratio OD=0.4	log2 ratio non-adherent	log2 ratio 24h p.i.	log2 ratio 32h p.i.	median over 24 and 32h ratio; median tendence - 1.5fc cutoff	median fold-change OD=0.4	median fold-change (direction) OD=0.4	median ratio over assays against 8h p.i. OD=0.4	p-value BH adjusted OD=0.4	median fold-change non-ad.	median fold-change (direction) non-ad.	median ratio over assays against 8h p.i. non-ad.	p-value BH adjusted non-ad.	median fold-change 24h p.i.	median fold-change (direction) 24h p.i.	median ratio over assays against 8h p.i. 24h p.i.	p-value BH adjusted 24h p.i.	median fold-change 32h p.i.	median fold-change (direction) 32h p.i.	median ratio over assays against 8h p.i. 32h p.i.	p-value BH adjusted 32h p.i.
SAOUHSC_01820	AckA	acetate kinase	Central carbohydrate metabolism;Fermentation	0.09	0.60	-0.17	-0.03	-	1.07	up	1.07	1.000E+00	1.51	up	1.51	3.114E-02	1.12	down	0.89	1.000E+00	1.02	down	0.98	1.000E+00
SAOUHSC_01416	SucB	dihydrolipoamide succinyltransferase	Central carbohydrate metabolism;Lipoic acid	-1.09	-0.64	0.94	1.20	▲	2.13	down	0.47	1.130E-08	1.56	down	0.64	1.000E+00	1.92	up	1.92	5.830E-10	2.30	up	2.30	5.420E-19
SAOUHSC_01807	PfkA	6-phosphofructokinase	Central carbohydrate metabolism;Monosaccharides	-0.37	-0.59	-0.67	-0.74	▼	1.29	down	0.78	1.000E+00	1.51	down	0.66	1.000E+00	1.60	down	0.63	2.397E-02	1.67	down	0.60	1.379E-02
SAOUHSC_00900	Pgi	glucose-6-phosphate isomerase	Central carbohydrate metabolism;One-carbon Metabolism	-0.85	-0.80	-0.78	-0.72	▼	1.81	down	0.55	1.167E-04	1.74	down	0.58	3.871E-01	1.71	down	0.58	9.680E-05	1.64	down	0.61	8.378E-02
SAOUHSC_01216	SucC	succinyl-CoA synthetase subunit beta	Central carbohydrate metabolism;One-carbon Metabolism	-0.20	-0.13	0.84	0.98	▲	1.14	down	0.87	1.000E+00	1.10	down	0.91	1.000E+00	1.79	up	1.79	2.020E-09	1.97	up	1.97	8.540E-28
SAOUHSC_01218	SucD	succinyl-CoA synthetase subunit alpha	Central carbohydrate metabolism;One-carbon Metabolism	-0.13	-0.20	0.76	1.11	▲	1.09	down	0.91	1.000E+00	1.15	down	0.87	1.000E+00	1.69	up	1.69	1.090E-05	2.16	up	2.16	6.370E-18
SAOUHSC_01910	PckA	phosphoenolpyruvate carboxykinase	Central carbohydrate metabolism;One-carbon Metabolism	-2.10	-2.45	1.37	1.72	▲	4.28	down	0.23	2.140E-05	5.47	down	0.18	7.070E-07	2.59	up	2.59	7.840E-20	3.29	up	3.29	1.170E-32
SAOUHSC_01104	SdhA	succinate dehydrogenase flavoprotein subunit	Central carbohydrate metabolism;One-carbon Metabolism;Electron donating reactions	0.13	-0.10	0.97	1.24	▲	1.10	up	1.10	1.000E+00	1.07	down	0.94	1.000E+00	1.96	up	1.96	6.200E-14	2.36	up	2.36	4.630E-25
SAOUHSC_01105	SdhB	succinate dehydrogenase iron-sulfur subunit	Central carbohydrate metabolism;One-carbon Metabolism;Electron donating reactions	0.37	0.34	0.72	0.94	▲	1.29	up	1.29	1.000E+00	1.27	up	1.27	1.000E+00	1.64	up	1.64	5.080E-03	1.92	up	1.92	1.760E-12
SAOUHSC_01347	CitB	aconitate hydratase	Central carbohydrate metabolism;One-carbon Metabolism;Respiration - no subcategory	1.16	0.31	0.64	0.97	▲	2.24	up	2.24	5.930E-60	1.24	up	1.24	1.000E+00	1.56	up	1.56	3.913E-02	1.96	up	1.96	1.260E-53
SAOUHSC_02577		D-isomer specific 2-hydroxyacid dehydrogenase NAD binding domain-containing protein	Central carbohydrate metabolism;Organic acids	-0.78	-1.12	0.92	1.00	▲	1.72	down	0.58	1.338E-03	2.18	down	0.46	1.690E-15	1.89	up	1.89	1.060E-17	2.01	up	2.01	2.590E-27
SAOUHSC_01794	GapB	glyceraldehyde 3-phosphate dehydrogenase 2	Central carbohydrate metabolism;Oxidative stress	-1.27	-0.75	-0.38	0.71	-	2.40	down	0.42	9.741E-04	1.68	down	0.60	5.139E-01	1.30	down	0.77	1.000E+00	1.63	up	1.63	5.467E-01
SAOUHSC_00132	AldA	aldehyde dehydrogenase	Central carbohydrate metabolism;Phospholipids	-1.53	n.d.	1.14	1.42	▲	2.89	down	0.35	4.280E-02					2.20	up	2.20	2.580E-07	2.68	up	2.68	1.450E-12
SAOUHSC_02142	AldH	aldehyde dehydrogenase	Central carbohydrate metabolism;Phospholipids	-0.64	-0.67	0.52	0.71	▲	1.55	down	0.64	1.000E+00	1.59	down	0.63	1.000E+00	1.43	up	1.43	1.000E+00	1.64	up	1.64	9.920E-07
SAOUHSC_02363		aldehyde dehydrogenase	Central carbohydrate metabolism;Phospholipids	0.82	-0.01	0.60	0.72	▲	1.77	up	1.77	5.030E-08	1.01	down	0.99	1.000E+00	1.52	up	1.52	2.349E-01	1.65	up	1.65	3.850E-07
SAOUHSC_01038	Def	peptide deformylase	Central carbohydrate metabolism;Plant-Prokaryote comparative genomics;Protein biosynthesis	0.77	0.04	-0.28	-0.07	-	1.70	up	1.70	6.178E-04	1.03	up	1.03	1.000E+00	1.21	down	0.82	1.000E+00	1.05	down	0.95	1.000E+00
SAOUHSC_02849	CidC	pyruvate oxidase	Central carbohydrate metabolism;Programmed Cell Death and Toxin-antitoxin Systems	1.18	1.07	0.53	0.71	▲	2.26	up	2.26	8.420E-25	2.09	up	2.09	1.110E-16	1.44	up	1.44	1.000E+00	1.64	up	1.64	4.533E-04
SAOUHSC_00879	AmpA	cytosol aminopeptidase	Central carbohydrate metabolism;Protein degradation	-0.97	0.34	0.59	0.71	▲	1.95	down	0.51	4.429E-03	1.26	up	1.26	1.000E+00	1.51	up	1.51	1.000E+00	1.64	up	1.64	1.870E-01
SAOUHSC_02918	PanC	pantoate--beta-alanine ligase	Coenzyme A	-0.01	0.42	-0.89	-0.92	▼	1.00	down	1.00	1.000E+00	1.34	up	1.34	1.000E+00	1.86	down	0.54	1.000E+00	1.89	down	0.53	3.056E-03
SAOUHSC_02919	PanB	3-methyl-2-oxobutanoate hydroxymethyltransferase	Coenzyme A;Folate and pterines	-0.50	-0.27	-0.81	-0.94	▼	1.41	down	0.71	1.000E+00	1.21	down	0.83	1.000E+00	1.76	down	0.57	2.670E-03	1.92	down	0.52	5.792E-04
SAOUHSC_02329	ThiM	hydroxyethylthiazole kinase	Cofactors, Vitamins, Prosthetic Groups, Pigments - no subcategory	-2.09	-2.72	-0.29	-0.21	-	4.25	down	0.24	9.310E-06	6.58	down	0.15	2.030E-07	1.22	down	0.82	1.000E+00	1.16	down	0.86	1.000E+00
SAOUHSC_02330	ThiD2	phosphomethylpyrimidine kinase	Cofactors, Vitamins, Prosthetic Groups, Pigments - no subcategory	-1.75	-1.40	-0.03	-0.11	-	3.37	down	0.30	1.690E-03	2.65	down	0.38	3.624E-03	1.02	down	0.98	1.000E+00	1.08	down	0.93	1.000E+00
SAOUHSC_02331	TenA	hypothetical protein	Cofactors, Vitamins, Prosthetic Groups, Pigments - no subcategory	-1.79	-2.13	-0.27	-0.06	-	3.46	down	0.29	1.830E-05	4.36	down	0.23	2.430E-02	1.21	down	0.83	1.000E+00	1.04	down	0.96	1.000E+00
SAOUHSC_00819	CspC	hypothetical protein	Cold shock	-0.73	-0.05	-1.32	-1.41	▼	1.65	down	0.60	5.407E-01	1.04	down	0.97	1.000E+00	2.49	down	0.40	3.800E-09	2.65	down	0.38	1.040E-14
SAOUHSC_03045	CspB	cold shock protein	Cold shock	-0.19	0.20	-2.05	-2.19	▼	1.14	down	0.88	1.000E+00	1.15	up	1.15	1.000E+00	4.15	down	0.24	2.971E-03	4.57	down	0.22	3.000E-05
SAOUHSC_01430	Crr	phosphotransferase system enzyme IIA	Di- and oligosaccharides	-1.74	-2.32	-0.82	-0.87	▼	3.35	down	0.30	6.770E-21	4.98	down	0.20	9.590E-22	1.77	down	0.57	3.926E-03	1.82	down	0.55	5.040E-05
SAOUHSC_00162	HsdR	HsdR family type I site-specific deoxyribonuclease	DNA Metabolism - no subcategory	0.23	0.75	n.d.	0.51	-	1.17	up	1.17	1.000E+00	1.68	up	1.68	1.554E-02					1.42	up	1.42	1.000E+00
SAOUHSC_00349	Ssb	bacteriophage L54a, single-stranded DNA binding protein	DNA repair	0.62	0.87	-0.02	0.34	-	1.53	up	1.53	3.012E-01	1.83	up	1.83	2.598E-04	1.01	down	0.99	1.000E+00	1.26	up	1.26	1.000E+00
SAOUHSC_01768	Tag	hypothetical protein	DNA repair	0.27	-0.29	-0.99	-0.42	▼	1.21	up	1.21	1.000E+00	1.22	down	0.82	1.000E+00	1.99	down	0.50	3.633E-02	1.34	down	0.75	1.000E+00
SAOUHSC_01241	PolC	DNA polymerase III PolC	DNA replication	-0.40	0.01	-0.69	-0.80	▼	1.32	down	0.76	1.000E+00	1.01	up	1.01	1.000E+00	1.61	down	0.62	5.703E-01	1.74	down	0.57	1.450E-02
SAOUHSC_01351	ParE	DNA topoisomerase IV subunit B	DNA replication;Resistance to antibiotics and toxic compounds	0.34	0.74	-0.16	-0.11	-	1.27	up	1.27	1.000E+00	1.67	up	1.67	3.100E-07	1.12	down	0.90	1.000E+00	1.08	down	0.92	1.000E+00
SAOUHSC_00935	TrfA	adaptor protein	DNA uptake, competence	1.38	1.00	n.d.	n.d.	-	2.60	up	2.60	5.640E-11	2.00	up	2.00	5.070E-06								
SAOUHSC_02517	TopB	DNA topoisomerase III	DNA uptake, competence	0.83	1.04	-0.36	-0.26	-	1.78	up	1.78	6.270E-08	2.06	up	2.06	1.550E-12	1.29	down	0.78	1.000E+00	1.20	down	0.84	1.000E+00
SAOUHSC_00469	SpoVG	regulatory protein SpoVG	Dormancy and Sporulation - no subcategory	1.02	0.80	0.21	0.83	-	2.03	up	2.03	1.950E-05	1.74	up	1.74	1.340E-05	1.15	up	1.15	1.000E+00	1.78	up	1.78	6.970E-05
SAOUHSC_01031	CydA	cytochrome d ubiquinol oxidase subunit I	Electron accepting reactions	-2.84	-0.88	-0.49	-0.65	-	7.15	down	0.14	9.370E-09	1.84	down	0.54	3.556E-02	1.40	down	0.71	1.000E+00	1.57	down	0.64	1.000E+00
SAOUHSC_01032	CydB	cytochrome d ubiquinol oxidase subunit II	Electron accepting reactions	-2.72	-1.23	-0.39	-0.68	-	6.57	down	0.15	7.290E-07	2.34	down	0.43	3.340E-06	1.31	down	0.76	1.000E+00	1.60	down	0.63	2.947E-01
SAOUHSC_00835		hypothetical protein	Electron accepting reactions;Resistance to antibiotics and toxic compounds	-0.80	-1.41	-0.42	-0.16	-	1.74	down	0.58	1.880E-03	2.66	down	0.38	3.540E-08	1.34	down	0.75	1.000E+00	1.12	down	0.90	1.000E+00
SAOUHSC_02873	CopA	cation transporter E1-E2 family ATPase	Electron accepting reactions;Resistance to antibiotics and toxic compounds	-1.52	-1.42	-0.37	-0.33	-	2.87	down	0.35	3.000E-14	2.68	down	0.37	1.720E-11	1.30	down	0.77	1.000E+00	1.26	down	0.80	1.000E+00
SAOUHSC_00875		hypothetical protein	Electron donating reactions	0.68	0.44	0.33	0.52	-	1.60	up	1.60	5.831E-04	1.36	up	1.36	1.000E+00	1.26	up	1.26	1.000E+00	1.43	up	1.43	1.000E+00
SAOUHSC_00920	FabH	3-oxoacyl-(acyl carrier protein) synthase III	Fatty acids	-0.57	0.86	-0.12	0.10	-	1.49	down	0.67	1.000E+00	1.81	up	1.81	2.470E-09	1.09	down	0.92	1.000E+00	1.07	up	1.07	1.000E+00
SAOUHSC_01624	AccB	acetyl-CoA carboxylase, biotin carboxyl carrier protein	Fatty acids;Miscellaneous - no subcategory	-0.11	0.17	-0.84	-0.55	▼	1.08	down	0.93	1.000E+00	1.13	up	1.13	1.000E+00	1.80	down	0.56	1.453E-03	1.47	down	0.68	1.000E+00
SAOUHSC_00086	ButA	acetoin reductase	Fermentation	-1.45	-2.29	0.93	1.16	▲	2.73	down	0.37	2.890E-12	4.90	down	0.20	1.710E-15	1.90	up	1.90	1.760E-07	2.24	up	2.24	7.200E-20
SAOUHSC_00206	Ldh1	L-lactate dehydrogenase	Fermentation	-0.92	0.25	-1.68	-2.27	▼	1.90	down	0.53	1.010E-07	1.19	up	1.19	1.000E+00	3.20	down	0.31	2.960E-12	4.82	down	0.21	4.883E-04
SAOUHSC_00608	Adh	alcohol dehydrogenase	Fermentation;Phospholipids	-2.04	-0.89	-1.68	-1.89	▼	4.11	down	0.24	3.950E-11	1.86	down	0.54	1.389E-03	3.20	down	0.31	3.633E-02	3.70	down	0.27	3.074E-01
SAOUHSC_00187	PflB	formate acetyltransferase	Fermentation;Respiration - no subcategory	-4.43	-2.07	-0.89	-1.29	▼	21.59	down	0.05	2.237E-02	4.20	down	0.24	2.690E-17	1.86	down	0.54	2.315E-02	2.45	down	0.41	1.050E-09
SAOUHSC_02549	ModA	molybdenum ABC transporter periplasmic molybdate-binding protein	Folate and pterines	0.13	0.40	0.58	0.78	▲	1.09	up	1.09	1.000E+00	1.32	up	1.32	1.000E+00	1.50	up	1.50	1.000E+00	1.71	up	1.71	1.901E-03
SAOUHSC_01435	ThyA	thymidylate synthase	Folate and pterines;Pyrimidines	0.89	0.24	0.10	0.10	-	1.85	up	1.85	4.556E-02	1.18	up	1.18	1.000E+00	1.07	up	1.07	1.000E+00	1.07	up	1.07	1.000E+00
SAOUHSC_01887	RibA	riboflavin biosynthesis protein	Folate and pterines;Riboflavin, FMN, FAD	0.78	0.14	-0.12	-0.12	-	1.71	up	1.71	4.980E-02	1.10	up	1.10	1.000E+00	1.08	down	0.92	1.000E+00	1.09	down	0.92	1.000E+00
SAOUHSC_00549	FolE2	putative GTP cyclohydrolase	Folate and pterines;RNA processing and modification	0.82	0.27	-0.18	0.25	-	1.77	up	1.77	9.002E-04	1.21	up	1.21	1.000E+00	1.13	down	0.88	1.000E+00	1.19	up	1.19	1.000E+00
SAOUHSC_00895	GudB	glutamate dehydrogenase, NAD-specific	Glutamine, glutamate, aspartate, asparagine; ammonia assimilation	-1.41	-1.13	0.57	0.89	▲	2.65	down	0.38	6.210E-21	2.18	down	0.46	1.740E-14	1.49	up	1.49	2.754E-01	1.85	up	1.85	7.750E-24
SAOUHSC_01287	GlnA	glutamine synthetase, type I	Glutamine, glutamate, aspartate, asparagine; ammonia assimilation;Cell Wall and Capsule - no subcategory;Nitrogen Metabolism - no subcategory	0.78	0.02	-0.05	0.15	-	1.72	up	1.72	4.140E-06	1.01	up	1.01	1.000E+00	1.03	down	0.97	1.000E+00	1.11	up	1.11	1.000E+00
SAOUHSC_00640	TagA	teichoic acid biosynthesis protein	Gram-Positive cell wall components	-1.60	-0.02	-0.21	-0.11	-	3.02	down	0.33	7.963E-04	1.02	down	0.98	1.000E+00	1.16	down	0.86	1.000E+00	1.08	down	0.93	1.000E+00
SAOUHSC_00644	TagX	tagX protein	Gram-Positive cell wall components	-0.97	-0.37	-0.05	-0.37	-	1.96	down	0.51	4.435E-03	1.30	down	0.77	1.000E+00	1.03	down	0.97	1.000E+00	1.30	down	0.77	1.000E+00
SAOUHSC_00973	TarM	hypothetical protein	Gram-Positive cell wall components	1.23	1.55	0.70	0.74	▲	2.35	up	2.35	1.146E-03	2.92	up	2.92	3.410E-06	1.63	up	1.63	5.759E-01	1.66	up	1.66	2.613E-02
SAOUHSC_00974		glycosyl transferase, group 1	Gram-Positive cell wall components	1.12	1.15	0.88	0.80	▲	2.17	up	2.17	3.230E-06	2.22	up	2.22	8.950E-09	1.84	up	1.84	2.754E-01	1.74	up	1.74	9.988E-02
SAOUHSC_02649		hypothetical protein	Gram-Positive cell wall components	-1.09	-1.26	-0.04	0.26	-	2.13	down	0.47	1.282E-04	2.40	down	0.42	1.500E-05	1.03	down	0.97	1.000E+00	1.20	up	1.20	1.000E+00
SAOUHSC_01081	IsdA	hypothetical protein	Gram-Positive cell wall components;Iron acquisition and metabolism - no subcategory	-2.53	-0.43	-1.77	-2.23	▼	5.76	down	0.17	2.980E-12	1.35	down	0.74	1.000E+00	3.41	down	0.29	1.260E-06	4.70	down	0.21	6.500E-09
SAOUHSC_00869	DltA	D-alanine--poly(phosphoribitol) ligase subunit 1	Gram-Positive cell wall components;Resistance to antibiotics and toxic compounds	0.93	0.58	0.06	0.36	-	1.91	up	1.91	2.810E-15	1.50	up	1.50	1.762E-01	1.04	up	1.04	1.000E+00	1.28	up	1.28	1.000E+00
SAOUHSC_00872	DltD	extramembranal protein	Gram-Positive cell wall components;Resistance to antibiotics and toxic compounds	0.56	1.20	0.59	0.77	▲	1.48	up	1.48	1.000E+00	2.29	up	2.29	8.780E-17	1.50	up	1.50	1.256E-01	1.70	up	1.70	9.390E-06

2

The comparison of the early intracellular phase sample (8h p.i.) and late intracellular phase samples (24h and 32h p.i.) revealed proteins that were not significantly altered in their amount by using the non-adherent control as point of reference, because proteins were not detected in the non-adherent control sample or proteins were altered by opposing changes (early phase / late phase).

locus tag	protein symbol	description	theSEED functional category	log2 ratio OD=0.4	log2 ratio non-adherent	log2 ratio 24h p.i.	log2 ratio 32h p.i.	median over 24 and 32h ratio; median tendence - 1.5fc cutoff	median fold-change OD=0.4	median fold-change (direction) OD=0.4	median ratio over assays against 8h p.i. OD=0.4	p-value BH adjusted OD=0.4	median fold-change non-ad.	median fold-change (direction) non-ad.	median ratio over assays against 8h p.i. non-ad.	p-value BH adjusted non-ad.	median fold-change 24h p.i.	median fold-change (direction) 24h p.i.	median ratio over assays against 8h p.i. 24h p.i.	p-value BH adjusted 24h p.i.	median fold-change 32h p.i.	median fold-change (direction) 32h p.i.	median ratio over assays against 8h p.i. 32h p.i.	p-value BH adjusted 32h p.i.
SAOUHSC_00130	IsdI	heme-degrading monooxygenase IsdI	Iron acquisition and metabolism - no subcategory	-1.54	-1.48	0.67	0.99	▲	2.91	down	0.34	4.110E-05	2.78	down	0.36	2.460E-07	1.59	up	1.59	6.925E-01	1.99	up	1.99	6.530E-10
SAOUHSC_00749	SstD	hypothetical protein	Iron acquisition and metabolism - no subcategory	-1.95	0.27	-0.88	-0.98	▼	3.87	down	0.26	5.650E-14	1.20	up	1.20	1.000E+00	1.84	down	0.54	2.052E-02	1.98	down	0.51	7.760E-06
SAOUHSC_02434		hypothetical protein	Iron acquisition and metabolism - no subcategory	-2.32	-1.57	-0.03	-0.37	-	4.98	down	0.20	2.070E-09	2.96	down	0.34	2.400E-09	1.02	down	0.98	1.000E+00	1.29	down	0.77	1.000E+00
SAOUHSC_02436		hypothetical protein	Iron acquisition and metabolism - no subcategory	-1.48	-1.02	-0.30	-0.45	-	2.79	down	0.36	5.160E-15	2.03	down	0.49	1.830E-07	1.23	down	0.81	1.000E+00	1.37	down	0.73	1.000E+00
SAOUHSC_02430	HtsA	ABC transporter periplasmic binding protein	Iron acquisition and metabolism - no subcategory;Stress Response - no subcategory	-1.81	0.05	0.15	0.28	-	3.52	down	0.28	2.020E-10	1.04	up	1.04	1.000E+00	1.11	up	1.11	1.000E+00	1.21	up	1.21	1.000E+00
SAOUHSC_02860	MvaS	HMG-CoA synthase	Isoprenoids	-0.71	-1.06	-0.66	-0.62	▼	1.64	down	0.61	6.893E-02	2.09	down	0.48	1.091E-04	1.58	down	0.63	1.000E+00	1.54	down	0.65	1.000E+00
SAOUHSC_02877	CrtN	squalene synthase	Isoprenoids	1.29	1.34	n.d.	0.87	▲	2.45	up	2.45	9.940E-05	2.54	up	2.54	1.226E-03					1.83	up	1.83	2.589E-01
SAOUHSC_00861	LipA	lipoyl synthase	Lipoic acid	0.64	1.42	-1.04	-1.07	▼	1.56	up	1.56	2.729E-01	2.67	up	2.67	5.790E-15	2.05	down	0.49	3.867E-04	2.10	down	0.48	5.700E-08
SAOUHSC_00842	MetN1	ABC transporter ATP-binding protein	Lysine, threonine, methionine, and cysteine	0.29	-0.20	0.83	0.99	▲	1.22	up	1.22	1.000E+00	1.15	down	0.87	1.000E+00	1.78	up	1.78	4.002E-04	1.99	up	1.99	1.533E-04
SAOUHSC_00989		hypothetical protein	Lysine, threonine, methionine, and cysteine	-0.88	-0.39	0.20	-0.16	-	1.85	down	0.54	5.986E-04	1.31	down	0.76	1.000E+00	1.15	up	1.15	1.000E+00	1.12	down	0.89	1.000E+00
SAOUHSC_01395	Asd	aspartate semialdehyde dehydrogenase	Lysine, threonine, methionine, and cysteine	-2.67	-3.31	0.15	0.25	-	6.35	down	0.16	7.860E-18	9.92	down	0.10	7.550E-21	1.11	up	1.11	1.000E+00	1.19	up	1.19	1.000E+00
SAOUHSC_01396	DapA	dihydrodipicolinate synthase	Lysine, threonine, methionine, and cysteine	-3.11	-4.02	-0.05	0.18	-	8.61	down	0.12	6.580E-14	16.23	down	0.06	2.270E-09	1.04	down	0.96	1.000E+00	1.13	up	1.13	1.000E+00
SAOUHSC_01398	DapD	2,3,4,5-tetrahydropyridine-2-carboxylate N-succinyltransferase	Lysine, threonine, methionine, and cysteine	-2.89	-3.97	-0.02	0.14	-	7.39	down	0.14	2.370E-12	15.65	down	0.06	1.990E-10	1.02	down	0.98	1.000E+00	1.11	up	1.11	1.000E+00
SAOUHSC_01399		hypothetical protein	Lysine, threonine, methionine, and cysteine	-2.15	-2.60	0.04	0.16	-	4.42	down	0.23	4.750E-16	6.06	down	0.16	3.680E-14	1.03	up	1.03	1.000E+00	1.12	up	1.12	1.000E+00
SAOUHSC_01400	Alr2	alanine racemase	Lysine, threonine, methionine, and cysteine	-2.04	-0.49	-0.60	-0.52	-	4.10	down	0.24	4.280E-02	1.41	down	0.71	1.000E+00	1.51	down	0.66	1.000E+00	1.43	down	0.70	1.000E+00
SAOUHSC_01401	LysA	diaminopimelate decarboxylase	Lysine, threonine, methionine, and cysteine	-0.53	-1.45	-0.32	-0.25	-	1.44	down	0.69	1.000E+00	2.73	down	0.37	1.970E-12	1.25	down	0.80	1.000E+00	1.19	down	0.84	1.000E+00
SAOUHSC_00426	MetQ2	ABC transporter substrate-binding protein	Lysine, threonine, methionine, and cysteine;Pathogenicity islands	0.10	-0.02	1.21	1.21	▲	1.08	up	1.08	1.000E+00	1.01	down	0.99	1.000E+00	2.31	up	2.31	7.810E-06	2.31	up	2.31	7.600E-05
SAOUHSC_00844	MetQ1	hypothetical protein	Lysine, threonine, methionine, and cysteine;Pathogenicity islands	0.49	-0.03	0.72	0.84	▲	1.41	up	1.41	1.000E+00	1.02	down	0.98	1.000E+00	1.65	up	1.65	1.940E-02	1.79	up	1.79	4.320E-06
SAOUHSC_01828		GAF domain-containing protein	Miscellaneous - no subcategory	-0.19	-0.98	-0.46	-0.50	-	1.14	down	0.88	1.000E+00	1.97	down	0.51	1.795E-02	1.37	down	0.73	1.000E+00	1.42	down	0.71	1.000E+00
SAOUHSC_02425		hypothetical protein	Miscellaneous - no subcategory	-2.03	-2.62	1.06	1.44	▲	4.08	down	0.25	3.710E-09	6.15	down	0.16	2.460E-11	2.08	up	2.08	1.851E-04	2.71	up	2.71	5.630E-09
SAOUHSC_02447		hypothetical protein	Miscellaneous - no subcategory	0.71	-0.26	1.12	1.47	▲	1.64	up	1.64	4.335E-02	1.20	down	0.84	1.000E+00	2.18	up	2.18	8.590E-11	2.77	up	2.77	1.120E-19
SAOUHSC_02754		ABC transporter ATP-binding protein	Miscellaneous - no subcategory	0.93	1.45	n.d.	n.d.	-	1.91	up	1.91	4.582E-02	2.74	up	2.74	5.711E-02								
SAOUHSC_01018	PurD	phosphoribosylamine--glycine ligase	Miscellaneous - no subcategory;Purines	-0.27	-0.03	-1.37	-1.10	▼	1.21	down	0.83	1.000E+00	1.02	down	0.98	1.000E+00	2.58	down	0.39	1.590E-15	2.14	down	0.47	6.070E-20
SAOUHSC_00721	QueC	hypothetical protein	Miscellaneous - no subcategory;RNA processing and modification	1.97	1.23	0.96	1.13	▲	3.92	up	3.92	5.220E-08	2.34	up	2.34	4.412E-04	1.94	up	1.94	1.515E-02	2.19	up	2.19	2.597E-04
SAOUHSC_00364	AhpF	alkyl hydroperoxide reductase subunit F	Miscellaneous - no subcategory;Sulfur Metabolism - no subcategory	0.96	-0.16	0.68	0.86	▲	1.95	up	1.95	8.170E-22	1.12	down	0.90	1.000E+00	1.60	up	1.60	2.514E-03	1.82	up	1.82	2.100E-20
SAOUHSC_00365	AhpC	alkyl hydroperoxide reductase subunit C	Miscellaneous - no subcategory;Sulfur Metabolism - no subcategory	1.02	0.07	1.08	1.27	▲	2.03	up	2.03	4.470E-17	1.05	up	1.05	1.000E+00	2.11	up	2.11	9.270E-17	2.42	up	2.42	1.070E-20
SAOUHSC_00239	RbsK	ribokinase	Monosaccharides	-0.36	-1.03	-0.76	2.51	▲	1.28	down	0.78	1.000E+00	2.04	down	0.49	1.006E-01	1.69	down	0.59	1.000E+00	5.70	up	5.70	8.070E-04
SAOUHSC_00708	FruA	fructose specific permease	Monosaccharides	-0.69	0.84	0.38	0.49	-	1.61	down	0.62	2.803E-01	1.79	up	1.79	3.740E-07	1.30	up	1.30	1.000E+00	1.40	up	1.40	1.000E+00
SAOUHSC_01907		hypothetical protein	Monosaccharides	-1.11	-1.41	0.46	0.63	-	2.16	down	0.46	1.530E-05	2.65	down	0.38	1.990E-10	1.38	up	1.38	1.000E+00	1.55	up	1.55	1.593E-01
SAOUHSC_02809	GntR	gluconate operon transcriptional repressor	Monosaccharides	-1.07	n.d.	1.05	0.85	▲	2.09	down	0.48	2.895E-01					2.06	up	2.06	6.530E-01	1.80	up	1.80	5.907E-04
SAOUHSC_02793		hypothetical protein	Monosaccharides;Capsular and extracellular polysacchrides;Cell Wall and Capsule - no subcategory;Plant-Prokaryote comparative genomics	-0.79	-0.91	-0.70	-0.52	▼	1.73	down	0.58	9.370E-09	1.88	down	0.53	1.385E-02	1.62	down	0.62	6.003E-01	1.44	down	0.70	1.000E+00
SAOUHSC_00501	NupC	hypothetical protein	Monosaccharides;Purines	-0.83	0.21	0.10	-0.01	-	1.78	down	0.56	4.401E-04	1.16	up	1.16	1.000E+00	1.07	up	1.07	1.000E+00	1.01	down	0.99	1.000E+00
SAOUHSC_02377	Pdp	pyrimidine-nucleoside phosphorylase	Monosaccharides;Pyrimidines	-1.32	-1.14	0.64	0.89	▲	2.50	down	0.40	1.160E-28	2.21	down	0.45	1.400E-25	1.56	up	1.56	2.815E-03	1.85	up	1.85	1.770E-32
SAOUHSC_02133		nicotinate phosphoribosyltransferase	NAD and NADP;Oxidative stress	-0.33	-0.18	-0.81	-0.69	▼	1.26	down	0.80	1.000E+00	1.13	down	0.88	1.000E+00	1.75	down	0.57	1.652E-04	1.62	down	0.62	5.873E-02
SAOUHSC_00093	SodM	superoxide dismutase	Nitrogen Metabolism - no subcategory;Oxidative stress	0.47	-0.16	0.80	1.00	▲	1.39	up	1.39	1.000E+00	1.12	down	0.89	1.000E+00	1.74	up	1.74	2.301E-01	1.99	up	1.99	3.802E-02
SAOUHSC_01653	SodA	superoxide dismutase, Mn	Nitrogen Metabolism - no subcategory;Oxidative stress	-1.02	-1.57	0.58	0.84	▲	2.03	down	0.49	3.610E-09	2.96	down	0.34	1.510E-07	1.49	up	1.49	4.708E-01	1.79	up	1.79	1.740E-05
SAOUHSC_00229	ScdA	cell wall biosynthesis protein ScdA	Nitrogen Metabolism - no subcategory;Stress Response - no subcategory	-2.00	-1.32	-1.35	-1.28	▼	4.00	down	0.25	1.282E-04	2.49	down	0.40	2.820E-03	2.54	down	0.39	2.971E-03	2.42	down	0.41	2.493E-02
SAOUHSC_00743	NrdF	ribonucleotide-diphosphate reductase subunit beta	Nucleosides and Nucleotides - no subcategory	1.31	0.53	-0.76	-0.79	▼	2.48	up	2.48	3.480E-26	1.44	up	1.44	2.977E-01	1.69	down	0.59	1.770E-01	1.73	down	0.58	6.430E-07
SAOUHSC_00742	NrdE	ribonucleotide-diphosphate reductase subunit alpha	Nucleosides and Nucleotides - no subcategory;Oxidative stress	1.57	0.97	-0.81	-0.78	▼	2.96	up	2.96	2.550E-71	1.96	up	1.96	2.180E-33	1.75	down	0.57	2.121E-04	1.72	down	0.58	7.720E-11
SAOUHSC_00367	TcyP	hypothetical protein	Organic sulfur assimilation	n.d.	-1.64	2.02	2.37	▲					3.13	down	0.32	2.938E-01	4.04	up	4.04	2.540E-08	5.18	up	5.18	1.820E-11
SAOUHSC_00731		ABC transporter	Osmotic stress	-1.09	-0.73	-0.46	-0.65	-	2.13	down	0.47	1.530E-08	1.66	down	0.60	1.000E+00	1.38	down	0.73	1.000E+00	1.57	down	0.64	4.808E-01
SAOUHSC_00732	OpuBB	amino acid ABC transporter permease	Osmotic stress	-1.24	-0.48	-0.38	-0.54	-	2.36	down	0.42	9.590E-11	1.40	down	0.71	1.000E+00	1.30	down	0.77	1.000E+00	1.45	down	0.69	1.000E+00
SAOUHSC_02932	BetA	choline dehydrogenase	Osmotic stress	1.86	1.50	0.72	0.77	▲	3.63	up	3.63	3.480E-05	2.84	up	2.84	9.840E-06	1.64	up	1.64	3.180E-01	1.71	up	1.71	1.858E-01
SAOUHSC_02933	BetB	betaine aldehyde dehydrogenase	Osmotic stress	1.94	0.95	0.67	0.93	▲	3.83	up	3.83	1.400E-13	1.93	up	1.93	3.570E-06	1.59	up	1.59	1.000E+00	1.90	up	1.90	2.150E-07
SAOUHSC_00831	OsmC	hypothetical protein	Oxidative stress	1.36	0.30	0.41	n.d.	-	2.57	up	2.57	1.282E-04	1.23	up	1.23	1.000E+00	1.33	up	1.33	1.000E+00				
SAOUHSC_01282	BsaA	glutathione peroxidase	Oxidative stress	-0.39	-0.86	-0.55	-0.12	-	1.31	down	0.76	1.000E+00	1.82	down	0.55	1.518E-02	1.46	down	0.69	1.000E+00	1.09	down	0.92	1.000E+00
SAOUHSC_01327	KatA	catalase	Oxidative stress	0.52	-0.22	0.78	1.22	▲	1.44	up	1.44	1.000E+00	1.16	down	0.86	1.000E+00	1.72	up	1.72	2.971E-03	2.32	up	2.32	1.010E-23
SAOUHSC_02381	Dps	hypothetical protein	Oxidative stress	0.09	-0.25	1.65	2.19	▲	1.06	up	1.06	1.000E+00	1.19	down	0.84	1.000E+00	3.15	up	3.15	3.490E-07	4.55	up	4.55	9.270E-14
SAOUHSC_00204	Hmp	globin domain-containing protein	Oxidative stress;Stress Response - no subcategory	-2.25	-1.48	-1.48	-1.33	▼	4.76	down	0.21	4.390E-13	2.79	down	0.36	5.290E-14	2.79	down	0.36	6.660E-09	2.51	down	0.40	8.030E-10
SAOUHSC_00711		hypothetical protein	Phosphorus Metabolism - no subcategory	0.06	0.80	0.38	0.44	-	1.05	up	1.05	1.000E+00	1.74	up	1.74	2.288E-03	1.30	up	1.30	1.000E+00	1.36	up	1.36	1.000E+00
SAOUHSC_01384	PhoU	PhoU family transcriptional regulator	Phosphorus Metabolism - no subcategory	-2.54	n.d.	1.87	2.25	▲	5.83	down	0.17	2.895E-01					3.64	up	3.64	2.267E-04	4.75	up	4.75	6.680E-07
SAOUHSC_01389	PstS	phosphate ABC transporter periplasmic phosphate-binding protein	Phosphorus Metabolism - no subcategory	-3.96	-4.92	2.09	2.40	▲	15.58	down	0.06	1.544E-01	30.30	down	0.03	2.938E-01	4.25	up	4.25	1.080E-11	5.29	up	5.29	7.120E-10
SAOUHSC_01800	PhoP	alkaline phosphatase synthesis transcriptional regulatory protein	Phosphorus Metabolism - no subcategory	-0.97	-0.81	0.72	0.96	▲	1.95	down	0.51	1.584E-04	1.75	down	0.57	1.000E+00	1.65	up	1.65	2.180E-02	1.95	up	1.95	1.020E-12
SAOUHSC_01251	PnpA	polynucleotide phosphorylase/polyadenylase	Plant-Prokaryote comparative genomics	0.45	0.71	-0.15	0.06	-	1.37	up	1.37	1.000E+00	1.64	up	1.64	2.910E-06	1.11	down	0.90	1.000E+00	1.04	up	1.04	1.000E+00
SAOUHSC_01252	RnjB	hypothetical protein	Plant-Prokaryote comparative genomics	0.77	0.68	0.12	0.13	-	1.71	up	1.71	3.060E-05	1.60	up	1.60	6.356E-04	1.09	up	1.09	1.000E+00	1.10	up	1.10	1.000E+00
SAOUHSC_02583		transcriptional regulator	Plant-Prokaryote comparative genomics;Regulation and Cell signaling - no subcategory	-2.25	-2.11	-0.56	-0.82	▼	4.77	down	0.21	1.510E-11	4.32	down	0.23	6.090E-15	1.48	down	0.68	1.000E+00	1.76	down	0.57	4.383E-04
SAOUHSC_02627		hypothetical protein	Polysaccharides	-1.29	0.07	0.14	0.15	-	2.44	down	0.41	1.039E-03	1.05	up	1.05	1.000E+00	1.10	up	1.10	1.000E+00	1.11	up	1.11	1.000E+00
SAOUHSC_01701		hypothetical protein	Predictions based on plant-prokaryote comparative analysis - no subcategory	-0.53	1.01	0.34	-0.66	-	1.45	down	0.69	1.000E+00	2.02	up	2.02	1.180E-06	1.27	up	1.27	1.000E+00	1.58	down	0.63	1.000E+00
SAOUHSC_01689	RpsT	30S ribosomal protein S20	Predictions based on plant-prokaryote comparative analysis - no subcategory;Protein biosynthesis	0.32	0.11	-0.97	-1.09	▼	1.25	up	1.25	1.000E+00	1.08	up	1.08	1.000E+00	1.96	down	0.51	1.406E-01	2.14	down	0.47	2.805E-04
SAOUHSC_00556	ProP	proline/betaine transporter	Proline and 4-hydroxyproline	2.16	1.68	n.d.	1.68	▲	4.46	up	4.46	2.237E-02	3.21	up	3.21	1.959E-01					3.20	up	3.20	1.000E+00
SAOUHSC_01884	PutA	hypothetical protein	Proline and 4-hydroxyproline;Electron donating reactions	n.d.	n.d.	1.43	1.55	▲									2.69	up	2.69	1.140E-07	2.93	up	2.93	1.510E-09
SAOUHSC_01695	RsfS	hypothetical protein	Proline and 4-hydroxyproline;NAD and NADP	0.76	0.83	-0.52	-0.41	-	1.70	up	1.70	2.046E-02	1.78	up	1.78	1.509E-03	1.43	down	0.70	1.000E+00	1.33	down	0.75	1.000E+00
SAOUHSC_00017	RplI	50S ribosomal protein L9	Protein biosynthesis	1.05	0.81	-1.17	-0.69	▼	2.06	up	2.06	3.150E-08	1.76	up	1.76	6.609E-03	2.25	down	0.44	4.987E-02	1.61	down	0.62	1.000E+00

The comparison of the early intracellular phase sample (8h p.i.) and late intracellular phase samples (24h and 32h p.i.) revealed proteins that were not significantly altered in their amount by using the non-adherent control as point of reference, because proteins were not detected in the non-adherent control sample or proteins were altered by opposing changes (early phase / late phase).

locus tag	protein symbol	description	theSEED functional category	log2 ratio OD=0.4	log2 ratio non-adherent	log2 ratio 24h p.i.	log2 ratio 32h p.i.	median over 24 and 32h ratio; median tendence - 1.5fc cutoff	median fold-change OD=0.4	median fold-change (direction) OD=0.4	median ratio over assays against 8h p.i. OD=0.4	p-value BH adjusted OD=0.4	median fold-change non-ad.	median fold-change (direction) non-ad.	median ratio over assays against 8h p.i. non-ad.	p-value BH adjusted non-ad.	median fold-change 24h p.i.	median fold-change (direction) 24h p.i.	median ratio over assays against 8h p.i. 24h p.i.	p-value BH adjusted 24h p.i.	median fold-change 32h p.i.	median fold-change (direction) 32h p.i.	median ratio over assays against 8h p.i. 32h p.i.	p-value BH adjusted 32h p.i.
SAOUHSC_00348	RpsF	30S ribosomal protein S6	Protein biosynthesis	0.39	0.79	-0.20	0.07	-	1.31	up	1.31	1.000E+00	1.73	up	1.73	1.690E-04	1.14	down	0.87	1.000E+00	1.05	up	1.05	1.000E+00
SAOUHSC_00474	RplY	50S ribosomal protein L25/general stress protein Ctc	Protein biosynthesis	0.89	0.19	-0.22	-0.03	-	1.85	up	1.85	2.820E-11	1.14	up	1.14	1.000E+00	1.17	down	0.86	1.000E+00	1.02	down	0.98	1.000E+00
SAOUHSC_00518	RplK	50S ribosomal protein L11	Protein biosynthesis	0.86	0.82	-0.52	-0.48	-	1.82	up	1.82	3.240E-06	1.77	up	1.77	5.790E-05	1.44	down	0.70	1.000E+00	1.40	down	0.72	1.000E+00
SAOUHSC_00519	RplA	50S ribosomal protein L1	Protein biosynthesis	0.78	1.04	-0.20	0.04	-	1.72	up	1.72	3.180E-05	2.05	up	2.05	2.400E-09	1.15	down	0.87	1.000E+00	1.03	up	1.03	1.000E+00
SAOUHSC_00767	SaHPF	hypothetical protein	Protein biosynthesis	-0.67	-1.40	0.98	1.26	▲	1.59	down	0.63	1.000E+00	2.63	down	0.38	1.060E-06	1.97	up	1.97	3.030E-09	2.40	up	2.40	1.360E-17
SAOUHSC_01058	TypA	GTP-binding protein TypA	Protein biosynthesis	0.24	0.99	0.28	0.40	-	1.18	up	1.18	1.000E+00	1.98	up	1.98	1.990E-25	1.21	up	1.21	1.000E+00	1.32	up	1.32	1.000E+00
SAOUHSC_01092	PheS	phenylalanyl-tRNA synthetase subunit alpha	Protein biosynthesis	0.85	1.00	0.07	0.23	-	1.80	up	1.80	4.840E-07	1.99	up	1.99	2.980E-12	1.05	up	1.05	1.000E+00	1.18	up	1.18	1.000E+00
SAOUHSC_01093	PheT	phenylalanyl-tRNA synthetase subunit beta	Protein biosynthesis	0.54	0.90	-0.06	0.09	-	1.46	up	1.46	1.000E+00	1.86	up	1.86	4.880E-23	1.04	down	0.96	1.000E+00	1.07	up	1.07	1.000E+00
SAOUHSC_01163		hypothetical protein	Protein biosynthesis	0.47	0.66	0.34	0.33	-	1.38	up	1.38	1.000E+00	1.58	up	1.58	2.227E-02	1.27	up	1.27	1.000E+00	1.26	up	1.26	1.000E+00
SAOUHSC_01211	RplS	50S ribosomal protein L19	Protein biosynthesis	0.45	0.71	-0.21	-0.09	-	1.36	up	1.36	1.000E+00	1.63	up	1.63	3.483E-03	1.15	down	0.87	1.000E+00	1.06	down	0.94	1.000E+00
SAOUHSC_01232	RpsB	30S ribosomal protein S2	Protein biosynthesis	0.54	0.64	-0.10	-0.03	-	1.45	up	1.45	4.546E-01	1.55	up	1.55	1.795E-02	1.07	down	0.93	1.000E+00	1.02	down	0.98	1.000E+00
SAOUHSC_01234	Tsf	elongation factor Ts	Protein biosynthesis	0.61	0.06	-0.21	0.02	-	1.53	up	1.53	1.601E-03	1.04	up	1.04	1.000E+00	1.15	down	0.87	1.000E+00	1.01	up	1.01	1.000E+00
SAOUHSC_01678	RpsU	30S ribosomal protein S21	Protein biosynthesis	0.50	0.82	-1.30	-0.94	▼	1.41	up	1.41	8.378E-01	1.77	up	1.77	5.238E-02	2.45	down	0.41	5.531E-03	1.92	down	0.52	3.269E-02
SAOUHSC_01755	RpmA	50S ribosomal protein L27	Protein biosynthesis	1.02	0.71	-0.61	-0.47	-	2.02	up	2.02	5.370E-07	1.64	up	1.64	1.593E-01	1.53	down	0.65	1.000E+00	1.38	down	0.72	1.000E+00
SAOUHSC_01757	RplU	50S ribosomal protein L21	Protein biosynthesis	0.56	0.95	-0.28	-0.20	-	1.48	up	1.48	1.000E+00	1.93	up	1.93	3.660E-10	1.21	down	0.82	1.000E+00	1.15	down	0.87	1.000E+00
SAOUHSC_01829	RpsD	30S ribosomal protein S4	Protein biosynthesis	0.34	0.84	-0.05	-0.24	-	1.27	up	1.27	1.000E+00	1.79	up	1.79	5.050E-09	1.04	down	0.96	1.000E+00	1.19	down	0.84	1.000E+00
SAOUHSC_02361	RpmE2	50S ribosomal protein L31 type B	Protein biosynthesis	0.80	0.81	-0.44	-0.31	-	1.75	up	1.75	1.138E-02	1.76	up	1.76	1.304E-02	1.36	down	0.74	1.000E+00	1.24	down	0.81	1.000E+00
SAOUHSC_02486	RpsK	30S ribosomal protein S11	Protein biosynthesis	0.61	0.98	-0.53	-0.74	▼	1.53	up	1.53	4.413E-01	1.97	up	1.97	1.470E-06	1.44	down	0.69	1.000E+00	1.67	down	0.60	1.339E-02
SAOUHSC_02487	RpsM	30S ribosomal protein S13	Protein biosynthesis	0.75	1.10	-0.10	-0.12	-	1.68	up	1.68	1.536E-02	2.15	up	2.15	1.420E-09	1.07	down	0.93	1.000E+00	1.08	down	0.92	1.000E+00
SAOUHSC_02492	RplO	50S ribosomal protein L15	Protein biosynthesis	0.82	1.00	-0.43	-0.33	-	1.77	up	1.77	4.910E-06	2.00	up	2.00	3.180E-09	1.35	down	0.74	1.000E+00	1.26	down	0.80	1.000E+00
SAOUHSC_02493	RpmD	50S ribosomal protein L30	Protein biosynthesis	0.73	0.32	-0.38	-0.19	-	1.66	up	1.66	8.766E-03	1.25	up	1.25	1.000E+00	1.31	down	0.77	1.000E+00	1.14	down	0.88	1.000E+00
SAOUHSC_02496	RplF	50S ribosomal protein L6	Protein biosynthesis	0.67	0.53	-0.09	-0.10	-	1.59	up	1.59	8.685E-03	1.45	up	1.45	3.891E-01	1.07	down	0.94	1.000E+00	1.07	down	0.93	1.000E+00
SAOUHSC_02500	RplE	50S ribosomal protein L5	Protein biosynthesis	0.39	0.57	0.03	0.04	-	1.31	up	1.31	1.000E+00	1.48	up	1.48	1.419E-02	1.02	up	1.02	1.000E+00	1.03	up	1.03	1.000E+00
SAOUHSC_02504	RpmC	50S ribosomal protein L29	Protein biosynthesis	0.81	0.77	-0.42	-0.36	-	1.75	up	1.75	1.883E-02	1.71	up	1.71	7.542E-02	1.34	down	0.75	1.000E+00	1.28	down	0.78	1.000E+00
SAOUHSC_02505	RplP	50S ribosomal protein L16	Protein biosynthesis	0.41	1.07	-0.58	-0.36	-	1.33	up	1.33	1.000E+00	2.10	up	2.10	8.200E-06	1.50	down	0.67	1.000E+00	1.28	down	0.78	1.000E+00
SAOUHSC_02506	RpsC	30S ribosomal protein S3	Protein biosynthesis	0.76	0.97	0.19	0.10	-	1.69	up	1.69	1.710E-06	1.96	up	1.96	6.310E-11	1.14	up	1.14	1.000E+00	1.07	up	1.07	1.000E+00
SAOUHSC_02507	RplV	50S ribosomal protein L22	Protein biosynthesis	0.47	0.77	-0.08	-0.35	-	1.39	up	1.39	8.814E-01	1.70	up	1.70	7.770E-03	1.05	down	0.95	1.000E+00	1.28	down	0.78	1.000E+00
SAOUHSC_02508	RpsS	30S ribosomal protein S19	Protein biosynthesis	0.51	0.89	-0.52	-0.90	▼	1.43	up	1.43	1.000E+00	1.85	up	1.85	1.486E-02	1.43	down	0.70	1.000E+00	1.86	down	0.54	6.339E-02
SAOUHSC_02509	RplB	50S ribosomal protein L2	Protein biosynthesis	0.51	0.84	-0.24	-0.28	-	1.43	up	1.43	1.000E+00	1.79	up	1.79	3.530E-07	1.18	down	0.85	1.000E+00	1.21	down	0.82	1.000E+00
SAOUHSC_02511	RplD	50S ribosomal protein L4	Protein biosynthesis	0.71	0.85	-0.52	-0.17	-	1.63	up	1.63	9.113E-03	1.80	up	1.80	5.080E-06	1.43	down	0.70	1.000E+00	1.12	down	0.89	1.000E+00
SAOUHSC_00324		ribosomal-protein-serine acetyltransferase	Protein biosynthesis;Protein processing and modification	-0.44	-1.01	0.89	1.01	▲	1.35	down	0.74	1.000E+00	2.02	down	0.50	2.255E-02	1.85	up	1.85	3.857E-02	2.02	up	2.02	2.530E-06
SAOUHSC_00527	RpsL	30S ribosomal protein S12	Protein biosynthesis;Protein processing and modification;Virulence - no subcategory	1.16	0.76	-0.01	-0.27	-	2.23	up	2.23	8.980E-07	1.70	up	1.70	1.793E-01	1.01	down	0.99	1.000E+00	1.21	down	0.83	1.000E+00
SAOUHSC_00528	RpsG	30S ribosomal protein S7	Protein biosynthesis;Virulence - no subcategory	0.66	1.01	-0.70	-0.79	▼	1.58	up	1.58	3.641E-02	2.01	up	2.01	1.080E-09	1.63	down	0.61	6.003E-01	1.72	down	0.58	4.320E-06
SAOUHSC_00248	LytM	peptidoglycan hydrolase	Protein degradation	2.13	1.02	n.d.	n.d.	-	4.37	up	4.37	4.910E-06	2.02	up	2.02	1.748E-02								
SAOUHSC_00505	ClpC	endopeptidase	Protein degradation	0.63	0.48	0.26	0.68	-	1.55	up	1.55	1.335E-02	1.39	up	1.39	9.458E-01	1.20	up	1.20	1.000E+00	1.60	up	1.60	1.840E-08
SAOUHSC_01778	ClpX	ATP-dependent protease ATP-binding subunit ClpX	Protein degradation	0.46	0.72	-0.83	-0.96	▼	1.38	up	1.38	1.000E+00	1.64	up	1.64	2.600E-06	1.77	down	0.56	8.170E-05	1.95	down	0.51	3.410E-09
SAOUHSC_01861		hypothetical protein	Protein degradation	1.35	0.95	n.d.	n.d.	-	2.55	up	2.55	3.790E-06	1.94	up	1.94	7.873E-03								
SAOUHSC_00912	ClpB	ATP-dependent Clp protease, ATP-binding subunit ClpB	Protein degradation;Protein folding	0.72	0.75	1.84	2.60	▲	1.65	up	1.65	1.890E-06	1.69	up	1.69	9.590E-09	3.58	up	3.58	4.460E-43	6.08	up	6.08	4.140E-66
SAOUHSC_01683	DnaK	molecular chaperone DnaK	Protein folding;Heat shock	0.61	0.37	0.44	0.70	-	1.52	up	1.52	8.946E-02	1.29	up	1.29	1.000E+00	1.36	up	1.36	1.000E+00	1.63	up	1.63	1.630E-09
SAOUHSC_01431	MsrB	methionine sulfoxide reductase B	Protein processing and modification	-2.18	-2.69	-0.70	-0.81	▼	4.54	down	0.22	7.000E-08	6.47	down	0.15	6.840E-10	1.63	down	0.61	1.000E+00	1.75	down	0.57	4.775E-04
SAOUHSC_01432	MsrA2	methionine sulfoxide reductase A	Protein processing and modification	-2.32	-2.57	-0.82	-0.95	▼	5.00	down	0.20	2.140E-11	5.94	down	0.17	1.660E-11	1.77	down	0.57	8.883E-01	1.93	down	0.52	1.940E-05
SAOUHSC_01269	MiaB	(dimethylallyl)adenosine tRNA methylthiotransferase	Protein processing and modification;RNA processing and modification	1.21	1.29	-1.56	-1.51	▼	2.31	up	2.31	2.300E-10	2.44	up	2.44	1.140E-11	2.94	down	0.34	3.929E-01	2.85	down	0.35	2.493E-02
SAOUHSC_01679	MtaB	hypothetical protein	Protein processing and modification;RNA processing and modification;Heat shock	1.17	1.36	n.d.	n.d.	-	2.25	up	2.25	2.090E-03	2.57	up	2.57	3.847E-04								
SAOUHSC_00257	EsxA	hypothetical protein	Protein secretion system, Type VII	-0.16	-0.31	0.62	0.81	▲	1.12	down	0.90	1.000E+00	1.24	down	0.81	1.000E+00	1.54	up	1.54	1.000E+00	1.75	up	1.75	1.777E-02
SAOUHSC_01972	PrsA	protein export protein PrsA	Protein translocation across cytoplasmic membrane;Plant-Prokaryote comparative genomics;Protein folding	-2.16	-2.19	-0.37	-0.76	-	4.47	down	0.22	4.150E-16	4.56	down	0.22	4.420E-19	1.29	down	0.78	1.000E+00	1.69	down	0.59	2.437E-01
SAOUHSC_01008	PurE	phosphoribosylaminoimidazole carboxylase, catalytic subunit	Purines	-0.64	-0.46	-1.08	-0.74	▼	1.56	down	0.64	1.000E+00	1.38	down	0.73	1.000E+00	2.11	down	0.47	2.149E-02	1.67	down	0.60	5.883E-01
SAOUHSC_01009	PurK	phosphoribosylaminoimidazole carboxylase ATPase subunit	Purines	-0.55	-0.34	-1.13	-0.96	▼	1.46	down	0.68	1.000E+00	1.26	down	0.79	1.000E+00	2.19	down	0.46	2.890E-06	1.94	down	0.51	3.580E-07
SAOUHSC_01010	PurC	phosphoribosylaminoimidazole-succinocarboxamide synthase	Purines	-0.38	-0.39	-1.26	-1.02	▼	1.30	down	0.77	1.000E+00	1.31	down	0.76	1.000E+00	2.39	down	0.42	2.310E-20	2.03	down	0.49	4.740E-19
SAOUHSC_01011	PurS	phosphoribosylformylglycinamidine synthase, PurS protein	Purines	-0.45	-0.73	-1.50	-1.21	▼	1.37	down	0.73	1.000E+00	1.66	down	0.60	2.650E-01	2.83	down	0.35	2.230E-12	2.31	down	0.43	5.080E-15
SAOUHSC_01012	PurQ	phosphoribosylformylglycinamidine synthase I	Purines	-0.48	-0.54	-1.40	-1.27	▼	1.40	down	0.72	1.000E+00	1.45	down	0.69	1.000E+00	2.64	down	0.38	1.160E-12	2.40	down	0.42	4.650E-19
SAOUHSC_01013	PurL	phosphoribosylformylglycinamidine synthase II	Purines	-0.51	-0.49	-0.75	-0.80	▼	1.42	down	0.70	1.000E+00	1.40	down	0.71	1.000E+00	1.68	down	0.59	5.061E-03	1.74	down	0.57	6.190E-11
SAOUHSC_01015	PurM	phosphoribosylaminoimidazole synthetase	Purines	-0.55	-0.63	-1.40	-0.95	▼	1.47	down	0.68	1.000E+00	1.54	down	0.65	1.000E+00	2.64	down	0.38	1.640E-07	1.93	down	0.52	1.190E-05
SAOUHSC_01017	PurH	bifunctional phosphoribosylaminoimidazolecarboxamide formyltransferase/IMP cyclohydrolase	Purines	-0.45	0.17	-0.78	-0.74	▼	1.36	down	0.73	1.000E+00	1.12	up	1.12	1.000E+00	1.72	down	0.58	1.000E+00	1.67	down	0.60	9.543E-04
SAOUHSC_01014	PurF	amidophosphoribosyltransferase	Purines;Bacteriocins, ribosomally synthesized antibacterial peptides	-0.58	-0.54	-1.49	-1.31	▼	1.50	down	0.67	1.000E+00	1.45	down	0.69	1.000E+00	2.80	down	0.36	4.210E-12	2.48	down	0.40	3.770E-18
SAOUHSC_00375	GuaA	GMP synthase	Purines;Pathogenicity islands	0.08	-0.29	-0.74	-0.56	▼	1.06	up	1.06	1.000E+00	1.22	down	0.82	1.000E+00	1.67	down	0.60	2.911E-02	1.48	down	0.68	1.000E+00
SAOUHSC_01016	PurN	phosphoribosylglycinamide formyltransferase	Purines;Potassium metabolism - no subcategory	-1.24	-0.58	-1.12	-1.11	▼	2.37	down	0.42	1.960E-11	1.50	down	0.67	1.000E+00	2.18	down	0.46	6.300E-07	2.15	down	0.46	5.510E-10
SAOUHSC_01485	Ndk	nucleoside diphosphate kinase	Purines;Pyrimidines	0.08	-0.28	0.71	0.76	▲	1.06	up	1.06	1.000E+00	1.22	down	0.82	1.000E+00	1.64	up	1.64	1.665E-02	1.69	up	1.69	2.780E-05
SAOUHSC_01166	PyrB	aspartate carbamoyltransferase catalytic subunit	Pyrimidines	0.17	0.87	0.05	0.29	-	1.13	up	1.13	1.000E+00	1.83	up	1.83	2.469E-04	1.04	up	1.04	1.000E+00	1.22	up	1.22	1.000E+00
SAOUHSC_01169	CarA	carbamoyl phosphate synthase small subunit	Pyrimidines	-0.01	0.76	0.23	0.24	-	1.01	down	0.99	1.000E+00	1.70	up	1.70	6.070E-05	1.17	up	1.17	1.000E+00	1.18	up	1.18	1.000E+00
SAOUHSC_01170	CarB	carbamoyl phosphate synthase large subunit	Pyrimidines	0.02	0.81	-0.01	0.23	-	1.01	up	1.01	1.000E+00	1.76	up	1.76	2.050E-23	1.01	down	0.99	1.000E+00	1.17	up	1.17	1.000E+00
SAOUHSC_02909	PyrD	dihydroorotate dehydrogenase 2	Pyrimidines	-1.08	-0.40	-0.15	-0.31	-	2.12	down	0.47	6.130E-06	1.32	down	0.76	1.000E+00	1.11	down	0.90	1.000E+00	1.24	down	0.80	1.000E+00
SAOUHSC_02654		hypothetical protein	Pyrimidines;Sulfur Metabolism - no subcategory	-2.42	-0.70	0.31	0.06	-	5.34	down	0.19	6.310E-03	1.62	down	0.62	1.000E+00	1.24	up	1.24	1.000E+00	1.04	up	1.04	1.000E+00
SAOUHSC_02298	SigB	RNA polymerase sigma factor SigB	Quorum sensing and biofilm formation;Transcription;Stress Response - no subcategory;Resistance to antibiotics and toxic compounds	1.03	0.54	-0.21	-0.20	-	2.04	up	2.04	1.760E-09	1.45	up	1.45	2.838E-01	1.16	down	0.86	1.000E+00	1.15	down	0.87	1.000E+00

The comparison of the early intracellular phase sample (8h p.i.) and late intracellular phase samples (24h and 32h p.i.) revealed proteins that were not significantly altered in their amount by using the non-adherent control as point of reference, because proteins were not detected in the non-adherent control sample or proteins were altered by opposing changes (early phase / late phase).

locus tag	protein symbol	description	theSEED functional category	log2 ratio OD=0.4	log2 ratio non-adherent	log2 ratio 24h p.i.	log2 ratio 32h p.i.	median over 24 and 32h ratio; median tendence - 1.5fc cutoff	median fold-change OD=0.4	median fold-change (direction) OD=0.4	median ratio over assays against 8h p.i. OD=0.4	p-value BH adjusted OD=0.4	median fold-change non-ad.	median fold-change (direction) non-ad.	median ratio over assays against 8h p.i. non-ad.	p-value BH adjusted non-ad.	median fold-change 24h p.i.	median fold-change (direction) 24h p.i.	median ratio over assays against 8h p.i. 24h p.i.	p-value BH adjusted 24h p.i.	median fold-change 32h p.i.	median fold-change (direction) 32h p.i.	median ratio over assays against 8h p.i. 32h p.i.	p-value BH adjusted 32h p.i.
SAOUHSC_00940		hypothetical protein	Regulation and Cell signaling - no subcategory	-0.81	-1.04	n.d.	n.d.	-	1.75	down	0.57	1.000E+00	2.05	down	0.49	5.122E-03								
SAOUHSC_01361	MsrR	transcriptional regulator	Regulation and Cell signaling - no subcategory	-1.20	-0.84	-0.73	-1.03	▼	2.30	down	0.43	2.180E-10	1.79	down	0.56	2.137E-02	1.66	down	0.60	1.000E+00	2.05	down	0.49	2.310E-05
SAOUHSC_00998	FmtA	fmt protein	Regulation and Cell signaling - no subcategory;Resistance to antibiotics and toxic compounds	-2.24	-1.34	-0.48	-1.01	▼	4.73	down	0.21	1.310E-09	2.52	down	0.40	2.710E-05	1.39	down	0.72	1.000E+00	2.02	down	0.50	1.400E-03
SAOUHSC_01979		hypothetical protein	Resistance to antibiotics and toxic compounds	0.65	0.92	-1.38	-1.71	▼	1.57	up	1.57	5.967E-01	1.89	up	1.89	2.780E-07	2.59	down	0.39	4.000E-11	3.28	down	0.30	1.820E-11
SAOUHSC_02630		hypothetical protein	Resistance to antibiotics and toxic compounds	0.01	1.57	-0.52	-0.28	-	1.01	up	1.01	1.000E+00	2.97	up	2.97	4.674E-03	1.43	down	0.70	1.000E+00	1.22	down	0.82	1.000E+00
SAOUHSC_02635	TcaA	hypothetical protein	Resistance to antibiotics and toxic compounds	-1.35	-0.76	-0.25	-0.45	-	2.54	down	0.39	3.889E-04	1.69	down	0.59	1.608E-02	1.19	down	0.84	1.000E+00	1.37	down	0.73	1.000E+00
SAOUHSC_02582	FdhA	formate dehydrogenase subunit alpha	Respiration - no subcategory	1.83	1.37	0.40	0.78	▲	3.56	up	3.56	1.430E-12	2.58	up	2.58	5.440E-11	1.32	up	1.32	1.000E+00	1.72	up	1.72	3.717E-02
SAOUHSC_01889	RibD	riboflavin biosynthesis protein RibD	Riboflavin, FMN, FAD	1.37	1.24	n.d.	1.01	▲	2.58	up	2.58	2.237E-02	2.36	up	2.36	9.459E-01					2.02	up	2.02	3.074E-01
SAOUHSC_00719	QueE	hypothetical protein	RNA processing and modification	1.77	1.75	0.21	1.08	▲	3.42	up	3.42	2.237E-02	3.36	up	3.36	8.621E-02	1.16	up	1.16	1.000E+00	2.12	up	2.12	1.000E+00
SAOUHSC_00951		hypothetical protein	RNA processing and modification	-0.84	-1.11	1.28	1.53	▲	1.79	down	0.56	8.121E-02	2.16	down	0.46	2.290E-06	2.42	up	2.42	1.560E-13	2.88	up	2.88	7.740E-23
SAOUHSC_01659		hypothetical protein	RNA processing and modification	0.17	0.72	0.02	0.18	-	1.12	up	1.12	1.000E+00	1.65	up	1.65	3.534E-02	1.02	up	1.02	1.000E+00	1.14	up	1.14	1.000E+00
SAOUHSC_02097		hypothetical protein	RNA processing and modification	1.60	1.11	0.16	0.55	-	3.03	up	3.03	1.050E-12	2.16	up	2.16	4.680E-07	1.12	up	1.12	1.000E+00	1.47	up	1.47	1.000E+00
SAOUHSC_02316	CshA	DEAD-box ATP dependent DNA helicase	RNA processing and modification	0.37	1.07	0.08	0.20	-	1.30	up	1.30	1.000E+00	2.11	up	2.11	3.310E-36	1.06	up	1.06	1.000E+00	1.15	up	1.15	1.000E+00
SAOUHSC_00074	SirA	periplasmic binding protein	Siderophores	-2.49	-0.12	-0.23	-0.30	-	5.62	down	0.18	9.430E-15	1.09	down	0.92	1.000E+00	1.18	down	0.85	1.000E+00	1.23	down	0.81	1.000E+00
SAOUHSC_00075	SbnA	hypothetical protein	Siderophores	n.d.	-2.20	0.17	-0.71	-					4.61	down	0.22	7.070E-07	1.13	up	1.13	1.000E+00	1.63	down	0.61	1.000E+00
SAOUHSC_00077	SbnC	hypothetical protein	Siderophores	n.d.	-2.13	-0.39	-0.90	▼					4.38	down	0.23	1.860E-05	1.31	down	0.76	1.000E+00	1.86	down	0.54	2.287E-02
SAOUHSC_00079	SbnE	hypothetical protein	Siderophores	n.d.	-1.93	0.35	-0.22	-					3.80	down	0.26	3.360E-10	1.27	up	1.27	1.000E+00	1.16	down	0.86	1.000E+00
SAOUHSC_00080	SbnF	hypothetical protein	Siderophores	n.d.	-1.62	-0.10	-0.34	-					3.07	down	0.33	6.204E-04	1.07	down	0.94	1.000E+00	1.26	down	0.79	1.000E+00
SAOUHSC_01819	UspA1	hypothetical protein	Stress Response - no subcategory	-1.13	-1.46	1.23	1.54	▲	2.19	down	0.46	4.110E-12	2.74	down	0.36	6.430E-21	2.35	up	2.35	6.130E-19	2.91	up	2.91	1.170E-32
SAOUHSC_02299	RsbW	serine-protein kinase RsbW	Stress Response - no subcategory	0.86	0.57	-0.24	0.04	-	1.81	up	1.81	5.210E-09	1.48	up	1.48	3.802E-02	1.18	down	0.84	1.000E+00	1.03	up	1.03	1.000E+00
SAOUHSC_02300	RsbV	STAS domain-containing protein	Stress Response - no subcategory	0.87	0.42	-0.01	0.20	-	1.82	up	1.82	1.968E-04	1.34	up	1.34	1.000E+00	1.01	down	0.99	1.000E+00	1.15	up	1.15	1.000E+00
SAOUHSC_02402	MtlA	PTS system mannitol-specific transporter subunit IIA	Sugar alcohols	1.49	0.64	0.58	1.04	▲	2.81	up	2.81	9.470E-12	1.56	up	1.56	2.196E-01	1.50	up	1.50	1.000E+00	2.06	up	2.06	1.000E-07
SAOUHSC_02403	MtlD	mannitol-1-phosphate 5-dehydrogenase	Sugar alcohols	1.34	0.38	0.74	1.06	▲	2.53	up	2.53	3.020E-15	1.30	up	1.30	1.000E+00	1.67	up	1.67	6.794E-02	2.08	up	2.08	1.070E-08
SAOUHSC_01278	GlpD	aerobic glycerol-3-phosphate dehydrogenase	Sugar alcohols;Phospholipids;Electron donating reactions	0.20	0.33	0.63	0.90	▲	1.15	up	1.15	1.000E+00	1.26	up	1.26	1.000E+00	1.55	up	1.55	3.462E-03	1.86	up	1.86	1.020E-28
SAOUHSC_00833		hypothetical protein	Tetrapyrroles	-1.80	-0.91	-0.55	-0.29	-	3.49	down	0.29	3.420E-09	1.88	down	0.53	6.912E-02	1.46	down	0.68	1.000E+00	1.22	down	0.82	1.000E+00
SAOUHSC_01960	HemY	protoporphyrinogen oxidase	Tetrapyrroles	0.37	0.48	0.62	1.02	▲	1.29	up	1.29	1.000E+00	1.40	up	1.40	1.000E+00	1.53	up	1.53	8.970E-01	2.02	up	2.02	3.662E-02
SAOUHSC_02747		hypothetical protein	Tetrapyrroles	-0.74	-1.22	-0.66	-0.41	-	1.67	down	0.60	2.165E-02	2.33	down	0.43	1.180E-06	1.58	down	0.63	8.363E-01	1.33	down	0.75	1.000E+00
SAOUHSC_01121	Hla	alpha-hemolysin	Toxins and superantigens	n.d.	n.d.	1.71	1.85	▲									3.28	up	3.28	2.397E-02	3.61	up	3.61	4.069E-03
SAOUHSC_02706	Sbi	immunoglobulin G-binding protein Sbi	Toxins and superantigens	n.d.	-1.03	3.43	2.96	▲					2.04	down	0.49	5.235E-01	10.77	up	10.77	9.560E-13	7.76	up	7.76	4.050E-09
SAOUHSC_01714	GreA	transcription elongation factor GreA	Transcription	0.22	-0.49	-0.75	-0.58	▼	1.16	up	1.16	1.000E+00	1.41	down	0.71	1.000E+00	1.68	down	0.59	1.277E-02	1.49	down	0.67	1.000E+00
SAOUHSC_02362	Rho	transcription termination factor Rho	Transcription	-0.85	-0.05	-0.17	-0.37	-	1.81	down	0.55	1.650E-06	1.04	down	0.97	1.000E+00	1.13	down	0.89	1.000E+00	1.29	down	0.77	1.000E+00
SAOUHSC_01732	CymR	hypothetical protein	Transcription;Stress Response - no subcategory;Organic sulfur assimilation	-0.59	-0.80	-0.59	-0.78	▼	1.50	down	0.67	1.000E+00	1.74	down	0.57	1.000E+00	1.50	down	0.67	1.000E+00	1.72	down	0.58	1.558E-02
SAOUHSC_03006	Lip	lipase	Triacylglycerols	-0.42	n.d.	1.16	1.62	▲	1.34	down	0.75	1.000E+00					2.24	up	2.24	1.000E+00	3.06	up	3.06	2.493E-02
SAOUHSC_00625	MnhA2	putative monovalent cation/H+ antiporter subunit A	Uni- Sym- and Antiporters	1.00	1.24	0.72	0.20	-	2.00	up	2.00	1.237E-02	2.37	up	2.37	1.286E-03	1.64	up	1.64	1.000E+00	1.15	up	1.15	1.000E+00
SAOUHSC_00014		hypothetical protein		-1.27	0.05	-0.11	-0.30	-	2.41	down	0.41	1.479E-02	1.04	up	1.04	1.000E+00	1.08	down	0.93	1.000E+00	1.23	down	0.81	1.000E+00
SAOUHSC_00070	SarS	accessory regulator-like protein		2.35	2.05	1.20	1.33	▲	5.09	up	5.09	3.710E-09	4.13	up	4.13	3.990E-09	2.30	up	2.30	9.313E-04	2.52	up	2.52	3.490E-09
SAOUHSC_00135		hypothetical protein		-0.24	-0.42	n.d.	0.94	▲	1.18	down	0.84	1.000E+00	1.34	down	0.75	1.000E+00					1.92	up	1.92	4.759E-02
SAOUHSC_00173	AcpD	azoreductase		1.56	1.07	1.03	1.61	▲	2.95	up	2.95	6.310E-03	2.10	up	2.10	6.083E-02	2.05	up	2.05	1.000E+00	3.06	up	3.06	4.883E-04
SAOUHSC_00182		hypothetical protein		-1.71	0.07	0.53	-0.28	-	3.27	down	0.31	1.798E-02	1.05	up	1.05	1.000E+00	1.45	up	1.45	1.000E+00	1.21	down	0.82	1.000E+00
SAOUHSC_00197	FadD	hypothetical protein		n.d.	n.d.	1.68	2.06	▲									3.21	up	3.21	1.660E-22	4.18	up	4.18	1.070E-31
SAOUHSC_00198	FadE	hypothetical protein		n.d.	n.d.	1.51	1.85	▲									2.85	up	2.85	5.620E-11	3.61	up	3.61	3.470E-17
SAOUHSC_00199	FadX	hypothetical protein		n.d.	n.d.	1.53	1.46	▲									2.88	up	2.88	1.250E-01	2.75	up	2.75	1.808E-03
SAOUHSC_00221	TarJ'	alcohol dehydrogenase, zinc-containing		-1.44	-0.38	0.00	0.05	-	2.72	down	0.37	4.351E-03	1.30	down	0.77	1.000E+00	1.00	up	1.00	1.000E+00	1.03	up	1.03	1.000E+00
SAOUHSC_00244		hypothetical protein		-0.20	0.21	0.69	0.97	▲	1.15	down	0.87	1.000E+00	1.16	up	1.16	1.000E+00	1.62	up	1.62	1.000E+00	1.95	up	1.95	4.759E-02
SAOUHSC_00253		hypothetical protein		0.95	2.51	1.18	1.20	▲	1.93	up	1.93	1.978E-02	5.69	up	5.69	1.800E-08	2.27	up	2.27	1.794E-02	2.29	up	2.29	9.390E-06
SAOUHSC_00285		hypothetical protein		1.16	1.47	1.06	0.98	▲	2.23	up	2.23	1.327E-04	2.77	up	2.77	6.430E-07	2.08	up	2.08	1.109E-01	1.97	up	1.97	5.907E-04
SAOUHSC_00311		hypothetical protein		-5.31	n.d.	0.92	0.99	▲	39.66	down	0.03	5.375E-01					1.89	up	1.89	1.601E-01	1.98	up	1.98	9.330E-04
SAOUHSC_00319		hypothetical protein		0.48	0.24	0.60	0.76	▲	1.40	up	1.40	1.000E+00	1.18	up	1.18	1.000E+00	1.52	up	1.52	1.000E+00	1.69	up	1.69	1.373E-02
SAOUHSC_00356		hypothetical protein		2.61	1.85	0.61	1.01	▲	6.11	up	6.11	8.120E-17	3.61	up	3.61	4.560E-14	1.53	up	1.53	2.754E-01	2.02	up	2.02	1.230E-07
SAOUHSC_00357		hypothetical protein		-0.30	-0.69	-0.88	-1.17	▼	1.23	down	0.81	1.000E+00	1.62	down	0.62	8.293E-01	1.84	down	0.54	4.633E-01	2.25	down	0.44	2.641E-02
SAOUHSC_00362		hypothetical protein		-0.28	-0.49	-0.56	-1.04	▼	1.21	down	0.82	1.000E+00	1.41	down	0.71	1.000E+00	1.48	down	0.68	1.000E+00	2.05	down	0.49	9.480E-06
SAOUHSC_00371		hypothetical protein		-1.14	-1.78	0.98	1.19	▲	2.20	down	0.46	3.610E-12	3.44	down	0.29	2.040E-15	1.98	up	1.98	1.890E-07	2.28	up	2.28	1.440E-17
SAOUHSC_00427	Sle1	autolysin		1.23	1.18	-0.35	-1.24	▼	2.35	up	2.35	1.650E-06	2.27	up	2.27	1.800E-05	1.27	down	0.79	1.000E+00	2.36	down	0.42	3.074E-01
SAOUHSC_00428		hypothetical protein		-2.72	-2.59	-0.68	-1.10	▼	6.59	down	0.15	2.640E-06	6.02	down	0.17	2.460E-11	1.60	down	0.63	1.000E+00	2.14	down	0.47	5.550E-04
SAOUHSC_00502	CtsR	hypothetical protein		-0.53	-0.29	1.33	1.24	▲	1.44	down	0.69	1.000E+00	1.22	down	0.82	1.000E+00	2.51	up	2.51	2.960E-12	2.36	up	2.36	8.930E-07
SAOUHSC_00504	McsB	ATP:guanido phosphotransferase		0.18	0.67	0.56	1.02	▲	1.14	up	1.14	1.000E+00	1.59	up	1.59	1.995E-02	1.47	up	1.47	1.000E+00	2.03	up	2.03	1.750E-15
SAOUHSC_00533	HchA	chaperone protein HchA		0.79	0.08	-0.12	0.05	-	1.73	up	1.73	1.356E-04	1.06	up	1.06	1.000E+00	1.08	down	0.92	1.000E+00	1.04	up	1.04	1.000E+00
SAOUHSC_00534	AraB	ribulokinase		-1.07	-0.14	n.d.	n.d.	-	2.09	down	0.48	1.212E-02	1.10	down	0.91	1.000E+00								
SAOUHSC_00538		haloacid dehalogenase-like hydrolase		-0.66	-1.33	-0.69	-0.63	▼	1.58	down	0.63	7.587E-01	2.52	down	0.40	1.650E-07	1.61	down	0.62	5.760E-01	1.54	down	0.65	8.529E-01
SAOUHSC_00569		hypothetical protein		1.10	1.13	0.15	0.61	-	2.14	up	2.14	4.787E-04	2.18	up	2.18	3.340E-05	1.11	up	1.11	1.000E+00	1.52	up	1.52	1.000E+00
SAOUHSC_00617		hypothetical protein		-1.14	-1.47	n.d.	n.d.	-	2.20	down	0.45	6.820E-05	2.77	down	0.36	2.938E-01								
SAOUHSC_00634	MntC	ABC transporter substrate-binding protein		1.30	1.94	-0.66	-1.11	▼	2.46	up	2.46	6.080E-38	3.83	up	3.83	1.250E-43	1.58	down	0.63	1.000E+00	2.16	down	0.46	5.960E-13
SAOUHSC_00637	MntA	hypothetical protein		0.35	0.85	-0.14	-0.07	-	1.28	up	1.28	1.000E+00	1.80	up	1.80	3.450E-05	1.11	down	0.90	1.000E+00	1.05	down	0.95	1.000E+00
SAOUHSC_00647	AbcA	hypothetical protein		-0.04	-0.29	1.15	1.16	▲	1.03	down	0.97	1.000E+00	1.22	down	0.82	1.000E+00	2.22	up	2.22	1.010E-25	2.24	up	2.24	7.170E-37
SAOUHSC_00652	FhuA	iron compound ABC transporter ATP-binding protein		-2.11	-0.01	-0.57	-0.94	▼	4.32	down	0.23	4.750E-16	1.01	down	0.99	1.000E+00	1.49	down	0.67	1.000E+00	1.92	down	0.52	1.240E-07

The comparison of the early intracellular phase sample (8h p.i.) and late intracellular phase samples (24h and 32h p.i.) revealed proteins that were not significantly altered in their amount by using the non-adherent control as point of reference, because proteins were not detected in the non-adherent control sample or proteins were altered by opposing changes (early phase / late phase).

locus tag	protein symbol	description	theSEED functional category	log2 ratio OD=0.4	log2 ratio non-adherent	log2 ratio 24h p.i.	log2 ratio 32h p.i.	median over 24 and 32h ratio; median tendence - 1.5fc cutoff	median fold-change OD=0.4	median fold-change (direction) OD=0.4	median ratio over assays against 8h p.i. OD=0.4	p-value BH adjusted OD=0.4	median fold-change non-ad.	median fold-change (direction) non-ad.	median ratio over assays against 8h p.i. non-ad.	p-value BH adjusted non-ad.	median fold-change 24h p.i.	median fold-change (direction) 24h p.i.	median ratio over assays against 8h p.i. 24h p.i.	p-value BH adjusted 24h p.i.	median fold-change 32h p.i.	median fold-change (direction) 32h p.i.	median ratio over assays against 8h p.i. 32h p.i.	p-value BH adjusted 32h p.i.
SAOUHSC_00659		hypothetical protein		-1.34	-1.43	-0.52	-0.17	-	2.53	down	0.40	9.310E-06	2.69	down	0.37	2.396E-02	1.43	down	0.70	1.000E+00	1.13	down	0.89	1.000E+00
SAOUHSC_00694	MgrA	hypothetical protein		-0.40	-0.83	-0.85	-0.76	▼	1.32	down	0.76	1.000E+00	1.78	down	0.56	2.272E-01	1.80	down	0.56	1.290E-05	1.70	down	0.59	2.958E-03
SAOUHSC_00714	SaeS	sensor histidine kinase SaeS		-2.47	-1.31	-0.76	-1.14	▼	5.55	down	0.18	1.670E-12	2.48	down	0.40	3.380E-12	1.69	down	0.59	6.424E-01	2.20	down	0.45	6.500E-09
SAOUHSC_00715	SaeR	response regulator		-1.88	-1.78	-0.83	-0.99	▼	3.68	down	0.27	2.560E-10	3.44	down	0.29	7.700E-10	1.78	down	0.56	2.492E-04	1.98	down	0.51	3.740E-11
SAOUHSC_00717	SaeP	hypothetical protein		-4.39	-2.50	0.21	-0.76	-	21.02	down	0.05	1.282E-04	5.66	down	0.18	1.705E-04	1.16	up	1.16	1.000E+00	1.69	down	0.59	1.000E+00
SAOUHSC_00736		hypothetical protein		1.00	0.22	1.33	1.25	▲	1.99	up	1.99	6.637E-03	1.17	up	1.17	1.000E+00	2.52	up	2.52	1.074E-02	2.38	up	2.38	4.050E-09
SAOUHSC_00754		hypothetical protein		0.97	0.90	-0.14	-0.28	-	1.95	up	1.95	8.910E-05	1.87	up	1.87	3.830E-04	1.10	down	0.91	1.000E+00	1.21	down	0.82	1.000E+00
SAOUHSC_00792		hypothetical protein		0.79	0.56	0.65	0.86	▲	1.73	up	1.73	2.990E-07	1.48	up	1.48	7.493E-01	1.57	up	1.57	3.918E-01	1.82	up	1.82	1.260E-09
SAOUHSC_00834		thioredoxin		-0.37	-1.02	-0.70	-0.65	▼	1.29	down	0.77	1.000E+00	2.03	down	0.49	2.598E-04	1.62	down	0.62	3.796E-01	1.56	down	0.64	5.517E-01
SAOUHSC_00850	SufU	hypothetical protein		1.33	0.33	0.28	0.40	-	2.51	up	2.51	1.680E-12	1.25	up	1.25	1.000E+00	1.21	up	1.21	1.000E+00	1.32	up	1.32	1.000E+00
SAOUHSC_00863		hypothetical protein		-0.31	-1.07	-0.66	-0.83	▼	1.24	down	0.81	1.000E+00	2.09	down	0.48	1.205E-02	1.59	down	0.63	1.000E+00	1.78	down	0.56	1.339E-02
SAOUHSC_00934	SpxA	transcriptional regulator Spx		-0.05	-1.25	-0.40	-0.65	-	1.03	down	0.97	1.000E+00	2.38	down	0.42	1.010E-12	1.32	down	0.76	1.000E+00	1.57	down	0.64	2.457E-01
SAOUHSC_00972		hypothetical protein		1.36	0.90	0.37	0.80	▲	2.56	up	2.56	7.870E-09	1.87	up	1.87	2.160E-05	1.30	up	1.30	1.000E+00	1.74	up	1.74	3.668E-03
SAOUHSC_01050		hypothetical protein		0.76	0.64	-0.16	-0.08	-	1.70	up	1.70	2.965E-03	1.56	up	1.56	3.741E-02	1.12	down	0.90	1.000E+00	1.06	down	0.95	1.000E+00
SAOUHSC_01138		hypothetical protein		0.01	-0.76	1.02	1.23	▲	1.01	up	1.01	1.000E+00	1.69	down	0.59	9.545E-01	2.03	up	2.03	8.980E-07	2.35	up	2.35	9.480E-10
SAOUHSC_01154	SepF	hypothetical protein		0.07	-0.04	-1.32	-1.33	▼	1.05	up	1.05	1.000E+00	1.03	down	0.97	1.000E+00	2.49	down	0.40	2.020E-09	2.52	down	0.40	3.630E-13
SAOUHSC_01185		ribosomal RNA large subunit methyltransferase N		1.39	2.03	-0.52	-0.66	-	2.62	up	2.62	3.680E-11	4.08	up	4.08	2.690E-12	1.43	down	0.70	1.000E+00	1.58	down	0.63	4.540E-01
SAOUHSC_01222	TopA	DNA topoisomerase I		0.62	0.80	-0.07	0.07	-	1.54	up	1.54	3.958E-01	1.75	up	1.75	4.130E-06	1.05	down	0.95	1.000E+00	1.05	up	1.05	1.000E+00
SAOUHSC_01263	Rny| cvfA	phosphodiesterase		0.70	0.60	0.14	0.09	-	1.63	up	1.63	3.662E-03	1.51	up	1.51	1.721E-01	1.10	up	1.10	1.000E+00	1.06	up	1.06	1.000E+00
SAOUHSC_01264		hypothetical protein		0.97	0.17	-0.37	-0.23	-	1.95	up	1.95	1.304E-03	1.13	up	1.13	1.000E+00	1.29	down	0.78	1.000E+00	1.18	down	0.85	1.000E+00
SAOUHSC_01265		hypothetical protein		0.69	0.51	0.41	0.45	-	1.61	up	1.61	2.055E-02	1.43	up	1.43	3.776E-01	1.33	up	1.33	1.000E+00	1.36	up	1.36	1.000E+00
SAOUHSC_01324		hypothetical protein		-1.59	-1.28	-0.13	-0.28	-	3.00	down	0.33	3.820E-10	2.43	down	0.41	7.070E-07	1.09	down	0.92	1.000E+00	1.22	down	0.82	1.000E+00
SAOUHSC_01349		hypothetical protein		-0.84	-1.48	-0.66	-0.59	▼	1.79	down	0.56	8.760E-06	2.80	down	0.36	8.990E-11	1.58	down	0.63	4.846E-01	1.50	down	0.66	1.000E+00
SAOUHSC_01354	AlsT	sodium:alanine symporter family protein		0.64	0.85	0.54	0.58	-	1.56	up	1.56	8.554E-01	1.80	up	1.80	6.076E-03	1.46	up	1.46	1.000E+00	1.49	up	1.49	1.000E+00
SAOUHSC_01392		ABC transporter ATP-binding protein		0.51	0.77	-0.06	0.11	-	1.43	up	1.43	1.000E+00	1.70	up	1.70	3.580E-09	1.04	down	0.96	1.000E+00	1.08	up	1.08	1.000E+00
SAOUHSC_01415		hypothetical protein		-0.10	-0.70	0.36	0.66	-	1.07	down	0.93	1.000E+00	1.63	down	0.61	1.000E+00	1.29	up	1.29	1.000E+00	1.58	up	1.58	9.430E-04
SAOUHSC_01419	ArlS	hypothetical protein		-0.76	-0.39	0.90	n.d.	▲	1.69	down	0.59	3.508E-02	1.31	down	0.77	1.000E+00	1.86	up	1.86	3.929E-01				
SAOUHSC_01425		hypothetical protein		0.91	-0.12	-0.19	0.16	-	1.89	up	1.89	1.439E-03	1.09	down	0.92	1.000E+00	1.14	down	0.88	1.000E+00	1.12	up	1.12	1.000E+00
SAOUHSC_01427	CtpA	hypothetical protein		-1.00	-0.81	-0.49	-0.78	▼	2.00	down	0.50	2.380E-09	1.76	down	0.57	3.651E-04	1.41	down	0.71	1.000E+00	1.72	down	0.58	4.430E-02
SAOUHSC_01429		hypothetical protein		-2.02	-1.60	-1.87	-2.23	▼	4.06	down	0.25	1.068E-03	3.04	down	0.33	1.860E-05	3.65	down	0.27	2.971E-03	4.70	down	0.21	2.493E-02
SAOUHSC_01476		hypothetical protein		-0.01	-0.46	-1.29	-1.51	▼	1.01	down	0.99	1.000E+00	1.37	down	0.73	1.000E+00	2.45	down	0.41	3.929E-01	2.84	down	0.35	2.493E-02
SAOUHSC_01610		hypothetical protein		-0.20	-0.01	-0.89	-1.06	▼	1.15	down	0.87	1.000E+00	1.01	down	0.99	1.000E+00	1.86	down	0.54	1.922E-01	2.08	down	0.48	2.460E-06
SAOUHSC_01627		hypothetical protein		-0.50	-0.09	-0.54	-0.74	▼	1.42	down	0.71	1.000E+00	1.07	down	0.94	1.000E+00	1.46	down	0.69	1.000E+00	1.67	down	0.60	2.665E-04
SAOUHSC_01666	GlyS	glycyl-tRNA synthetase		0.75	0.07	-0.05	0.04	-	1.68	up	1.68	7.360E-10	1.05	up	1.05	1.000E+00	1.04	down	0.96	1.000E+00	1.03	up	1.03	1.000E+00
SAOUHSC_01716		hypothetical protein		0.82	1.57	n.d.	-0.57	-	1.76	up	1.76	7.455E-02	2.97	up	2.97	3.560E-05					1.49	down	0.67	1.000E+00
SAOUHSC_01717		hypothetical protein		1.08	1.38	-0.61	-0.79	▼	2.11	up	2.11	1.186E-02	2.61	up	2.61	2.450E-08	1.52	down	0.66	1.000E+00	1.73	down	0.58	1.000E+00
SAOUHSC_01729		hypothetical protein		2.32	0.08	0.45	-0.21	-	4.98	up	4.98	5.934E-04	1.06	up	1.06	1.000E+00	1.37	up	1.37	1.000E+00	1.16	down	0.87	1.000E+00
SAOUHSC_01730	CsbD	hypothetical protein		1.79	0.90	0.53	0.59	-	3.45	up	3.45	2.640E-06	1.87	up	1.87	1.063E-03	1.45	up	1.45	1.000E+00	1.50	up	1.50	1.000E+00
SAOUHSC_01761		hypothetical protein		-2.98	-3.01	-0.82	-1.48	▼	7.88	down	0.13	9.310E-06	8.07	down	0.12	5.370E-08	1.77	down	0.56	1.665E-02	2.79	down	0.36	9.390E-06
SAOUHSC_01786	InfC	translation initiation factor IF-3		0.61	1.02	-0.08	0.04	-	1.52	up	1.52	2.629E-01	2.03	up	2.03	1.700E-09	1.06	down	0.94	1.000E+00	1.03	up	1.03	1.000E+00
SAOUHSC_01787	LysP	hypothetical protein		-2.66	-1.92	-0.50	-0.86	▼	6.33	down	0.16	9.310E-06	3.78	down	0.26	3.740E-09	1.41	down	0.71	1.000E+00	1.81	down	0.55	4.834E-02
SAOUHSC_01816	PepQ	hypothetical protein		-1.49	-1.67	-0.69	-0.60	▼	2.81	down	0.36	6.790E-16	3.18	down	0.31	4.500E-13	1.61	down	0.62	1.000E+00	1.52	down	0.66	1.000E+00
SAOUHSC_01838		hypothetical protein		-2.92	-2.46	-1.57	-1.95	▼	7.55	down	0.13	8.870E-23	5.50	down	0.18	3.980E-28	2.96	down	0.34	2.480E-06	3.86	down	0.26	3.900E-21
SAOUHSC_01854		hypothetical protein		0.73	0.45	0.27	0.44	-	1.66	up	1.66	3.050E-06	1.37	up	1.37	1.000E+00	1.21	up	1.21	1.000E+00	1.35	up	1.35	1.000E+00
SAOUHSC_01863		hypothetical protein		-0.80	-0.46	0.15	-0.41	-	1.74	down	0.58	3.024E-02	1.37	down	0.73	1.000E+00	1.11	up	1.11	1.000E+00	1.33	down	0.75	1.000E+00
SAOUHSC_01868		dipeptidase PepV		-0.71	-1.19	-0.72	-0.54	▼	1.63	down	0.61	7.027E-02	2.29	down	0.44	1.440E-17	1.64	down	0.61	1.000E+00	1.46	down	0.69	1.000E+00
SAOUHSC_01869		hypothetical protein		0.69	0.10	0.11	0.38	-	1.62	up	1.62	2.162E-03	1.07	up	1.07	1.000E+00	1.08	up	1.08	1.000E+00	1.30	up	1.30	1.000E+00
SAOUHSC_01877		hypothetical protein		0.72	1.11	n.d.	n.d.	-	1.65	up	1.65	4.365E-01	2.16	up	2.16	1.802E-03								
SAOUHSC_01879	Rot	virulence factor regulator protein		0.52	1.49	0.46	0.47	-	1.44	up	1.44	1.000E+00	2.80	up	2.80	1.700E-08	1.38	up	1.38	1.000E+00	1.39	up	1.39	1.000E+00
SAOUHSC_01978		hypothetical protein		0.81	0.73	0.39	0.43	-	1.75	up	1.75	8.980E-03	1.66	up	1.66	1.276E-02	1.31	up	1.31	1.000E+00	1.35	up	1.35	1.000E+00
SAOUHSC_02013		hypothetical protein		1.84	0.68	1.16	1.50	▲	3.59	up	3.59	8.080E-14	1.61	up	1.61	1.676E-02	2.23	up	2.23	2.940E-08	2.83	up	2.83	5.090E-14
SAOUHSC_02096		hypothetical protein		-1.50	-1.32	n.d.	-1.08	▼	2.82	down	0.35	1.690E-03	2.50	down	0.40	4.755E-02					2.12	down	0.47	8.476E-01
SAOUHSC_02098	VraR	DNA-binding response regulator VraR		-1.38	-2.09	-0.87	-0.96	▼	2.61	down	0.38	8.020E-22	4.27	down	0.23	5.030E-24	1.82	down	0.55	2.844E-03	1.94	down	0.51	8.880E-14
SAOUHSC_02099	VraS	histidine kinase		-1.76	-1.54	-0.29	-0.66	-	3.39	down	0.29	8.580E-07	2.90	down	0.34	1.680E-09	1.22	down	0.82	1.000E+00	1.58	down	0.63	1.754E-01
SAOUHSC_02100	VraT	hypothetical protein		-1.23	-1.47	-0.51	-0.28	-	2.35	down	0.43	3.060E-05	2.78	down	0.36	4.420E-05	1.42	down	0.70	1.000E+00	1.21	down	0.83	1.000E+00
SAOUHSC_02108	FtnA	ferritin		1.32	0.77	0.85	1.00	▲	2.50	up	2.50	1.310E-12	1.71	up	1.71	3.802E-02	1.80	up	1.80	3.578E-03	2.00	up	2.00	2.820E-07
SAOUHSC_02150		hypothetical protein		-0.27	-1.19	0.29	0.63	-	1.21	down	0.83	1.000E+00	2.29	down	0.44	1.300E-05	1.22	up	1.22	1.000E+00	1.54	up	1.54	1.478E-01
SAOUHSC_02151	PmtD	hypothetical protein		-0.70	-1.78	0.90	0.64	▲	1.63	down	0.61	7.483E-01	3.43	down	0.29	1.889E-03	1.86	up	1.86	6.686E-02	1.55	up	1.55	2.589E-01
SAOUHSC_02152	PmtC	ABC transporter ATP-binding protein		-0.84	-1.15	0.79	0.83	▲	1.79	down	0.56	1.318E-03	2.21	down	0.45	2.660E-08	1.73	up	1.73	6.630E-08	1.78	up	1.78	8.050E-11
SAOUHSC_02155		hypothetical protein		-0.74	-1.80	-0.33	-0.50	-	1.67	down	0.60	1.000E+00	3.48	down	0.29	4.633E-02	1.26	down	0.80	1.000E+00	1.41	down	0.71	1.000E+00
SAOUHSC_02243	LukH	hypothetical protein		n.d.	-4.08	-0.62	-1.36	▼					16.92	down	0.06	1.889E-03	1.53	down	0.65	1.000E+00	2.57	down	0.39	9.570E-12
SAOUHSC_02258		hypothetical protein		0.69	0.89	1.15	1.35	▲	1.62	up	1.62	8.672E-01	1.85	up	1.85	7.103E-02	2.22	up	2.22	3.633E-02	2.55	up	2.55	3.470E-05
SAOUHSC_02264	AgrC	accessory gene regulator protein C		-0.12	-0.13	0.87	0.66	▲	1.09	down	0.92	1.000E+00	1.09	down	0.91	1.000E+00	1.83	up	1.83	2.850E-02	1.58	up	1.58	3.723E-01
SAOUHSC_02265	AgrA	accessory gene regulator protein A		-0.80	-1.13	1.06	1.03	▲	1.74	down	0.58	1.939E-02	2.18	down	0.46	1.680E-08	2.08	up	2.08	1.960E-05	2.04	up	2.04	7.120E-10
SAOUHSC_02315	KdpE	DNA-binding response regulator		1.84	1.90	1.47	2.25	▲	3.58	up	3.58	2.449E-04	3.74	up	3.74	3.560E-05	2.78	up	2.78	6.530E-01	4.74	up	4.74	1.339E-02

The comparison of the early intracellular phase sample (8h p.i.) and late intracellular phase samples (24h and 32h p.i.) revealed proteins that were not significantly altered in their amount by using the non-adherent control as point of reference, because proteins were not detected in the non-adherent control sample or proteins were altered by opposing changes (early phase / late phase).

locus tag	protein symbol	description	theSEED functional category	log2 ratio OD=0.4	log2 ratio non-adherent	log2 ratio 24h p.i.	log2 ratio 32h p.i.	median over 24 and 32h ratio; median tendence - 1.5fc cutoff	median fold-change OD=0.4	median fold-change (direction) OD=0.4	median ratio over assays against 8h p.i. OD=0.4	p-value BH adjusted OD=0.4	median fold-change non-ad.	median fold-change (direction) non-ad.	median ratio over assays against 8h p.i. non-ad.	p-value BH adjusted non-ad.	median fold-change 24h p.i.	median fold-change (direction) 24h p.i.	median ratio over assays against 8h p.i. 24h p.i.	p-value BH adjusted 24h p.i.	median fold-change 32h p.i.	median fold-change (direction) 32h p.i.	median ratio over assays against 8h p.i. 32h p.i.	p-value BH adjusted 32h p.i.
SAOUHSC_02364		hypothetical protein		-1.03	-1.10	n.d.	-0.88	▼	2.05	down	0.49	3.021E-02	2.15	down	0.47	6.943E-01					1.84	down	0.54	1.000E+00
SAOUHSC_02372		hypothetical protein		-0.20	0.05	-1.55	-1.70	▼	1.15	down	0.87	1.000E+00	1.03	up	1.03	1.000E+00	2.92	down	0.34	9.645E-02	3.24	down	0.31	2.493E-02
SAOUHSC_02382		hypothetical protein		-0.82	-0.82	-0.39	-0.12	-	1.76	down	0.57	4.460E-02	1.77	down	0.56	4.936E-01	1.31	down	0.76	1.000E+00	1.09	down	0.92	1.000E+00
SAOUHSC_02387		hypothetical protein		1.37	0.45	0.78	1.09	▲	2.58	up	2.58	4.250E-07	1.37	up	1.37	1.000E+00	1.72	up	1.72	1.882E-01	2.13	up	2.13	1.930E-06
SAOUHSC_02420	SdrM	hypothetical protein		-0.28	0.01	0.79	0.90	▲	1.22	down	0.82	1.000E+00	1.01	up	1.01	1.000E+00	1.72	up	1.72	1.503E-01	1.87	up	1.87	3.875E-03
SAOUHSC_02441	Asp23	alkaline shock protein 23		1.33	0.84	1.11	1.52	▲	2.51	up	2.51	3.660E-09	1.79	up	1.79	4.384E-03	2.15	up	2.15	3.830E-06	2.86	up	2.86	3.500E-14
SAOUHSC_02442		hypothetical protein		1.00	0.46	1.13	1.19	▲	2.00	up	2.00	6.297E-03	1.37	up	1.37	1.000E+00	2.18	up	2.18	7.280E-02	2.28	up	2.28	2.380E-05
SAOUHSC_02443	AmaP	hypothetical protein		1.75	1.38	1.30	1.67	▲	3.37	up	3.37	4.500E-12	2.60	up	2.60	5.410E-10	2.47	up	2.47	4.000E-11	3.17	up	3.17	2.160E-09
SAOUHSC_02448		hypothetical protein		-0.60	-0.14	-0.86	-0.87	▼	1.51	down	0.66	1.000E+00	1.10	down	0.91	1.000E+00	1.82	down	0.55	6.063E-02	1.83	down	0.55	7.128E-03
SAOUHSC_02554	FhuD2	hypothetical protein		-1.95	-0.21	-0.46	-0.64	-	3.87	down	0.26	5.250E-36	1.16	down	0.86	1.000E+00	1.37	down	0.73	1.000E+00	1.56	down	0.64	1.000E+00
SAOUHSC_02566	SarR	hypothetical protein		-0.75	0.05	-0.24	-0.14	-	1.68	down	0.60	2.965E-03	1.04	up	1.04	1.000E+00	1.18	down	0.85	1.000E+00	1.10	down	0.91	1.000E+00
SAOUHSC_02568		hypothetical protein		-0.62	-1.48	-0.25	0.19	-	1.54	down	0.65	1.000E+00	2.79	down	0.36	3.150E-09	1.19	down	0.84	1.000E+00	1.14	up	1.14	1.000E+00
SAOUHSC_02571	SsaA	secretory antigen		0.25	0.74	2.54	1.86	▲	1.19	up	1.19	1.000E+00	1.67	up	1.67	3.794E-01	5.80	up	5.80	6.500E-11	3.63	up	3.63	2.940E-09
SAOUHSC_02604		hypothetical protein		1.72	0.88	1.05	1.23	▲	3.30	up	3.30	2.420E-13	1.84	up	1.84	3.935E-02	2.08	up	2.08	1.730E-05	2.35	up	2.35	1.270E-08
SAOUHSC_02619		hypothetical protein		0.64	1.17	n.d.	0.82	▲	1.56	up	1.56	1.000E+00	2.25	up	2.25	1.058E-02					1.77	up	1.77	1.000E+00
SAOUHSC_02650		hypothetical protein		0.41	0.00	0.51	0.93	▲	1.33	up	1.33	1.000E+00	1.00	up	1.00	1.000E+00	1.42	up	1.42	1.000E+00	1.91	up	1.91	7.430E-05
SAOUHSC_02665		hypothetical protein		1.54	0.58	0.98	1.08	▲	2.90	up	2.90	4.370E-11	1.49	up	1.49	2.625E-01	1.97	up	1.97	7.140E-06	2.12	up	2.12	1.390E-08
SAOUHSC_02668		hypothetical protein		-0.23	0.39	-0.78	-1.06	▼	1.17	down	0.85	1.000E+00	1.31	up	1.31	1.000E+00	1.72	down	0.58	6.573E-03	2.09	down	0.48	1.100E-06
SAOUHSC_02669	SarZ	hypothetical protein		-0.98	-0.78	-0.37	-0.26	-	1.97	down	0.51	4.700E-07	1.72	down	0.58	1.000E+00	1.30	down	0.77	1.000E+00	1.20	down	0.83	1.000E+00
SAOUHSC_02690		hypothetical protein		0.12	0.87	0.65	0.50	-	1.09	up	1.09	1.000E+00	1.83	up	1.83	2.474E-04	1.56	up	1.56	1.000E+00	1.42	up	1.42	1.000E+00
SAOUHSC_02724		hypothetical protein		-1.81	-2.46	-0.23	-0.47	-	3.51	down	0.28	1.030E-10	5.49	down	0.18	7.720E-09	1.17	down	0.85	1.000E+00	1.39	down	0.72	1.000E+00
SAOUHSC_02727		hypothetical protein		-1.19	-0.49	-0.16	-0.25	-	2.28	down	0.44	1.400E-06	1.40	down	0.71	1.000E+00	1.11	down	0.90	1.000E+00	1.19	down	0.84	1.000E+00
SAOUHSC_02761		hypothetical protein		-0.89	-0.57	-0.49	-0.86	▼	1.86	down	0.54	1.625E-04	1.49	down	0.67	1.000E+00	1.41	down	0.71	1.000E+00	1.82	down	0.55	1.820E-03
SAOUHSC_02811	RelP	hypothetical protein		-1.38	-1.10	-1.22	-1.21	▼	2.61	down	0.38	7.650E-08	2.15	down	0.47	5.370E-08	2.33	down	0.43	5.356E-02	2.32	down	0.43	3.056E-03
SAOUHSC_02820		hypothetical protein		1.45	2.08	0.88	0.91	▲	2.72	up	2.72	4.660E-06	4.22	up	4.22	1.850E-10	1.84	up	1.84	7.752E-02	1.87	up	1.87	3.654E-04
SAOUHSC_02829	Frp	NAD(P)H-flavin oxidoreductase		0.70	0.04	1.42	1.67	▲	1.62	up	1.62	2.438E-01	1.03	up	1.03	1.000E+00	2.67	up	2.67	3.320E-09	3.19	up	3.19	9.180E-16
SAOUHSC_02846		hypothetical protein		-1.26	-1.38	0.56	-0.96	-	2.40	down	0.42	5.220E-08	2.60	down	0.38	2.426E-02	1.47	up	1.47	1.000E+00	1.95	down	0.51	1.526E-01
SAOUHSC_02862	ClpL	ATP-dependent Clp protease, ATP-binding subunit ClpC		-0.46	-1.09	0.21	0.46	-	1.38	down	0.73	1.000E+00	2.13	down	0.47	1.019E-02	1.15	up	1.15	1.000E+00	1.38	up	1.38	1.000E+00
SAOUHSC_02866		hypothetical protein		2.01	2.10	n.d.	0.96	▲	4.03	up	4.03	7.290E-07	4.29	up	4.29	7.070E-07					1.95	up	1.95	1.327E-04
SAOUHSC_02886		hypothetical protein		1.73	1.67	n.d.	n.d.	-	3.33	up	3.33	3.273E-03	3.17	up	3.17	2.617E-04								
SAOUHSC_02887	IsaA	immunodominant antigen A		0.50	-0.38	0.81	0.59	▲	1.41	up	1.41	1.000E+00	1.30	down	0.77	1.000E+00	1.75	up	1.75	2.490E-03	1.50	up	1.50	1.000E+00
SAOUHSC_02899		hypothetical protein		0.54	0.19	0.72	0.90	▲	1.46	up	1.46	1.000E+00	1.14	up	1.14	1.000E+00	1.65	up	1.65	2.744E-02	1.86	up	1.86	1.050E-09
SAOUHSC_02900		hypothetical protein		1.15	0.30	0.91	1.16	▲	2.22	up	2.22	7.390E-15	1.23	up	1.23	1.000E+00	1.88	up	1.88	3.980E-09	2.23	up	2.23	1.790E-15
SAOUHSC_02910		hypothetical protein		-0.94	-1.11	-0.65	-0.64	▼	1.92	down	0.52	1.520E-01	2.16	down	0.46	3.441E-03	1.57	down	0.64	1.000E+00	1.56	down	0.64	1.000E+00
SAOUHSC_02945		precorrin-2 dehydrogenase		-0.78	-0.50	n.d.	0.14	-	1.72	down	0.58	3.743E-02	1.42	down	0.71	1.000E+00					1.10	up	1.10	1.000E+00
SAOUHSC_02955	NsaS	nisin susceptibility-associated sensor histidine kinase		-0.95	-0.25	n.d.	-0.68	▼	1.93	down	0.52	4.280E-02	1.19	down	0.84	1.000E+00					1.60	down	0.62	1.000E+00
SAOUHSC_02961		hypothetical protein		-0.90	-0.68	-0.08	-0.24	-	1.87	down	0.53	1.304E-02	1.60	down	0.62	1.000E+00	1.05	down	0.95	1.000E+00	1.18	down	0.85	1.000E+00
SAOUHSC_02972	IsaB	immunodominant antigen B		-3.09	-2.44	1.24	1.53	▲	8.51	down	0.12	1.690E-03	5.42	down	0.18	4.633E-02	2.36	up	2.36	3.410E-06	2.88	up	2.88	1.020E-12
SAOUHSC_02978		phage infection protein		0.45	0.73	-0.33	-0.29	-	1.37	up	1.37	1.000E+00	1.66	up	1.66	1.350E-07	1.26	down	0.80	1.000E+00	1.23	down	0.82	1.000E+00
SAOUHSC_02979		N-acetylmuramoyl-L-alanine amidase		-0.12	0.53	1.36	0.82	▲	1.08	down	0.92	1.000E+00	1.44	up	1.44	1.000E+00	2.57	up	2.57	2.013E-02	1.76	up	1.76	2.076E-01
SAOUHSC_03022		hypothetical protein		1.09	0.69	1.37	1.65	▲	2.12	up	2.12	3.540E-06	1.61	up	1.61	3.547E-01	2.58	up	2.58	1.469E-04	3.13	up	3.13	3.470E-12
SAOUHSC_03034		hypothetical protein		-0.41	-1.62	0.80	0.80	▲	1.32	down	0.76	1.000E+00	3.07	down	0.33	8.400E-07	1.74	up	1.74	5.051E-02	1.74	up	1.74	8.000E-06

log_2-ratios

-2 0 2

supplemental table S6: Detected proteins with at least 2 peptides: murine pneumonia model and S9 cell line infection model (log2 ratios to controls [non-adherent or pre-infection])

locus tag	protein symbol	description	theSEED functional category	S9 cell line infection model				murine pneumonia model			median over 8,24 and 32h ratio	
				exp.	8h	24h	32h	8h	24h	32h	median tendence (S9) - 1.5fc cutoff	median tendence (mice) - 1.5fc cutoff
SAOUHSC_00069	Spa	protein A	Adhesion	0.56	-3.90	-0.79	-2.21	-5.33	-3.44	n.d.	▼	▼
SAOUHSC_00487	HslO	Hsp33-like chaperonin	Adhesion	0.23	-0.15	-0.97	-0.85	-0.26	-0.82	-0.69	▼	▼
SAOUHSC_01501	EbpS	elastin binding protein	Adhesion	0.12	0.47	0.47	0.62	-0.37	-0.30	-0.34	–	–
SAOUHSC_02161	Eap|map	MHC class II analog protein	Adhesion	n.d.	n.d.	n.d.	n.d.	n.d.	n.d.	2.50	n.d.	▲
SAOUHSC_02982		hypothetical protein	Adhesion	0.40	0.16	-1.35	2.91	n.d.	n.d.	1.03	–	▲
SAOUHSC_00836	GcvH	glycine cleavage system protein H	Alanine, serine, and glycine;Alanine, serine, and glycine	0.32	0.23	0.51	0.70	0.80	2.23	2.20	–	▲
SAOUHSC_01633	GcvPA	glycine dehydrogenase subunit 1	Alanine, serine, and glycine;Alanine, serine, and glycine	-0.57	0.34	0.55	0.61	-0.36	-0.10	0.42	–	–
SAOUHSC_01634	GcvT	glycine cleavage system aminomethyltransferase T	Alanine, serine, and glycine;Alanine, serine, and glycine	-0.49	0.27	0.60	0.81	0.24	0.31	0.29	▲	–
SAOUHSC_02354	GlyA	serine hydroxymethyltransferase	Alanine, serine, and glycine;Alanine, serine, and glycine;Folate and pterines;One-carbon Metabolism;Stress Response - no subcategory;Alanine, serine, and glycine;Alanine, serine, and glycine	-0.39	0.11	-0.16	-0.21	0.61	0.06	-0.29	–	–
SAOUHSC_02305	Alr	alanine racemase	Alanine, serine, and glycine;Central carbohydrate metabolism;RNA processing and modification	-0.16	-0.32	-1.20	-0.89	1.19	3.52	3.88	▼	▲
SAOUHSC_00532	Kbl	2-amino-3-ketobutyrate coenzyme A ligase	Alanine, serine, and glycine;Lysine, threonine, methionine, and cysteine;Alanine, serine, and glycine	0.07	0.42	0.39	0.79	-0.03	-0.14	0.23	–	–
SAOUHSC_00009	SerS	seryl-tRNA synthetase	Alanine, serine, and glycine;Protein biosynthesis	0.25	0.69	0.15	0.16	-0.96	-0.27	-0.77	–	▼
SAOUHSC_01617	ArgR	arginine repressor	Arginine; urea cycle, polyamines;Arginine; urea cycle, polyamines;Arginine; urea cycle, polyamines	0.23	0.79	0.45	0.58	-1.13	-0.40	-0.48	▲	–
SAOUHSC_00894	RocD	ornithine--oxo-acid transaminase	Arginine; urea cycle, polyamines;Arginine; urea cycle, polyamines;Proline and 4-hydroxyproline	0.58	1.59	2.64	2.91	0.25	0.89	1.18	▲	▲
SAOUHSC_01702	MtnN	MTA/SAH nucleosidase	Arginine; urea cycle, polyamines;Lysine, threonine, methionine, and cysteine;Lysine, threonine, methionine, and cysteine	0.45	0.30	-0.30	-0.09	-0.91	1.16	-0.75	–	▼
SAOUHSC_01483	AroC	chorismate synthase	Aromatic amino acids and derivatives;Aromatic amino acids and derivatives	0.11	-0.08	0.00	-0.01	-0.07	0.08	-0.79	–	–
SAOUHSC_01852	AroA2	bifunctional 3-deoxy-7-phosphoheptulonate synthase/chorismate mutase	Aromatic amino acids and derivatives;Aromatic amino acids and derivatives;Aromatic amino acids and derivatives	0.35	0.39	0.17	0.31	-0.39	-0.36	-0.33	–	–
SAOUHSC_02340	AtpC	F0F1 ATP synthase subunit epsilon	ATP synthases	-0.19	0.25	-0.27	-0.32	0.89	0.70	1.20	–	▲
SAOUHSC_02341	AtpD	F0F1 ATP synthase subunit beta	ATP synthases	-0.49	0.28	-0.12	-0.21	-0.31	-0.05	-0.69	–	–
SAOUHSC_02343	AtpG	F0F1 ATP synthase subunit gamma	ATP synthases	-0.55	0.17	-0.34	-0.26	0.01	0.07	0.00	–	–
SAOUHSC_02345	AtpA	F0F1 ATP synthase subunit alpha	ATP synthases	-0.47	0.18	-0.26	-0.41	-0.15	-0.10	-0.28	–	–
SAOUHSC_02346	AtpH	F0F1 ATP synthase subunit delta	ATP synthases	-0.18	0.39	-0.17	-0.22	-1.01	-0.91	-1.20	–	▼
SAOUHSC_02347	AtpF	F0F1 ATP synthase subunit B	ATP synthases	-0.43	0.35	0.40	-0.06	-0.41	-0.29	-0.39	–	–
SAOUHSC_01809	AccD	acetyl-CoA carboxylase subunit beta	Bacteriocins, ribosomally synthesized antibacterial peptides;Fatty acids	0.06	-0.28	-0.43	-0.39	1.59	0.03	-0.26	–	–
SAOUHSC_01014	PurF	amidophosphoribosyltransferase	Bacteriocins, ribosomally synthesized antibacterial peptides;Purines	-0.07	0.54	-1.00	-0.81	-0.23	0.51	1.02	▼	–
SAOUHSC_02714	BioB	biotin synthase	Biotin	1.45	0.57	-2.17	-1.84	-2.89	-1.90	-2.63	▼	▼
SAOUHSC_02715	BioA	adenosylmethionine-8-amino-7-oxononanoate aminotransferase	Biotin	1.07	0.89	0.64	0.36	-0.27	-0.08	-0.18	▲	–
SAOUHSC_02716	BioD	dethiobiotin synthase	Biotin	1.06	0.72	-0.11	-0.37	0.03	-2.46	n.d.	–	▼
SAOUHSC_00336	Thl	acetyl-CoA acetyltransferase	Biotin;Fermentation;Isoprenoids;Isoprenoids;Fatty Acids, Lipids, and Isoprenoids - no subcategory;One-carbon Metabolism;Fermentation;Branched-chain amino acids	-0.14	0.81	-0.11	-0.18	-0.33	-0.23	0.06	–	–
SAOUHSC_01612	BfmBAB	2-oxoisovalerate dehydrogenase, E1 component, beta subunit	Branched-chain amino acids;Central carbohydrate metabolism;Branched-chain amino acids	-0.34	-0.08	-0.22	-0.23	0.21	0.22	0.08	–	–
SAOUHSC_01613	BfmBAA	2-oxoisovalerate dehydrogenase, E1 component subunit alpha	Branched-chain amino acids;Central carbohydrate metabolism;Branched-chain amino acids	-0.43	-0.19	-0.65	-0.58	-0.35	-0.18	-0.83	▼	–
SAOUHSC_00471	GlmU	bifunctional N-acetylglucosamine-1-phosphate uridyltransferase/glucosamine-1-phosphate acetyltransferase	Capsular and extracellular polysacchrides;Cell Wall and Capsule - no subcategory;Cell Wall and Capsule - no subcategory	-0.30	-0.02	-0.73	-0.62	-1.24	0.04	1.01	▼	–
SAOUHSC_02399	GlmS	glucosamine--fructose-6-phosphate aminotransferase	Capsular and extracellular polysacchrides;Cell Wall and Capsule - no subcategory;One-carbon Metabolism	-0.16	-0.38	-0.47	-0.51	0.55	0.18	1.18	–	–
SAOUHSC_01149	FtsA	cell division protein	Cell Division and Cell Cycle - no subcategory	0.25	0.23	0.07	0.15	-0.21	-0.28	-0.16	–	–
SAOUHSC_01150	FtsZ	cell division protein FtsZ	Cell Division and Cell Cycle - no subcategory	0.13	0.02	-0.28	-0.34	0.06	0.19	0.03	–	–
SAOUHSC_01158		hypothetical protein	Cell Division and Cell Cycle - no subcategory	0.30	0.07	0.07	0.32	0.10	-0.48	-0.31	–	–
SAOUHSC_01253	FtsK	hypothetical protein	Cell Division and Cell Cycle - no subcategory	-0.34	-0.30	-0.41	-0.30	4.05	-0.06	n.d.	–	▲
SAOUHSC_01460		hypothetical protein	Cell Division and Cell Cycle - no subcategory	-0.02	-0.15	-0.40	-0.16	0.12	-0.09	0.87	–	–
SAOUHSC_01462	GpsB	hypothetical protein	Cell Division and Cell Cycle - no subcategory	0.44	0.32	0.04	0.18	-0.83	-0.48	-0.49	–	–
SAOUHSC_01827	EzrA	septation ring formation regulator EzrA	Cell Division and Cell Cycle - no subcategory	-0.32	-0.63	-0.20	-0.08	0.16	-0.41	0.03	–	–
SAOUHSC_03049		hypothetical protein	Cell Division and Cell Cycle - no subcategory;Cell Division and Cell Cycle - no subcategory	0.07	0.25	-0.22	-0.16	-0.45	-0.30	-0.09	–	–
SAOUHSC_01205	FtsY	signal recognition particle-docking protein FtsY	Cell Division and Cell Cycle - no subcategory;Protein biosynthesis	0.21	0.14	-0.02	0.23	n.d.	-0.39	0.04	–	–
SAOUHSC_01424	MurG	undecaprenyldiphospho-muramoylpentapeptide beta-N- acetylglucosaminyltransferase	Cell Wall and Capsule - no subcategory	0.03	-0.63	-0.15	-0.10	0.67	0.17	-0.24	–	–
SAOUHSC_00752	MurB	UDP-N-acetylenolpyruvoylglucosamine reductase	Cell Wall and Capsule - no subcategory;Cell Wall and Capsule - no subcategory	0.37	0.09	0.10	-0.02	-0.67	-0.16	-0.14	–	–
SAOUHSC_02318	Ddl	D-alanyl-alanine synthetase A	Cell Wall and Capsule - no subcategory;Cell Wall and Capsule - no subcategory	0.15	0.60	0.57	0.45	-1.45	-0.54	0.67	–	–

locus tag	protein symbol	description	theSEED functional category	S9 cell line infection model				murine pneumonia model			median over 8,24 and 32h ratio	
				exp.	8h	24h	32h	8h	24h	32h	median tendence (S9) - 1.5fc cutoff	median tendence (mice) - 1.5fc cutoff
**SAOUHSC_02337**	MurA1	UDP-N-acetylglucosamine 1-carboxyvinyltransferase	Cell Wall and Capsule - no subcategory;Cell Wall and Capsule - no subcategory	-0.17	-0.39	-0.56	-0.56	-0.54	-0.41	-0.44	–	–
**SAOUHSC_02365**	MurA2	UDP-N-acetylglucosamine 1-carboxyvinyltransferase	Cell Wall and Capsule - no subcategory;Cell Wall and Capsule - no subcategory	-0.05	1.27	0.29	0.26	n.d.	0.69	1.96	–	▲
**SAOUHSC_02423**		UDP-N-acetylglucosamine pyrophosphorylase	Cell Wall and Capsule - no subcategory;Cell Wall and Capsule - no subcategory	0.45	0.57	0.17	0.36	0.25	-0.27	-0.04	–	–
**SAOUHSC_02317**	MurF	UDP-N-acetylmuramoylalanyl-D-glutamyl-2,6-diaminopimelate--D-alanyl-D-alanyl ligase	Cell Wall and Capsule - no subcategory;Cell Wall and Capsule - no subcategory;Resistance to antibiotics and toxic compounds	-0.04	0.22	-0.18	-0.24	-1.78	0.37	-0.87	–	▼
**SAOUHSC_01373**	FemA	methicillin resistance factor, FemA	Cell Wall and Capsule - no subcategory;Resistance to antibiotics and toxic compounds	-0.28	-0.56	-0.66	-0.62	-0.14	0.02	0.36	▼	–
**SAOUHSC_01374**	FemB	methicillin resistance factor	Cell Wall and Capsule - no subcategory;Resistance to antibiotics and toxic compounds	-0.27	-0.29	-0.25	-0.46	0.56	0.21	-0.11	–	–
**SAOUHSC_02405**	GlmM	phosphoglucosamine mutase	Cell Wall and Capsule - no subcategory;Resistance to antibiotics and toxic compounds;Capsular and extracellular polysacchrides	0.43	0.02	-0.11	-0.01	-0.58	-0.56	-0.44	–	–
**SAOUHSC_00796**	Pgk	phosphoglycerate kinase	Central carbohydrate metabolism	0.21	-0.09	-0.49	-0.40	0.01	-0.17	-0.33	–	–
**SAOUHSC_00798**	Pgm	phosphoglyceromutase	Central carbohydrate metabolism	-0.36	-0.30	-0.91	-0.74	-0.30	-0.28	-0.35	▼	–
**SAOUHSC_01064**	PycA	pyruvate carboxylase	Central carbohydrate metabolism	-0.21	0.42	0.07	0.06	0.28	0.37	0.61	–	–
**SAOUHSC_01337**	Tkt	transketolase	Central carbohydrate metabolism	0.37	0.83	0.14	0.31	-0.30	-0.37	-0.27	–	–
**SAOUHSC_01646**	Glk	glucokinase	Central carbohydrate metabolism	0.25	0.35	-0.01	-0.00	1.44	-0.73	-0.78	–	▼
**SAOUHSC_01801**	CitC	isocitrate dehydrogenase	Central carbohydrate metabolism	0.25	0.77	2.02	2.45	0.86	0.18	1.03	▲	▲
**SAOUHSC_01810**		NADP-dependent malic enzyme	Central carbohydrate metabolism	0.13	-0.29	-0.26	-0.12	0.63	-0.29	-0.45	–	–
**SAOUHSC_01818**	Ald2	alanine dehydrogenase	Central carbohydrate metabolism	0.09	0.36	1.09	1.44	-0.54	0.98	n.d.	▲	–
**SAOUHSC_01867**	Dat	D-alanine aminotransferase	Central carbohydrate metabolism	0.30	0.90	0.41	0.39	0.40	0.40	-0.20	–	–
**SAOUHSC_01983**	FumC	fumarate hydratase	Central carbohydrate metabolism	0.24	0.77	1.35	1.45	1.12	1.25	1.30	▲	▲
**SAOUHSC_02647**	Mqo1	malate:quinone oxidoreductase	Central carbohydrate metabolism	-0.18	0.16	1.01	1.05	1.49	1.14	-0.30	▲	▲
**SAOUHSC_02703**	GpmA	phosphoglyceromutase	Central carbohydrate metabolism	0.41	0.32	-0.26	0.04	0.37	0.37	0.23	–	–
**SAOUHSC_02927**	Lqo	malate:quinone oxidoreductase	Central carbohydrate metabolism	-0.20	0.26	0.20	0.25	-0.02	-0.01	-0.57	–	–
**SAOUHSC_01611**	BmfBB	2-oxoisovalerate dehydrogenase, E2 component, dihydrolipoamide acetyltransferase	Central carbohydrate metabolism;Branched-chain amino acids;Branched-chain amino acids;Lipoic acid	-0.19	0.04	-0.25	-0.09	-0.13	0.39	n.d.	–	–
**SAOUHSC_00536**	IlvE	branched-chain amino acid aminotransferase	Central carbohydrate metabolism;Branched-chain amino acids;Central carbohydrate metabolism;Branched-chain amino acids;Branched-chain amino acids;Alanine, serine, and glycine;Branched-chain amino acids	0.75	1.27	0.82	0.98	0.15	-0.08	0.49	▲	–
**SAOUHSC_00797**	TpiA	triosephosphate isomerase	Central carbohydrate metabolism;Central carbohydrate metabolism	0.86	0.05	-0.34	-0.32	-0.22	-0.66	-0.78	–	▼
**SAOUHSC_01040**	PdhA	pyruvate dehydrogenase complex, E1 component subunit alpha	Central carbohydrate metabolism;Central carbohydrate metabolism	-0.55	-0.07	-0.57	-0.52	-0.43	-1.15	-1.68	–	▼
**SAOUHSC_01041**	PdhB	pyruvate dehydrogenase complex, E1 component, pyruvate dehydrogenase beta subunit	Central carbohydrate metabolism;Central carbohydrate metabolism	-0.54	-0.20	-0.44	-0.49	-0.04	0.78	-0.26	–	–
**SAOUHSC_01418**	SucA	2-oxoglutarate dehydrogenase E1 component	Central carbohydrate metabolism;Central carbohydrate metabolism	-0.35	0.45	1.24	1.54	n.d.	2.92	1.78	▲	▲
**SAOUHSC_01043**	PdhD	dihydrolipoamide dehydrogenase	Central carbohydrate metabolism;Central carbohydrate metabolism;Central carbohydrate metabolism	-0.60	-0.12	-0.75	-0.55	-0.19	-0.20	-0.26	–	–
**SAOUHSC_01218**	SucD	succinyl-CoA synthetase subunit alpha	Central carbohydrate metabolism;Central carbohydrate metabolism;One-carbon Metabolism	0.16	0.20	1.01	1.27	-1.12	0.04	-0.15	▲	–
**SAOUHSC_00574**	EutD	phosphotransacetylase	Central carbohydrate metabolism;Fermentation;Fermentation	0.33	0.67	0.11	0.21	-0.44	-0.57	-0.60	–	–
**SAOUHSC_01416**	SucB	dihydrolipoamide succinyltransferase	Central carbohydrate metabolism;Lipoic acid;Central carbohydrate metabolism	-0.44	0.64	1.61	1.86	1.43	2.32	1.52	▲	▲
**SAOUHSC_00553**	HxlA	hypothetical protein	Central carbohydrate metabolism;One-carbon Metabolism	1.08	0.38	-0.07	0.12	-0.66	-0.59	-1.22	–	▼
**SAOUHSC_02143**		hypothetical protein	Central carbohydrate metabolism;One-carbon Metabolism	0.53	0.79	0.54	0.66	0.41	0.42	0.26	▲	–
**SAOUHSC_01216**	SucC	succinyl-CoA synthetase subunit beta	Central carbohydrate metabolism;One-carbon Metabolism;Central carbohydrate metabolism	-0.04	0.13	1.01	1.11	-0.39	-0.01	-0.60	▲	–
**SAOUHSC_00866**		D-isomer specific 2-hydroxyacid dehydrogenase NAD binding domain-containing protein	Central carbohydrate metabolism;Organic acids	0.19	0.36	0.30	0.31	0.79	2.93	2.55	–	▲
**SAOUHSC_02577**		D-isomer specific 2-hydroxyacid dehydrogenase NAD binding domain-containing protein	Central carbohydrate metabolism;Organic acids	0.32	1.12	2.01	2.18	0.51	1.12	0.49	▲	–
**SAOUHSC_01806**	PykA	pyruvate kinase	Central carbohydrate metabolism;Organic acids;Central carbohydrate metabolism;Fermentation	0.06	0.36	-0.15	-0.18	-0.64	0.06	-0.38	–	–
**SAOUHSC_00795**	GapA	glyceraldehyde-3-phosphate dehydrogenase, type I	Central carbohydrate metabolism;Oxidative stress;Oxidative stress;Pyridoxine	0.38	-0.07	-0.59	-0.43	4.40	4.32	4.02	–	▲
**SAOUHSC_02142**	AldH	aldehyde dehydrogenase	Central carbohydrate metabolism;Phospholipids;Central carbohydrate metabolism	0.12	0.67	1.20	1.43	-1.40	-0.40	-0.13	▲	–
**SAOUHSC_02849**	CidC	pyruvate oxidase	Central carbohydrate metabolism;Programmed Cell Death and Toxin-antitoxin Systems	0.19	-1.07	-0.48	-0.32	-0.68	-0.04	-0.51	–	–
**SAOUHSC_01178**	CoaBC	phosphopantothenoylcysteine decarboxylase/phosphopantothenate--cysteine ligase	Coenzyme A	-0.32	-0.15	-0.19	-0.28	n.d.	-0.03	0.78	–	–
**SAOUHSC_02918**	PanC	pantoate--beta-alanine ligase	Coenzyme A	-0.34	-0.42	-1.28	-1.41	n.d.	1.63	0.77	▼	▲
**SAOUHSC_02919**	PanB	3-methyl-2-oxobutanoate hydroxymethyltransferase	Coenzyme A	-0.04	0.27	-0.71	-0.64	-0.86	-0.30	-1.02	▼	▼
**SAOUHSC_00819**	CspC	hypothetical protein	Cold shock	-0.63	0.05	-1.20	-1.27	0.57	-0.23	-1.13	▼	–
**SAOUHSC_01403**	CspA	cold shock protein	Cold shock	0.35	2.14	1.95	2.15	3.79	3.02	2.95	▲	▲
**SAOUHSC_02791**		pyrophosphohydrolase	Detoxification	0.57	0.68	0.29	0.50	-0.55	-0.76	n.d.	–	▼
**SAOUHSC_01741**	Dtd	D-tyrosyl-tRNA(Tyr) deacylase	Detoxification;Regulation and Cell signaling - no subcategory;Organic sulfur assimilation	0.84	0.21	0.20	0.17	-0.73	0.03	n.d.	–	–
**SAOUHSC_00155**	GlcA	PTS system glucose-specific protein	Di- and oligosaccharides	-0.64	-0.31	-0.17	-0.15	0.13	-0.34	-1.14	–	–

locus tag	protein symbol	description	theSEED functional category	S9 cell line infection model				murine pneumonia model			median over 8,24 and 32h ratio	
				exp.	8h	24h	32h	8h	24h	32h	median tendence (S9) - 1.5fc cutoff	median tendence (mice) - 1.5fc cutoff
**SAOUHSC_01430**	Crr	phosphotransferase system enzyme IIA	Di- and oligosaccharides	0.59	2.32	1.47	1.59	0.17	-0.52	-0.58	▲	–
**SAOUHSC_02268**	ScrB	sucrose-6-phosphate hydrolase	Di- and oligosaccharides	0.23	0.35	0.05	0.16	0.53	-0.15	n.d.	–	–
**SAOUHSC_02848**	GlcB	PTS system glucose-specific transporter subunit IIABC	Di- and oligosaccharides	-0.08	0.50	0.79	0.82	-0.75	0.60	0.47	▲	–
**SAOUHSC_01490**	Hup	DNA-binding protein HU	DNA Metabolism - no subcategory	0.21	-0.05	-0.01	-0.04	-0.80	0.21	0.74	–	–
**SAOUHSC_01472**	DinG	DnaQ family exonuclease/DinG family helicase	DNA repair	-0.06	0.23	-0.02	0.05	3.60	0.30	1.88	–	▲
**SAOUHSC_01620**	XseA	exodeoxyribonuclease VII large subunit	DNA repair	-0.23	-0.26	-0.34	0.09	n.d.	0.12	n.d.	–	–
**SAOUHSC_01975**		hypothetical protein	DNA repair	-0.48	-0.41	-0.58	-0.58	0.54	0.41	0.06	▼	–
**SAOUHSC_02123**	PcrA	ATP-dependent DNA helicase PcrA	DNA repair	-0.53	-0.46	-0.74	-0.88	n.d.	-0.22	1.73	▼	▲
**SAOUHSC_01262**	RecA	recombinase A	DNA repair;DNA repair;Plant-Prokaryote comparative genomics;DNA replication;DNA repair	-0.34	-0.61	-0.61	-0.54	0.16	-0.03	-0.34	▼	–
**SAOUHSC_01615**	RecN	DNA repair protein RecN	DNA repair;DNA replication	-0.26	0.40	0.23	0.17	-0.34	1.46	0.14	–	–
**SAOUHSC_00001**	DnaA	chromosomal replication initiation protein	DNA replication	-0.46	-0.24	-0.03	-0.19	n.d.	0.82	-0.16	–	–
**SAOUHSC_00002**	DnaN	DNA polymerase III subunit beta	DNA replication	-0.04	0.50	0.48	0.52	0.63	-0.09	-0.09	–	–
**SAOUHSC_01241**	PolC	DNA polymerase III PolC	DNA replication	-0.45	-0.01	-0.68	-0.84	0.28	-0.07	n.d.	▼	–
**SAOUHSC_01791**	DnaI	primosomal protein DnaI	DNA replication	0.09	0.03	0.40	0.37	n.d.	n.d.	0.53	–	–
**SAOUHSC_01797**	PolA	DNA polymerase I	DNA replication;DNA repair	-0.15	0.29	-0.11	0.06	-1.00	-0.18	-0.63	–	▼
**SAOUHSC_00442**	DnaX	DNA polymerase III subunits gamma and tau	DNA replication;DNA uptake, competence	-0.26	-0.03	-0.48	-0.53	-0.93	-0.11	1.20	–	–
**SAOUHSC_00005**	GyrB	DNA gyrase, B subunit	DNA replication;Resistance to antibiotics and toxic compounds	-0.37	-0.07	-0.41	-0.33	2.98	-0.73	0.37	–	–
**SAOUHSC_00006**	GyrA	DNA gyrase, A subunit	DNA replication;Resistance to antibiotics and toxic compounds	-0.48	-0.11	-0.38	-0.37	0.50	-0.06	0.26	–	–
**SAOUHSC_01351**	ParE	DNA topoisomerase IV subunit B	DNA replication;Resistance to antibiotics and toxic compounds	-0.36	-0.74	-0.96	-0.82	0.85	0.02	0.78	▼	▲
**SAOUHSC_01352**	ParC	DNA topoisomerase IV subunit A	DNA replication;Resistance to antibiotics and toxic compounds	-0.32	-0.33	-0.77	-0.65	1.44	-0.01	n.d.	▼	▲
**SAOUHSC_02517**	TopB	DNA topoisomerase III	DNA uptake, competence	-0.14	-1.04	-1.28	-1.77	n.d.	-0.61	6.00	▼	▲
**SAOUHSC_00444**		hypothetical protein	DNA uptake, competence;DNA repair	-0.24	0.35	-0.29	-0.37	1.76	1.94	2.17	–	▲
**SAOUHSC_00469**	SpoVG	regulatory protein SpoVG	Dormancy and Sporulation - no subcategory	0.11	-0.80	-0.64	-0.09	-0.33	-0.80	-0.61	▼	▼
**SAOUHSC_00483**		hypothetical protein	Dormancy and Sporulation - no subcategory	-0.20	-0.47	-0.60	-0.84	0.16	-0.40	-0.42	▼	–
**SAOUHSC_00480**		hypothetical protein	Dormancy and Sporulation - no subcategory;Detoxification	-0.03	0.19	-0.51	-0.51	-1.26	-0.77	5.62	–	▼
**SAOUHSC_00477**	Mfd	transcription-repair coupling factor	Dormancy and Sporulation - no subcategory;DNA replication;Transcription	-0.04	-0.21	0.05	-0.13	n.d.	0.70	n.d.	–	▲
**SAOUHSC_00481**		hypothetical protein	Dormancy and Sporulation - no subcategory;Heat shock	0.91	0.52	0.06	-0.07	-0.48	-0.82	-0.92	–	▼
**SAOUHSC_01002**	QoxA	quinol oxidase AA3 subunit II	Electron accepting reactions	-0.48	-0.46	-0.26	-0.23	-0.41	-1.03	0.06	–	–
**SAOUHSC_00875**		hypothetical protein	Electron donating reactions	0.27	-0.44	-0.11	0.16	-0.83	-0.07	-0.10	–	–
**SAOUHSC_00878**	Ndh2	hypothetical protein	Electron donating reactions	-0.18	-0.28	-0.28	-0.29	0.77	0.55	0.30	–	–
**SAOUHSC_01104**	SdhA	succinate dehydrogenase flavoprotein subunit	Electron donating reactions;Central carbohydrate metabolism;One-carbon Metabolism	0.24	0.10	1.13	1.24	0.44	0.69	0.26	▲	–
**SAOUHSC_00920**	FabH	3-oxoacyl-(acyl carrier protein) synthase III	Fatty acids	-1.39	-0.86	-0.96	-0.77	n.d.	-0.89	-1.49	▼	▼
**SAOUHSC_00921**	FabF	3-oxoacyl- synthase	Fatty acids	-0.92	-0.52	-0.61	-0.37	0.04	-0.16	-0.52	–	–
**SAOUHSC_00947**	FabI	enoyl-(acyl carrier protein) reductase	Fatty acids	-0.13	-0.01	-0.32	-0.29	-0.49	0.03	-0.47	–	–
**SAOUHSC_01198**	FabD	malonyl CoA-acyl carrier protein transacylase	Fatty acids	-0.59	-0.26	-0.57	-0.26	-0.40	-0.18	-0.73	–	–
**SAOUHSC_01199**	FabG	3-oxoacyl-(acyl-carrier-protein) reductase	Fatty acids	-0.24	-0.15	-0.29	-0.08	0.51	-0.36	-0.95	–	–
**SAOUHSC_01808**	AccA	acetyl-CoA carboxylase carboxyltransferase subunit alpha	Fatty acids	0.01	-0.19	-0.13	-0.27	n.d.	0.02	n.d.	–	–
**SAOUHSC_01623**	AccC	acetyl-CoA carboxylase biotin carboxylase subunit	Fatty acids;Miscellaneous - no subcategory	-0.07	0.09	-0.51	-0.36	0.17	-0.54	1.00	–	–
**SAOUHSC_00086**	ButA	acetoin reductase	Fermentation	0.82	2.29	3.34	3.68	3.11	2.73	3.09	▲	▲
**SAOUHSC_00206**	Ldh1	L-lactate dehydrogenase	Fermentation;Fermentation	-1.15	-0.25	-2.09	-2.87	0.44	0.64	0.61	▼	▲
**SAOUHSC_02830**	Ddh	D-lactate dehydrogenase	Fermentation;Fermentation	0.59	0.31	-0.19	-0.19	0.59	-0.16	-0.53	–	–
**SAOUHSC_02922**	Ldh2	L-lactate dehydrogenase	Fermentation;Fermentation	0.51	0.89	0.25	0.35	-0.13	-0.04	-0.02	–	–
**SAOUHSC_01820**	AckA	acetate kinase	Fermentation;Fermentation;Central carbohydrate metabolism	-0.45	-0.60	-0.73	-0.64	-0.44	-0.05	-0.58	▼	–
**SAOUHSC_00608**	Adh	alcohol dehydrogenase	Fermentation;Fermentation;Phospholipids	-1.11	0.89	-0.40	-0.54	1.10	0.69	n.d.	–	▲
**SAOUHSC_00187**	PflB	formate acetyltransferase	Fermentation;Fermentation;Respiration - no subcategory	-2.19	2.07	1.19	0.75	1.12	1.65	1.12	▲	▲
**SAOUHSC_02544**	MoaB	molybdopterin precursor biosynthesis moaB	Folate and pterines	0.31	0.37	0.51	0.69	0.81	0.43	0.37	–	–
**SAOUHSC_00549**	FolE2	putative GTP cyclohydrolase	Folate and pterines;RNA processing and modification	0.87	-0.27	-0.40	-0.01	0.70	0.39	0.67	–	▲
**SAOUHSC_01497**	AnsA	L-asparaginase	Glutamine, glutamate, aspartate, asparagine; ammonia assimilation	0.03	0.08	-0.49	-0.35	1.24	0.23	n.d.	–	▲

locus tag	protein symbol	description	theSEED functional category	S9 cell line infection model				murine pneumonia model			median over 8,24 and 32h ratio	
				exp.	8h	24h	32h	8h	24h	32h	median tendence (S9) - 1.5fc cutoff	median tendence (mice) - 1.5fc cutoff
**SAOUHSC_01106**	MurI	glutamate racemase	Glutamine, glutamate, aspartate, asparagine; ammonia assimilation;Cell Wall and Capsule - no subcategory	-0.18	0.10	-0.26	-0.16	n.d.	0.91	-0.78	–	–
**SAOUHSC_00895**	GudB	glutamate dehydrogenase, NAD-specific	Glutamine, glutamate, aspartate, asparagine; ammonia assimilation;Glutamine, glutamate, aspartate, asparagine; ammonia assimilation	-0.36	1.13	1.76	2.09	3.19	0.94	2.06	▲	▲
**SAOUHSC_00223**	TarF	teichoic acid biosynthesis protein F	Gram-Positive cell wall components	0.03	-0.12	0.12	-0.06	-0.31	2.19	-1.53	–	–
**SAOUHSC_00641**	TagH	teichoic acids export protein ATP-binding subunit	Gram-Positive cell wall components	-0.57	-0.63	-0.75	-0.74	0.17	n.d.	-1.63	▼	▼
**SAOUHSC_00974**		glycosyl transferase, group 1	Gram-Positive cell wall components	-0.13	-1.15	-0.18	-0.16	n.d.	1.57	n.d.	–	▲
**SAOUHSC_00872**	DltD	extramembranal protein	Gram-Positive cell wall components;Gram-Positive cell wall components;Resistance to antibiotics and toxic compounds	-0.60	-1.20	-0.44	-0.40	-0.25	-0.49	n.d.	–	–
**SAOUHSC_00225**	TarI	2-C-methyl-D-erythritol 4-phosphate cytidylyltransferase	Gram-Positive cell wall components;Plant-Prokaryote comparative genomics;Isoprenoids	0.76	0.37	0.44	0.62	-0.53	-0.28	-0.43	–	–
**SAOUHSC_01683**	DnaK	molecular chaperone DnaK	Heat shock;Protein folding	0.20	-0.37	-0.01	0.30	0.01	0.22	0.10	–	–
**SAOUHSC_02610**	HutG	formimidoylglutamase	Histidine Metabolism	0.49	-0.05	n.d.	0.56	1.09	-0.69	n.d.	–	–
**SAOUHSC_01738**	HisS	histidyl-tRNA synthetase	Histidine Metabolism;Organic sulfur assimilation;Regulation and Cell signaling - no subcategory	-0.16	-0.50	-1.03	-0.89	-1.20	-0.87	-1.08	▼	▼
**SAOUHSC_00749**	SstD	hypothetical protein	Iron acquisition and metabolism - no subcategory;Iron acquisition and metabolism - no subcategory	-2.15	-0.27	-0.81	-1.18	-0.21	n.d.	n.d.	▼	–
**SAOUHSC_00579**	MvaK2	phosphomevalonate kinase	Isoprenoids;Isoprenoids	-0.17	0.08	0.07	0.00	-1.30	0.37	1.18	–	–
**SAOUHSC_02859**	MvaA	hydroxymethylglutaryl-CoA reductase, degradative	Isoprenoids;Isoprenoids	-0.17	-0.26	-0.36	-0.11	n.d.	2.61	0.21	–	▲
**SAOUHSC_02860**	MvaS	HMG-CoA synthase	Isoprenoids;Isoprenoids	0.35	1.06	0.40	0.45	-0.02	0.16	0.01	–	–
**SAOUHSC_01042**	PdhC	branched-chain alpha-keto acid dehydrogenase subunit E2	Lipoic acid;Central carbohydrate metabolism;Central carbohydrate metabolism	-0.41	-0.06	-0.40	-0.31	0.78	0.08	0.32	–	–
**SAOUHSC_00338**	MetE	5-methyltetrahydropteroyltriglutamate--homocysteine S-methyltransferase	Lysine, threonine, methionine, and cysteine	0.47	0.69	0.79	1.21	n.d.	2.73	3.10	▲	▲
**SAOUHSC_01321**	ThrC	threonine synthase	Lysine, threonine, methionine, and cysteine	0.98	0.91	1.19	1.11	1.44	1.57	2.25	▲	▲
**SAOUHSC_01398**	DapD	2,3,4,5-tetrahydropyridine-2-carboxylate N-succinyltransferase	Lysine, threonine, methionine, and cysteine	0.80	3.97	3.69	3.89	n.d.	2.09	n.d.	▲	▲
**SAOUHSC_01401**	LysA	diaminopimelate decarboxylase	Lysine, threonine, methionine, and cysteine	0.83	1.45	1.23	1.25	-0.62	0.26	0.09	▲	–
**SAOUHSC_00488**	CysK	hypothetical protein	Lysine, threonine, methionine, and cysteine;Lysine, threonine, methionine, and cysteine	0.38	0.55	0.66	0.76	-0.73	-0.49	0.14	▲	–
**SAOUHSC_01320**	Hom	homoserine dehydrogenase	Lysine, threonine, methionine, and cysteine;Lysine, threonine, methionine, and cysteine	0.75	0.64	1.11	1.20	1.64	1.64	1.81	▲	▲
**SAOUHSC_01395**	Asd	aspartate semialdehyde dehydrogenase	Lysine, threonine, methionine, and cysteine;Lysine, threonine, methionine, and cysteine	0.51	3.31	3.45	3.51	1.63	1.44	2.06	▲	▲
**SAOUHSC_01909**	MetK	S-adenosylmethionine synthetase	Lysine, threonine, methionine, and cysteine;Lysine, threonine, methionine, and cysteine	0.24	-0.29	-0.04	-0.07	0.32	-0.22	0.08	–	–
**SAOUHSC_02579**		hypothetical protein	Metabolism of Aromatic Compounds - no subcategory;Metabolism of central aromatic intermediates;Peripheral pathways for catabolism of aromatic compounds	-0.33	-0.28	-0.14	-0.38	0.23	-0.16	n.d.	–	–
**SAOUHSC_00906**		hypothetical protein	Metabolism of central aromatic intermediates;Metabolism of Aromatic Compounds - no subcategory	0.64	0.65	0.41	0.55	0.49	0.30	0.52	–	–
**SAOUHSC_01845**	Fhs	formate--tetrahydrofolate ligase	Miscellaneous - no subcategory;One-carbon Metabolism;One-carbon Metabolism;Folate and pterines	-0.18	0.48	0.30	0.52	-0.33	0.11	-0.27	–	–
**SAOUHSC_01018**	PurD	phosphoribosylamine--glycine ligase	Miscellaneous - no subcategory;Purines	-0.29	0.03	-1.40	-1.11	-0.21	-0.29	-0.00	▼	–
**SAOUHSC_02368**	PyrG	CTP synthetase	Miscellaneous - no subcategory;Pyrimidines	-0.07	-0.55	-0.46	-0.40	-1.00	-0.45	-0.16	–	–
**SAOUHSC_00101**	DeoB	phosphopentomutase	Monosaccharides	0.45	0.35	0.30	0.77	0.14	0.05	-0.14	–	–
**SAOUHSC_01907**		hypothetical protein	Monosaccharides	0.11	1.41	1.95	2.08	n.d.	0.36	3.60	▲	▲
**SAOUHSC_02793**		hypothetical protein	Monosaccharides;Cell Wall and Capsule - no subcategory;Capsular and extracellular polysacchrides	0.10	0.91	0.21	0.42	n.d.	-0.18	n.d.	–	–
**SAOUHSC_01807**	PfkA	6-phosphofructokinase	Monosaccharides;Central carbohydrate metabolism	0.10	0.59	-0.04	-0.15	0.48	0.07	-0.45	–	–
**SAOUHSC_01901**	Tal	putative translaldolase	Monosaccharides;Central carbohydrate metabolism	0.63	0.74	0.25	0.15	-0.21	-0.43	-0.64	–	–
**SAOUHSC_01028**	PtsH	phosphocarrier protein HPr	Monosaccharides;Regulation and Cell signaling - no subcategory;Sugar alcohols	0.49	0.64	-0.02	-0.01	1.07	0.80	0.78	–	▲
**SAOUHSC_01029**	PtsI	phosphoenolpyruvate-protein phosphotransferase	Monosaccharides;Sugar alcohols	0.03	0.39	-0.25	-0.13	-0.80	-0.58	-0.77	–	▼
**SAOUHSC_02132**	NadE	NAD synthetase	NAD and NADP	0.53	0.70	0.07	0.41	0.33	-0.22	1.08	–	–
**SAOUHSC_02122**	LigA	DNA ligase, NAD-dependent	NAD and NADP;DNA repair	-0.11	0.05	0.01	-0.28	0.28	-0.28	0.04	–	–
**SAOUHSC_01287**	GlnA	glutamine synthetase, type I	Nitrogen Metabolism - no subcategory;Glutamine, glutamate, aspartate, asparagine; ammonia assimilation;Cell Wall and Capsule - no subcategory;Glutamine, glutamate, aspartate, asparagine; ammonia assimilation	0.64	-0.02	-0.09	0.17	0.64	0.17	-0.01	–	–
**SAOUHSC_00743**	NrdF	ribonucleotide-diphosphate reductase subunit beta	Nucleosides and Nucleotides - no subcategory	0.80	-0.53	-1.29	-1.32	-1.20	-1.36	-1.53	▼	▼
**SAOUHSC_01587**	RluB	ribosomal large subunit pseudouridine synthase B	Nucleosides and Nucleotides - no subcategory;Cell Division and Cell Cycle - no subcategory	-0.22	-0.38	-0.99	-0.74	-0.47	-0.58	0.09	▼	–
**SAOUHSC_00742**	NrdE	ribonucleotide-diphosphate reductase subunit alpha	Nucleosides and Nucleotides - no subcategory;Oxidative stress	0.59	-0.97	-1.88	-1.94	-2.50	-2.12	-1.43	▼	▼
**SAOUHSC_02828**		hypothetical protein	One-carbon Metabolism	0.37	0.13	1.81	1.63	n.d.	1.36	1.40	▲	▲
**SAOUHSC_00900**	Pgi	glucose-6-phosphate isomerase	One-carbon Metabolism;Central carbohydrate metabolism	-0.05	0.80	-0.10	0.09	0.25	2.15	2.40	–	▲
**SAOUHSC_02366**	FbaA	fructose-bisphosphate aldolase	One-carbon Metabolism;Central carbohydrate metabolism	0.04	0.52	-0.17	-0.10	-0.11	0.14	-0.15	–	–
**SAOUHSC_02926**	FdaB	fructose-1,6-bisphosphate aldolase	One-carbon Metabolism;Central carbohydrate metabolism	0.18	0.18	-0.08	0.23	-0.64	-0.41	-0.62	–	▼
**SAOUHSC_00799**	Eno	phosphopyruvate hydratase	One-carbon Metabolism;Central carbohydrate metabolism;Central carbohydrate metabolism	0.18	0.13	-0.23	-0.15	0.31	0.07	0.29	–	–
**SAOUHSC_01599**	Zwf	glucose-6-phosphate 1-dehydrogenase	One-carbon Metabolism;Central carbohydrate metabolism;Fermentation;Central carbohydrate metabolism	0.19	0.73	0.15	0.10	-0.49	-0.60	-0.26	–	–

locus tag	protein symbol	description	theSEED functional category	S9 cell line infection model				murine pneumonia model			median over 8,24 and 32h ratio	
				exp.	8h	24h	32h	8h	24h	32h	median tendence (S9) - 1.5fc cutoff	median tendence (mice) - 1.5fc cutoff
**SAOUHSC_01605**	Gnd	6-phosphogluconate dehydrogenase	One-carbon Metabolism;Central carbohydrate metabolism;Monosaccharides	0.12	0.48	-0.15	0.02	-0.34	0.05	0.29	–	–
**SAOUHSC_01007**	FolD	bifunctional 5,10-methylene-tetrahydrofolate dehydrogenase/ 5,10-methylene-tetrahydrofolate cyclohydrolase	One-carbon Metabolism;One-carbon Metabolism;Folate and pterines	0.39	0.73	0.41	0.61	0.20	0.28	-0.23	▲	–
**SAOUHSC_01735**		hypothetical protein	Organic sulfur assimilation	0.19	-0.08	-0.20	-0.02	n.d.	-0.13	0.36	–	–
**SAOUHSC_01737**	AspS	aspartyl-tRNA synthetase	Organic sulfur assimilation	0.03	-0.26	-0.40	-0.48	-0.16	-0.33	-0.27	–	–
**SAOUHSC_02699**	TcyA	hypothetical protein	Organic sulfur assimilation	-0.06	-0.08	0.25	0.31	0.01	-1.15	n.d.	–	–
**SAOUHSC_00849**	SufS	aminotransferase	Organic sulfur assimilation;Alanine, serine, and glycine	0.49	0.07	0.34	0.62	-0.49	-0.05	n.d.	–	–
**SAOUHSC_00851**	SufB	hypothetical protein	Organic sulfur assimilation;Phages, Prophages	0.16	-0.25	-0.25	-0.18	-0.80	-1.74	-1.20	–	▼
**SAOUHSC_01346**	OpuD1	glycine betaine transporter	Osmotic stress	0.02	-0.21	-0.10	-0.38	-0.29	-0.70	-1.22	–	▼
**SAOUHSC_02932**	BetA	choline dehydrogenase	Osmotic stress	0.42	-1.50	-0.77	-0.51	n.d.	n.d.	3.95	▼	▲
**SAOUHSC_00757**	PepT	peptidase T	Oxidative stress	0.04	0.40	0.26	0.47	-0.32	0.14	-0.17	–	–
**SAOUHSC_00831**	OsmC	hypothetical protein	Oxidative stress	1.06	-0.30	0.06	n.d.	4.34	0.72	0.58	–	▲
**SAOUHSC_01282**	BsaA	glutathione peroxidase	Oxidative stress	0.47	0.86	0.51	0.73	n.d.	n.d.	1.64	▲	▲
**SAOUHSC_01592**	Fur	transcriptional regulator, Fur	Oxidative stress	0.56	0.23	0.21	0.05	n.d.	0.83	-0.02	–	–
**SAOUHSC_02273**	Rex	redox-sensing transcriptional repressor Rex	Oxidative stress	0.06	0.06	0.09	-0.04	0.17	-0.05	-0.52	–	–
**SAOUHSC_02381**	Dps	hypothetical protein	Oxidative stress	0.20	0.26	1.70	2.53	-0.87	2.88	0.14	▲	–
**SAOUHSC_02133**		nicotinate phosphoribosyltransferase	Oxidative stress;NAD and NADP	-0.14	0.18	-0.66	-0.57	0.33	0.10	0.18	–	–
**SAOUHSC_01327**	KatA	catalase	Oxidative stress;Oxidative stress	0.82	0.22	0.88	1.42	2.71	3.36	3.90	▲	▲
**SAOUHSC_00093**	SodM	superoxide dismutase	Oxidative stress;Oxidative stress;Nitrogen Metabolism - no subcategory	0.26	0.16	1.10	0.29	0.92	2.76	2.71	–	▲
**SAOUHSC_01653**	SodA	superoxide dismutase, Mn	Oxidative stress;Oxidative stress;Nitrogen Metabolism - no subcategory	0.51	1.57	1.84	1.99	0.34	1.92	1.99	▲	▲
**SAOUHSC_01630**		hypothetical protein	Oxidative stress;Potassium metabolism - no subcategory	-0.04	-0.41	-0.91	-0.67	-1.24	n.d.	-1.60	▼	▼
**SAOUHSC_01249**	RibC	riboflavin biosynthesis protein RibF	Oxidative stress;Riboflavin, FMN, FAD;Riboflavin, FMN, FAD	0.29	-0.07	-0.45	-0.40	-0.11	-0.80	-0.32	–	–
**SAOUHSC_00204**	Hmp	globin domain-containing protein	Oxidative stress;Stress Response - no subcategory;Stress Response - no subcategory	-0.86	1.48	-0.08	0.07	0.52	-0.93	-0.05	–	–
**SAOUHSC_00426**	MetQ2	ABC transporter substrate-binding protein	Pathogenicity islands;Lysine, threonine, methionine, and cysteine;Lysine, threonine, methionine, and cysteine	0.23	0.02	1.33	1.17	n.d.	0.99	1.52	▲	▲
**SAOUHSC_00844**	MetQ1	hypothetical protein	Pathogenicity islands;Lysine, threonine, methionine, and cysteine;Lysine, threonine, methionine, and cysteine	0.54	0.03	0.79	0.90	0.67	0.50	1.10	▲	▲
**SAOUHSC_02254**	GroEL	chaperonin GroEL	Pathogenicity islands;Protein folding	0.09	-0.36	-0.57	-0.50	0.04	0.04	0.23	–	–
**SAOUHSC_02071**		single-strand DNA-binding protein	Phages, Prophages	n.d.	n.d.	n.d.	n.d.	3.18	2.78	4.15	n.d.	▲
**SAOUHSC_02080**		bacteriophage L54a, antirepressor	Phages, Prophages	n.d.	n.d.	n.d.	n.d.	4.10	3.54	3.34	**n.d.**	▲
**SAOUHSC_01193**	FakA	hypothetical protein	Phospholipids	-0.12	-0.33	-0.65	-0.38	-0.15	0.05	-0.10	–	–
**SAOUHSC_01197**	PlsX	putative glycerol-3-phosphate acyltransferase PlsX	Phospholipids	-0.39	-0.16	-0.39	-0.29	-1.25	-0.94	n.d.	–	▼
**SAOUHSC_01310**	Cls1	cardiolipin synthetase	Phospholipids	-0.51	-0.27	-0.28	-0.08	n.d.	-0.26	-0.05	–	–
**SAOUHSC_02363**		aldehyde dehydrogenase	Phospholipids;Central carbohydrate metabolism;Central carbohydrate metabolism	0.85	0.01	0.62	0.81	-0.48	-0.20	0.25	▲	–
**SAOUHSC_01278**	GlpD	aerobic glycerol-3-phosphate dehydrogenase	Phospholipids;Electron donating reactions;Sugar alcohols	-0.13	-0.33	0.38	0.55	1.13	0.94	1.39	–	▲
**SAOUHSC_01201**	AcpP	acyl carrier protein	Phospholipids;Resistance to antibiotics and toxic compounds;Fatty acids	0.77	0.04	0.06	0.23	-0.51	-1.03	-0.99	–	▼
**SAOUHSC_01491**	GpsA	NAD(P)H-dependent glycerol-3-phosphate dehydrogenase	Phospholipids;Sugar alcohols;Oxidative stress	-0.50	-0.31	-0.52	-0.42	8.22	0.34	0.97	–	▲
**SAOUHSC_00060**		hypothetical protein	Phosphorus Metabolism - no subcategory	-0.44	0.06	0.22	0.20	1.80	1.31	n.d.	–	▲
**SAOUHSC_01672**	YbeY	hypothetical protein	Phosphorus Metabolism - no subcategory	0.23	0.76	0.27	0.51	0.41	-0.39	n.d.	–	–
**SAOUHSC_02140**	PpaC	putative manganese-dependent inorganic pyrophosphatase	Phosphorus Metabolism - no subcategory	0.20	0.06	-0.28	-0.17	-0.52	-0.22	-0.02	–	–
**SAOUHSC_00511**	CysS	cysteinyl-tRNA synthetase	Plant-Prokaryote comparative genomics	0.36	0.23	0.12	0.26	0.02	-0.15	-0.10	–	–
**SAOUHSC_00513**		hypothetical protein	Plant-Prokaryote comparative genomics	-0.18	-0.06	-0.39	-0.38	-0.57	-0.57	n.d.	–	–
**SAOUHSC_01261**	CinA	competence-damage inducible protein cinA	Plant-Prokaryote comparative genomics;Riboflavin, FMN, FAD	-0.35	0.08	-0.08	-0.56	1.17	-0.06	n.d.	–	–
**SAOUHSC_01034**	TrkA	hypothetical protein	Potassium metabolism - no subcategory;Potassium metabolism - no subcategory;Potassium metabolism - no subcategory	-0.52	0.16	-0.27	-0.16	n.d.	1.93	1.84	–	▲
**SAOUHSC_01016**	PurN	phosphoribosylglycinamide formyltransferase	Potassium metabolism - no subcategory;Purines	-0.57	0.58	-0.48	-0.56	n.d.	n.d.	0.52	–	–
**SAOUHSC_02158**		hypothetical protein	Proline and 4-hydroxyproline;Lysine, threonine, methionine, and cysteine;Glutamine, glutamate, aspartate, asparagine; ammonia assimilation	0.00	0.26	-0.15	-0.17	-0.99	-0.18	n.d.	–	▼
**SAOUHSC_02869**	RocA	1-pyrroline-5-carboxylate dehydrogenase	Proline and 4-hydroxyproline;Proline and 4-hydroxyproline;Arginine; urea cycle, polyamines	0.32	3.89	5.03	5.22	n.d.	2.26	2.79	▲	▲
**SAOUHSC_01597**	ProC	pyrroline-5-carboxylate reductase	Proline and 4-hydroxyproline;Proline and 4-hydroxyproline;Proline and 4-hydroxyproline;Proline and 4-hydroxyproline	0.84	0.83	1.10	1.02	2.16	-0.08	1.32	▲	▲
**SAOUHSC_00346**	YchF	GTP-dependent nucleic acid-binding protein EngD	Protein biosynthesis	0.43	0.26	0.16	0.07	-0.29	-0.32	-0.48	–	–
**SAOUHSC_00348**	RpsF	30S ribosomal protein S6	Protein biosynthesis	-0.38	-0.79	-0.99	-0.76	0.01	0.76	2.01	▼	▲

locus tag	protein symbol	description	theSEED functional category	S9 cell line infection model				murine pneumonia model			median over 8,24 and 32h ratio	
				exp.	8h	24h	32h	8h	24h	32h	median tendence (S9) - 1.5fc cutoff	median tendence (mice) - 1.5fc cutoff
**SAOUHSC_00474**	RplY	50S ribosomal protein L25/general stress protein Ctc	Protein biosynthesis	0.62	-0.19	-0.47	-0.24	-0.67	-0.52	-0.95	–	▼
**SAOUHSC_00518**	RplK	50S ribosomal protein L11	Protein biosynthesis	-0.01	-0.82	-1.28	-1.31	-0.24	-1.24	-1.51	▼	▼
**SAOUHSC_00519**	RplA	50S ribosomal protein L1	Protein biosynthesis	-0.21	-1.04	-1.14	-0.97	0.04	0.21	0.23	▼	–
**SAOUHSC_00520**	RplJ	50S ribosomal protein L10	Protein biosynthesis	-0.22	-0.53	-0.77	-0.59	-0.38	-0.31	-0.73	▼	–
**SAOUHSC_00521**	RplL	50S ribosomal protein L7/L12	Protein biosynthesis	0.07	-0.45	-0.61	-0.57	2.56	1.49	0.87	–	▲
**SAOUHSC_00611**	ArgS	arginyl-tRNA synthetase	Protein biosynthesis	0.35	0.16	-0.08	-0.02	-0.31	0.06	-0.05	–	–
**SAOUHSC_00767**	SaHPF	Staphylococcus aureus hibernation promoting factor	Protein biosynthesis	0.89	1.40	2.42	2.80	1.78	2.69	1.99	▲	▲
**SAOUHSC_00933**	TrpS	tryptophanyl-tRNA synthetase	Protein biosynthesis	0.23	0.24	0.08	0.26	-0.27	0.56	0.81	–	–
**SAOUHSC_00956**	PrfC	peptide chain release factor 3	Protein biosynthesis	-0.33	-0.31	-0.03	-0.24	0.27	1.36	2.17	–	▲
**SAOUHSC_01058**	TypA	GTP-binding protein TypA	Protein biosynthesis	-0.70	-0.99	-0.68	-0.52	0.08	-0.17	0.06	▼	–
**SAOUHSC_01092**	PheS	phenylalanyl-tRNA synthetase subunit alpha	Protein biosynthesis	-0.03	-1.00	-0.87	-0.81	-0.79	-0.25	-1.09	▼	▼
**SAOUHSC_01093**	PheT	phenylalanyl-tRNA synthetase subunit beta	Protein biosynthesis	-0.36	-0.90	-0.96	-0.79	-0.05	0.35	-0.32	▼	–
**SAOUHSC_01163**		hypothetical protein	Protein biosynthesis	-0.20	-0.66	-0.19	-0.18	-0.30	n.d.	-0.47	–	–
**SAOUHSC_01208**	RpsP	30S ribosomal protein S16	Protein biosynthesis	0.40	0.03	-0.38	-0.39	-0.47	-0.75	-1.06	–	▼
**SAOUHSC_01211**	RplS	50S ribosomal protein L19	Protein biosynthesis	-0.18	-0.71	-0.78	-0.74	0.99	1.41	0.17	▼	▲
**SAOUHSC_01214**	RbgA	ribosomal biogenesis GTPase	Protein biosynthesis	-0.11	-0.26	-0.02	0.09	n.d.	-0.23	-0.17	–	–
**SAOUHSC_01232**	RpsB	30S ribosomal protein S2	Protein biosynthesis	-0.10	-0.64	-0.84	-0.77	0.51	-0.06	-0.60	▼	–
**SAOUHSC_01234**	Tsf	elongation factor Ts	Protein biosynthesis	0.63	-0.06	-0.20	0.02	0.09	0.03	-0.18	–	–
**SAOUHSC_01236**	Frr	ribosome recycling factor	Protein biosynthesis	0.55	-0.16	-0.25	-0.14	-0.42	-0.55	-0.70	–	–
**SAOUHSC_01471**	AsnC	asparaginyl-tRNA synthetase	Protein biosynthesis	-0.01	0.25	-0.18	-0.04	-0.21	0.10	0.14	–	–
**SAOUHSC_01492**	EngA	GTP-binding protein EngA	Protein biosynthesis	-0.37	-0.40	-0.35	-0.27	-0.25	-0.92	-0.55	–	–
**SAOUHSC_01493**	RpsA	30S ribosomal protein S1	Protein biosynthesis	0.34	0.53	0.34	0.46	-0.09	0.03	0.03	–	–
**SAOUHSC_01625**	Efp	elongation factor P	Protein biosynthesis	0.57	0.35	-0.31	-0.05	-1.08	0.52	-0.72	–	▼
**SAOUHSC_01668**	Era	GTP-binding protein Era	Protein biosynthesis	-0.25	0.21	0.28	0.22	n.d.	-1.59	-2.26	–	▼
**SAOUHSC_01678**	RpsU	30S ribosomal protein S21	Protein biosynthesis	-0.30	-0.82	-1.85	-1.53	-1.69	-0.98	-0.66	▼	▼
**SAOUHSC_01722**	AlaS	alanyl-tRNA synthetase	Protein biosynthesis	-0.02	-0.14	-0.61	-0.33	0.05	-0.28	-0.05	–	–
**SAOUHSC_01753**	ObgE	GTPase ObgE	Protein biosynthesis	-0.12	-0.37	-0.05	0.06	0.26	-0.48	-1.08	–	–
**SAOUHSC_01755**	RpmA	50S ribosomal protein L27	Protein biosynthesis	0.43	-0.71	-1.25	-1.12	-1.55	-0.90	-1.33	▼	▼
**SAOUHSC_01757**	RplU	50S ribosomal protein L21	Protein biosynthesis	-0.35	-0.95	-1.22	-1.14	-0.18	-0.46	-0.74	▼	–
**SAOUHSC_01788**	ThrS	threonyl-tRNA synthetase	Protein biosynthesis	0.11	0.01	-0.43	-0.45	-0.17	-0.08	-0.22	–	–
**SAOUHSC_01829**	RpsD	30S ribosomal protein S4	Protein biosynthesis	-0.50	-0.84	-0.90	-1.12	-0.03	0.13	-0.21	▼	–
**SAOUHSC_01982**		ribosomal large subunit pseudouridine synthase D	Protein biosynthesis	-0.27	-0.02	-0.40	-0.39	0.11	0.45	n.d.	–	–
**SAOUHSC_02116**	GatB	aspartyl/glutamyl-tRNA amidotransferase subunit B	Protein biosynthesis	0.29	0.27	-0.05	-0.03	-0.24	-0.23	-0.26	–	–
**SAOUHSC_02117**	GatA	aspartyl/glutamyl-tRNA amidotransferase subunit A	Protein biosynthesis	0.29	0.17	0.09	0.03	-0.42	-0.39	-0.26	–	–
**SAOUHSC_02361**	RpmE2	50S ribosomal protein L31 type B	Protein biosynthesis	-0.03	-0.81	-1.19	-1.14	-0.36	-1.60	-0.32	▼	–
**SAOUHSC_02477**	RpsI	30S ribosomal protein S9	Protein biosynthesis	-0.41	-0.60	-1.23	-1.38	-0.97	-0.88	-1.06	▼	▼
**SAOUHSC_02478**	RplM	50S ribosomal protein L13	Protein biosynthesis	0.13	-0.42	-0.59	-0.52	0.93	0.35	0.80	–	▲
**SAOUHSC_02484**	RplQ	50S ribosomal protein L17	Protein biosynthesis	0.48	-0.34	-0.23	-0.33	0.37	-0.01	-0.36	–	–
**SAOUHSC_02486**	RpsK	30S ribosomal protein S11	Protein biosynthesis	-0.37	-0.98	-1.59	-1.79	-0.79	-0.78	-1.11	▼	▼
**SAOUHSC_02487**	RpsM	30S ribosomal protein S13	Protein biosynthesis	-0.33	-1.10	-1.22	-1.29	0.03	0.24	-0.04	▼	–
**SAOUHSC_02489**	InfA	translation initiation factor IF-1	Protein biosynthesis	0.28	-0.02	0.01	0.02	-0.67	-2.24	-2.64	–	▼
**SAOUHSC_02492**	RplO	50S ribosomal protein L15	Protein biosynthesis	-0.22	-1.00	-1.33	-1.38	-0.69	-1.06	-2.19	▼	▼
**SAOUHSC_02493**	RpmD	50S ribosomal protein L30	Protein biosynthesis	0.51	-0.32	-0.73	-0.53	-0.55	-2.84	-3.21	–	▼
**SAOUHSC_02495**	RplR	50S ribosomal protein L18	Protein biosynthesis	-0.03	-0.61	-0.95	-0.93	0.43	-0.98	-1.40	▼	▼
**SAOUHSC_02496**	RplF	50S ribosomal protein L6	Protein biosynthesis	0.13	-0.53	-0.65	-0.63	0.01	-0.36	0.51	▼	–
**SAOUHSC_02498**	RpsH	30S ribosomal protein S8	Protein biosynthesis	0.36	-0.22	-0.86	-0.82	-0.16	-0.47	-0.75	▼	–
**SAOUHSC_02500**	RplE	50S ribosomal protein L5	Protein biosynthesis	-0.08	-0.57	-0.47	-0.55	-0.05	0.75	0.29	–	–
**SAOUHSC_02503**	RpsQ	30S ribosomal protein S17	Protein biosynthesis	0.33	-0.56	-1.41	-1.54	-0.10	1.62	-0.41	▼	–

locus tag	protein symbol	description	theSEED functional category	S9 cell line infection model				murine pneumonia model			median over 8,24 and 32h ratio	
				exp.	8h	24h	32h	8h	24h	32h	median tendence (S9) - 1.5fc cutoff	median tendence (mice) - 1.5fc cutoff
**SAOUHSC_02504**	RpmC	50S ribosomal protein L29	Protein biosynthesis	0.07	-0.77	-1.20	-1.11	-0.15	-0.57	-0.89	▼	–
**SAOUHSC_02505**	RplP	50S ribosomal protein L16	Protein biosynthesis	-0.60	-1.07	-1.50	-1.37	1.49	3.47	1.21	▼	▲
**SAOUHSC_02506**	RpsC	30S ribosomal protein S3	Protein biosynthesis	-0.18	-0.97	-0.70	-0.80	-0.68	-0.15	-0.34	▼	–
**SAOUHSC_02507**	RplV	50S ribosomal protein L22	Protein biosynthesis	-0.36	-0.77	-0.77	-0.96	-0.78	-0.53	-0.68	▼	▼
**SAOUHSC_02509**	RplB	50S ribosomal protein L2	Protein biosynthesis	-0.30	-0.84	-1.04	-1.05	1.12	-0.10	-0.19	▼	–
**SAOUHSC_02510**	RplW	50S ribosomal protein L23	Protein biosynthesis	-0.05	-0.55	-0.81	-0.67	-4.25	-1.47	0.70	▼	▼
**SAOUHSC_02511**	RplD	50S ribosomal protein L4	Protein biosynthesis	-0.29	-0.85	-1.42	-1.10	-0.15	-0.54	-0.68	▼	–
**SAOUHSC_02512**	RplC	50S ribosomal protein L3	Protein biosynthesis	-0.46	-0.70	-1.24	-0.75	0.47	0.67	0.27	▼	–
**SAOUHSC_01207**	Ffh	signal recognition particle protein	Protein biosynthesis;Cell Division and Cell Cycle - no subcategory	-0.13	-0.47	-0.61	-0.53	-0.43	-0.22	-0.16	–	–
**SAOUHSC_01038**	Def	peptide deformylase	Protein biosynthesis;Central carbohydrate metabolism	0.74	-0.04	-0.10	-0.01	-0.42	0.28	0.36	–	–
**SAOUHSC_01183**	Fmt	methionyl-tRNA formyltransferase	Protein biosynthesis;Folate and pterines	0.28	-0.01	0.09	0.25	-0.37	-0.51	-1.03	–	–
**SAOUHSC_01688**	LepA	GTP-binding protein LepA	Protein biosynthesis;Heat shock;Tetrapyrroles;Protein biosynthesis	-0.38	-0.26	-0.52	-0.47	-0.52	-0.56	n.d.	–	–
**SAOUHSC_02359**	PrfA	peptide chain release factor 1	Protein biosynthesis;Oxidative stress	0.07	0.06	-0.34	-0.21	0.23	-0.64	0.72	–	–
**SAOUHSC_01246**	InfB	translation initiation factor IF-2	Protein biosynthesis;Protein biosynthesis	0.15	-0.07	0.44	0.54	0.18	0.02	-0.04	–	–
**SAOUHSC_00529**	FusA	elongation factor G	Protein biosynthesis;Protein biosynthesis;Virulence - no subcategory;Protein biosynthesis	-0.06	-0.27	-0.37	-0.16	-0.23	-0.36	-0.48	–	–
**SAOUHSC_00509**	GltX	glutamyl-tRNA synthetase	Protein biosynthesis;Tetrapyrroles	0.29	0.10	-0.24	-0.01	0.05	-0.41	-0.14	–	–
**SAOUHSC_00505**	ClpC	endopeptidase	Protein degradation	0.15	-0.48	-0.19	0.22	0.04	-0.10	0.17	–	–
**SAOUHSC_01626**	PepQ2	proline dipeptidase	Protein degradation	0.81	0.87	0.29	0.55	0.67	-0.10	0.99	–	▲
**SAOUHSC_02092**	AmpS	aminopeptidase PepS	Protein degradation	0.47	0.04	-0.00	0.03	0.06	0.04	0.29	–	–
**SAOUHSC_02102**	Map	methionine aminopeptidase	Protein degradation;Protein biosynthesis	0.69	0.03	-0.03	-0.16	-0.54	-1.34	-1.28	–	▼
**SAOUHSC_01226**	HslU	ATP-dependent protease ATP-binding subunit HslU	Protein degradation;Protein degradation	-0.14	-0.05	0.15	-0.02	0.90	1.09	0.48	–	▲
**SAOUHSC_01778**	ClpX	ATP-dependent protease ATP-binding subunit ClpX	Protein degradation;Protein degradation	-0.24	-0.72	-1.53	-1.66	-0.38	-0.65	-1.17	▼	▼
**SAOUHSC_00790**	ClpP	ATP-dependent Clp protease proteolytic subunit	Protein degradation;Protein degradation;Regulation and Cell signaling - no subcategory	0.07	-0.13	-0.16	-0.03	-0.60	-0.81	-0.87	–	▼
**SAOUHSC_01972**	PrsA	protein export protein PrsA	Protein folding	0.05	2.19	1.78	1.37	-0.58	-3.11	n.d.	▲	▼
**SAOUHSC_02255**	GroES	co-chaperonin GroES	Protein folding	0.45	-0.08	0.02	-0.20	0.69	0.43	0.37	–	–
**SAOUHSC_01682**	DnaJ	chaperone protein DnaJ	Protein folding;Heat shock	-0.08	-0.45	-0.60	-0.44	n.d.	0.17	-0.33	–	–
**SAOUHSC_01684**	GrpE	heat shock protein GrpE	Protein folding;Heat shock	0.34	-0.30	-0.39	-0.36	-0.13	0.01	-0.34	–	–
**SAOUHSC_00912**	ClpB	ATP-dependent Clp protease, ATP-binding subunit ClpB	Protein folding;Protein degradation	0.01	-0.75	1.10	1.79	0.70	1.22	1.80	▲	▲
**SAOUHSC_00903**	SpsB	Signal peptidase IB	Protein processing and modification	-0.20	0.54	0.12	0.01	-0.50	-1.03	-1.28	–	▼
**SAOUHSC_01432**	MsrA2	methionine sulfoxide reductase A	Protein processing and modification	0.27	2.57	1.65	1.53	1.18	0.54	0.48	▲	–
**SAOUHSC_02494**	RpsE	30S ribosomal protein S5	Protein processing and modification;Protein biosynthesis	-0.01	-0.60	-1.11	-1.28	0.11	-0.16	-0.19	▼	–
**SAOUHSC_00527**	RpsL	30S ribosomal protein S12	Protein processing and modification;Virulence - no subcategory;Protein biosynthesis	0.46	-0.76	-0.76	-1.02	-0.77	-0.59	-1.20	▼	▼
**SAOUHSC_00257**	EsxA	hypothetical protein	Protein secretion system, Type VII	0.16	0.31	0.67	1.16	0.68	0.43	1.05	▲	▲
**SAOUHSC_00374**	GuaB	inosine-5-monophosphate dehydrogenase	Purines	-0.05	-0.23	-0.96	-0.75	0.21	-0.20	0.36	▼	–
**SAOUHSC_00467**	PurR	pur operon repressor	Purines	-0.20	0.20	0.13	0.17	0.63	1.16	0.16	–	▲
**SAOUHSC_00485**	Hpt	hypoxanthine phosphoribosyltransferase	Purines	0.06	-0.23	-0.18	-0.08	-0.26	-0.56	-0.87	–	–
**SAOUHSC_00539**		hypothetical protein	Purines	0.30	-0.41	-0.60	-0.38	-0.17	-0.63	1.77	–	–
**SAOUHSC_01008**	PurE	phosphoribosylaminoimidazole carboxylase, catalytic subunit	Purines	0.02	0.46	-0.65	-0.31	-0.78	-0.39	-1.12	–	▼
**SAOUHSC_01009**	PurK	phosphoribosylaminoimidazole carboxylase ATPase subunit	Purines	-0.21	0.34	-0.77	-0.70	-0.52	0.09	-0.58	▼	–
**SAOUHSC_01010**	PurC	phosphoribosylaminoimidazole-succinocarboxamide synthase	Purines	-0.01	0.39	-0.97	-0.69	0.20	0.42	0.17	▼	–
**SAOUHSC_01011**	PurS	phosphoribosylformylglycinamidine synthase, PurS protein	Purines	0.27	0.73	-0.84	-0.71	1.17	0.27	-0.05	▼	–
**SAOUHSC_01012**	PurQ	phosphoribosylformylglycinamidine synthase I	Purines	0.14	0.54	-0.84	-0.71	-0.80	-0.49	-0.86	▼	▼
**SAOUHSC_01013**	PurL	phosphoribosylformylglycinamidine synthase II	Purines	-0.09	0.49	-0.31	-0.37	0.09	-0.10	-0.22	–	–
**SAOUHSC_01015**	PurM	phosphoribosylaminoimidazole synthetase	Purines	0.09	0.63	-0.56	-0.34	n.d.	0.10	0.22	–	–
**SAOUHSC_01017**	PurH	bifunctional phosphoribosylaminoimidazolecarboxamide formyltransferase/IMP cyclohydrolase	Purines	-0.58	-0.17	-0.97	-0.91	-0.68	-0.02	-0.05	▼	–
**SAOUHSC_01330**	GuaC	guanosine 5-monophosphate oxidoreductase	Purines	-0.63	-0.62	-0.86	-0.67	0.50	-0.37	0.50	▼	–
**SAOUHSC_02490**	Adk	adenylate kinase	Purines	0.36	0.02	-0.15	0.01	-1.61	0.21	-0.78	–	▼
**SAOUHSC_00472**	Prs	ribose-phosphate pyrophosphokinase	Purines;Central carbohydrate metabolism	-0.59	-0.01	-0.45	-0.41	0.40	-0.09	0.00	–	–

locus tag	protein symbol	description	theSEED functional category	S9 cell line infection model				murine pneumonia model			median over 8,24 and 32h ratio	
				exp.	8h	24h	32h	8h	24h	32h	median tendence (S9) - 1.5fc cutoff	median tendence (mice) - 1.5fc cutoff
SAOUHSC_00019	PurA	adenylosuccinate synthetase	Purines;Histidine Metabolism;Miscellaneous - no subcategory	0.07	0.04	-0.40	-0.27	0.38	0.05	-0.20	–	–
SAOUHSC_00375	GuaA	GMP synthase	Purines;Pathogenicity islands	0.31	0.29	-0.58	-0.36	-0.17	-0.15	-0.17	–	–
SAOUHSC_00372	Xpt	xanthine phosphoribosyltransferase	Purines;Purines	-0.03	-0.37	-1.01	-0.88	0.13	0.37	0.27	▼	–
SAOUHSC_02126	PurB	adenylosuccinate lyase	Purines;Purines	0.01	-0.30	-0.62	-0.48	-0.61	-0.02	-0.44	–	–
SAOUHSC_01485	Ndk	nucleoside diphosphate kinase	Purines;Pyrimidines	0.42	0.28	1.11	1.12	0.19	-0.24	n.d.	▲	–
SAOUHSC_01743	Apt	adenine phosphoribosyltransferase	Purines;Regulation and Cell signaling - no subcategory;Regulation and Cell signaling - no subcategory	0.44	-0.35	0.41	0.37	n.d.	0.42	0.37	–	–
SAOUHSC_00499	PdxS	pyridoxal biosynthesis lyase PdxS	Pyridoxine	-0.41	-0.02	-0.43	-0.31	n.d.	0.99	0.41	–	▲
SAOUHSC_00451	Tmk	thymidylate kinase	Pyrimidines	0.20	0.40	-0.11	0.12	n.d.	0.36	n.d.	–	–
SAOUHSC_01166	PyrB	aspartate carbamoyltransferase catalytic subunit	Pyrimidines	-0.71	-0.87	-0.72	-0.57	n.d.	-0.42	n.d.	▼	–
SAOUHSC_01168	PyrC	dihydroorotase	Pyrimidines	-0.14	-0.03	0.01	0.06	0.22	-1.57	-0.99	–	▼
SAOUHSC_01169	CarA	carbamoyl phosphate synthase small subunit	Pyrimidines	-0.86	-0.76	-0.52	-0.42	n.d.	0.21	1.44	–	▲
SAOUHSC_01170	CarB	carbamoyl phosphate synthase large subunit	Pyrimidines	-0.78	-0.81	-0.88	-0.66	-0.61	-0.41	-0.67	▼	▼
SAOUHSC_01172	PyrE	orotate phosphoribosyltransferase	Pyrimidines	0.01	-0.31	-0.48	-0.18	-0.70	0.73	0.65	–	▲
SAOUHSC_01496	Cmk	cytidylate kinase	Pyrimidines	0.24	-0.38	-0.51	-0.37	0.96	-0.09	-0.14	–	–
SAOUHSC_01715	Udk	uridine kinase	Pyrimidines	-0.25	-0.19	-0.58	-0.61	-0.49	-0.58	-0.90	▼	▼
SAOUHSC_02377	Pdp	pyrimidine-nucleoside phosphorylase	Pyrimidines;Monosaccharides	-0.16	1.14	1.87	2.10	1.84	2.08	1.65	▲	▲
SAOUHSC_01164	PyrR	bifunctional pyrimidine regulatory protein PyrR uracil phosphoribosyltransferase	Pyrimidines;Pyrimidines	0.08	-0.01	0.13	0.07	-0.24	-0.14	-0.00	–	–
SAOUHSC_02353	Upp	uracil phosphoribosyltransferase	Pyrimidines;Pyrimidines	-0.01	0.01	-0.03	-0.02	0.22	0.22	-0.39	–	–
SAOUHSC_00785	TrxB	thioredoxin reductase	Pyrimidines;Sulfur Metabolism - no subcategory	0.28	0.22	0.03	0.24	0.08	0.02	0.19	–	–
SAOUHSC_00980	MenA	1,4-dihydroxy-2-naphthoate octaprenyltransferase	Quinone cofactors;Quinone cofactors	-0.41	-0.32	-0.48	-0.59	0.98	1.71	n.d.	–	▲
SAOUHSC_00985	MenB	naphthoate synthase	Quinone cofactors;Quinone cofactors	0.27	0.20	-0.06	0.09	0.04	0.30	0.06	–	–
SAOUHSC_01487	UbiE	ubiquinone/menaquinone biosynthesis methyltransferase	Quinone cofactors;Quinone cofactors	-0.44	-0.14	-0.40	-0.57	n.d.	1.21	-0.74	–	–
SAOUHSC_00620	SarA	accessory regulator A	Quorum sensing and biofilm formation	0.25	0.08	-0.25	-0.39	1.88	1.25	1.37	–	▲
SAOUHSC_01850	CcpA	catabolite control protein A	Regulation and Cell signaling - no subcategory	0.11	0.33	0.32	0.27	0.33	0.18	-0.09	–	–
SAOUHSC_02374	HmrA	aminobenzoyl-glutamate utilization protein B	Resistance to antibiotics and toxic compounds	0.28	-0.32	-0.63	-0.16	n.d.	1.13	1.61	–	▲
SAOUHSC_02525		hypothetical protein	Resistance to antibiotics and toxic compounds	-0.14	-0.21	-0.31	-0.28	n.d.	-0.57	4.95	–	▲
SAOUHSC_01467	Pbp2	penicillin-binding protein 2	Resistance to antibiotics and toxic compounds;Cell Division and Cell Cycle - no subcategory;Cell Wall and Capsule - no subcategory	-0.20	-0.14	-0.45	-0.42	-1.41	n.d.	n.d.	–	▼
SAOUHSC_00954	MurE	UDP-N-acetylmuramoylalanyl-D-glutamate--L-lysine ligase	Resistance to antibiotics and toxic compounds;Cell Wall and Capsule - no subcategory	-0.24	-0.38	-0.77	-0.62	n.d.	1.24	n.d.	▼	▲
SAOUHSC_02527	FmhB	FmhB protein	Resistance to antibiotics and toxic compounds;Cell Wall and Capsule - no subcategory	-0.31	-0.23	-0.47	-0.52	n.d.	-0.14	-0.12	–	–
SAOUHSC_00869	DltA	D-alanine--poly(phosphoribitol) ligase subunit 1	Resistance to antibiotics and toxic compounds;Gram-Positive cell wall components;Gram-Positive cell wall components	0.32	-0.58	-0.54	-0.23	0.11	-0.23	-0.43	–	–
SAOUHSC_02582	FdhA	formate dehydrogenase subunit alpha	Respiration - no subcategory	0.38	-1.37	-1.06	-0.62	0.72	n.d.	-0.40	▼	–
SAOUHSC_01347	CitB	aconitate hydratase	Respiration - no subcategory;One-carbon Metabolism;Central carbohydrate metabolism	0.83	-0.31	0.25	0.69	0.35	0.32	0.49	–	–
SAOUHSC_01961	HemH	ferrochelatase	Respiration - no subcategory;Tetrapyrroles	0.07	0.21	0.53	0.59	-0.33	-0.22	-0.24	–	–
SAOUHSC_01248	TruB	tRNA pseudouridine synthase B	Riboflavin, FMN, FAD;RNA processing and modification	0.15	-0.42	-0.28	-0.10	0.39	0.01	0.53	–	–
SAOUHSC_00803	Rnr	ribonuclease R	RNA processing and modification	-0.38	-0.46	-0.32	-0.17	-0.37	-0.47	n.d.	–	–
SAOUHSC_00951		hypothetical protein	RNA processing and modification	0.30	1.11	2.45	2.54	0.83	1.14	1.64	▲	▲
SAOUHSC_01659		hypothetical protein	RNA processing and modification	-0.42	-0.72	-0.41	-0.43	-0.28	-0.29	-0.17	–	–
SAOUHSC_01748	Tgt	queuine tRNA-ribosyltransferase	RNA processing and modification	-0.34	-0.34	-0.36	-0.34	n.d.	-1.22	-0.84	–	▼
SAOUHSC_01749	QueA	S-adenosylmethionine:tRNA ribosyltransferase-isomerase	RNA processing and modification	-0.12	-0.25	-0.52	-0.90	n.d.	-0.43	n.d.	–	–
SAOUHSC_02097		hypothetical protein	RNA processing and modification	0.32	-1.11	-0.99	-0.72	0.11	0.27	0.19	▼	–
SAOUHSC_02316	CshA	DEAD-box ATP dependent DNA helicase	RNA processing and modification	-0.67	-1.07	-0.99	-0.90	-0.33	-0.38	-0.51	▼	–
SAOUHSC_00721	QueC	hypothetical protein	RNA processing and modification;Miscellaneous - no subcategory	0.79	-1.23	-0.37	-0.24	0.28	0.51	n.d.	–	–
SAOUHSC_01247	RbfA	ribosome-binding factor A	RNA processing and modification;Protein biosynthesis	0.34	0.75	0.28	0.51	n.d.	-0.09	n.d.	–	–
SAOUHSC_00074	SirA	periplasmic binding protein	Siderophores	-2.39	0.12	-0.03	-0.15	0.02	-1.76	n.d.	–	▼
SAOUHSC_01742	RelA	GTP pyrophosphokinase	Signal transduction in Eukaryotes;Regulation and Cell signaling - no subcategory	0.01	-0.23	-0.12	0.00	1.05	0.32	0.67	–	▲
SAOUHSC_01413		hypothetical protein	Stress Response - no subcategory	0.10	0.03	-0.41	-0.59	-0.45	-0.10	-0.81	–	–
SAOUHSC_01814	UspA2	hypothetical protein	Stress Response - no subcategory	0.08	0.37	0.54	0.75	0.70	1.08	1.36	–	▲

locus tag	protein symbol	description	theSEED functional category	S9 cell line infection model				murine pneumonia model			median over 8,24 and 32h ratio	
				exp.	8h	24h	32h	8h	24h	32h	median tendence (S9) - 1.5fc cutoff	median tendence (mice) - 1.5fc cutoff
**SAOUHSC_01819**	UspA1	hypothetical protein	Stress Response - no subcategory	0.30	1.46	2.61	3.08	3.39	2.90	2.99	▲	▲
**SAOUHSC_02299**	RsbW	serine-protein kinase RsbW	Stress Response - no subcategory	0.28	-0.57	-0.92	-0.53	-0.37	-0.42	-0.97	–	–
**SAOUHSC_02300**	RsbV	STAS domain-containing protein	Stress Response - no subcategory	0.46	-0.42	-0.41	-0.16	-0.27	-0.03	-0.06	–	–
**SAOUHSC_02301**	RsbU	sigmaB regulation protein RsbU	Stress Response - no subcategory	0.25	-0.28	-0.26	-0.08	0.57	0.20	n.d.	–	–
**SAOUHSC_02302**		sigmaB regulation protein RsbU	Stress Response - no subcategory	0.59	0.21	0.22	0.08	-0.10	0.20	0.78	–	–
**SAOUHSC_02430**	HtsA	ABC transporter periplasmic binding protein	Stress Response - no subcategory;Iron acquisition and metabolism - no subcategory	-1.87	-0.05	0.10	0.26	-0.03	-0.64	-0.21	–	–
**SAOUHSC_02298**	SigB	RNA polymerase sigma factor SigB	Stress Response - no subcategory;Transcription;Quorum sensing and biofilm formation;Resistance to antibiotics and toxic compounds	0.42	-0.54	-0.98	-0.77	n.d.	-0.78	0.52	▼	–
**SAOUHSC_01071**		glycerophosphoryl diester phosphodiesterase	Sugar alcohols	-0.01	0.35	0.55	0.48	n.d.	0.78	n.d.	–	▲
**SAOUHSC_02402**	MtlA	PTS system mannitol-specific transporter subunit IIA	Sugar alcohols	0.91	-0.64	0.13	0.36	2.20	1.94	2.40	–	▲
**SAOUHSC_02403**	MtlD	mannitol-1-phosphate 5-dehydrogenase	Sugar alcohols	0.87	-0.38	0.23	0.51	0.14	0.71	0.67	–	▲
**SAOUHSC_01276**	GlpK	glycerol kinase	Sugar alcohols;Phospholipids;Central carbohydrate metabolism	0.19	0.82	0.80	0.84	0.14	0.13	0.29	▲	–
**SAOUHSC_00781**	HprK	HPr kinase/phosphorylase	Sugar alcohols;Regulation and Cell signaling - no subcategory	-0.06	0.29	-0.22	-0.17	n.d.	0.74	0.61	–	▲
**SAOUHSC_00364**	AhpF	alkyl hydroperoxide reductase subunit F	Sulfur Metabolism - no subcategory	1.03	0.16	0.85	1.04	-0.04	-0.01	0.08	▲	–
**SAOUHSC_00365**	AhpC	alkyl hydroperoxide reductase subunit C	Sulfur Metabolism - no subcategory	1.00	-0.07	1.09	1.34	0.08	-0.77	-0.90	▲	▼
**SAOUHSC_01822**	Tpx	thiol peroxidase	Sulfur Metabolism - no subcategory	0.71	0.58	0.49	0.72	0.76	0.53	0.22	–	–
**SAOUHSC_01999**		bacterioferritin comigratory protein	Sulfur Metabolism - no subcategory	0.87	1.07	1.04	1.31	-0.24	0.23	1.19	▲	–
**SAOUHSC_00573**	HemQ	putative heme peroxidase	Tetrapyrroles	0.14	0.53	0.11	0.35	0.36	0.05	0.42	–	–
**SAOUHSC_01771**	HemL	glutamate-1-semialdehyde aminotransferase	Tetrapyrroles	0.21	0.02	-0.31	-0.13	-0.03	-0.26	-0.35	–	–
**SAOUHSC_01772**	HemB	delta-aminolevulinic acid dehydratase	Tetrapyrroles	0.05	0.06	-0.29	0.03	2.89	2.67	2.54	–	▲
**SAOUHSC_01774**	HemC	porphobilinogen deaminase	Tetrapyrroles	0.25	0.20	-0.08	0.17	n.d.	-0.79	n.d.	–	▼
**SAOUHSC_01962**	HemE	uroporphyrinogen decarboxylase	Tetrapyrroles	0.20	-0.24	-0.05	-0.08	-1.41	1.21	n.d.	–	–
**SAOUHSC_02000**	GsaB	glutamate-1-semialdehyde aminotransferase	Tetrapyrroles	0.48	0.44	0.42	0.67	-0.60	-0.14	-0.97	–	▼
**SAOUHSC_00517**	NusG	transcription antitermination protein	Transcription	0.04	0.01	-0.36	-0.39	-0.37	-0.52	-0.57	–	–
**SAOUHSC_01177**	RpoZ	DNA-directed RNA polymerase subunit omega	Transcription	-0.22	-0.45	-1.01	-0.96	n.d.	-2.09	n.d.	▼	▼
**SAOUHSC_01243**	NusA	transcription elongation factor NusA	Transcription	0.06	0.02	-0.19	-0.07	-0.10	-0.29	-0.48	–	–
**SAOUHSC_01621**	NusB	transcription antitermination protein NusB	Transcription	0.10	0.12	-0.66	-0.39	-0.40	-0.51	-0.30	–	–
**SAOUHSC_01662**	SigA	RNA polymerase sigma factor RpoD	Transcription	-0.13	-0.22	-0.60	-0.31	-1.09	0.17	-0.27	–	–
**SAOUHSC_01714**	GreA	transcription elongation factor GreA	Transcription	0.70	0.49	-0.34	-0.08	-0.06	-0.55	-0.80	–	–
**SAOUHSC_02362**	Rho	transcription termination factor Rho	Transcription	-0.82	0.05	-0.16	-0.30	n.d.	-1.45	-1.48	–	▼
**SAOUHSC_02369**	RpoE	DNA-directed RNA polymerase subunit delta	Transcription	-0.05	-0.24	-0.83	-0.74	-0.42	-0.36	-0.40	▼	–
**SAOUHSC_02485**	RpoA	DNA-directed RNA polymerase subunit alpha	Transcription	-0.49	-0.57	-0.85	-0.65	-0.03	0.35	-0.03	▼	–
**SAOUHSC_00524**	RpoB	DNA-directed RNA polymerase subunit beta	Transcription;Virulence - no subcategory	-0.29	-0.43	-0.59	-0.53	-0.31	-0.13	-0.14	–	–
**SAOUHSC_00886**	MnhD	putative monovalent cation/H+ antiporter subunit D	Uni- Sym- and Antiporters	-0.47	-0.18	-0.82	-0.83	n.d.	0.36	n.d.	▼	–
**SAOUHSC_00528**	RpsG	30S ribosomal protein S7	Virulence - no subcategory;Protein biosynthesis	-0.20	-1.01	-1.70	-1.93	-0.40	-0.27	-0.43	▼	–
**SAOUHSC_00530**	Tuf	elongation factor Tu	Virulence - no subcategory;Protein biosynthesis;Protein biosynthesis	-0.24	-0.22	-0.31	-0.34	-0.25	-0.10	-0.30	–	–
**SAOUHSC_00525**	RpoC	DNA-directed RNA polymerase subunit beta	Virulence - no subcategory;Transcription	-0.31	-0.35	-0.51	-0.49	-0.47	-0.07	-0.52	–	–
**SAOUHSC_00015**		hypothetical protein		-0.09	-0.25	0.33	0.25	1.29	0.67	n.d.	–	▲
**SAOUHSC_00070**	SarS	accessory regulator-like protein		0.29	-2.05	-0.89	-0.65	n.d.	-1.77	-0.18	▼	▼
**SAOUHSC_00228**	TarS	hypothetical protein		-0.36	0.03	-0.42	-0.33	-1.42	-1.01	0.44	–	▼
**SAOUHSC_00356**		hypothetical protein		0.92	-1.85	-1.13	-0.82	-0.60	-1.04	-1.81	▼	▼
**SAOUHSC_00369**		hypothetical protein		-0.18	-0.06	-0.36	-0.35	0.25	-0.29	-0.70	–	–
**SAOUHSC_00371**		hypothetical protein		0.57	1.78	2.73	2.91	1.29	1.60	1.61	▲	▲
**SAOUHSC_00417**		hypothetical protein		0.22	0.54	0.53	0.55	n.d.	n.d.	-0.26	–	–
**SAOUHSC_00461**	MetS	methionyl-tRNA synthetase		-0.08	0.06	-0.60	-0.49	-0.39	-0.21	-0.53	–	–
**SAOUHSC_00486**	FtsH	hypothetical protein		0.16	-0.40	-0.30	-0.33	-0.53	-0.20	-0.38	–	–
**SAOUHSC_00493**	LysS	lysyl-tRNA synthetase		0.18	0.04	-0.18	-0.09	-0.34	0.28	-0.05	–	–
**SAOUHSC_00533**	HchA	chaperone protein HchA		0.78	-0.08	-0.13	-0.02	-0.04	-0.23	0.58	–	–

locus tag	protein symbol	description	theSEED functional category	S9 cell line infection model				murine pneumonia model			median over 8,24 and 32h ratio	
				exp.	8h	24h	32h	8h	24h	32h	median tendence (S9) - 1.5fc cutoff	median tendence (mice) - 1.5fc cutoff
SAOUHSC_00542		hypothetical protein		0.11	-0.02	0.19	0.22	-0.52	0.16	0.11	–	–
SAOUHSC_00562	ThiD1	phosphomethylpyrimidine kinase		0.08	0.21	-0.23	-0.10	0.22	-0.01	-0.06	–	–
SAOUHSC_00605		hypothetical protein		0.08	-0.32	-0.21	-0.38	0.64	-0.47	1.02	–	▲
SAOUHSC_00616		hypothetical protein		0.28	0.00	0.56	0.40	-0.18	0.22	-0.05	–	–
SAOUHSC_00634	MntC	ABC transporter substrate-binding protein		-0.56	-1.94	-2.68	-3.14	-2.51	-3.14	n.d.	▼	▼
SAOUHSC_00637	MntA	hypothetical protein		-0.46	-0.85	-0.82	-0.88	-0.33	-0.66	-0.46	▼	–
SAOUHSC_00638	MntR	hypothetical protein		0.08	0.30	-0.04	0.17	n.d.	2.90	1.15	–	▲
SAOUHSC_00647	AbcA	hypothetical protein		0.28	0.29	1.38	1.45	n.d.	0.79	-0.26	▲	–
SAOUHSC_00669		hypothetical protein		-0.29	-0.52	-0.20	-0.04	0.65	0.89	-0.51	–	▲
SAOUHSC_00675		hypothetical protein		0.62	0.67	0.18	0.35	-0.48	0.05	0.12	–	–
SAOUHSC_00686		hypothetical protein		0.10	-0.09	-0.32	-0.29	-0.72	-0.28	-0.87	–	▼
SAOUHSC_00690		hypothetical protein		n.d.	n.d.	n.d.	n.d.	0.09	-0.06	-0.01	n.d.	–
SAOUHSC_00694	MgrA	hypothetical protein		0.35	0.83	0.00	-0.01	-0.10	-0.06	-0.09	–	–
SAOUHSC_00755		hypothetical protein		0.93	-0.11	-0.22	-0.11	n.d.	-0.53	-1.06	–	▼
SAOUHSC_00769	SecA	preprotein translocase subunit SecA		-0.23	-0.43	-0.15	-0.23	-0.18	0.03	0.21	–	–
SAOUHSC_00787		hypothetical protein		-0.01	0.29	-0.08	-0.05	-0.53	0.18	n.d.	–	–
SAOUHSC_00792		hypothetical protein		0.28	-0.56	0.23	0.46	3.81	3.04	-0.20	–	▲
SAOUHSC_00794	GapR	glycolytic operon regulator		-0.39	-0.66	-1.05	-1.07	4.64	n.d.	n.d.	▼	▲
SAOUHSC_00847	SufC	ABC transporter ATP-binding protein		0.28	-0.25	-0.02	0.07	-0.08	-0.36	-0.57	–	–
SAOUHSC_00848	SufD	hypothetical protein		0.31	-0.24	0.01	0.12	-0.33	0.24	-0.33	–	–
SAOUHSC_00850	SufU	hypothetical protein		0.98	-0.33	-0.17	0.02	-0.86	-0.92	n.d.	–	▼
SAOUHSC_00865		hypothetical protein		0.39	0.83	0.38	0.47	-0.20	-0.32	-0.06	–	–
SAOUHSC_00908	Cdr	coenzyme A disulfide reductase		-0.31	0.06	-0.17	-0.03	n.d.	1.03	0.88	–	▲
SAOUHSC_00909		hypothetical protein		-0.30	-0.01	-0.39	-0.36	0.53	-0.34	0.63	–	–
SAOUHSC_00934	SpxA	transcriptional regulator Spx		1.19	1.25	0.66	0.57	0.97	0.75	1.49	▲	▲
SAOUHSC_00937	PepF	oligoendopeptidase F		0.20	0.29	-0.15	-0.18	-0.69	-0.19	-0.21	–	–
SAOUHSC_00958	HtrA	serine protease HtrA		-0.49	-0.32	-0.22	-1.42	-0.24	0.42	0.28	–	–
SAOUHSC_00972		hypothetical protein		0.26	-0.90	-0.63	-0.32	-0.17	-0.03	-0.49	▼	–
SAOUHSC_01035	RnjA	hypothetical protein		0.02	-0.19	-0.01	0.12	0.54	-0.11	0.43	–	–
SAOUHSC_01036	RpoY	hypothetical protein		0.71	0.30	0.13	0.48	1.81	1.08	1.90	–	▲
SAOUHSC_01039		hypothetical protein		0.27	-0.01	-0.21	-0.27	-0.25	-1.90	-1.72	–	▼
SAOUHSC_01050		hypothetical protein		0.05	-0.64	-0.77	-0.75	-0.18	-0.42	-0.60	▼	–
SAOUHSC_01054		hypothetical protein		0.48	0.82	0.66	0.60	-0.14	-0.06	-0.42	▲	–
SAOUHSC_01061		hypothetical protein		0.21	0.56	0.76	0.58	-0.17	0.80	-0.13	–	–
SAOUHSC_01091		hypothetical protein		-0.40	0.06	-0.54	-0.64	-1.27	-1.95	n.d.	–	▼
SAOUHSC_01100	TrxA	thioredoxin		0.57	0.46	0.44	0.52	-0.34	-0.87	-0.52	–	–
SAOUHSC_01138		hypothetical protein		0.76	0.76	1.88	2.07	1.35	1.68	0.80	▲	▲
SAOUHSC_01143	MraW	S-adenosyl-methyltransferase MraW		-0.35	0.07	0.07	-0.10	-0.23	-0.14	-0.44	–	–
SAOUHSC_01159	IleS	isoleucyl-tRNA synthetase		0.05	-0.27	-0.54	-0.40	0.01	-0.08	-0.06	–	–
SAOUHSC_01186	Stp1	hypothetical protein		0.06	0.26	0.05	0.18	-0.47	0.66	n.d.	–	–
SAOUHSC_01187	PknB	hypothetical protein		-0.24	-0.38	-0.20	-0.37	2.30	-1.61	n.d.	–	–
SAOUHSC_01192		hypothetical protein		0.06	-0.52	-0.13	-0.17	-0.19	-0.69	0.18	–	–
SAOUHSC_01222	TopA	DNA topoisomerase I		-0.15	-0.80	-0.88	-0.81	-1.22	-0.97	n.d.	▼	▼
SAOUHSC_01223	Gid	tRNA (uracil-5-)-methyltransferase Gid		-0.10	-0.47	-0.48	-0.34	0.35	0.28	0.22	–	–
SAOUHSC_01228	CodY	transcriptional repressor CodY		0.33	0.44	0.21	0.47	0.26	-0.16	-0.29	–	–
SAOUHSC_01235	PyrH	uridylate kinase		-0.18	-0.55	-0.49	-0.51	0.03	-0.20	-0.16	–	–
SAOUHSC_01240	ProS	prolyl-tRNA synthetase		-0.01	0.22	-0.22	-0.26	-0.34	-0.52	-0.16	–	–

locus tag	protein symbol	description	theSEED functional category	S9 cell line infection model				murine pneumonia model			median over 8,24 and 32h ratio	
				exp.	8h	24h	32h	8h	24h	32h	median tendence (S9) - 1.5fc cutoff	median tendence (mice) - 1.5fc cutoff
SAOUHSC_01251	PnpA	polynucleotide phosphorylase/polyadenylase		-0.21	-0.71	-0.74	-0.66	0.01	-0.25	-0.19	▼	–
SAOUHSC_01252	RnjB	hypothetical protein		0.05	-0.68	-0.57	-0.58	-0.35	-0.39	-0.36	▼	–
SAOUHSC_01256		hypothetical protein		0.11	-0.20	-0.52	-0.32	n.d.	1.33	0.05	–	▲
SAOUHSC_01263	Rny|cvfA	phosphodiesterase		0.10	-0.60	-0.46	-0.45	-0.92	-0.72	-0.47	–	▼
SAOUHSC_01264		hypothetical protein		0.81	-0.17	-0.56	-0.39	-1.43	-0.98	-0.11	–	▼
SAOUHSC_01265		hypothetical protein		0.03	-0.51	-0.06	-0.03	n.d.	0.82	0.49	–	▲
SAOUHSC_01284		hypothetical protein		0.07	0.14	0.04	-0.03	n.d.	-0.24	n.d.	–	–
SAOUHSC_01317		hypothetical protein		0.02	0.63	0.32	0.21	-0.37	-0.58	n.d.	–	–
SAOUHSC_01323		hypothetical protein		0.40	0.61	0.14	0.55	-0.18	0.06	-0.47	–	–
SAOUHSC_01336		hypothetical protein		0.51	0.19	-0.69	-0.68	6.41	-1.06	-1.38	▼	▼
SAOUHSC_01349		hypothetical protein		0.59	1.48	0.65	0.93	n.d.	-0.84	n.d.	▲	▼
SAOUHSC_01354	AlsT	sodium:alanine symporter family protein		-0.18	-0.85	-0.20	-0.39	1.16	-0.28	n.d.	–	–
SAOUHSC_01383		hypothetical protein		0.30	0.69	0.25	0.22	-1.38	-1.71	n.d.	–	▼
SAOUHSC_01391	CvfB	hypothetical protein		-0.30	-0.34	-0.71	-0.49	n.d.	-0.42	-0.02	–	–
SAOUHSC_01392		ABC transporter ATP-binding protein		-0.19	-0.77	-0.73	-0.64	-0.03	-0.31	-0.54	▼	–
SAOUHSC_01408		hypothetical protein		0.41	0.74	0.32	0.29	n.d.	-1.18	n.d.	–	▼
SAOUHSC_01414		hypothetical protein		0.07	0.12	0.31	-0.04	-0.52	0.63	n.d.	–	–
SAOUHSC_01415		hypothetical protein		0.48	0.70	1.05	1.29	0.20	0.11	0.31	▲	–
SAOUHSC_01433	FakB2	hypothetical protein		0.20	0.24	0.17	0.47	-0.77	-0.04	-1.06	–	▼
SAOUHSC_01436		hypothetical protein		0.38	-0.11	-0.09	-0.12	-0.48	-0.59	-0.91	–	▼
SAOUHSC_01455		hypothetical protein		0.19	-0.03	-0.18	-0.16	0.49	0.32	0.40	–	–
SAOUHSC_01575		helix-turn-helix domain-containing protein		0.65	0.96	1.09	1.16	n.d.	0.92	n.d.	▲	▲
SAOUHSC_01586	SrrA	DNA-binding response regulator		0.41	0.47	0.26	0.42	0.50	-0.70	-1.27	–	▼
SAOUHSC_01604		hypothetical protein		0.44	0.50	-0.25	0.01	n.d.	-0.39	0.86	–	–
SAOUHSC_01606		peptidase T		0.51	0.79	1.00	1.17	-0.93	n.d.	3.41	▲	▲
SAOUHSC_01610		hypothetical protein		-0.09	0.01	-0.62	-0.80	n.d.	0.20	n.d.	▼	–
SAOUHSC_01661		hypothetical protein		0.37	-0.10	-0.43	-0.12	n.d.	1.08	n.d.	–	▲
SAOUHSC_01666	GlyS	glycyl-tRNA synthetase		0.66	-0.07	-0.17	-0.01	-0.39	-1.17	-0.36	–	–
SAOUHSC_01676		hypothetical protein		-0.26	0.26	0.32	0.21	-0.52	-0.72	-0.36	–	–
SAOUHSC_01698		hypothetical protein		0.08	-0.36	-0.82	-0.27	-0.39	-0.13	0.48	–	–
SAOUHSC_01729		hypothetical protein		n.d.	n.d.	n.d.	n.d.	0.26	-1.99	n.d.	n.d.	▼
SAOUHSC_01746	SecF	bifunctional preprotein translocase subunit SecD/SecF		-0.55	-0.23	-0.04	-0.11	-0.36	-0.77	-0.78	–	▼
SAOUHSC_01767	ValS	valyl-tRNA synthetase		0.29	-0.10	-0.31	-0.06	-0.27	-0.25	-0.15	–	–
SAOUHSC_01779	Tig	trigger factor		0.34	0.15	-0.14	-0.12	-0.04	0.03	-0.13	–	–
SAOUHSC_01782		hypothetical protein		0.51	0.83	0.28	0.23	-0.50	-1.22	n.d.	–	▼
SAOUHSC_01786	InfC	translation initiation factor IF-3		-0.33	-1.02	-1.15	-0.90	-0.33	0.14	-0.62	▼	–
SAOUHSC_01815		metal-dependent hydrolase		-0.41	-0.98	n.d.	0.58	-0.24	0.35	1.06	–	–
SAOUHSC_01816	PepQ	hypothetical protein		0.14	1.67	0.94	1.07	n.d.	n.d.	-0.54	▲	–
SAOUHSC_01839	TyrS	tyrosyl-tRNA synthetase		0.34	-0.07	-0.26	-0.13	-0.15	0.17	0.14	–	–
SAOUHSC_01854		hypothetical protein		0.24	-0.45	-0.11	0.10	0.85	-0.14	0.11	–	–
SAOUHSC_01855		hypothetical protein		-0.04	-0.38	0.29	0.30	0.26	-0.01	0.17	–	–
SAOUHSC_01858		hypothetical protein		0.33	-0.06	-0.13	-0.14	0.09	-0.33	-0.24	–	–
SAOUHSC_01860		hypothetical protein		0.66	0.68	0.68	0.69	-0.24	-0.37	-0.56	▲	–
SAOUHSC_01868		dipeptidase PepV		0.52	1.19	0.55	0.74	-0.02	0.04	-0.06	▲	–
SAOUHSC_01869		hypothetical protein		0.63	-0.10	0.15	0.28	-0.43	-0.70	-0.93	–	▼
SAOUHSC_01875	LeuS	leucyl-tRNA synthetase		0.47	0.43	-0.14	-0.01	-0.49	-0.47	-0.36	–	–
SAOUHSC_01895		hypothetical protein		-0.31	-0.31	-0.82	-0.95	0.33	n.d.	n.d.	▼	–

locus tag	protein symbol	description	theSEED functional category	S9 cell line infection model				murine pneumonia model			median over 8,24 and 32h ratio	
				exp.	8h	24h	32h	8h	24h	32h	median tendence (S9) - 1.5fc cutoff	median tendence (mice) - 1.5fc cutoff
SAOUHSC_01908		hypothetical protein		-0.11	-0.23	-0.08	-0.16	n.d.	0.98	2.84	–	▲
SAOUHSC_01964	TRAP	hypothetical protein		0.69	0.90	0.71	0.78	0.51	0.66	0.99	▲	▲
SAOUHSC_01968	Hit	hypothetical protein		0.74	0.81	1.13	1.46	0.48	0.44	0.80	▲	–
SAOUHSC_01969		hypothetical protein		-0.14	-0.01	0.01	0.40	1.19	0.64	1.03	–	▲
SAOUHSC_01973	Cbf1	3-5 exoribonuclease YhaM		-0.41	-0.47	-0.36	-0.55	n.d.	0.48	0.23	–	–
SAOUHSC_01974		hypothetical protein		-0.36	-0.20	-0.12	-0.04	0.25	0.28	1.15	–	–
SAOUHSC_01977		hypothetical protein		0.67	0.48	0.17	0.56	-0.08	-0.36	-0.62	–	–
SAOUHSC_01987		hypothetical protein		0.87	0.35	0.52	0.49	0.66	0.39	1.27	–	▲
SAOUHSC_02013		hypothetical protein		1.17	-0.68	0.45	0.82	0.84	1.19	0.88	–	▲
SAOUHSC_02073		hypothetical protein		n.d.	n.d.	n.d.	n.d.	2.53	n.d.	3.38	n.d.	▲
SAOUHSC_02098	VraR	DNA-binding response regulator VraR		0.62	2.09	1.26	1.16	0.42	1.26	3.60	▲	▲
SAOUHSC_02107		UDP-N-acetylmuramyl tripeptide synthetase		-0.41	-0.41	-0.47	-0.47	0.13	0.06	0.67	–	–
SAOUHSC_02108	FtnA	ferritin		0.54	-0.77	-0.05	0.18	0.23	-0.24	0.14	–	–
SAOUHSC_02114		putative lipid kinase		0.41	0.16	-0.27	0.07	-0.28	0.48	0.51	–	–
SAOUHSC_02145		hypothetical protein		0.56	-0.20	n.d.	0.83	-0.08	-0.55	-0.11	–	–
SAOUHSC_02150		hypothetical protein		0.95	1.19	1.54	1.83	-0.35	-0.14	-0.54	▲	–
SAOUHSC_02152	PmtC	ABC transporter ATP-binding protein		0.39	1.15	1.84	1.98	n.d.	0.52	-0.18	▲	–
SAOUHSC_02218		hypothetical protein		0.22	0.25	0.20	0.55	-0.43	-0.11	-0.06	–	–
SAOUHSC_02297		S1 RNA-binding domain-containing protein		-0.17	0.02	-0.48	-0.50	-0.44	-0.49	-0.40	–	–
SAOUHSC_02370		hypothetical protein		0.06	-0.17	-0.41	-0.43	n.d.	1.18	2.23	–	▲
SAOUHSC_02372		hypothetical protein		0.06	-0.05	-1.49	-1.66	n.d.	0.69	1.53	▼	▲
SAOUHSC_02373		hypothetical protein		0.58	-0.08	0.02	0.03	n.d.	2.72	n.d.	–	▲
SAOUHSC_02383		hypothetical protein		-0.02	-0.42	-0.58	-0.57	0.60	0.19	-0.78	–	–
SAOUHSC_02387		hypothetical protein		0.88	-0.45	0.42	0.73	0.46	0.12	0.17	–	–
SAOUHSC_02441	Asp23	alkaline shock protein 23		0.55	-0.84	0.29	0.78	0.70	0.33	0.43	–	–
SAOUHSC_02442		hypothetical protein		0.47	-0.46	0.57	0.76	n.d.	0.04	n.d.	–	–
SAOUHSC_02443	AmaP	hypothetical protein		0.34	-1.38	-0.24	0.17	1.96	0.61	1.15	–	▲
SAOUHSC_02460		hypothetical protein		0.53	0.82	0.21	0.26	0.41	0.30	-0.40	–	–
SAOUHSC_02491	SecY	preprotein translocase subunit SecY		-0.21	-0.66	-0.54	-0.56	-0.24	-0.38	n.d.	–	–
SAOUHSC_02554	FhuD2	hypothetical protein		-1.70	0.21	-0.28	-0.41	-0.90	-1.49	-1.56	–	▼
SAOUHSC_02566	SarR	hypothetical protein		-0.76	-0.05	-0.34	-0.25	3.71	0.33	0.05	–	–
SAOUHSC_02574		hypothetical protein		0.06	-0.22	-0.19	-0.10	-0.16	0.42	-0.04	–	–
SAOUHSC_02611	LyrA	hypothetical protein		0.24	-0.02	-0.42	-0.49	-1.05	-0.46	n.d.	–	▼
SAOUHSC_02666		hypothetical protein		-0.13	-0.21	-0.52	-0.42	n.d.	-1.06	n.d.	–	▼
SAOUHSC_02724		hypothetical protein		0.59	2.46	2.09	2.03	2.17	n.d.	n.d.	▲	▲
SAOUHSC_02747		hypothetical protein		0.46	1.22	0.59	0.79	n.d.	0.23	n.d.	▲	–
SAOUHSC_02820		hypothetical protein		-0.56	-2.08	-0.99	-0.69	0.12	0.36	0.56	▼	–
SAOUHSC_02829	Frp	NAD(P)H-flavin oxidoreductase		0.45	-0.04	1.41	1.65	1.11	-0.11	0.30	▲	–
SAOUHSC_02844		hypothetical protein		0.15	0.66	0.91	0.90	0.35	0.36	n.d.	▲	–
SAOUHSC_02862	ClpL	ATP-dependent Clp protease, ATP-binding subunit ClpC		0.53	1.09	1.15	1.52	0.37	-0.08	-0.34	▲	–
SAOUHSC_02895		hypothetical protein		-0.11	-0.59	-0.24	-0.06	n.d.	0.69	n.d.	–	▲
SAOUHSC_02900		hypothetical protein		0.92	-0.30	0.71	0.92	-0.44	-0.93	-2.43	▲	▼
SAOUHSC_02929		acetyl-CoA synthetase		0.24	0.20	-0.14	-0.05	0.11	0.16	-0.04	–	–
SAOUHSC_02972	IsaB	immunodominant antigen B		-0.56	2.44	3.68	3.90	2.35	n.d.	n.d.	▲	▲
SAOUHSC_02978		phage infection protein		-0.26	-0.73	-1.03	-0.93	1.24	0.77	n.d.	▼	▲
SAOUHSC_02980		hypothetical protein		0.39	0.92	0.75	0.83	n.d.	-0.45	0.78	▲	–
SAOUHSC_03022		hypothetical protein		0.64	-0.69	0.61	0.92	0.99	1.04	0.56	▲	▲

locus tag	protein symbol	description	theSEED functional category	S9 cell line infection model				murine pneumonia model			median over 8,24 and 32h ratio	
				exp.	8h	24h	32h	8h	24h	32h	median tendence (S9) - 1.5fc cutoff	median tendence (mice) - 1.5fc cutoff
**SAOUHSC_03052**	GidA	tRNA uridine 5-carboxymethylaminomethyl modification enzyme GidA		-0.27	-0.32	-0.55	-0.64	n.d.	1.18	0.09	–	▲

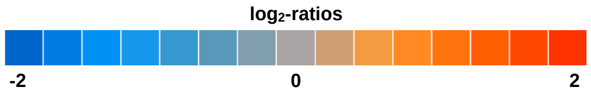

supplemental table S7:

locus	*S. aureus* strain	gene	description	localization (PSORT)	molar weight [Da] [µg/µmol] Uniprot	protein mix
SA0393	N315	*set15*	superantigen-like protein SET15	extracellular	25440	mix 2
SA0506	N315	*tuf*	elongation factor Tu	;ytoplasmic membrane	43104	mix 2
SA1063	N315	*prkC*	protein kinase	extracellular	74377	mix 2
SA1646	N315	*sei*	staphylococcal enterotoxin I	extracellular	27864	mix 2
SA1648	N315	*seo*	staphylococcal enterotoxin O	extracellular	29836	mix 2
SA1816	N315	*sel*	staphylococcal enterotoxin L	extracellular	27496	mix 2
SA1819	N315	*tsst-1*	toxic shock syndrom toxin-1	extracellular	26393	mix 2
SAA6159_01007	JKD6159	*efb*	extracellular finbrinogen binding protein	extracellular	12597	mix 2
SAB0363	RF122	*sec*	staphylococcal enterotoxin C	extracellular	31267	mix 2
SACOL0886	COL	*sek*	staphylococcal enterotoxin K	extracellular	27727	mix 2
SACOL0887	COL	*seq*	staphylococcal enterotoxin Q	extracellular	28185	mix 2
SACOL0908	COL	*SACOL0908*	hypothetical protein	unknown	20346	mix 2
SACOL1722	COL	*tig*	trigger factor	cytoplasmic	48609	mix 2
SACOL1802	COL	*SACOL1802*	hypothetical protein	unknown	15734	mix 2
SACOL2197	COL	*SACOL2197*	surface protein	;ytoplasmic membrane	15448	mix 2
SAKOR_01991	CN1	*fhuD2*	ferrichrome-binding protein	non available	34843	mix 2
SAOUHSC_00051	NCTC8325/HG001	*plc*	sphatidylinositol phosphodiesterase precursor, pu	extracellular	37087	mix 1
SAOUHSC_00081	NCTC8325/HG001	*SAOUHSC_00081*	aldolase	cytoplasmic	28588	mix 2
SAOUHSC_00192	NCTC8325/HG001	*coa*	coagulase	extracellular	71718	mix 1
SAOUHSC_00196	NCTC8325/HG001	*SAOUHSC_00196*	3-hydroxyacyl-CoA dehydrogenase protein	unknown	84608	mix 1
SAOUHSC_00257	NCTC8325/HG001	*SAOUHSC_00257*	ESAT-6 family virulence protein	extracellular	11036	mix 1
SAOUHSC_00300	NCTC8325/HG001	*geh*	lipase precursor	extracellular	76418	mix 1
SAOUHSC_00383	NCTC8325/HG001	*set6*	superantigen-like protein	extracellular	25647	mix 1
SAOUHSC_00384	NCTC8325/HG001	*set7*	superantigen-like protein	extracellular	26500	mix 1
SAOUHSC_00386	NCTC8325/HG001	*set8*	superantigen-like protein	extracellular	40181	mix 1
SAOUHSC_00389	NCTC8325/HG001	*SAOUHSC_00389*	superantigen-like protein	extracellular	34072	mix 1
SAOUHSC_00390	NCTC8325/HG001	*set10*	superantigen-like protein 5	extracellular	27170	mix 1
SAOUHSC_00391	NCTC8325/HG001	*SAOUHSC_00391*	superantigen-like protein	extracellular	26656	mix 1
SAOUHSC_00392	NCTC8325/HG001	*set11*	superantigen-like protein 7	extracellular	26168	mix 1
SAOUHSC_00393	NCTC8325/HG001	*SAOUHSC_00393*	superantigen-like protein	extracellular	26875	mix 1
SAOUHSC_00394	NCTC8325/HG001	*SAOUHSC_00394*	superantigen-like protein	extracellular	26676	mix 1
SAOUHSC_00399	NCTC8325/HG001	*set15*	superantigen-like protein	extracellular	25366	mix 1
SAOUHSC_00401	NCTC8325/HG001	*SAOUHSC_00401*	hypothetical protein SAOUHSC_00401	unknown	11302	mix 2
SAOUHSC_00427	NCTC8325/HG001	*aaa/sle1*	autolysin precursor, putative	cell wall	35836	mix 2
SAOUHSC_00749	NCTC8325/HG001	*SAUSA300_0721*	transferrin receptor	;ytoplasmic membrane	37854	mix 2
SAOUHSC_00795	NCTC8325/HG001	*SAOUHSC_00795*	ılyceraldehyde-3-phosphate dehydrogenase, type	cytoplasmic	36281	mix 2
SAOUHSC_00897	NCTC8325/HG001	*glpQ*	glycerophosphoryl diester phosphodiesterase	extracellular	35311	mix 1
SAOUHSC_00918	NCTC8325/HG001	*SAOUHSC_00918*	truncated MHC class II analog protein	;ytoplasmic membrane	15838	mix 2
SAOUHSC_01082	NCTC8325/HG001	*isdC*	LPXTG cell wall surface anchor protein	unknown	24855	mix 2
SAOUHSC_01114	NCTC8325/HG001	*fib*	fibrinogen-binding protein	extracellular	18765	mix 1
SAOUHSC_01115	NCTC8325/HG001	*SAOUHSC_01115*	fibrinogen-binding protein precursor-like protein	unknown	13114	mix 2
SAOUHSC_01121	NCTC8325/HG001	*hlY*	alpha-hemolysin precursor	extracellular	35973	mix 1
SAOUHSC_01653	NCTC8325/HG001	*sodA2*	superoxide dismutase, Mn, putative	extracellular	22711	mix 1
SAOUHSC_01683	NCTC8325/HG001	*dnaK*	DNAk protein, putative	cytoplasmic	66361	mix 1
SAOUHSC_01714	NCTC8325/HG001	*SAOUHSC_01714*	transcription elongation factor GreA	cytoplasmic	17743	mix 2
SAOUHSC_01935	NCTC8325/HG001	*splF*	serine protease SplF, putative	extracellular	25655	mix 1
SAOUHSC_01936	NCTC8325/HG001	*splE*	serine protease SplE	extracellular	25679	mix 2
SAOUHSC_01938	NCTC8325/HG001	*splD*	serine protease SplD	extracellular	25678	mix 2
SAOUHSC_01939	NCTC8325/HG001	*splC*	serine protease SplC	extracellular	26098	mix 2
SAOUHSC_01942	NCTC8325/HG001	*splA*	serine protease SplA	extracellular	25549	mix 2
SAOUHSC_01972	NCTC8325/HG001	*prsA*	protein export protein PrsA, putative	;ytoplasmic membrane	35638	mix 1
SAOUHSC_02003	NCTC8325/HG001	*grfA*	C transporter, ATP-binding/permease protein, puta	;ytoplasmic membrane	64863	mix 2
SAOUHSC_02169	NCTC8325/HG001	*chp*	chemotaxis-inhibiting protein CHIPS	unknown	17040	mix 1
SAOUHSC_02171	NCTC8325/HG001	*sak*	staphylokinase precursor, putative	extracellular	18504	mix 1
SAOUHSC_02254	NCTC8325/HG001	*groEL*	chaperonin, 60 kDa, GrpEL, putative	cytoplasmic	57630	mix 1
SAOUHSC_02257	NCTC8325/HG001	*sdrH*	sdrH protein	unknown	46620	mix 2
SAOUHSC_02260	NCTC8325/HG001	*hld*	hemolysin D	extracellular	2979	mix 2
SAOUHSC_02466	NCTC8325/HG001	*SAOUHSC_02466*	truncated MHC class II analog protein	;ytoplasmic membrane	15448	mix 1
SAOUHSC_02708	NCTC8325/HG001	*hlgA*	gamma-hemolysin h-gamma-ii subunit, putative	extracellular	34956	mix 2
SAOUHSC_02710	NCTC8325/HG001	*hlgB*	leukocidin f subunit precursor	extracellular	36711	mix 2
SAOUHSC_02802	NCTC8325/HG001	*fnbpB*	fibronectin binding protein B, putative	cell wall	101025	mix 2
SAOUHSC_02803	NCTC8325/HG001	*fnbA*	fibronectin binding protein A	cell wall	111780	mix 2
SAOUHSC_02887	NCTC8325/HG001	*isaA*	immunodominant antigen A, putative	extracellular	24203	mix 1
SAOUHSC_02972	NCTC8325/HG001	*isaB*	immunodominant antigen B	extracellular	19370	mix 1
SAOUHSC_03006	NCTC8325/HG001	*lip*	lipase	extracellular	76675	mix 1
SAP057A_011	plasmid SAP057A	*sspA*	serine proteinase SspA	non available	30765	mix 2
SAR0567	MRSA252	*bbp*	bone sialoprotein-binding protein	non available	123326	mix 2
SAUSA300_1065	USA300_FPR3757	*eta*	exofoliative toxin A	;ytoplasmic membrane	35310	mix 2
SAUSA300_1381	USA300_FPR3757	*lukF-PV*	Pantaon-Valentin-leukocidin, LukF-PV	extracellular	36962	mix 2
SAUSA300_1382	USA300_FPR3757	*lukS-PV*	Pantaon-Valentin-leukocidin, LukS-PV	extracellular	35313	mix 2
SAUSA300_1982	USA300_FPR3757	*groES*	chaperonin, GroES	cytoplasmic	57630	mix 2
SAUSA300_2296	USA300_FPR3757	*SAUSA300_2296*	esterase-like protein	cytoplasmic	35545	mix 2
USA300HOU_1167	USA300_TCH1516	*fabG*	3-oxoacyl-[acy-carrier-protein] reductase	cytoplasmic	26146	mix 2
